# WINPEPI (PEPI-for-Windows): computer programs for epidemiologists

**DOI:** 10.1186/1742-5573-1-6

**Published:** 2004-12-17

**Authors:** Joseph H Abramson

**Affiliations:** 1 School of Public Health and Community Medicine, Hebrew University, Jerusalem, Israel

## Abstract

**Background:**

The WINPEPI (PEPI-for-Windows) computer programs for epidemiologists are designed for use in practice and research in the health field and as learning or teaching aids. They aim to complement other statistics packages. The programs are free, and can be downloaded from the Internet.

**Implementation:**

There are at present four WINPEPI programs: DESCRIBE, for use in descriptive epidemiology, COMPARE2, for use in comparisons of two independent groups or samples, PAIRSetc, for use in comparisons of paired and other matched observations, and WHATIS, a "ready reckoner" utility program. The programs contain 75 modules, each of which provides a number, sometimes a large number, of statistical procedures. The manuals explain the uses, limitations and applicability of specific procedures, and furnish formulae and references.

**Conclusions:**

WINPEPI provides a wide variety of statistical routines commonly used by epidemiologists, and is a handy resource for many procedures that are not very commonly used or easily found. The programs are in general user-friendly, although some users may be confused by the large numbers of options and results provided. The main limitations are the inability to read data files and the fact that only one of the programs presents graphic results. WINPEPI has a considerable potential as a learning and teaching aid.

## Background

This paper describes the WINPEPI (PEPI-for-Windows) programs recently added to the PEPI suite of computer programs for epidemiologists, and discusses some of their uses and limitations. The programs were developed for use in practice and research in the health field and as learning or teaching aids.

PEPI (an acronym for *P*rograms for *EPI*demiologists) grew from a set of programs for programmable pocket calculators that was published in 1983 to "make life easier for investigators, extend the use of appropriate analytic methods, and enable researchers to concentrate on substantive issues rather than on procedural technicalities" [1]. The first version of PEPI appeared in 1993 [2], and was followed by version 2 (where the name "PEPI" was first used, in 1995) [3], version 3 (in 1999) [4], and version 4 (containing 43 programs, in 2001) [5].

The original programs were DOS-based. The first WINPEPI program, WHATIS, was included in version 4 of the PEPI package, a review of which stated: "WHATIS, the only Windows program, is our pick for the best program in PEPI. If all the programs could be converted into the WHATIS type of format, PEPI will be a truly outstanding package!" [6]. Four WINPEPI programs, containing 75 modules, have so far been issued. They provide many procedures not offered by the DOS-based programs, but do not include all those provided by the latter (which can be run in Windows as DOS applications, complementing the WINPEPI programs).

## Implementation

There are at present four WINPEPI programs, DESCRIBE, COMPARE2, PAIRSetc, and WHATIS. The programs are free, and can be downloaded from the Internet. New versions have been issued at frequent intervals. Comprehensive manuals are provided. These furnish full information about each module, including explanations of the uses, limitations and applicability of specific procedures, and formulae or references.

### DESCRIBE

DESCRIBE has 14 modules for use in descriptive epidemiology. It can appraise rates or proportions and categorical or numerical data (including survival data), examine a sequence of rates or other values (including the appraisal of seasonal variation), perform direct and indirect standardization, estimate prevalence from a cluster or stratified sample or by the capture-recapture method, determine required sample sizes, and appraise screening or diagnostic tests (with procedures for use in meta-analyses of studies of these tests).

### COMPARE2

COMPARE2 has 28 modules for use in comparisons of two independent groups or samples, and may be used to analyze cross-sectional, cohort and case-control studies, and trials. It can compare proportions or odds, risks, rates, and categorical and numerical data (including survival data), appraise the effect of misclassification, and determine power and sample size for a variety of tests.

The program can deal with stratified data, analyzing the combined strata as well as each stratum; it permits the control of possible confounding by the stratifying variable or variables, and the assessment of heterogeneity as an indication of effect modification. It can be used in meta-analyses, to compare study results and, if warranted, combine them.

### PAIRSetc

PAIRSetc has 29 modules for use in comparisons of paired and other matched observations, such as matched-control trials and cohort studies, matched case-control studies, before-after studies, and reliability studies that compare replicate observations or methods of measurement. The "etc" in its name indicates the program's ability to deal with matched sets larger than pairs. The program can compare dichotomous, categorical and numerical data (including paired survival data), appraise the effect of misclassification, and determine power and sample size for a variety of tests and for measuring *kappa *or intraclass correlation coefficients. Like COMPARE2, PAIRSetc can deal with stratified data.

### WHATIS

WHATIS is a "ready reckoner" utility program with four modules. It provides a calculator (expression evaluator) that stores values and formulae, enabling them to be recalled when needed, and it computes confidence intervals for a variety of statistics, P-values corresponding to given values of *z*, *t*, chi-square, or *F*, or vice versa, and time-spans.

## The modules

Some of the modules have very specific purposes; for example, to determine the sample size required to perform a specific test with a given power or precision, or to appraise the effect of misclassification in a given situation by computing the "true" findings that would give rise to the observed findings. Other modules provide many statistical procedures, as is illustrated by the following summaries of what two of the richer modules do. A module may not only provide numerous tests and measures, it may also use alternative methods of estimation.

### Comparison of proportions or odds (module A of COMPARE2)

After entry of a 2 × 2 table, this module provides exact one-tailed and two-tailed tests (Fisher's, mid-P, and Overall's continuity-corrected tests and Tocher's test), chi-square tests (with and without Yates's, Upton's, and Haber's corrections), an optional equivalence test, the ratio of proportions (with its standard error and 90%, 95% and 99% confidence intervals and Jewell's low-bias estimator), the difference between proportions (with its standard error and 90%, 95% and 99% confidence intervals computed by Fleiss's procedure and by Wilson's score method, without and with a continuity correction), the odds ratio (with 90%, 95% and 99% confidence intervals – Cornfield's and exact Fisher's and mid-P intervals – and Jewell's low-bias estimator), Yule's *Q, phi*, and *lambda*. Separate results are shown for studies in which inverse sampling was used.

For stratified data, the combined analysis provides Fisher and mid-P exact and Mantel-Haenszel tests, an optional equivalence test, three estimators of the overall ratio of proportions and of the overall difference between proportions (precision-based, Mantel-Haenszel, and DerSimonian-Laird estimators, with 90%, 95%, and 99% confidence intervals), and four estimators of the overall odds ratio (conditional and unconditional maximum-likelihood estimators, a Mantel-Haenszel estimator, and a DerSimonian-Laird estimator, with 90%, 95%, and 99% Fisher's, mid-P, Mantel-Haenszel, Cornfield-Gart, and Dersimonian-Laird intervals), heterogeneity tests and measures (*H *and *I*-squared, with their 95% confidence intervals), and (for meta-analysis) estimates of the fail-safe N and two tests for a skewed funnel plot (regression asymmetry and adjusted rank correlation tests).

### Appraisal of numerical data (module D of DESCRIBE)

This module appraises a frequency distribution, and also appraises a sequence of numbers. It describes the frequency distribution in terms of its central tendency (the mean, with its standard error and 90%, 95% and 99% confidence intervals, three robust estimators of the mean, the geometric mean, and the median, with its 95% confidence interval) and dispersion (quantiles, standard deviation, variance, mean deviation from the mean, and median absolute deviation from the median), and it performs the Grubbs test for outliers. The shape of the frequency distribution is appraised in terms of symmetry or skewness (Bowley's quartiles-based skewness coefficient, Randles-Fligner-Policello-Wolfe test, Wilcoxon signed-rank test of symmetry around the sample median) and peakedness or flatness (Moors octiles-based kurtosis coefficient, Kolmogorov-Smirnov test for an even distribution). The shape of the frequency distribution is pictured in box-and-whisker diagrams, for both raw and log-transformed data. Two tests for normality (Lilliefors and D'Agostini-Pearson tests) are applied to the raw and log-transformed data. The median or mean can be compared with a hypothetical value (using a *t*-test and Wilcoxon's signed-ranks test), and the Poisson dispersion test for heterogeneity is done (appropriate only if the values that were entered are counts).

If a sequence of numbers is entered, it is tested for randomness (two runs tests, an up-and-down-runs test, and the mean square successive difference test), trend (Mann-Kendall and Cox-Stuart tests – including a test controlling for seasonal variation), a change-point, and centrifugality. The module provides Sen's estimator of slope, parametric and nonparametric linear regression analyses, and Spearman, Kendall's, and Pearson's correlation coefficients, and it smooths the curve, using procedures based on running medians and on Fourier transforms. Regression lines, smoothed curves, and the change-point are shown in a graph.

## Operating the Programs

There is no special installation procedure; the programs need only be put in a folder of the user's choice.

**Figure 1:**
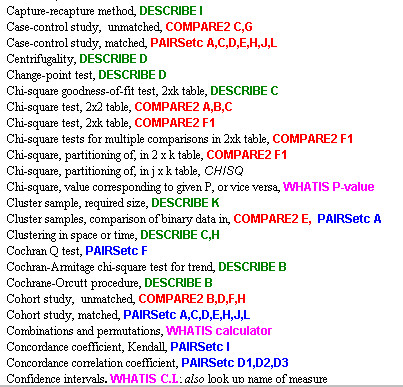
PEPI FINDER: Excerpt.

**Figure 2:**
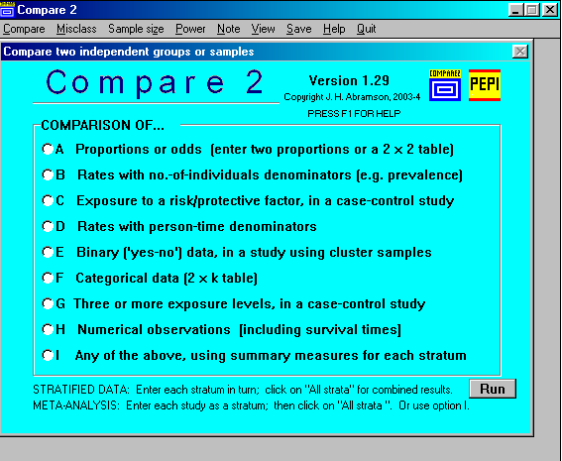
COMPARE2: Opening screen.

The appropriate program and module must first be selected. As an aid, a Pepi Finder (a Windows help file, FINDER.HLP) is provided; it is called up by clicking on its icon, and can be printed for easy reference. The Pepi Finder is an alphabetical index that shows which programs and modules deal with a specified procedure, measure, or kind of study. As seen in the excerpt shown in Figure 1, the four WINPEPI programs are colour-coded. The Finder may point to more than one module; the entry for "Case-control study, unmatched", for example, is "COMPARE2 C,G". When COMPARE2 is opened (Figure 2) it is clear that its module G is designed for a case-control study with more than two exposure categories. The index also includes procedures provided by PEPI DOS programs (shown in italics) but not by WINPEPI programs.

**Figure 3:**
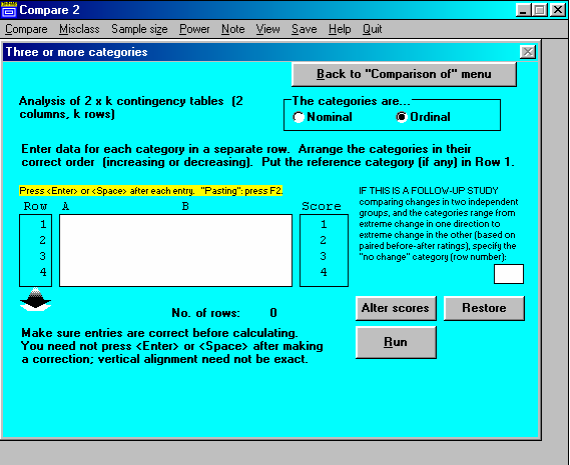
COMPARE2: Data-entry screen (for 2 × *k *table).

Each program has an opening screen (Figure 2) that displays a main menu and a top menu. Except in WHATIS, data entry is possible only after a selection has been made; a data-entry screen then appears. As an example, if option F of COMPARE2 is selected, i.e. "Categorical data (2 × k table)" (see Figure 2), the opening screen is replaced by the data-entry screen shown in Figure 3.

The programs do not read data files, but require the entry of data that have already been counted or summarized, either manually or by using statistical software that processes primary data. The data can be entered at the keyboard, or (in multiple-entry boxes for the entry of tables) can be "pasted" from a file in which they are available. Once entered, tabular data can be pasted to a text file for future re-use by pasting. Alternative forms of data are often accepted, e.g. numerators instead of rates or proportions, and either individual or grouped observations. Warning messages are shown if obvious errors are made when entering data or if essential items are omitted.

Simple on-screen instructions are provided, using simple language. For example, dichotomous variables are referred to as "yes-no" variables, and metric-scale observations, continuous or discrete, as "numerical". The term "rate" is used both for rates that have person-time denominators (e.g. incidence density) and for measures whose denominators are numbers of individuals (e.g. prevalence and risk); when the distinction is important, this is indicated. The instructions make use of terms well-known to epidemiologists, such as "case-control study", "exposed" and "not exposed", and "risk factor". (If the programs are used outside an epidemiological context, allowance must be made for their epidemiological labels.)

To simplify operation, the program generally performs and reports all the prescribed procedures that the data will permit, without requiring choices by the user. But some options may be offered. In Figure 2, for example, three options are shown: the categories may be nominal or ordinal, the scores allotted to the categories can be changed, and there is an option for performing a very specific kind of follow-up study. If "nominal" is checked instead of "ordinal", the instructions change, and the only option is for the partitioning of chi-square. Clicking on an option may modify the procedures a module performs, the manner in which the computation is done (e.g. depending on whether number-of-individuals or person-time denominators are entered, or whether a normal distribution can be assumed), and the data requirement (e.g. monthly or weekly or daily data for the appraisal of seasonal variation). Choice of an option may also modify the output. For example, the module that does a meta-analysis of studies of screening or diagnostic tests and produces forest plots for sensitivity, etc., permits optional display or suppression of the detailed numerical results for all studies.

Pop-up hints and help screens are provided.

**Figure 4:**
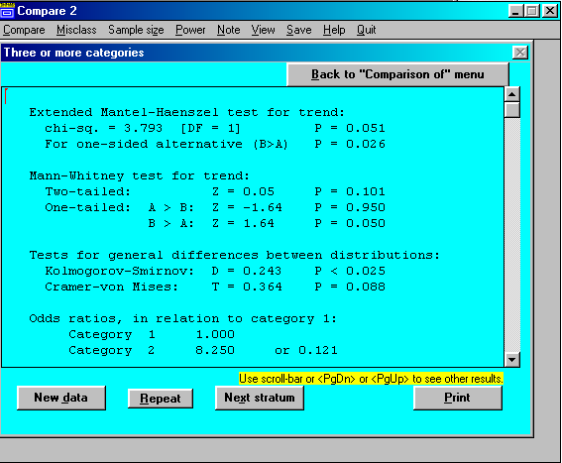
COMPARE2: Results screen (for 2 × *k *table).

Results are shown in an output screen (Figure 4), from which it is easy to return to the main menu or the previous screen. Results automatically go to the Windows clipboard, from which they can be pasted to other files. Clicking on "View" in the top menu displays all results obtained in the current session. "Print" options are offered. By clicking on "Note" in the top menu, it is possible to add comments to the results, for pasting, printing, or saving. A "Repeat" button is provided, permitting repeated analyses of the same data with changed options.

All results are saved in a disk file, unless the user changes this default. The WINPEPI package contains a utility program (JOINTEXT) that can merge result files.

**Figure 5:**
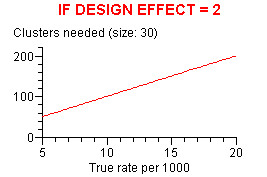
Number of clusters required for a cluster-based prevalence study.

**Figure 6:**
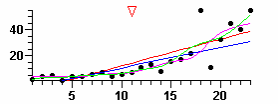
A series of numerical observations. The straight lines are simple linear and nonparametric regression lines; the curved lines represent smoothing by two different methods; the red triangle marks the first point at which there is a significant change.

**Figure 7:**
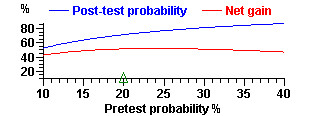
Post-test probabilities and net gain for a diagnostic test. Positive likelihood ratio = 10. The net gain is the absolute difference between pretest and post-test probabilities.

**Figure 8:**
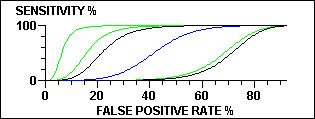
Comparison of ROC curves.

DESCRIBE (but no other WINPEPI program) displays graphs – box-and-whisker plots, survival curves, seasonal peaks, regression lines, smoothed curves, forest plots, scattergrams, summary ROC curves, and graphs showing required sample sizes under different conditions. In most of the graphs, numerical values can be read by mouse-clicking at any location, optionally after magnifying a segment (zooming). Specimen graphs are shown in Figures 5 to 8 Figure 5 shows the number of clusters required for a cluster-based prevalence study (with stipulated requirements) for a true prevalence ranging from 5 to 20 per 100; the number can be read by clicking on the graph. Figure 6 shows a series of numerical observations, with regression lines, smoothed curves, and the change-point. Figure 7 shows post-test probabilities and net gain for a diagnostic test with a given likelihood ratio, for a range of pretest probabilities. Figure 8 shows a comparison of ROC curves, for use in appraising the effect of a covariate on the accuracy of a diagnostic test.

## Documentation

Comprehensive manuals are provided. These furnish full information about each module, including explanations of the uses, limitations, and applicability of specific procedures, and formulae or references. The Pepi Finder serves as an index to the manuals.

## Discussion

Criteria for the appraisal of statistical software for epidemiology [7] include not only its capabilities, but also "smoothness of the installation, simplicity of the interface, ease of use, completeness and statistical quality of the documentation, completeness and appearance of statistical graphics, accuracy of statistical computations".

The WINPEPI programs are easy to install and easy to use (with the reservations discussed below). Their documentation is very detailed and (at the price of repetitiveness) includes a separate self-contained description of each module. A regrettable shortcoming of WINPEPI is that only one of the programs, DESCRIBE, presents graphic results. This is because DESCRIBE is the only 32-bit program, and the graph unit used by WINPEPI [8] is appropriate only for 32-bit programs. As for accuracy, the programs have been tested extensively, and all errors found have been promptly corrected; but (to cite the PEPI manual), it unfortunately remains a truism that no computer software can be entirely problem-free.

But the WINPEPI programs do not provide data management facilities, and some other software package must be used if the data require processing. An epidemiologist or student whose data have been stored and maybe processed in another package, and who is well versed in the use of that package, may therefore have no need for the WINPEPI programs, despite their ease of operation, except when these do analyses not done by the other package. The WINPEPI programs aim "to complement – not replace – other statistics packages" [5].

Also (unlike the DOS-based PEPI programs for multiple logistic and Poisson regression analyses), the WINPEPI programs do not read data files. Data must be entered each time a program is used. This drawback is partly overcome by the possibility of pasting tabular data into data-entry boxes. But data entry can be tiresome, and users accustomed to programs that use data files may find it particularly vexatious. On the other hand, for some purposes keyboard entry may be seen as a boon: "Although conventional statistical software packages are adequate when you have a data set to work with, they are not always helpful when you need to do keyboard entry of data and rapidly perform simple analyses. For instance, you may want to replicate some analyses from a journal article and compute a Mantel-Haenszel odds ratio, or you may want to compute the sample size for your study while writing a grant proposal. Maybe you want to demonstrate to your students the impact of increasing sample size on the confidence intervals of a proportion. Perhaps you are a student and would like to do your epidemiology or biostatistics homework with some easy-to-use analytical routines... It is in this niche area that PEPI scores!" [6].

A criticism of version 3 of PEPI as being insufficiently user-friendly [9] led to a major revision in version 4. In the WINPEPI programs, user-friendliness is maximized by the provision of the Pepi Finder, simple on-screen instructions, pop-up hints and help screens, and warning messages, by streamlined data-entry procedures, which accept alternative forms of data, by the automatic saving of results, by the ease with which results can be recalled, annotated, printed, and pasted, and sometimes by the provision (in the output screens) of comments on the applicability of specific results.

Unfortunately the wide variety of statistical procedures that is offered makes the WINPEPI programs less convenient to use; versatility carries a price. Even the provision made for the entry of alternative forms of data, meant as a convenience, necessitates a decision and may hence be an inconvenience – for example, a simple comparison of two proportions (using module A of COMPARE2) requires a choice between entry of four frequencies, of numerators and denominators, or of proportions and denominators.

The DOS-based PEPI package elicited the comments "there are so many modules that sometimes it is difficult to remember which one to use" [10] and, with less restraint, "it is comprised of a large number of separate modules, which can make it a pain to use" [11]. The Pepi Finder was introduced (in version 3 of the package) to mitigate this problem. The advent of the WINPEPI programs, with their added statistical procedures, increased the potential for confusion and hence the value of the Finder, both for finding what program and module to use, and as an index to the detailed descriptions supplied in the manuals.

The possibility of confusion is of course much reduced by the fact that related modules – for example, those concerning comparisons of two independent samples – are concentrated in the same WINPEPI program. Having opened the appropriate program, the user need only click on the kind of analysis that is required. But even that may tax some users. In COMPARE2, for example, a choice between modules B and D (see Figure 2) requires an awareness of whether the denominators are number-of-individuals or person-time ones.

A further penalty for WINPEPI's versatility is that users may be confused by the large number of results in the output, some of them of little or no obvious relevance. As described above, module A of COMPARE2 (for a 2 × 2 table), for example, provides numerous "exact" and chi-square tests, and three measures of association, with confidence limits computed by different methods, as well as other results, including some that are valid only if inverse sampling was used. Similarly, module D1 of PAIRSetc (for paired numerical observations) provides three tests, six intraclass coefficients and a number of other measures of agreement, appropriate for different purposes. For this reason, every WINPEPI manual carries the admonition: "This program offers more options than most users will ever need, and will usually display more results than are needed. Ignore the options and results you don't require". (This of course assumes that the user knows what he or she wants.)

But while all the results cannot be of interest to an ordinary user, each of them may be of interest to some users. As pointed out in a review of epidemiological software [11], "what one person might call 'statistical clutter' might be desirable to other people or even to that person if the person learned about that statistic". A review of PEPI says "Will you need all the programs in PEPI? Probably not. We have, for example, never used the Jonckheere-Tepstra test for trend or the Kullback-Leibler distances. However, more is good..." [6]. If a user wishes only to compute *kappa*, it can do no harm if the output provides extra results that draw attention to the fact that *kappa *has a ceiling value, or that its value can be adjusted to avoid paradoxical results. The user may be stimulated to use some of the additional procedures, after (if necessary) learning more about them. The manuals carry the warning: "It is unwise to use a statistical procedure whose use one does not understand. This manual cannot supply this knowledge, and it is certainly no substitute for the basic understanding of statistics and epidemiological thinking that is essential for the wise choice of methods and the correct interpretation of their results".

The provision of alternative tests, and estimators based on alternative methods, may of course be confusing, whatever explanatory comments may be offered in the output or the manuals. But it may permit a knowledgeable user to select the method most appropriate in a particular situation, and it serves as a reminder to the less knowledgeable user that different methods exist, based on different assumptions and using different models, most of them yielding approximations, and none of them having absolute validity for all purposes, and as a warning that caution is indicated if different methods lead to very different conclusions. "Exact" results computed in different ways differ, and "exact" probabilities and confidence intervals are not always preferable to probabilities and confidence intervals computed in other ways.

The length of the list in the Pepi Finder testifies to the wide variety of statistical routines offered. "The programs cover an amazing array of applications", says one review [6]. PEPI has repeatedly been called a "Swiss army knife" of utilities for epidemiologists and biomedical researchers [6,12,13]. One reviewer added, "one will find here more analytic options for a simple 2 × 2 or 2 × K table than will probably be needed during an entire epidemiology career" [13]. Another compared several packages when estimating sample size for a matched case-control study, and "found that PEPI provides an output richer than others do. This feature is common to other programs in PEPI" [14].

PEPI is of course very far from being a complete compendium of statistical routines for epidemiologists. It does not, for example, provide Cox regression, log-linear analysis, multiple regression analysis, procedures for the study of disease clustering, and many other procedures of interest to epidemiologists [7,11], which must be sought elsewhere. But it is a handy resource for many routines that are not very commonly used or very easily found, such as those concerned with misclassification, meta-analysis, reliability studies, the appraisal of screening and diagnostic tests, the equivalence of two proportions or means, cluster samples, inverse sampling, capture-recapture studies, serial correlation of residuals, skewed funnel plots, direct standardization using age intervals as weights, smoothing of curves, generalized odds ratios for ordinal data, quantitative measures of heterogeneity, harmonic analysis in the study of seasonality, and bias-adjusted and prevalence-adjusted bias-adjusted estimates of *kappa *(a feature picked out as "unique" in one comparison of PEPI with other epidemiological software [11]).

From the viewpoint of veterinary epidemiologists, a shortcoming of WINPEPI is its use of "person-time" and not "animal-time". But they are doubtless used to this.

WINPEPI's potential as a learning and teaching aid is worth stressing. Students welcome the facts that the package is free and requires no special installation procedure, and that (unlike major general-purpose statistical packages) it uses epidemiological language and provides results that are meaningful to epidemiologists. They rapidly learn to use the Pepi Finder and the programs themselves. They find the programs easy to use, although they may at first be confused by the multiplicity of modules and results; but they rapidly learn to focus on the specific modules and results that interest them, and to disregard others. At the same time, the rich output may serve to acquaint the student with other measures and tests, and excite interest in them. The weight the programs give to measures of association and their confidence intervals may help to counteract the belief that significance testing is the be-all and end-all of an analysis.

"PEPI facilitates a ready understanding of important epidemiologic concepts, unfettered by the complexities of statistical programming", says a reviewer [6]. With appropriate data, for example, the Mantel-Haenszel results provided by COMPARE2's module B can serve as an object lesson on the assessment of confounding and effect modification, the control of confounding, and appraisal of the defensibility of a summary odds or risk ratio. The student can concentrate on analysis and interpretation, with no need to get involved in data management, sorting and tabulation.

A useful feature is that, by clicking on the "Repeat" button and making changes to the data or options, students can easily do "what if?" exercises [15]. For example, they can easily learn, by manipulating data, how differences in prevalence or the number of controls per case can alter the required sample size, or how consideration of cost can alter sample size decisions in stratified sampling, or how the sensitivity or specificity of measures can alter a prevalence estimate or an odds or risk ratio. The sensitivity analysis provided by a module in COMPARE2 can demonstrate how markedly a single aberrant result can affect the results of a meta-analysis. Using the "misclassification" modules, it may be a salutary experience for students – and possibly also for some more experienced epidemiologists – to learn that an observed prevalence of 120 per 1000, using a measure whose sensitivity and specificity are 90%, points to a true prevalence of only 25 per 1000, or to find how inaccurate their guesses about the effect of misclassification on an odds or risk ratio can be.

A recent epidemiology textbook makes frequent use of PEPI in its exercises "to relieve students from some of the tedium and anxiety of hand calculation, while opening up possibilities of using advanced techniques that might not otherwise be available. It is time to familiarize even introductory students to these essential tools of the trade" [16].

## Conclusions

WINPEPI complements other statistics packages. It is versatile, providing a wide variety of statistical routines commonly used by epidemiologists, but is far from being a complete compendium of such routines. It is a handy source of many procedures that are not very commonly or easily found.

The programs are in general user-friendly, although some users may be confused by the large numbers of options and results provided. The main limitation is the inability to read data files, but tabular data can be entered by pasting, and for some purposes keyboard entry of data is an advantage. Only one of the programs presents graphic results.

WINPEPI has a considerable potential as a learning and teaching aid.

## Availability and requirements

The current version (at the time of this writing) of the software is available for free download as an additional file (WINPEPI.ZIP) attached to this article. It includes the programs, their manuals, and the Pepi Finder. Subsequent versions will be available at http://www.brixtonhealth.com for free download. Information about the latest WINPEPI version can be found at http://www.sagebrushpress.com/pepibook.html, where the DOS-based programs are available for free download.

The programs and manuals are copyrighted, but may be freely copied and distributed for personal use; they may not be exploited commercially without permission.

COMPARE2, PAIRSetc, and WHATIS are 16-bit programs (written in Delphi version 1) that can be run in any version of Windows. DESCRIBE is a 32-bit program (written in Delphi version 5), and can be run in any version of Windows except Windows 3.

The manuals for DESCRIBE, COMPARE2, and PAIRSetc are in PDF format, and can be read or printed with Adobe Acrobat. WHATIS is documented in the version 4 manual [5].

## Competing interests

The author wrote the WINPEPI programs and manuals and is co-author of the DOS-based programs and manual, and hence may be biased in their favour.

## Supplementary Material

Additional File 1 WINPEPI package. WINPEPI programs, with manuals and Pepi Finder. Click here for file
