# Supplementary material for: WINPEPI (PEPI-for-Windows): computer programs for epidemiologists
Source: Epidemiol Perspect Innov. 2004 Dec 17;1:6. doi: 10.1186/1742-5573-1-6 (PMC544871; doi:10.1186/1742-5573-1-6)
Supplement: Additional File 1 — WINPEPI package. WINPEPI programs, with manuals and Pepi Finder. [file 1742-5573-1-6-S1.zip › Compare2.pdf]

COMPARE2

MANUAL

(Version 1.31)

© J.H. Abramson

Revised Oct. 21, 2004

What COMPARE2 does

COMPARE2 is a WINPEPI (PEPI-for-Windows) program, part of the PEPI suite of computer programs for epidemiologists. ("PEPI" is an acronym for "Programs for EPIdemiologists".) It can be run in any version of Windows.

COMPARE2 provides procedures for use in comparisons of two independent groups or samples. It may be used for analyses and meta-analyses of cross-sectional, cohort and case-control studies, and of trials. It can handle both categorical data (dichotomous, nominal or ordinal; including clustered binomial data) and numerical data (including survival times). It can analyse stratified data and can compute power and sample sizes. It has 28 modules:

How to use COMPARE2 3

Comparisons:

A. Comparison of proportions or odds (analysis of 2 x 2 tables) 5

B. Comparison of rates that have number-of-individuals denominators 16

C. Comparison of exposure to a risk/protective factor (in a case-control study) 26

D. Comparison of rates that have person-time denominators 34

E. Comparison of binary ("yes-no") data, in a study using cluster samples 42

F. Comparison of categorical data (analysis of 2 x *k* tables)

- **F1. Comparison of categorical data (3 or more nominal categories)**...............................44
- **F2. Comparison of categorical data (3 or more ordered categories)**...............................49

G. Comparison of three or more exposure levels (in a case-control study) 54

H. Comparison of numerical observations (including survival times)

- **H1. Comparison of numerical observations : normal distribution not assumed** 59
- **H2. Comparison of numerical observations : normal distribution assumed** 64
- **H3. Comparison of survival times** 68

I. Any comparison, using summary measures for each stratum or study 74

Misclassification

M. Effect of misclassification 80

Power

P1. Power of test for comparison of proportions 81
P2. Power of test for comparison of proportions: stratified data 82
P3. Power of test for comparison of distributions in ordered categories 83
P4. Power of test for comparison of means 84

Sample sizes

S1. Sample sizes for comparison of proportions 85
S2. Sample sizes for testing equivalence of proportions 88
S3. Sample sizes for comparison of proportions (stratified data) 89
S4. Sample sizes for comparison of proportions (multiple logistic regression) 90
S5. Sample sizes for comparison of distributions in ordered categories 92
S6. Sample sizes for comparison of means 93
S7. Sample sizes for testing equivalence of means 94
S8. Sample sizes for comparison of means (multiple linear regression) 95
S9. Sample sizes for comparison of numbers of events (e.g. disease onsets/spells) 96
S10. Sample sizes for comparison of survival (time to event) 97
S11. Sample sizes for study of change (using before-after ordinal-scale ratings) 99

References 101

WORDS OF CAUTION

This program offers more options than most users will ever need, and will usually display more results than are needed. Ignore the options and results you don't require.

It is unwise to use a statistical procedure whose use one does not understand. This manual cannot supply this knowledge, and it is certainly no substitute for the basic understanding of statistics and epidemiological thinking that is essential for the wise choice of methods and the correct interpretation of their results.

How to use COMPARE2

First **choose a module.** Then follow the simple on-screen instructions. To return to the "*Comparison of*" menu, click on the "*Back to Comparison of.*" button, or on "*Compare*" in the top menu.

If the data are **stratified**, enter each stratum in turn, and click on "*All strata"* whenever combined results are required. For **meta-analyses**, enter each study as a separate stratum, and click on "*All strata*" whenever combined results are required; or use module **I**.

Choosing a module:

If you wish to compare two groups or samples, select a module between **A** and **I** in the "*Comparison of*" menu. The choice depends mainly on the dependent variable– what is measured in the two groups?

- If the dependent variable is a *dichotomy* (a "Yes-No" variable), select a module between **A** and **E**. Modules **A, B, and C** are appropriate if the denominators are numbers of individuals, but are not appropriate for person-time data. Module **A** can be used for any 2 x 2 table, **B** is preferable in studies that focus on risks (e.g. cases per 1,000) rather than proportions, and **C** for comparisons of cases and controls. Module **D** is designed for studies using person-time data, and **E** for studies based on cluster samples**.**
- If the dependent variable has *three or more categories*, click on **F** or (for a case-control study) **G.** After clicking on **F**, you will be asked to choose **F1** (nominal categories) or **F2** (ordinal categories).
- If the dependent variable is *numerica*l, click on **H.** You will be asked to choose between **H1** (assuming a normal distribution), **H2** (not assuming a normal distribution), and **H3** (for comparing survival times).
- Module I is designed for meta-analyses in which summary data are already available for each study, so that it is not necessary to enter a series of tables. It can also compute a weighted mean of a set of simple proportions.

To appraise the possible effect of *misclassification* on a 2 x 2 table, click on "*Misclass"* in the top menu (module **M**).

To estimate the *power of a test,* click on "*Power'* in the menu at the top, then select a module between **P1** and **P5**. .

To estimate a required *sample size,* click on" *Sample size"* in the top menu, then select a module between **S1** and **S11.**.

Easy entry of data:

- If entries are required in different boxes, pressing *Enter* or *Tab* after entering a number will generally take you to the next box; pressing *Escape* will clear the entry.
- If several entries are required in the same box, press *Enter* or *Space* after each entry.
- Optionally, data can be "pasted" into entry boxes (see next page).

Recalling results:

Click on *"View"* in the top menu to display the current session's previous results

Pasting results:

Results shown on the screen are automatically placed in the Windows clipboard**,** from which they can be pasted to other applications, at the site of the cursor (usually by pressing *Shift-Insert* or *Ctrl-V*). Click on "*Not*e" in the top menu if you wish to add explanatory comments to be placed in the clipboard (or printed)) with the results

If the current session's previous results are recalled (by *clicking on* "View"*)*, text can be marked (drag the mouse over it with button pressed) and copied to the clipboard (by pressing *Ctrl-Insert* or *Ctrl-C)* for pasting elsewhere.

Saving results:

By default, all results of Pepi-for-Windows programs are saved in C:\PEPI.TXT, with a warning if it exceeds 500K. Results also go to C:\PEPI.TMP (for display in the 'View' option); this file may be overwritten unless it is renamed on quitting COMPARE2. Click on “Save” (in the top menu) to see the default procedure or to change it. TXT files can be combined with JOINTEXT, available free from www.brixtonhealth.com.

Printing results:

Click on *“Print”.* If this fails, try switching the printer off and on again. Or paste the results from the clipboard to Word or another program, and print from there. Results can also be printed from the file in which they are saved. Note: the “Print” option ejects full pages only.

The ”*Run fast*” option (if offered) omits exact computations; this may be useful for slow computers or large numbers.
If you get an “*Error opening window*” message, close and re-open COMPARE2.

PASTING DATA

If the data are available in a text file (e.g. a TXT file created by Notepad), they can be copied to the Windows clipboard copied from a data-entry box and pasted to a text file for future re-use; press *Ctrl-A* to mark it for copying.]

Precautions:

- The data must be pasted into the box as a single block, and not piecemeal.
- The data must be in the format required in the box, with spaces between the numbers; exact alignment of the
  columns is not necessary. For example
  ```
  45 66  1
  20  3 132
  53  11  44
  ```
- If a defined number of rows is required, this number must be entered first, e.g. in the “Number of strata” or “Number of categories” box.
- If row numbers are shown on the left (1, 2, etc.), ensure that the”1” is visible before pasting.
- The cursor must be in the top left corner of the box when the “paste” keys are pressed.

HOW TO OBTAIN PEPI PROGRAMS

All WINPEPI (PEPI-for-Windows) and other PEPI programs can be downloaded free. The latest versions of WINPEPI programs – currently COMPARE2, DESCRIBE, PAIRSetc, and WHATIS – can be downloaded from www.brixtonhealth.com; and the latest release of Version 4 of PEPI, which contains over 40 DOS-based programs (which can be used in Windows) and WHATIS, can be downloaded from www.sagebrushpress.com/pepibook.html or www.simtel.net/pub/pd/54632.html

COMPARE2, DESCRIBE, and PAIRSetc are distributed with manuals (as computer files). A printed manual is available for the DOS-based programs and WHATIS (Abramson and Gahlinger 2001.).

WINPEPI programs are provided with no liability to users and without any warranties, whether expressed or implied. They are copyrighted, but may be freely copied and distributed for personal use; they may not be exploited commercially without permission.

A. COMPARISON OF PROPORTIONS OR ODDS (analysis of 2 x 2 tables)

This module can be used to analyse any simple 2 x 2 contingency table that shows the findings in two independent groups, or a series of such tables representing the findings in different strata or in different studies. The data may be derived from an observational study (cross-sectional or cohort) or from a trial. The program can handle data collected by *inverse sampling* (see below).

This module is not appropriate for person-time data (for which module D of this program should be chosen). Module B is to be preferred in studies that focus on rates (e.g. cases per 1,000) rather than proportions, or if measures of impact (e.g. attributable fractions) are required, or the numbers that are needed to avoid or produce one case. Module C is preferable for comparisons of cases and controls with respect to their exposure to a risk or protective factor.

Data may be entered in a 2 x 2 table format, or as numerators (case frequencies) and denominators, or as proportions and denominators. In a table showing the relationship between two variables, there is an option for entering the number of missing values (for one variable).

For *stratified data*, enter the table for each stratum in turn, and then click on "*All strata*" to obtain the combined results, which permit appraisal of the association while controlling for confounding effects of the stratifying variable or variables, and assessment of heterogeneity.

For a *meta-analysis*, enter the table for each study in turn (as a separate stratum), and then click on "*All strata*" to compare and combine the results in separate studies.

To compare the changes observed in two groups in a *before-after study* with independent *"before"* and *"after"* observations, enter the before-after table for each group in turn, and then click on "*All strata*" for heterogeneity tests.

For *each table* entered, the program provides **exact probabilities** (Fisher's, mid-P and continuity-corrected), **chi-square tests of association**, an optional **equivalence test**, and several measures of association, the **ratio of proportions** (with its confidence intervals), the **difference between proportions** (with confidence intervals computed by a large-sample method and Wilson's score method), the **odds ratio** (with Fisher's exact, mid-P exact, and Cornfield's confidence intervals); **Yule's *Q*, phi and lambda**. If the numbers of **missing values** for one variable are entered the difference between proportions (with respect to the other variable) is estimated by imputation.

For a *series of tables*, the program computes **exact probabilities** (Fisher's and mid-P), a **Mantel-Haenszel** test, an optional **equivalence test, heterogeneity tests and measures,** estimators of the **overall ratio of proportions, difference between proportions, and odds ratio** (with their confidence intervals), and (for use in *meta-analysis)* estimates of the **fail-safe N** and two **tests for a skewed funnel plot** (suggesting publication bias) .

For studies in which **inverse sampling** was used, the program provides a large-sample significance test and estimates the difference between proportions, the ratio of proportions, and the odds ratio.

Exact probabilities

For each table, the program computes P-values based on exact tests. A number of results are provided, since there is no consensus as to the best method of computation (Armitage *et al.* 2002: 136-137). If these results lead to conflicting conclusions, inferences may require careful consideration.

One-tailed P-values are shown for each tail – first assuming that the direction of the findings is consistent with the study hypothesis, and then assuming that the findings point in the opposite direction. *Fisher's P,* the one-tailed value usually used, is the conditional probability of the observed number of individuals, or of any more extreme number, in the relevant direction (i.e. of a larger number if the observed number exceeds chance expectation, or of a smaller number if the observed number is less than might be expected by chance). Fisher's P has a conservative bias, which may be important when sample sizes are small. The other one-tailed values shown reduce this bias – *mid-P estimates* (half the probability of the observed number, plus the probability of a more extreme number) and *continuity-corrected estimates* (Overall 1990). A mid-P value "does not guarantee that the Type I error rate falls below a fixed value. However, it usually performs well and is less conservative than Fisher's exact test" (Agresti 1996: 43). Armitage *et al.* (2002: 120) recommend that both the Fisher P and the mid-P value should be given but that with more emphasis on the latter.

If the Fisher exact test "doesn't quite make it" – that is, if Fisher's one-tailed P-value (based on the probability of the observed number or a more extreme number) lies above a critical level (0.05, 0.01, 0.005, or 0.001), but the P-value based solely on more extreme findings falls below this significance level –the program applies *Tocher's test*, which is a more powerful modification of the Fisher exact test. The test uses a random number to decide whether probabilities that are exactly equal to that of the observed constellation should be taken into account;. If the test is repeated, it will use a different random number and may give a different result.

Six *two-tailed P-values* based on exact tests are displayed.. *Fisher's two-tailed P* is the sum of the probabilities of all sets of possible findings (whether the number of exposed cases is smaller or larger than the observed number) that have specific probabilities that do not exceed the probability of the observed constellation. This estimate has a conservative bias, and is very sensitive to small perturbations in the table (Dupont 1986). *Double the one-tailed Fisher's P* for the observed direction (Dupont 1986, Mantel 1990), an estimate recommended by some statisticians, is a conservative one that is usually close to the value provided by Yates's correction; a significant result is strong evidence for a difference in the observed direction (Armitage *et al.* 2002: 136). The *mid-P two-tailed value* is the sum of probabilities (of specific configurations) that are smaller than the probability of the observed configuration, plus half the sum of probabilities that coincide with the probability of the observed configuration. *Double the one-tailed mid-P value* is suggested by Rothman 1986:162. The program also computes the *continuity-corrected two-tailed P* and *double the continuity-corrected one-tailed P.*

For a *series of tables* ("all strata"), the program computes one-tailed *Fisher* and *mid-P* probabilities, and doubles them to obtain two-sided P values. Many statisticians prefer the mid-P value when results from several studies are combined (Armitage *et al.* 2002: 137)

Chi-square tests of association

For each table, a simple chi-square test of association is performed, supplemented by tests with Yates's, Upton's, and Haber's corrections to overcome the slight anticonservative bias of the

uncorrected value. Yates's correction is criticized by many statisticians on the grounds that a test that uses it is too conservative, with an increased risk of a type II error (failure to reject the null hypothesis when, in fact, it is false). The other (smaller) modifications may be preferred; Zar (1998: 494) calls Haber's correction (as modified by Ghent) "excellent". If the tests lead to conflicting conclusions, inferences may require careful consideration.

Chi-square tests may be misleading if the expected frequencies (under the null hypothesis) are too small. The program displays a warning if there are cells with expected frequencies of less than 5.

Mantel-Haenszel test

This overall test of association controls for confounding effects of the stratifying variable or variables – e.g. of age and sex if the strata represent different age-sex groups. In a meta-analysis, it is an overall test, controlling for the differences between the studies.

The test is performed with and without a continuity correction. It is usually applied without a continuity correction.

The Mantel-Haenszel test is valid even for sparse data, provided that overall numbers are sufficient. A message is displayed if the overall numbers are too small to warrant use of the test.

Heterogeneity tests and measures

For stratified data (i.e., a series of tables), the program provides *heterogeneity tests* for the ratios of proportions, the differences between proportions, and the odds ratios in the different strata. These tests should be interpreted with caution, since their power is low; if the result is significant at the 0.05 level, the hypothesis of homogeneity can be rejected; but "a high p-value ... does not show that the measure is uniform, it only means that heterogeneity ... was not detected by the test" (Rothman and Greenland 1998: 276); the larger the strata, the more valid the test.

The program also provides two *measures of heterogeneity*, *H* and *I-squared*, with their approximate 95% intervals, for the ratios of proportions, the differences between proportions, and the odds ratios An *H* value of less than 1.2 suggests absence of noteworthy heterogeneity, whereas a value exceeding 1.5 suggests its presence, even if the heterogeneity test is not significant. *I-squared* expresses the proportion of variation that can be attributed to heterogeneity (in a meta-analysis, to interstudy variation) rather than to sampling error.

Estimates of the supposed common underlying value of the ratio of proportions, the difference between proportions, and the odds ratio (see below) are of questionable value if the findings in the various strata are very disparate. If the results are not uniform, explorations of possible causes – e.g. associations with study design or quality or with the sizes or other characteristics of the samples – may be revealing

The uniformity or heterogeneity of the measures in the different strata can be appraised not only by these tests and measures, but by plotting the values and their confidence intervals graphically, and comparing them.

Equivalence tests

Equivalence tests are offered, for use in appraising the similarity of two proportions. These tests may be indicated if no statistically significant difference between the proportions has been found, e.g. in "negative trials" comparing a new treatment with an established standard treatment, where there may be a reason to prefer the new treatment if it is at least as effective as the standard treatment.

If equivalence tests are requested (by using the "*Check here for equivalence tests*" instruction) they will be done for the table entered, for subsequent strata, and for the combined strata.

The bounds of "equivalence" mist be defined, by specifying either the largest difference between proportions (e.g. 0.05) that is to be regarded as negligible, or the largest ratio of proportions (e.g. 1.1) that is to be considered negligible.

Two tests are done, the null hypothesis in each being that there is more than the specified "negligible" difference. In one test, the null hypothesis is that the first proportion is more than negligibly higher than the first. In the other test, the null hypothesis is that the second proportion is more than negligibly higher than the first. In each test, a significant result supports the alternative hypothesis that the one-sided difference is negligible.

The proportions can be regarded as equivalent if both tests yield significant results,(supporting both the alternative hypotheses). If only one test is significant, this indicates that one proportion is at least as high as the other. Non-significant results may be attributable to small sample size.

Ratio of proportions

For each table, the program displays the ratio of the proportion in group A to the proportion in group B, with its standard error and 90%, 95% and 99% confidence intervals. This ratio is likely to be of interest in epidemiological studies of causal associations.

It also displays *Jewell's low-bias estimate* of the ratio of proportions in the population, which draws attention to the tendency for the ratio in a sample, especially a small one, to misrepresent the true ratio in the population represented. A disadvantage is that this estimator is affected by the direction of computation: the reciprocal of the A:B estimator is not the same as the B:A estimator.

Difference between proportions

For each table, the program displays the difference between the proportions (the proportion in group A minus the proportion in group B), with its standard error and 90%, 95% and 99% confidence intervals computed by three different methods.

This difference is likely to be of interest in studies of the magnitude and economic aspects of public health problems. The confidence intervals are estimated by Fleiss's procedure (appropriate for large samples only), and by Wilson's score method, without and with a continuity correction. The latter methods are said to have advantages over the Fleiss intervals (Newcombe 1998).

Odds ratio

For each table, the program displays the odds ratio (the ratio of the odds in group A to the odds in group B, with its reciprocal), its 90%, 95% and 99% confidence intervals (Cornfield's intervals, and exact Fisher's and mid-P confidence intervals), and an adjusted estimate of the odds ratio (after adding 0.5 to each cell frequency).

It also displays *Jewell's low-bias estimate* of the odds ratio in the population, which draws attention to the tendency for the odds ratio in a sample, especially a small one, to misrepresent the true odds ratio in the population represented. A disadvantage is that (unlike the ordinary odds ratio) this estimator is affected by the direction of computation: the reciprocal of the A:B estimator is not the same as the B:A estimator (Walter and Cook 1991, Mantel 1992).

The odds ratio is likely to be of interest in epidemiological studies of causal associations.

Overall ratio of proportions, difference between proportions, and odds ratio

For a *series of table*s ("all strata"), the program computes three estimators of the *overall ratio of proportions* and of the *overall difference between proportions* (precision-based, Mantel-Haenszel, and DerSimonian-Laird estimators, with 90%, 95%, and 99% confidence intervals), and four estimators of the *overall odds ratio* (conditional and unconditional maximum-likelihood estimators, a Mantel-Haenszel estimator, and a DerSimonian-Laird estimator, with 90%. 95%, and 99% exact Fisher's, exact mid-P, Mantel-Haenszel, Cornfield-Gart, and Dersimonian-Laird intervals).

The ratio of proportions and odds ratio are likely to be of interest in epidemiological studies of causal associations, and the difference between proportions is likely to be of interest in studies of the magnitude and economic aspects of public health problems.

These estimates of the supposed common underlying values, and their confidence intervals, should be treated with caution if the findings in the various strata are very disparate. The heterogeneity tests have a low power, and are unreliable with small frequencies (Rothman 1986: 223).

The estimators other than the DerSimonian-Laird estimator are based on a *fixed-effect model*, which assumes that the strata provide estimates of the same true effect. The *precision-based* estimators are weighted means, using the reciprocal of the variance in each stratum as the weight for the stratum when the data are pooled, and are appropriate if the sample in each stratum is large; strata that have a zero proportion do not enter into the calculation, and an appropriate message is shown. The *Mantel-Haenszel* estimators are applicable even when there are sparse data in each stratum.

The *DerSimonian-Laird* estimator is based on a *random-effects* model, which assumes that the strata provide estimates of randomly differing effects. The variation between strata is taken into account, resulting in wider confidence intervals and a more conservative significance test. The random-effects model gives more weight to small studies, and may be inappropriate if sample sizes are very small. Some investigators use it when unexplained heterogeneity is present (Fleiss and Gross 1991, Petitti 1994, Whitehead and Whitehead 1991). "In essence," say Rothman and Greenland (1198: 668), "a random-effects model exchanges a doubtful homogeneity assumption for a fictitious random distribution of effects . The advantage ... is that the standard errors and confidence limits ... will more accurately reflect uncertainty about unaccounted-for sources of variation". The Dersimonian-Laird estimator is not displayed if this procedure does not increase the variance. A chi-square test appraises whether the estimator differs from zero.

Yule's Q, phi and lambda

Yule's *Q*, *ph*i and *lambda* (measures of association) are displayed for each table.

Yule's Q (Zar 1998:404) ranges from 0 (no association) to 1 (complete positive dependence) or -1 (complete negative dependence). In a 2 x 2 table it is equivalent to the *gamma* coefficient.

Phi (Zar (1998: 403) ranges from -1 (complete inverse dependence) to +1 (complete positive dependence); 0 indicates no association. It is not displayed if the expected frequency in one or more cells is <5. In a 2 x 2 table *phi* is equivalent to Cramer's coefficient *V.*

Goodman and Kruskal's *lambda (*Siegel and Castellan (1988: 298-303) is a coefficient of forecasting efficiency that expresses the capacity of one variable to "predict" the other. It is an assessment of the proportion of incorrect predictions concerning one variable that would be prevented if information about the other variable was available. Two *lambda* statistics are computed; one refers to the prediction of the variable whose categories are “Yes” or “No” when it is known to which group (A or B) the individual belongs, and the other refers to a prediction in the reverse direction. *Lambda* ranges from 0 (if the one variable is of no help in predicting the other) to 1 (if the one variable perfectly specifies the categories of the other).

Unlike Yule's *Q* (and the odds ratio), *phi* and *lambda* vary with the relative sizes of the two groups that are compared, and should in general be used only if these groups together make up a defined population, or comprise a representative sample of a defined population. The values of *ph*i and *lambda* are then applicable to this specific population.

Fail-safe N

Estimates of the fail-safe N are provided for use in *meta-analyses*, as rough guides to the possible importance of the "file drawer problem", i.e. the exclusion of studies that were not published or were not found for other reasons.

The program computes the numbers of new "null" studies (those with an odds ratio or risk ratio of 1) that will suffice to bring the overall odds or risk ratio to a negligible level (0.8, 0.9, 1.1 or 1.2). No account is taken of P-values or the size of the null studies.

Fail-safe N estimates based on the P-values in the various studies are provided by module I of this program.

Tests for a skewed funnel plot

Two tests for a skewed funnel plot are performed, for use in meta-analysis. The tests, which examine the association between the sizes of the effects in the component studies and their precision, are the *regression asymmetry test* and the *adjusted rank correlation test.*

In each test, a low P value suggests possible *publication bias*, although the "small-study effect" (Sterne *et al.* 2000) that it indicates may have some other cause, such as the use of higher-risk subjects in smaller studies, resulting in an association between sample size and the effect under consideration.

Both tests have a low power if they are based on few component studies. The regression asymmetry test is generally more powerful (Egger *et al*. 1997; Sterne *et al*. 2000). A critical P level of at least 0.1 should be used for both tests.

Missing values

An option is provided for entry of the numbers of missing values of one variable. This may be appropriate in a 2x2 table that shows the relationship between two variables (A and B), the numbers of missing values of variable B being entered. Imputation (i.e., estimation of what would have been observed had the values not been missing) is then used, in order to estimate the proportions of "A yes" when B is "yes" or "no", and the difference between these proportions. This procedure is not appropriate for a comparison of preselected groups, where the categories of variable A are (for example) “cases” and “controls”.

The procedure assumes that the probability of being missing (“missingness”) of information on B is dependent only on the value of A, and the results may be misleading if it also depends on the true value of B. There is no need for the procedure if “missingness” is completely random, since the observed data can then be expected to provide unbiased results.

The program compares the proportions of missing values when A is “Yes” or “No”, in order to confirm that these proportions are significantly different. It then compares the proportions of “A yes” when B is "yes" or "no" (using imputation), and (using the observed data only) the proportions of “B yes” when A is "yes" or "no", and the odds ratio. A 95% confidence interval is computed in each instance

Inverse sampling

Inverse sampling refers to the addition of subjects to each group (A and B) until the group contains a prespecified number of cases (i.e., subjects with “Yes” findings). This method of sampling may be chosen because cases are rare. Since the addition of subjects to a group stops as soon as the required number of cases has been found, this method is appropriate only if subjects are accrued sequentially, and their exposure status can be determined rapidly. The program provides a *large-sample significance test*, the *difference between proportions*, *the ratio of proportions*, and the *odds ratio*. In some instances, zero frequencies prevent computation of the large-sample test and the standard error of the difference.

METHODS

Exact probabilities (single table)

The *exact tests* are based on the conditional probabilities, under the null hypothesis (given the marginal frequencies), of each possible number in a specific cell. Basic formulae are provided by Zar (1998: 545-555) and (for mid-P) by Rothman and Boice (1982: 25); or see Gahlinger and Abramson (1995: 36-37). In the computation, Stirling's approximation is used in computing the logarithms of factorials for large numbers (Rothman and Boice 1982: 26). Siegel and Castellan (1988). Code provided by A. Ray Simons (from his STAT22) formed the basis of the procedure employed here.

Overall’s continuity-corrected P is described by Overall (1990).

For *Tocher’s test* (Tocher 1950, Siegel 1956: 101), a random proportion *R* between 0 and 1 is selected, and if this falls below a critical level the null hypothesis is rejected. The critical level for any given *alpha* (0.05, 0.01, etc.) is

$$(alpha - XP) / OP$$

where OP = the conditional probability of the observed number of exposed cases
XP = the sum of the conditional probabilities for all more extreme numbers of exposed cases
The random proportion R is computed by the inbuilt Turbo Pascal procedures, augmented by an additional randomizing shuffle using the algorithm of Bays and Durham (Press et al. 1989: 215-217), followed by the use of the formula

$$\mathrm{frac}(9821R + 0.211327).$$

Exact probabilities (set of tables)

The computation of exact probabilities for stratified data is sped up by the employment of an efficient algorithm for calculating the coefficients of the conditional distribution (Martin and Austin 1991, 1996), using code from David O. Martin's public-domain EXACTBB program.

Chi-square test of association

The usual formulae are used for Pearson's chi-square test without and with Yates's correction (Zar 1998:483-494: formula 23.6 and 23.7 respectively).

Upton's chi-square (Upton 1982; D'Agostino 1990) is the Pearson chi-square multiplied by

$$(N - 1) / N$$

where N = sample size.

Haber's corrected chi-square (Haber 1980), as modified by Ghent, is described by Zar (1998: 494).

Mantel-Haenszel test

The formula for the Mantel-Haenszel chi-square is provided by (*inter alios*) Rothman (1986: formula 12: 58).

The criterion used to determine whether the sample sizes are sufficient to warrant use of this test is that the sum of the expected frequencies in each cell of the 2 x 2 table must be not less than 5 (Fleiss 1981: 175).

Heterogeneity tests and measures

The *heterogeneity chi-square tests* use formulae 12-59 of Rothman (1986) for ratios of proportions, Rothman's formula 12-60 for differences between proportions, and formula 10.35 of Fleiss (1981) for odds ratios.
The *measures of heterogeneity*, *H* and *I-squared*, are described by Higgins and Thompson (2002). *H* is computed by Higgins and Thompson's formula 6, and increased to 1, indicating absence of heterogeneity, if it less than 1. A test-based interval is computed by Method III. *I-squared* and its 95% interval are computed from *H*, by formula 10.

Equivalence tests

.
Equivalence tests, both for single comparisons and for stratified data, are performed by the method described by Yanagawa, Tango and Hiejima (1994).

Yule's *Q*, *phi* and *lambda*

Yule's Q can be calculated from the odds ratio OR:

$$Q = (OR - 1) / (OR + 1).$$

Phi is computed by formula 19.61 in Zar (1998), and *lambda* by formulae 9.37 and 9.39 of Siegel and Castellan (1988).

Ratio of proportions

Standard errors and confidence intervals for the ratio of proportions are based on formula 12-9 of Rothman (1986).

Jewell's low-bias estimator of the ratio of proportion A to proportion B (Jewell 1986) is

$$\text{proportion A} / \ |(b + 1) / (B + 1)|$$

where b and B are the numerator and denominator of proportion B.

Difference between proportions

Three sets of confidence intervals are computed for differences between proportions: continuity-corrected intervals calculated by Fleiss's procedure (Fleiss (1981: formula 2.14), which is appropriate for large samples only, and two sets using Wilson's score method (Wilson 1927), one without and one with a continuity correction. The latter two methods are described by Newcombe (1998) as methods 10 and 11. Formulae provided by Newcombe and Altman (2000: 49-50) are used for method 10. For method 11, the program computes the upper and lower confidence limits of the two proportions by formulae 1.26 and 1.27 of Fleiss (1981: 14), and substitutes them for l1, l2, u1 and u2 in Newcombe's formulae for L and U.

Odds ratio

The computation *of exact confidence intervals* for the odds ratios uses the network algorithm of Mehta et al. (1985). It is based (with permission) on Turbo Pascal code written by Eduardo Franco and Nelson Campos Filho, and used by A. Ray Simons in his EPIDORCS program.

Cornfield's approximation to confidence intervals for the odds ratio is computed by an iterative procedure described by Fleiss (1979). Since the Cornfield estimates are conditional on the marginal totals, the lower confidence limit may exceed zero even when the observed odds ratio is zero. In such instances the lower limit is displayed as zero, and in the reverse situation the upper limit is displayed as infinity. A continuity correction is made (Fleiss 1979: 171-172). The accuracy of the estimates is checked by the Mantel-Fleiss test (formulae 15.18 and 15.19 in Kleinbaum *et a*l. 1982).

Jewell's low-bias estimator of the odds ratio is described by Jewell (1986). The standard error of the log odds ratio is computed by formula 5.33 of Fleiss (1981: 67).

Overall ratio of proportions, difference between proportions, and odds ratio

For the *overall ratio of proportions*, the *precision-based* estimator and its confidence intervals are based on formulae 12-10, 12-11 and 12-46 of Rothman (1986). The *Mantel-Haenszel* estimator is computed by formula 12-27 of Rothman (1986), and its standard error and confidence are based on formulae provided by Greenland and Robins (1985). In *the DerSimonian-Laird* procedure (DerSimonian and Laird 1986) the log of the ratio of proportions for each stratum, and its standard error, are computed after changing any zero proportion to 0.0001
The *Q* statistic, which plays a central role in the calculation, is based on the data for separate strata and the Mantel-Haenszel estimator. If *Q* + 1 is less than the number of strata, the random-effects approach does not change the results.

For the *overall difference between proportions*, the *precision-based* estimator and its confidence intervals are based on formulae 12-8 and 12-51 of Rothman (1986). The *Mantel-Haenszel* estimator is based on formulae provided by Greenland and Robins (1985), and the standard error is computed by the method described by Sato (1989). The *DerSimonian-Laird* procedure is described by DerSimonian and Laird 1986. The *Q* statistic, which plays a central role in the calculation, is based on the data for separate strata and the Mantel-Haenszel estimator. If *Q* + 1 is less than the number of strata the random-effects and fixed-effect models yield identical results.

For the *overall odds ratio*, the computation *of exact confidence intervals* is sped up by the employment of an efficient algorithm for calculating the coefficients of the conditional distribution (Martin and Austin 1991, 1996), using code from David O. Martin's public-domain EXACTBB program. The *maximum-likelihood* estimates are described by Rothman (1986: 194-195) and Rothman and Boice (1982:5-6). The *Mantel-Haenszel* estimator of the common odds ratio is estimated by formula 10.47 of Fleiss (1981), and the estimation of its confidence intervals is described by Robins, Breslow and Greenland (1986) and by Rothman (1986: 219-220). The *Cornfield-Gart* procedure for confidence intervals is described by Fleiss (1979); its computation incorporates a continuity correction (Fleiss 1979: 171-172). The *DerSimonian-Laird* procedure described by Fleiss and Gross (1991) is used. For this purpose the log odds ratio for each stratum, and its standard error, are computed after adding 0.5 to each cell in the 2 x 2 table (Fleiss 1981: 165-166). The Q statistic, which plays a central role in the calculation, is based on these results and, as suggested by Petitti (1994: 111-113), on the Mantel-Haenszel estimate of the common odds ratio. Chi-square is

$$(DL / SE)^2$$

where DL is the DerSimonian-Laird estimator and
SE is its standard error.

If Q + 1 is less than the number of strata, the random-effects and fixed-effect models yield identical results.

Fail-safe N

The fail-safe N is computed by the following formula, which is derived from Orwin's effect-size formula (Orwin 1983; Hedges and Olkin 1985, formula 9, p. 306; or Iyengar and Greenhouse 1988, formula 5):

Fail-safe N = k[abs(R) - abs(C)] / abs{C}

where k = No. of studies included in the analysis
R = log of the overall odds ratio or risk ratio
C = log of the chosen "negligible value" [0.8, 0.9, 1.1 or 1.2).

Tests for a skewed funnel plot

The *regression asymmetry test* (Egger et al. 1997) uses linear regression. It regresses the standard normal deviate (SND), defined as the effect measure divided by its standard error) against precision (the inverse of the standard error of the effect measure). In both this test and the adjusted rank correlation test, the measure of effect is the log of the odds ratio; the adjusted odds ratio (0.5 added to each cell) is used if the odds ratio is zero or infinity. The regression equation is

SND = intercept + b x precision.

In the absence of bias, an intercept of zero is expected. The program reports the intercept and its 90% confidence interval, and tests its difference from zero; two-tailed P is displayed. The usual formulae for least-squares linear regression are used (e.g. Woolson and Clarke 2002: 309-311; Zar 1998: formula 17.21).

The *adjusted rank correlation test* (Begg and Mazumdar 1994) uses Kendall's rank correlation (Siegel and Castellan 1988: 245-54) to appraise the association between the sizes of the effects in the component studies (after first standardizing these effect measures) and their standard errors. In the absence of bias, a *tau* of zero is expected. Allowance is made for ties in the computation. If there are 30 or fewer component studies, tables of critical levels for one-tailed P = 0.05, 0.025, 0.01, and 0.005 (Siegel and Castellan 1988: Tables RI and RII) are used. If two-tailed P exceeds 0.01 according to these tables, and for larger samples, a Z test (making allowance for ties) is used (Armitage *et al.*. 2002: 290). The two-tailed P value is displayed.

Inverse sampling

The large-sample *significance test* for the difference between the two groups uses the formula (Lui, personal communication)

$$z = [\text{abs}(U) - 0.5 * (1 / DenA + 1 / DenB)] / \sqrt{V}$$

where U = the uniformly minimum variance unbiased estimate of the difference between proportions (Lui 2004: 32)
= $(xA - 1) / (DenA - 1) - (xB - 1) / (DenB - 1)$
V (variance) = $p(1 - p)$ [(1 / ($DenA$ - 2) + (1 / ($DenB$- 2)]
$p = (xA + xB - 1) / (DenA + DenB - 1)$
xA and xB = numbers of exposed subjects in samples A and B respectively
$DenA$ and $DenB$ = the numbers in samples A and B respectively.

For the *difference between proportions,* the program displays a standard error based on the pooled proportions (i.e., the square root of V), an unbiased estimate of the difference (U), and confidence intervals estimated by formula 2.37 of Lui (2004: 32) or (if there is a zero numerator) by formula 2.35 of Lui (2004: 31).

For the *ratio of proportions,* the program displays an unbiased estimate of the ratio (Lui 2004: 76, formula 4.25), and two sets of confidence intervals, computed by formulae 4.27 and 4.28 of Lui (2004: 77). The second confidence interval is an approximate one suggested by Bennett (1981), and (as stated in the output) it may be preferable if the numbers or proportions of cases are small (Lui 2004: 77). If there is a zero numerator, 0.5 is first added to each numerator.

Confidence intervals for the *odds ratio* are estimated by formula 5.52 of Lui (2004: 109). If there is a zero frequency, 0.5 is first added in each cell.

Missing values

The method of imputation used for estimating the proportions of “A yes” when B is “yes” or “no” is described by Fleiss *et al*. (2003: 498-499: formula 16.4). A 95% confidence interval for the difference between these proportions is estimated by formula 3.14 from the variance, which is computed by formula 16.6.

Confidence intervals for the difference between proportions of “A yes” and “B yes” (ignoring the missing values) are computed by Wilson’s score method (method 10 of Newcombe), as described by Newcombe and Altman (2000: 49). Confidence intervals for the odds ratio are computed by the method described by Morris and Gardner (2000: 59), after adding 0.5 to all cell frequencies if any cell frequency is zero.

B. COMPARISON OF RATES THAT HAVE NUMBER-OF-INDIVIDUALS DENOMINATORS

This module is appropriate for comparisons of rates that have number-of-individuals denominators (e.g. measures of prevalence, cumulative incidence, and risk), derived from observational studies (cross-sectional or cohort) or trials. The program can handle data collected by *inverse sampling* (see below).

It is not appropriate for rates with person-time denominators (for which module D of this program should be chosen). It is similar to module A, but provides extra results (attributable, prevented and preventable fractions, and the numbers needed to avoid or produce one case), and does not provide the equivalence tests that module A offers.

The findings in two groups can be compared, or a series of comparisons can be made in different strata or in different studies. For each comparison, data may be entered in a 2 x 2 table format, or as numerators (case frequencies) and denominators, or as rates and denominators.

For *stratified data*, enter the data for each stratum in turn, and then click on "*All strata*" to obtain the combined results, which permit appraisal of the association while controlling for confounding effects of the stratifying variable or variables, and assessment of heterogeneity.

For a *meta-analysis*, enter the data for each study in turn (as a separate stratum), and then click on "*All strata*" to compare and combine the results in separate studies.

To compare the changes observed in two groups in a *before-after study* with independent "before" and "after" observations, enter the before-after data for each group in turn, and then click on "All strata" for heterogeneity tests.

For *each separate comparison,* the program provides **exact probabilities** (Fisher's, mid-P and continuity-corrected), **chi-square tests of association**, the **ratio of the rates** (with its confidence intervals), the **difference between the rates** (with confidence intervals computed by a large-sample method and Wilson's score method), the **odds ratio** (with Fisher's exact, mid-P exact, and Cornfield's confidence intervals), **Yule's Q, phi and lambda**, **measures of the impact of exposure** (attributable, prevented and preventable fractions), and the **number needed to avoid or produce one case**.

For a *series of comparisons*, the program computes **exact probabilities** (Fisher's and mid-P). a **Mantel-Haenszel test**, **heterogeneity tests and measures,** estimators of the **overall ratio of rates, difference between rates. and odds ratio** (with their confidence intervals), **measures of the impact of exposure** (attributable, prevented and preventable fractions), the **number needed to avoid or produce one case,** and (for use in meta-analysis*)* estimates of the **fail-safe N** and two **tests for a skewed funnel plot** (suggesting publication bias).

For studies in which **inverse sampling** was used, the program provides a large-sample significance test and estimates the difference between rates or proportions, the ratio of rates or proportions, and the odds ratio.

Exact probabilities (*See page 6 for fuller details.*).

The program computes *one-tailed and two-tailed P-values* based on exact tests. A number of results are provided, since there is no consensus as to the best methods of computation. If these results lead to conflicting conclusions, inferences may require careful consideration.

Chi-square tests of association

For each table, a simple chi-square test of association is performed, supplemented by tests with Yates's, Upton's, and Haber's corrections to overcome the slight anticonservative bias of the uncorrected value. Yates's correction is criticized by many statisticians on the grounds that a test that uses it is too conservative, with an increased risk of a type II error (failure to reject the null hypothesis when, in fact, it is false). The other (smaller) modifications may be preferred; Zar (1998: 494) calls Haber's correction (as modified by Ghent) "excellent". If the tests lead to conflicting conclusions, inferences may require careful consideration.

Chi-square tests may be misleading if the expected frequencies (under the null hypothesis) are too small. The program displays a warning if there are cells with expected frequencies of less than 5.

Mantel-Haenszel test

This overall test of association controls for confounding effects of the stratifying variable or variables – e.g. of age and sex if the strata represent different age-sex groups. In a meta-analysis, it is an overall test, controlling for the differences between the studies.

The test is performed with and without a continuity correction. It is usually applied without a continuity correction.

The Mantel-Haenszel test is valid even for sparse data, provided that overall numbers are sufficient. A message is displayed if the overall numbers are too small to warrant use of the test.

Heterogeneity tests and measures

For stratified data (i.e., a series of tables), the program provides *heterogeneity tests* for the ratios of rates, the differences between rates, and the odds ratios in the different strata. These tests should be interpreted with caution, since their power is low; if the result is significant at the 0.05 level, the hypothesis of homogeneity can be rejected; but "a high p-value ... does not show that the measure is uniform, it only means that heterogeneity ... was not detected by the test" (Rothman and Greenland 1998: 276); the larger the strata, the more valid the test.

The program also provides two *measures of heterogeneity*, *H* and *I-squared*, with their approximate 95% intervals, for the ratios of rates, the differences between rates, and the odds ratios An *H* value of less than 1.2 suggests absence of noteworthy heterogeneity, whereas a value exceeding 1.5 suggests its presence, even if the heterogeneity test is not significant. *I-squared* expresses the proportion of variation that can be attributed to heterogeneity (in a meta-analysis, to interstudy variation) rather than to sampling error.

Estimates of the supposed common underlying value of the ratio of rates, the difference between rates, and the odds ratio (see below) are of questionable value if the findings in the various strata are

very disparate. If the results are not uniform, explorations of possible causes – e.g. associations with study design or quality or with the sizes or other characteristics of the samples – may be revealing

The uniformity or heterogeneity of the measures in the different strata can be appraised not only by these tests and measures, but by plotting the values and their confidence intervals graphically, and comparing them.

Ratio of rates

For each table, the program displays the ratio of the rate in group A to the rate in group B, with its standard error and 90%, 95% and 99% confidence intervals. This ratio is likely to be of interest in epidemiological studies of causal associations.

It also displays *Jewell's low-bias estimate* of the ratio of rates in the population, which draws attention to the tendency for the ratio in a sample, especially a small one, to misrepresent the true ratio in the population represented. A disadvantage is that this estimator is affected by the direction of computation: the reciprocal of the A:B estimator is not the same as the B:A estimator.

Difference between rates

For each table, the program displays the difference between the rates (the rate in group A minus the rate in group B), with its standard error and 90%, 95% and 99% confidence intervals computed by three different methods.

This difference is likely to be of interest in studies of the magnitude and economic aspects of public health problems. The confidence intervals are estimated by Fleiss's procedure (appropriate for large samples only), and by Wilson's score method, without and with a continuity correction. The latter methods are said to have advantages over the Fleiss intervals (Newcombe 1998).

Odds ratio

For each table, the program displays the odds ratio (the ratio of the odds in group A to the odds in group B, with its reciprocal), its 90%, 95% and 99% confidence intervals (Cornfield's intervals, and exact Fisher's and mid-P confidence intervals), and an adjusted estimate of the odds ratio (after adding 0.5 to each cell frequency).

It also displays *Jewell's low-bias estimate* of the odds ratio in the population, which draws attention to the tendency for the odds ratio in a sample, especially a small one, to misrepresent the true odds ratio in the population represented. A disadvantage is that (unlike the ordinary odds ratio) this estimator is affected by the direction of computation: the reciprocal of the A:B estimator is not the same as the B:A estimator (Walter and Cook 1991, Mantel 1992).

The odds ratio is likely to be of interest in epidemiological studies of causal associations.

Overall ratio of rates, difference between rates, and odds ratio

For a *series of table*s ("all strata"), the program computes three estimators of the *overall ratio of rates* and of the *overall difference between rates* (precision-based, Mantel-Haenszel, and DerSimonian-Laird estimators, with 90%, 95%, and 99% confidence intervals), and four estimators

of the *overall odds ratio* (conditional and unconditional maximum-likelihood estimators, a Mantel-Haenszel estimator, and a DerSimonian-Laird estimator, with 90%. 95%, and 99% exact Fisher's, exact mid-P, Mantel-Haenszel, Cornfield-Gart, and Dersimonian-Laird confidence intervals).

The ratio of rates and odds ratio are likely to be of interest in epidemiological studies of causal associations, and the difference between rates is likely to be of interest in studies of the magnitude and economic aspects of public health problems.

These estimates of the supposed common underlying values, and their confidence intervals, should be treated with caution if the findings in the various strata are very disparate. The heterogeneity tests have a low power, and are unreliable with small frequencies (Rothman 1986: 223).

The estimators other than the DerSimonian-Laird estimator are based on a *fixed-effect model*, which assumes that the strata provide estimates of the same true effect. The *precision-based* estimators are weighted means, using the reciprocal of the variance in each stratum as the weight for the stratum when the data are pooled, and are appropriate if the sample in each stratum is large; strata in which one of the proportions is zero do not enter into the calculation, and an appropriate message is displayed**.** The *Mantel-Haenszel* estimators are applicable even when there are sparse data in each stratum.

The *DerSimonian-Laird* estimator is based on a *random-effects* model, which assumes that the strata provide estimates of randomly differing effects. The variation between strata is taken into account, resulting in wider confidence intervals and a more conservative significance test. The random-effects model gives more weight to small studies, and may be inappropriate if sample sizes are very small. Some investigators use it when unexplained heterogeneity is present (Fleiss and Gross 1991, Petitti 1994, Whitehead and Whitehead 1991). "In essence," say Rothman and Greenland (1198: 668), "a random-effects model exchanges a doubtful homogeneity assumption for a fictitious random distribution of effects . The advantage ... is that the standard errors and confidence limits ... will more accurately reflect uncertainty about unaccounted-for sources of variation". The Dersimonian-Laird estimator is not displayed if this procedure does not increase the variance of the ratio of proportions. A chi-square test appraises whether the estimator differs from zero.

Yule's Q, phi and lambda

Yule's *Q*, *ph*i and *lambda* (measures of association) are displayed for each table.

Yule's Q (Zar 1998: 404 ranges from 0 (no association) to 1 (complete positive dependence) or -1 (complete negative dependence). In a 2 x 2 table it is equivalent to the *gamma* coefficient.

Phi (Zar 1998: 403) ranges from -1 (complete inverse dependence) to +1 (complete positive dependence); 0 indicates no association. It is not displayed if the expected frequency in one or more cells is <5. In a 2 x 2 table *phi* is equivalent to Cramer's coefficient *V.*

Goodman and Kruskal's *lambda (*Siegel and Castellan (1988: 298-303) is a coefficient of forecasting efficiency that expresses the capacity of one variable to "predict" the other. It is an assessment of the proportion of incorrect predictions concerning one variable that would be prevented if information about the other variable was available. Two *lambda* statistics are computed; one refers to the prediction of the variable whose categories are "Yes" or "No" when it is known to which group (A or B) the individual belongs, and the other refers to a prediction in the

reverse direction. *Lambda* ranges between 0 (if the one variable is of no help in predicting the other) to 1 (if the one variable perfectly specifies the categories of the other).

Unlike Yule's *Q*, (and the odds ratio), *phi* and *lambda* vary with the relative sizes of the two groups that are compared, and should in general be used only if these groups together make up a defined population, or comprise a representative sample of a defined population. The values of *phi* and *lambda* are then applicable to this specific population.

Measures of the impact of exposure

Measures of the impact of exposure (attributable, prevented, and preventable fractions) are computed, on the assumption that one group or sample (A) is exposed to a risk or protective factor, and the other (B) is not. For a risk factor, the program computes the *attributable fractions in the exposed and in the population*. For a protective factor, it computes the *prevented fractions in the exposed and in the population*, and *the preventable fraction in the population*. The attributable fraction is the proportion of the rate that can (under certain assumptions) be attributed to exposure to the factor, and the prevented fraction is the proportion of the hypothetical rate (in the absence of exposure) that has been prevented by exposure.

By default, the computation of the impact in the population assumes that the two groups are together representative of the population. Optionally (if this assumption is incorrect), the proportion of the population exposed to the risk or protective factor can be entered at the keyboard, as a percentage. This value is then used in the analysis, except in the overall analyses of stratified data, when the combined groups (in all strata) are assumed to represent the population.

The method used for stratified data is appropriate for sparse data as well as for samples with large numbers. The assumption is made that the risk ratio is uniform across the strata.

Measures of impact should be interpreted with caution (*see* Rockhill et al. 1998). They assume that exposure has a causal effect, and that confounding and other biases are absent. The preventable fraction is appropriate only if exposure is amenable to change.

Number needed to avoid or produce one case

For use in studies in which the numerators of the rates are numbers of *cases* of a disease or other condition, or a disease complication, or death, the program reports the number of individuals who are needed in the group with a lower rate, in order to avoid a single case.

In a clinical trial where the rate is lower in the treated group than in the control group, this is the *number needed to treat,* or the *number needed to treat (benefit)* or *NNTB* (Altman 1998), i.e. the number of patients who must be treated in order to prevent one event (Sinclair and Bracken 1994, Feinstein 1995). In a clinical trial that shows that a treatment has undesired effects (i.e., there is a lower rate in the control group), the number needed in the control group to avoid one case is also the *number needed to harm* or *NNTH* (the number of patients needed to be treated to produce one episode of harm) (Sackett *et al.* 1997: 149; Bjerre and LeLorier 2000).

In an observational study of exposure to a supposed causal factor, if the rate is lower in the exposed group the number needed is the number who need to be exposed in order to avoid one case; whereas if the rate is lower in the unexposed group it is the number whose exposure must be prevented in

order to prevent one event (assuming that the findings reflect a cause-effect relationship and that the causal factor and its effect are modifiable).

The program displays 95% confidence limits for the number needed; these limits are the reciprocals of the confidence limits for the rate difference. If one of the latter confidence limits is negative, one of the confidence limits of the number needed will also be negative. For example, if group B has a lower rate and the rate difference is 10 per 100 (i.e. 0.1), with a confidence interval of -5 to 25 per 100 (spanning zero), the number needed in group B to avoid one case is 10 (the reciprocal of 0.1), with a confidence interval of 4 to -20, an interval that includes infinity (the reciprocal of zero). Since a negative number needed to avoid a case is equivalent, if the sign in reversed, to the "number needed to harm", and this is the same as the number needed in the *other* group to avoid a case, the program reports this confidence interval as:

'4 to infinity in B, then from infinity down to 20 in A'

in the hope that this will avoid and not further confound confusion .(see Altman 1998). This formulation indicates a continuous interval, first from 4 to infinity in the group with the lower rate, and then extending down from infinity to 20 in the *other* group.

The program also provides a formula for estimating the number needed to avoid one case in a different group or population, using the ratio of rates observed in the study sample, on the assumption (not necessarily valid) that the same ratio of rates is applicable..

Fail-safe N

Estimates of the fail-safe N are provided for use in *meta-analyses*, as rough guides to the possible importance of the "file drawer problem", i.e. the exclusion of studies that were not published or were not found for other reasons.

The program computes the numbers of new "null" studies (those with an odds ratio or risk ratio of 1) that will suffice to bring the overall odds or risk ratio to a negligible level (0.8, 0.9, 1.1 or 1.2). No account is taken of P-values or the size of the null studies.

Fail-safe N estimates based on the P-values in the studies are provided by module I of this program.

Tests for a skewed funnel plot

Two tests for a skewed funnel plot are performed, for use in meta-analysis. The tests, which examine the association between the sizes of the effects in the component studies and their precision, are the *regression asymmetry test* and the *adjusted rank correlation test.*

In each test, a low P value suggests possible *publication bias*, although the "small-study effect" (Sterne *et al.* 2000) that it indicates may have some other cause, such as the use of higher-risk subjects in smaller studies, resulting in an association between sample size and the effect under consideration.

Both tests have a low power if they are based on few component studies. The regression asymmetry test is generally more powerful (Egger *et al.* 1997; Sterne *et al.* 2000). A critical P level of at least 0.1 should be used for both tests.

Inverse sampling

Inverse sampling refers to the addition of subjects to each group (A and B) until the group contains a prespecified number of cases (i.e., subjects with "Yes" findings). This method of sampling may be chosen because cases are rare. Since the addition of subjects to a group stops as soon as the required number of cases has been found, this method is appropriate only if subjects are accrued sequentially, and their exposure status can be determined rapidly. The program provides a *large-sample significance test*, the *difference between rates or proportions*, *the ratio of rates or proportions*, and the *odds ratio*.

METHODS

Exact probabilities (single table)
see page 11.

Exact probabilities (set of tables)
see page 11.

Chi-square test of association
see page 11.

Mantel-Haenszel test
see page 11.

Heterogeneity tests and measures
see page 11

Equivalence tests
see page 12.

Yule's *Q*, *phi* and *lambda*
see page 12.

Ratio of rates

Standard errors and confidence intervals for the ratio of proportions are based on formula 12-9 of Rothman (1986).

Jewell's low-bias estimator of the ratio of rate A to rate B (Jewell 1986) is

$$\text{rate A} / \; |(b+1)/(B+1)|$$

where b and B are the numerator and denominator of rate B.

Difference between rates

Three sets of confidence intervals are computed for differences between rates: continuity-corrected intervals calculated by Fleiss's procedure (Fleiss (1981: formula 2.14), which is appropriate for large samples only, and two sets using Wilson's score method (Wilson 1927), one without and one with a continuity correction. The latter two methods are described by Newcombe (1998) as methods 10 and 11. Formulae provided by Newcombe and Altman (2000: 49-40) are used for method 10. For method 11, the program computes the upper and lower confidence limits of the two proportions by formulae 1.26 and 1.27 of Fleiss (1981: 14), and substitutes them for l1, l2, u1 and u2 in Newcombe's formulae for L and U.

Odds ratio
see page 12.

Overall ratio of rates, difference between rates, and odds ratio

For the *overall ratio of rates*, the *precision-based* estimator and its confidence intervals are based on formulae 12-10, 12-11 and 12-46 of Rothman (1986). The *Mantel-Haenszel* estimator is computed by formula 12-27 of Rothman (1986), and its standard error and confidence are based on formulae provided by Greenland and Robins (1985). In *the DerSimonian-Laird* procedure (DerSimonian and Laird 1986) the log of the ratio of rates for each stratum, and its standard error, are computed after changing any zero proportion to 0.0001 The *Q* statistic, which plays a central role in the calculation, is based on the data for separate strata and the Mantel-Haenszel estimator. If *Q* + 1 is less than the number of strata, the random-effects approach does not change the results.

For the *overall difference between rates*, the *precision-based* estimator and its confidence intervals are based on formulae 12-8 and 12-51 of Rothman (1986). The *Mantel-Haenszel* estimator is based on formulae provided by Greenland and Robins (1985), and the standard error is computed by the method described by Sato (1989). The *DerSimonian-Laird* procedure is described by DerSimonian and Laird 1986. The *Q* statistic, which plays a central role in the calculation, is based on the data for separate strata and the Mantel-Haenszel estimator. If *Q* + 1 is less than the number of strata the random-effects and fixed-effect models yield identical results.

For the *overall odds ratio*, the computation *of exact confidence intervals* is sped up by the employment of an efficient algorithm for calculating the coefficients of the conditional distribution (Martin and Austin 1991, 1996), using code from David O. Martin's public-domain EXACTBB program. The *maximum-likelihood* estimates are described by Rothman (1986: 194-195) and Rothman and Boice (1982:5-6). The *Mantel-Haenszel* estimator of the common odds ratio is estimated by formula 10.47 of Fleiss (1981), and the estimation of its confidence intervals is described by Robins, Breslow and Greenland (1986) and by Rothman (1986: 219-220). The *Cornfield-Gart* procedure for confidence intervals is described by Fleiss (1979); its computation incorporates a continuity correction (Fleiss 1979: 171-172). The *DerSimonian-Laird* procedure described by Fleiss and Gross (1991) is used. For this purpose the log odds ratio for each stratum, and its standard error, are computed after adding 0.5 to each cell in the 2 x 2 table (Fleiss 1981: 165-166). The Q statistic, which plays a central role in the calculation, is based on these results and, as suggested by Petitti (1994: 111-113), on the Mantel-Haenszel estimate of the common odds ratio. Chi-square is

$$(DL / SE)^2$$

where DL is the DerSimonian-Laird estimator and
SE is its standard error.

If Q + 1 is less than the number of strata, the random-effects and fixed-effect models yield identical results.

Measures of the impact of exposure

Attributable and prevented fractions in the exposed are calculated from the ratio of rates (R); for stratified data, the Mantel-Haenszel estimator is used. The formulae are

$$(R - 1) / R$$

for the attributable fraction, and

$$1 - R$$

for the prevented fraction.
Confidence intervals are estimated by replacing R in these formulae with its upper and lower confidence limits.

If the data are *not stratified*, or refer to a *single stratum*, the *attributable, prevented and preventable fractions in the population* are calculated from the ratio of rates (R) and the proportion exposed to the risk or protective factor in the population (E). By default, E is computed from the data entered for the two groups, on the assumption that together they represent the population (or population stratum). Optionally (if this assumption is incorrect), E can be entered at the keyboard, as a percentage. The formulae are

$$E(R - 1) / [E(R - 1) + 1]$$

for the population attributable fraction (AFp), and

$$E(1 - R)$$

for the population prevented fraction (PFp). For a risk factor, the preventable fraction (if appropriate) is the same as the attributable fraction; for a protective factor, it is calculated in the same way as the attributable fraction, after relabelling the exposed as "unexposed" and the unexposed as "exposed". Confidence intervals are estimated by the same formulae, but replacing R with its lower and upper confidence limits (Daly 1998). These intervals should be regarded as approximate, since uncertainty of the exposure proportion E is not taken into account (Greenland 1999); E is regarded as a fixed value.

For *stratified data*, attributable, prevented and preventable fractions in the total population are computed from the Mantel-Haenszel ratio of rates and the proportion of cases (i.e., individuals enumerated in the numerators of the exposed and unexposed groups) exposed to the risk or protective factor. These proportions are computed by combining the numbers entered for the two groups in each stratum, without reference to any values entered for E (see above), on the assumption that the combined groups represent the population. Formula 16-24 of Rothman and Greenland (1998: 296) is used for the attributable fraction in the population (AFp); if its value is negative, it is converted to the corresponding preventive fraction (see conversion equation, below). For the *preventable fraction*, the same formula is used as for the attributable fraction, after relabelling the exposed and unexposed. For stratified data, confidence intervals are computed from the variance of ln(1 - AFp), using formula 16-25 of Rothman and Greenland (1998: 296).

If the lower confidence limit of an attributable fraction (AF) is negative, the prevented fraction (PF) equivalent to this negative value is shown; and vice versa. The conversion equations used for this purpose are:

$$PF = 1 - 1 / (1 - AF)$$

and $$AF = 1 - 1 / (1 - PF).$$

In some instances confidence intervals are not shown because zero values interfere with their computation.

Number needed to avoid or produce one case

The number needed is the reciprocal of the rate difference. In a stratified analysis, it is the reciprocal of the Mantel-Haenszel rate difference..

The 95% confidence limits for the number needed are the reciprocals of the 95% confidence limits for the rate difference. In a single comparison, the continuity-corrected Wilson-score confidence interval is used.

The formula for estimating the number needed in group A or B (depending on whether the risk ratio exceeds 1 or is less than 1) to avoid one case in a different population, on the assumption that the ratio of rates observed in the study sample remains appropriate (Chatellier *et al.* 1996; Smeeth *et al.* 1999), is.

$$X / P,$$

where $X = 1 / (RR - 1)$
RR = the ratio of rates

Fail-safe N

The fail-safe N is computed by the following formula, which is derived from Orwin's effect-size formula (Orwin 1983; Hedges and Olkin 1985, formula 9, p. 306; or Iyengar and Greenhouse 1988, formula 5):

$$\text{Fail-safe N} = k[abs(R) - abs(C)] / abs\{C\}$$

where k = No. of studies included in the analysis
R = log of the overall odds ratio or ratio of rates
C = log of the chosen "negligible value" [0.8, 0.9, 1.1 or 1.2).

Tests for a skewed funnel plot

The *regression asymmetry test* (Egger *et al.* 1997) uses linear regression. It regresses the standard normal deviate (SND), defined as the effect measure divided by its standard error) against precision (the inverse of the standard error of the effect measure). In both this test and the adjusted rank correlation test, the measure of effect is the log of the odds ratio; the adjusted odds ratio (0.5 added to each cell) is used if the odds ratio is zero or infinity. The regression equation is

$$SND = \text{intercept} + b \text{ x precision}.$$

In the absence of bias, an intercept of zero is expected. The program reports the intercept and its 90% confidence interval, and tests its difference from zero; two-tailed P is displayed. The usual formulae for least-squares linear regression are used (e.g. Woolson and Clarke 2002: 309-311; Zar 1998: formula 17.21).

The *adjusted rank correlation test* (Begg and Mazumdar 1994) uses Kendall's rank correlation (Siegel and Castellan 1988: 245-54) to appraise the association between the sizes of the effects in the component studies (after first standardizing these effect measures) and their standard errors. In the absence of bias, a *tau* of zero is expected. Allowance is made for ties in the computation. If there are 30 or fewer component studies, tables of critical levels for one-tailed P = 0.05, 0.025, 0.01, and 0.005 (Siegel and Castellan 1988: Tables RI and RII) are used. If two-tailed P exceeds 0.01 according to these tables, and for larger samples, a Z test (making allowance for ties) is used (Armitage *et al.*. 2002). The two-tailed P value is displayed.

Inverse sampling

The large-sample *significance test* for the difference between the two groups uses the formula (Lui, personal communication)

$$z = [\text{abs}(U) - 0.5 * (1 / DenA + 1 / DenB)] / \sqrt{V}$$

where U = the uniformly minimum variance unbiased estimate of the difference between proportions (Lui 2004: 32)
$= (xA - 1) / (DenA - 1) - (xB - 1) / (DenB - 1)$
V (variance) $= p(1 - p) [(1 / (DenA - 2) + (1 / (DenB - 2)]$
$p = (xA + xB - 1) / (DenA + DenB - 1)$
xA and xB = numbers of exposed subjects in samples A and B respectively
$DenA$ and $DenB$ = the numbers in samples A and B respectively.

For the *difference between rates or proportions,* the program displays a standard error based on the pooled proportions (i.e., the square root of V), an unbiased estimate of the difference (U), and confidence intervals estimated by formula 2.37 of Lui (2004: 32) or (if there is a zero numerator) by formula 2.35 of Lui (2004: 31).

For the *ratio of rates or proportions,* the program displays an unbiased estimate of the ratio (Lui 2004: 76, formula 4.25), and two sets of confidence intervals, computed by formulae 4.27 and 4.28 of Lui (2004: 77). The second confidence interval is an approximate one suggested by Bennett (1981), and (as stated in the output) it may be preferable if the numbers or proportions of cases are small (Lui 2004: 77). If there is a zero numerator, 0.5 is first added to each numerator.

Confidence intervals for the *odds ratio* are estimated by formula 5.52 of Lui (2004: 109). If there is a zero frequency, 0.5 is first added in each cell.

C. COMPARISON OF EXPOSURE TO A RISK/PROTECTIVE FACTOR (IN A CASE-CONTROL STUDY)

This module is designed for the analysis of studies that compare cases and unmatched controls with respect to the proportions exposed to a supposed risk or protective factor. It might be used in the evaluation of a therapeutic or preventive procedure, by comparing the proportions who had been exposed to the procedure. The program can handle data collected by *inverse sampling* (see below).

The factor must be dichotomous ("exposed" or "not exposed"). If there are degrees of exposure, module G of this program should be used. This module (C) is not appropriate for case-control studies that use person-time denominators; module D provides limited results for such studies.

A single group of cases can be compared with a single group of controls, or a series of case-control comparisons can be made in different strata or in different studies. Each comparison requires the entry of data in a 2 x 2 table format. Optionally, the prevalence of exposure in the population or the relevant population stratum can also be entered, for use in appraising the impact of exposure in the population.

For *stratified data*, enter the data for each stratum in turn, and then click on "*All strata*" to obtain the combined results, which permit appraisal of the association while controlling for confounding effects of the stratifying variable or variables, and assessment of heterogeneity.

For a *meta-analysis*, enter the data for each study in turn (as a separate stratum), and then click on "*All strata*" to compare and combine the results in separate studies.

For *each separate comparison,* the program provides **exact probabilities** (Fisher's, mid-P and continuity-corrected, **chi-square tests of association**, the **odds ratio** (with Fisher's exact, mid-P exact, and Cornfield's confidence intervals), **Yule's Q, phi and lambda**, **measures of the impact of exposure** (attributable, prevented and preventable fractions), and the **number needed to avoid one case**.

For a *series of comparisons*, the program computes **exact probabilities** (Fisher's and mid-P). a **Mantel-Haenszel test**, **heterogeneity tests and measures,** estimators of the **overall odds ratio** (a conditional maximum-likelihood estimator, with exact Fisher's and mid-P confidence intervals, and unconditional maximum-likelihood, Mantel-Haenszel and Dersimonian-Laird estimators, with Mantel-Haenszel, Cornfield-Gart and Dersimonian-Laird confidence intervals), **measures of the impact of exposure** (attributable, prevented and preventable fractions), the **number needed to avoid one case,** and (for use in *meta-analysis)* estimates of the **fail-safe N** and two **tests for a skewed funnel plot** (suggesting publication bias)

For studies in which **inverse sampling** was used, the program provides a large-sample significance test and estimates the odds ratio and the attributable or prevented fraction.

Exact probabilities (*See page 6 for fuller details.*).

The program computes *one-tailed and two-tailed P-values* based on exact tests. A number of results are provided, since there is no consensus as to the best methods of computation. If these results lead to conflicting conclusions, inferences may require careful consideration.

Chi-square tests of association

For each table, a simple chi-square test of association is performed, supplemented by tests with Yates's, Upton's, and Haber's corrections to overcome the slight anticonservative bias of the uncorrected value. Yates's correction is criticized by many statisticians on the grounds that a test that uses it is too conservative, with an increased risk of a type II error (failure to reject the null hypothesis when, in fact, it is false). The other (smaller) modifications may be preferred; Zar (1998: 494) calls Haber's correction (as modified by Ghent) "excellent". If the tests lead to conflicting conclusions, inferences may require careful consideration.

Chi-square tests may be misleading if the expected frequencies (under the null hypothesis) are too small. The program displays a warning if there are cells with expected frequencies of less than 5.

Mantel-Haenszel test

This overall test of association controls for confounding effects of the stratifying variable or variables – e.g. of age and sex if the strata represent different age-sex groups. In a meta-analysis, it is an overall test, controlling for the differences between the studies.

The test is performed with and without a continuity correction. It is usually applied without a continuity correction.

The Mantel-Haenszel test is valid even in sparse data, provided that overall numbers are sufficient. A message is displayed if the overall numbers are too small to warrant use of the test.

Heterogeneity tests and measures

For stratified data (i.e., a series of tables), the program provides *heterogeneity tests* that compare the odds ratios in the different strata. These tests should be interpreted with caution, since their power is low; if the result is significant at the 0.05 level, the hypothesis of homogeneity can be rejected; but "a high p-value ... does not show that the measure is uniform, it only means that heterogeneity ... was not detected by the test" (Rothman and Greenland 1998: 276); the larger the strata, the more valid the test.

The program also provides two *measures of heterogeneity*, *H* and *I-squared*, with their approximate 95% intervals. An *H* value of less than 1.2 suggests absence of noteworthy heterogeneity, whereas a value exceeding 1.5 suggests its presence, even if the heterogeneity test is not significant. *I-squared* expresses the proportion of variation that can be attributed to heterogeneity (in a meta-analysis, to interstudy variation) rather than to sampling error.

Estimates of the supposed common underlying value of the odds ratio (see below) are of questionable value if the findings in the various strata are very disparate. If the results are not uniform, explorations of possible causes - e.g. associations with study design or quality or with the sizes or other characteristics of the samples - may be revealing

The uniformity or heterogeneity of the odds ratios in the different strata can be appraised not only by these tests and measures, but by plotting the odds ratios and their confidence intervals graphically, and comparing them.

Odds ratio

For each table, the program displays the odds ratio (the ratio of the odds in group A to the odds in group B, with its reciprocal), its 90%, 95% and 99% confidence intervals (Cornfield's intervals, and exact Fisher's and mid-P confidence intervals), and an adjusted estimate of the odds ratio (after adding 0.5 to each cell frequency).

It also displays *Jewell's low-bias estimate* of the odds ratio in the population, which draws attention to the tendency for the odds ratio in a sample, especially a small one, to misrepresent the true odds ratio in the population represented. A disadvantage is that (unlike the ordinary odds ratio) this estimator is affected by the direction of computation: the reciprocal of the A:B estimator is not the same as the B:A estimator (Walter and Cook 1991, Mantel 1992).

The odds ratio is likely to be of interest in epidemiological studies of causal associations.

Overall odds ratio

For a *series of table*s ("all strata"), the program computes four estimators of the *overall odds ratio* (conditional and unconditional maximum-likelihood estimators, a Mantel-Haenszel estimator, and a DerSimonian-Laird estimator, with 90%. 95%, and 99% exact Fisher's, exact mid-P, Mantel-Haenszel, Cornfield-Gart, and Dersimonian-Laird confidence intervals).

These estimates of the supposed common underlying value, and their confidence intervals, should be treated with caution if the findings in the various strata are very disparate. The heterogeneity tests have a low power, and are unreliable with small frequencies (Rothman 1986: 223).

The estimators other than the DerSimonian-Laird estimator are based on a *fixed-effect model*, which assumes that the strata provide estimates of the same true effect. The *precision-based* estimators are weighted means, using the reciprocal of the variance in each stratum as the weight for the stratum when the data are pooled, and are appropriate if the sample in each stratum is large; strata in which one of the proportions is zero do not enter into the calculation, and an appropriate message is displayed**.** The *Mantel-Haenszel* estimators are applicable even when there are sparse data in each stratum.

The *DerSimonian-Laird* estimator is based on a *random-effects* model, which assumes that the strata provide estimates of randomly differing effects. The variation between strata is taken into account, resulting in wider confidence intervals and a more conservative significance test. The random-effects model gives more weight to small studies, and may be inappropriate if sample sizes are very small. Some investigators use it when unexplained heterogeneity is present (Fleiss and Gross 1991, Petitti 1994, Whitehead and Whitehead 1991). "In essence," say Rothman and Greenland (1198: 668), "a random-effects model exchanges a doubtful homogeneity assumption for a fictitious random distribution of effects . The advantage ... is that the standard errors and confidence limits ... will more accurately reflect uncertainty about unaccounted-for sources of variation". The Dersimonian-Laird estimator is not displayed if this procedure does not increase the variance of the odds ratio. A chi-square test is reported, appraising whether the estimator differs from zero.

Miettinen's two *standardized odds ratios* ("standardized rate ratios") are also computed, first taking the exposed subjects as the standard ("ŜMR"), and then taking the unexposed as the standard ("ŜRR").

Yule's Q, phi and lambda

Yule's *Q*, *ph*i and *lambda* (measures of association) are displayed for each table.

Yule's Q (Zar 1998:404) ranges from 0 (no association) to 1 (complete positive dependence) or -1 (complete negative dependence). In a 2 x 2 table it is equivalent to the *gamma* coefficient.

Phi (Zar 1998: 403) ranges from -1 (complete inverse dependence) to +1 (complete positive dependence); 0 indicates no association. It is not displayed if the expected frequency in one or more cells is <5. In a 2 x 2 table *phi* is equivalent to Cramer's coefficient *V*.

Goodman and Kruskal's *lambda (*Siegel and Castellan (1988: 298-303) is a coefficient of forecasting efficiency that expresses the capacity of one variable to "predict" the other. It is an assessment of the proportion of incorrect predictions concerning one variable that would be prevented if information about the other variable was available. Two *lambda* statistics are computed; one refers to the prediction of the variable whose categories are "Yes" or "No" when it is known to which group (A or B) the individual belongs, and the other refers to a prediction in the reverse direction. *Lambda* ranges from 0 (if the one variable is of no help in predicting the other) to 1 (if the one variable perfectly specifies the categories of the other).

Unlike Yule's *Q*, (and the odds ratio), *phi* and *lambda* vary with the relative sizes of the two groups that are compared, and should in general be used only if these groups together make up a defined population, or comprise a representative sample of a defined population. The values of *ph*i and *lambda* are then applicable to this specific population.

Measures of the impact of exposure

If exposure is to a risk factor, the program computes the attributable fractions in the exposed and in the population. If exposure is to a protective factor, it computes the prevented fractions in the exposed and in the population. The results are valid if the odds ratio is an appropriate estimator of the risk ratio. The attributable fraction is the proportion of the rate that can (under certain assumptions) be attributed to exposure to the factor, and the prevented fraction is the proportion of the hypothetical rate (in the absence of exposure) that has been prevented by exposure.

By default, the computation of the impact in the population assumes that the cases and controls are together representative of the population. Optionally (if this assumption is incorrect), the proportion of the population exposed to the risk or protective factor can be entered at the keyboard, as a percentage. This value is then used in the analysis, except in the overall analyses of stratified data, when the combined cases and controls (in all strata) are assumed to represent the population.

The method used for stratified data is appropriate for sparse data as well as for samples with large numbers. The assumption is made that the odds ratio is uniform across the strata.

Measures of impact should be interpreted with caution (*see* Rockhill et al. 1998). They assume that exposure has a causal effect, and that confounding and other biases are absent. .

Number needed to avoid one case

The program provides a formula for calculating the approximate number of person-time units of exposure or non-exposure (depending on the direction of the effect) needed to avoid one case. The formula uses the assumed rate in the non-exposed, and is based on the assumptions that the odds ratio in the study sample (or the Mantel-Haenszel odds ratio, for stratified data) is an appropriate estimate of the incidence density ratio, and that the findings reflect a cause-effect relationship.

If the exposure under consideration is to a therapeutic or preventive procedure, this number is the *number needed to treat to prevent one cas*e (*NNT*) or the *number needed to treat to produce one episode of harm* (*NNTH*) , depending on the direction of the effect (Sackett *et al.* 1997; Bjerre and LeLorier 2000).

Fail-safe N

Estimates of the fail-safe N are provided for use in *meta-analyses*, as rough guides to the possible importance of the "file drawer problem", i.e. the exclusion of studies that were not published or not found for other reasons.

The program computes the numbers of new "null" studies (those with an odds ratio of 1) that will suffice to bring the overall odds ratio to a negligible level (0.8, 0.9, 1.1 or 1.2). No account is taken of P-values or the size of the null studies.

Fail-safe N estimates based on P-values are provided by module I of this program.

Tests for a skewed funnel plot

Two tests for a skewed funnel plot are performed, for use in meta-analysis. The tests, which examine the association between the sizes of the effects in the component studies and their precision, are the *regression asymmetry test* and the *adjusted rank correlation test.*

In each test, a low P value suggests possible *publication bias*, although the "small-study effect" (Sterne *et al.* 2000) that it indicates may have some other cause, such as the use of higher-risk subjects in smaller studies, resulting in an association between sample size and the effect under consideration.

Both tests have a low power if they are based on few component studies. The regression asymmetry test is generally more powerful (Egger *et al.* 1997; Sterne *et al.* 2000). A critical P level of at least 0.1 should be used for both tests.

Inverse sampling

Inverse sampling refers to the addition of subjects to each group (cases and controls) until the group contains a prespecified number of subjects exposed to the risk or protective factor under study. This method of sampling may be chosen because exposed subjects are rare. Since the addition of subjects to a group stops as soon as the required number of exposed subjects has been found, this method is appropriate only if subjects are accrued sequentially, and their exposure status can be determined rapidly. The program provides a *large-sample significance test*, the *odds ratio*, and the *attributable or prevented fraction* (see ”Measures of the impact of exposure”, above).

METHODS

Exact probabilities (single table)
see page 11.

Exact probabilities (set of tables):
see page 11.

Chi-square test of association
see page 11.

Mantel-Haenszel test
see page 11.

Yule's *Q*, *phi* and *lambda*
see page 12.

Heterogeneity test and measures

The *heterogeneity chi-square test* uses formula 10.35 of Fleiss (1981).

The *measures of heterogeneity*, *H* and *I-squared*, are described by Higgins and Thompson (2002). *H* is computed by Higgins and Thompson's formula 6, and increased to 1, indicating absence of heterogeneity, if it less than 1. A test-based interval is computed by Method III. *I-squared* and its 95% interval are computed from *H*, by formula 10.

Odds ratio
see page 12.

Overall odds ratio

For the *overall odds ratio*, the computation *of exact confidence intervals* is sped up by the employment of an efficient algorithm for calculating the coefficients of the conditional distribution (Martin and Austin 1991, 1996), using code from David O. Martin's public-domain EXACTBB program. The *maximum-likelihood* estimates are described by Rothman (1986: 194-195) and Rothman and Boice (1982:5-6). The *Mantel-Haenszel* estimator of the common odds ratio is estimated by formula 10.47 of Fleiss (1981), and the estimation of its confidence intervals is described by Robins, Breslow and Greenland (1986) and by Rothman (1986: 219-220). The *Cornfield-Gart* procedure for confidence intervals is described by Fleiss (1979); its computation incorporates a continuity correction (Fleiss 1979: 171-172). The *DerSimonian-Laird* procedure described by Fleiss and Gross (1991) is used. For this purpose the log odds ratio for each stratum, and its standard error, are computed after adding 0.5 to each cell in the 2 x 2 table (Fleiss 1981: 165-166). The Q statistic, which plays a central role in the calculation, is based on these results and, as suggested by Petitti (1994: 111-113), on the Mantel-Haenszel estimate of the common odds ratio. Chi-square is

$$(DL / SE)^2$$

where DL is the DerSimonian-Laird estimator and
SE is its standard error.

If Q + 1 is less than the number of strata, the random-effects and fixed-effect models yield identical results.

Miettinen's two *standardized odds ratios* ("standardized rate ratios") (Miettinen 1972) are computed by formulae 5 and 6 of Rothman and Boice (1979: 6).

Measures of the impact of exposure

Attributable and prevented fractions in the exposed are calculated from the odds ratio (OR); for stratified data, the Mantel-Haenszel odds ratio is used. The formulae are

$$(OR - 1) / OR$$

for the attributable fraction, and

$$1 - OR$$

for the prevented fraction. Confidence intervals are estimated by replacing OR in these formulae with its upper and lower confidence limits, using exact mid-P (if available) or Cornfield limits, or (for stratified data) Mantel-Haenszel limits.

If the data are *not stratified*, or refer to a *single stratum*, the *attributable and prevented fractions in the population* are calculated from the odds ratio (OR) and the proportion exposed to the risk or protective factor in the population (E). By default, E is computed from the data entered for the controls, on the assumption that it represents the population (or population stratum). Optionally (if this assumption is incorrect), E can be entered at the keyboard, as a percentage. The formulae are

$$E(OR - 1) \ / \ [E(OR - 1) + 1]$$

for the population attributable fraction (AFp), and

$$E(1 - OR)$$

for the population prevented fraction (PFp). Confidence intervals are estimated by the same formulae, but replacing OR with its lower and upper confidence limits (Daly 1998). These intervals should be regarded as approximate, since uncertainty of the exposure proportion E is not taken into account (Greenland 1999); E is regarded as a fixed value.

For *stratified data*, the *attributable and prevented fractions in the total population* are computed from the Mantel-Haenszel odds ratio and the proportion of cases exposed to the risk or protective factor, computed by combining the numbers entered in each stratum, without reference to any values entered for E (see above), on the assumption that the combined entries represent the population. Formula 16-24 of Rothman and Greenland (1998: 296) is used for the attributable fraction in the population (AFp); if its value is negative, it is converted to the corresponding preventive fraction (see conversion equation, below). For stratified data, confidence intervals are computed from the variance of ln(1 - AFp), using formula 16-25 of Rothman and Greenland (1998: 296).

If the lower confidence limit of an attributable fraction (AF) is negative, the prevented fraction (PF) equivalent to this negative value is shown; and vice versa. The conversion equations used for this purpose are:

$$PF = 1 - 1 / (1 - AF)$$

and $$AF = 1 - 1 / (1 - PF).$$

In some instances confidence intervals are not shown because zero values interfere with their computation.

Number needed to avoid one case

The formula (derived from Bjerre and LeLorier 2000, appendix) is

$1 / [R(1 - OR)]$ if the odds ratio (OR) is less than 1, and

$1 / [R(OR - 1)]$ if the odds ratio exceeds 1

where R is the assumed rate of events per 1000 person-time units in the non-exposed.

The Mantel-Haenszel odds ratio is used for stratified data.

Fail-safe N

The fail-safe N is computed by the following formula, which is derived from Orwin's effect-size formula (Orwin 1983; Hedges and Olkin 1985, formula 9, p. 306; or Iyengar and Greenhouse 1988, formula 5):

$$\text{Fail-safe } N = k[\text{abs}(R) - \text{abs}(C)] / \text{abs}\{C\}$$

where k = No. of studies included in the analysis
R = log of the overall odds ratio
C = log of the chosen "negligible value" [0.8, 0.9, 1.1 or 1.2).

Tests for a skewed funnel plot

see page 22.

Inverse sampling

The large-sample *significance test* for the difference between cases and controls uses the formula (Lui, personal communication)

$$z = [\text{abs}(U) - 0.5 * (1 / DenA + 1 / DenB)] / \sqrt{V}$$

where U = the uniformly minimum variance unbiased estimate of the difference between proportions (Lui 2004: 32)
$= (xA - 1) / (DenA - 1) - (xB - 1) / (DenB - 1)$
V (variance) $= p(1 - p)\ [(1 / (DenA - 2) + (1 / (DenB - 2)]$
$p = (xA + xB - 1) / (DenA + DenB - 1)$
xA and xB = numbers of exposed subjects among cases and controls respectively
$DenA$ and $DenB$ = the numbers of cases and controls respectively.

Confidence intervals for the *odds ratio* are estimated by formula 5.52 of Lui (2004: 109). If there is a zero frequency, 0.5 is first added in each cell.

The *attributable fraction in the population* (AF) is computed by the formula provided by Lui (2004: 157). Its confidence intervals are estimated by formula 7.47 (Lui 2004: 157), unless the proportion of exposed in the population (E) is entered, in which case they are computed by substituting the lower and upper confidence limits of the odds ratio, estimated by formula 5.52 of Lui (2004: 109), for OR in the formula

$$AF = E\ (OR - 1) / [1 + E\ (OR - 1)].$$

If the latter method is used, the confidence intervals should be regarded as approximate, since uncertainty of the exposure proportion E is not taken into account (Greenland 1999); E is regarded as a fixed value.
If AF is less than zero, it is converted to the equivalent *prevented fraction* (PF) by the formula

$$PF = 1 - 1 / (1 - AR)$$

If there is a zero frequency, 0.5 is added in each cell before computing the attributable or prevented fraction.

D. COMPARISON OF RATES THAT HAVE PERSON-TIME DENOMINATORS

This module is designed for the analysis of cohort studies or trials that compare two incidence, mortality, or other rates that have person-time denominators.

The findings in two groups can be compared, or a series of comparisons can be made in different strata or in different studies. For each comparison, data may be entered as numerators (case frequencies) or rates, with their denominators. Optionally, the prevalence of exposure in the population or the relevant population stratum can also be entered, for use in appraising the impact of exposure in the population.

For *stratified data*, enter the data for each stratum in turn, and then click on "*All strata*" to obtain the combined results, which permit appraisal of the association while controlling for confounding effects of the stratifying variable or variables, and assessment of heterogeneity.

For a *meta-analysis*, enter the data for each study in turn (as a separate stratum), and then click on "*All strata*" to compare and combine the results in separate studies.

For *each separate comparison*, the program provides **exact probabilities** (Fisher's and mid-P), the **rate ratio** (with exact, Cornfield's and Breslow's confidence intervals), the **rate difference** (with confidence intervals), the **number needed to avoid or produce one case**, and **measures of the impact of exposure** (attributable, prevented and preventable fractions)

For a *series of comparisons*, the program computes **exact probabilities** (Fisher's and mid-P). a **Mantel-Haenszel test**, **heterogeneity tests and measures**, **a test for trend**, the **overall rate ratio** (with exact Fisher's and mid-P, Cornfield, Breslow and Mantel-Haenszel and Dersimonian-Laird confidence intervals)**, the overall rate difference** (with precision-based and Mantel-Haenszel confidence intervals), **measures of the impact of exposure** (attributable, prevented and preventable fractions), the **number needed to avoid one case,** and (for use in *meta-analysis)* estimates of the **fail-safe N** and two **tests for a skewed funnel plot** (suggesting publication bias) .

The program also provides a **comparison of two numbers of events**, treated as Poisson variates. For example, it could test the equality of numbers of deaths or other relatively rare events occurring in the same population in two equal time periods; denominators are not required.

Exact probabilities

The program computes exact P values (Fisher's and mid-P, one-tailed and two-tailed). Fisher's P has a conservative bias, which may be important when sample sizes are small. Many statisticians prefer the mid-P value, especially when results from several studies are combined (Armitage *et al.* (2002: 137)

Mantel-Haenszel test

This overall test of association controls for confounding effects of the stratifying variable or variables – e.g. of age and sex if the strata represent different age-sex groups. In a meta-analysis, it is an overall test, controlling for the differences between the studies.

The Mantel-Haenszel test is valid even for sparse data, provided that overall numbers are sufficient. A message is displayed if the overall numbers are too small to warrant use of the test.

Heterogeneity tests and measures

For stratified data (i.e., a series of tables), the program provides *heterogeneity tests* for the rate ratios and rate differences in the different strata. These tests should be interpreted with caution, since their power is low; if the result is significant at the 0.05 level, the hypothesis of homogeneity can be rejected; but "a high p-value ... does not show that the measure is uniform, it only means that heterogeneity ... was not detected by the test" (Rothman and Greenland 1998: 276); the larger the strata, the more valid the test.

The program also provides two *measures of heterogeneity*, *H* and *I-squared*, with their approximate 95% intervals, for the rate ratios and rate differences. An *H* value of less than 1.2 suggests absence of noteworthy heterogeneity, whereas a value exceeding 1.5 suggests its presence, even if the heterogeneity test is not significant. *I-squared* expresses the proportion of variation that can be attributed to heterogeneity (in a meta-analysis, to interstudy variation) rather than to sampling error.

Estimates of the supposed common underlying value of the rate ratio and rate difference (see below) are of questionable value if the findings in the various strata are very disparate. If the results are not uniform, explorations of possible causes - e.g. associations with study design or quality or with the sizes or other characteristics of the samples - may be revealing

The uniformity or heterogeneity of the measures in the different strata can be appraised not only by these tests and measures, but by plotting the values and their confidence intervals graphically, and comparing them.

Test for trend

If three or more strata are entered, a chi-square test is performed for trend of the rate ratios. This may be useful if the strata fall into a natural order, and are entered in that order.

Rate ratio

For each comparison, the program displays the ratio of the rate in group A to the rate in group B, and its 90%, 95% and 99% confidence intervals, computed by exact (Fisher's and mid-P), Cornfield's and Breslow's methods. This ratio is likely to be of interest in epidemiological studies of causal associations.

Difference between rates

For each comparison, the program displays the difference between the rates (the rate in group A minus the rate in group B), with its standard error and 90%, 95% and 99% confidence intervals. . This difference is likely to be of interest in studies of the magnitude and economic aspects of public health problems.

Overall rate ratio and overall rate difference

For a *series of table*s ("all strata"), the program computes estimators of the overall rate ratio and the overall rate difference, with their 90%, 95% and 99% confidence intervals. The rate ratio is likely to be of interest in epidemiological studies of causal associations, and the difference between rates in studies of the magnitude and economic aspects of public health problems.

The *overall rate ratio* is computed by maximum-likelihood, Mantel-Haenszel and DerSimonian-Laird methods, and its confidence intervals by exact, Mantel-Haenszel, Cornfield, Breslow and DerSimonian-Laird methods. The Cornfield estimates are identical with the intervals (not displayed) computed by the noniterative Poisson-score procedure described by Graham et al. (2003). The *overall rate difference* and its confidence intervals are estimated by precision-based, Mantel-Haenszel and DerSimonian-Laird procedures, and exact intervals are also computed.

The estimates of the supposed common underlying values, and their confidence intervals, should be treated with caution if the findings in the various strata are very disparate. The heterogeneity tests have a low power, and are unreliable with small frequencies (Rothman 1986: 223).

The estimates based on the Mantel-Haenszel procedure are appropriate even when there are zero or small frequencies. The precision-based estimates are weighted means of the stratum-specific values, using the reciprocals of their variances as weights. The DerSimonian-Laird estimate uses a random-effects model; it takes account of the variation between strata, resulting in wider confidence intervals and a more conservative significance test; the procedure may be inappropriate if sample sizes are very small.

The *DerSimonian-Laird* estimator is based on a *random-effects* model, which assumes that the strata provide estimates of randomly differing effects. The variation between strata is taken into account, resulting in wider confidence intervals and a more conservative significance test. The random-effects model gives more weight to small studies, and may be inappropriate if sample sizes are very small. Some investigators use it when unexplained heterogeneity is present (Fleiss and Gross 1991, Petitti 1994, Whitehead and Whitehead 1991). "In essence," say Rothman and Greenland (1198: 668), "a random-effects model exchanges a doubtful homogeneity assumption for a fictitious random distribution of effects . The advantage ... is that the standard errors and confidence limits ... will more accurately reflect uncertainty about unaccounted-for sources of variation". The Dersimonian-Laird estimator is not displayed if this procedure does not increase the variance of the ratio of proportions. A chi-square test is reported, appraising whether the estimator differs from zero.

Measures of the impact of exposure

Measures of the impact of exposure (attributable, prevented, and preventable fractions) are computed, on the assumption that one group or sample (A) is exposed to a risk or protective factor, and the other (B) is not. For a risk factor, the program computes the *attributable fractions in the*

exposed and in the population. For a protective factor, it computes the *prevented fractions in the exposed and in the population*, and *the preventable fraction in the population*. The attributable fraction is the proportion of the rate that can (under certain assumptions) be attributed to exposure to the factor, and the prevented fraction is the proportion of the hypothetical rate (in the absence of exposure) that has been prevented by exposure.

By default, the computation of the impact in the population assumes that the two groups are together representative of the population. Optionally (if this assumption is incorrect), the proportion of the population exposed to the risk or protective factor can be entered at the keyboard, as a percentage. This value is then used in the analysis, except in the overall analyses of stratified data, when the combined groups (in all strata) are assumed to represent the population.

The method used for stratified data is appropriate for sparse data as well as for samples with large numbers. The assumption is made that the risk ratio is uniform across the strata.

Measures of impact should be interpreted with caution (*see* Rockhill *et al.* 1998). They assume that exposure has a causal effect, and that confounding and other biases are absent. The preventable fraction is appropriate only if exposure is amenable to change.

Number needed to avoid one case

For use in studies in which the numerators of the rates are numbers of *cases* of a disease or other condition, or a disease complication, or death, the program reports the number of person-time units needed in the group with a lower rate, in order to avoid a single case.

In a clinical trial where the rate is lower in the treated group than in the control group, this is the *number needed to treat,* or the *number needed to treat (benefit)* or *NNTB* (Altman 1998), i.e. the number of patients who must be treated in order to prevent one event (Sinclair and Bracken 1994, Feinstein 1995). In a clinical trial that shows that a treatment has undesired effects (i.e., there is a lower rate in the control group), the number needed in the control group to avoid one case is also the *number needed to harm* or *NNTH* (the number of patients needed to be treated to produce one episode of harm) (Sackett *et al.* 1997: 149; Bjerre and LeLorier 2000).

In an observational study of exposure to a supposed causal factor, if the rate is lower in the exposed group the number needed is the number who need to be exposed in order to avoid one case; whereas if the rate is lower in the unexposed group it is the number whose exposure must be prevented in order to prevent one event (assuming that the findings reflect a cause-effect relationship and that the causal factor and its effect are modifiable).

The program displays 95% confidence limits for the number needed; these limits are the reciprocals of the confidence limits for the rate difference. If one of the latter confidence limits is negative, one of the confidence limits of the number needed will also be negative. For example, if group B has a lower rate and the rate difference is 10 per 100 (i.e. 0.1), with a confidence interval of - 5 to 25 per 100 (spanning zero), the number needed in group B to avoid one case is 10 (the reciprocal of 0.1), with a confidence interval of 4 to -20, an interval that includes infinity (the reciprocal of zero). Since a negative number needed to avoid a case is equivalent, if the sign in reversed, to the "number needed to harm", and this is the same as the number needed in the *other* group to avoid a case, the program reports this confidence interval as:

> '4 to infinity in B, then from infinity down to 20 in A'

in the hope that this will avoid and not further confound confusion .(see Altman 1998). This formulation indicates a continuous interval, first from 4 to infinity in the group with the lower rate, and then extending down from infinity to 20 in the *other* group.

The program also provides a formula for estimating the number needed to avoid one case in a different group or population, using the rate ratio observed in the study sample, on the assumption (not necessarily valid) that this rate ratio of rates remains applicable.. The number is the number of person-time units needed, i.e. the number of persons needed for 1 time unit in the group with the lower rate.

Fail-safe N

Estimates of the fail-safe N are provided for use in *meta-analyses*, as rough guides to the possible importance of the "file drawer problem", i.e. the exclusion of studies that were not published or were not found for other reasons.

The program computes the numbers of new "null" studies (those with an odds ratio or risk ratio of 1) that will suffice to bring the overall rate ratio to a negligible level (0.8, 0.9, 1.1 or 1.2). No account is taken of P-values or the size of the null studies.

Fail-safe N estimates based on the P-values in the various studies are provided by module I of this program.

Tests for a skewed funnel plot

Two tests for a skewed funnel plot are performed, for use in meta-analysis. The tests, which examine the association between the sizes of the effects in the component studies and their precision, are the *regression asymmetry test* and the *adjusted rank correlation test.*

In each test, a low P value suggests possible *publication bias*, although the "small-study effect" (Sterne *et al.* 2000) that it indicates may have some other cause, such as the use of higher-risk subjects in smaller studies, resulting in an association between sample size and the effect under consideration.

Both tests have a low power if they are based on few component studies. The regression asymmetry test is generally more powerful (Egger *et al.* 1997; Sterne *et al.* 2000). A critical P level of at least 0.1 should be used for both tests.

Comparison of two numbers of events

The program can also compare two numbers of events, treated as Poisson variates. It might be used to test the equality of numbers of deaths or other relatively rare events occurring in the same population in two equal time periods. For this purpose, only the two numbers are entered (in the "Numerator" boxes). Approximate confidence intervals are computed for the ratio of the two numbers.

METHODS

Exact probabilities

Formulae for the exact binomial probabilities (Fisher and mid-P*) in individual comparisons* are provided by Rothman and Boice (1982: 27-28). The program uses source code from XLIM (by A Ray Simons: version SP2.5); this is based on an F-distribution algorithm, supplemented by bisection and *regula falsi* root-solvers when there is a marked imbalance between the two numerators and the sample is large. If the bisection solver (which is relatively slow) is required and the sum of the numerators is 3,000 or less, a fast algorithm from David O. Martin's public-domain EXACTBB program (Martin and Austin 1991, 1996) is used instead.

The computation of exact probabilities *in stratified data* is sped up by the employment of an efficient algorithm for calculating the coefficients of the conditional distribution (Martin and Austin 1991, 1996), using code from David O. Martin's public-domain EXACTBB program.

Mantel-Haenszel test

The modified Mantel-Haenszel test of association is one adapted for use in cohort analyses (Breslow 1984; Rothman and Boice (1982: 11). It is performed without a continuity correction.

Heterogeneity tests and measures

Heterogeneity tests for rate ratios are now based on comparisons with the Mantel-Haenszel estimator, and the heterogeneity tests for rate differences are now based on comparisons with the Mantel-Haenszel estimator, the maximum likelihood estimator of the uniform rate ratio and the precision-based estimator of the rate difference. The tests use the Wald statistic (Rothman and Greenland 1998: 275-277).

The *measures of heterogeneity*, *H* and *I-squared*, are described by Higgins and Thompson (2002). *H* is computed by Higgins and Thompson's formula 6, and increased to 1, indicating absence of heterogeneity, if it less than 1. A test-based interval is computed by Method III. *I-squared* and its 95% interval are computed from *H*, by formula 10.

Test for trend

The test for trend of rate ratios uses formula 8 of Breslow (1984).

Rate ratio

Confidence intervals for the rate ratio are estimated by the Cornfield method (Cornfield 1956) and Breslow's method (Breslow 1984; see Rothman 1986, formula 11-16). Exact binomial intervals are also estimated, unless there are more than 20 strata.

Rate difference

Confidence intervals for the rate difference are based on formula 11-15 in Rothman (1986). Exact binomial intervals are also estimated, unless there are more than 20 strata.

Overall rate ratio and overall rate difference

The computation of exact confidence intervals for the *overall rate* is sped up by the employment of an efficient algorithm for calculating the coefficients of the conditional distribution (Martin and Austin 1991, 1996), using code from David O. Martin's public-domain EXACTBB program. The Mantel-Haenszel estimate of the overall rate ratio is based on formula 12.27 of Rothman (1986). Its confidence intervals are based on the variance computed by the method of Greenland and Robins (1985): formula 12-50 of Rothman (1986). The maximum-likelihood estimate is obtained by an iterative procedure (Rothman 1986: formula 12-17) starting with the Mantel-Haenszel estimator. Confidence intervals (Breslow's method) are based on formula 12-49 of Rothman (1986). The Cornfield estimates of confidence intervals are based on Miettinen and Nurminen's extension of Cornfield's method (Cornfield 1956; Miettinen and Nurminen 1985). The Cornfield estimates are computed without a continuity correction, as recommended by Guess *et al.* (1987) on the basis of limited simulation studies.

The Mantel-Haenszel estimates of the *overall rate difference* and its confidence intervals are now computed by the method described by Rothman and Greenland (1998: pp 269-270), which is appropriate even if the data are sparsely distributed. The precision-based estimates are based on formulae 12-1, 12-2 and 12-3 of Rothman (1986); they are appropriate only if the number in each stratum is large.

In the random-effects DerSimonian-Laird procedure (DerSimonian and Laird 1986) for the rate ratio, the log rate ratio for each stratum, and its standard error, are computed after changing any zero rate to

$$0.0001 \,/\, Base$$

(where *Bas*e = 1,000 or whatever other base is used for the rates). The Q statistic, which plays a central role in the analysis, is based on the data for separate strata and the Mantel-Haenszel estimator of the rate ratio or rate difference. If Q + 1 is less than the number of strata the random-effects and fixed-effect models yield identical results.

Measures of the impact of exposure

Attributable and prevented fractions in the exposed are calculated from the rate ratio (R); for stratified data, the Mantel-Haenszel rate ratio is used. The formulae are

$$(R - 1) \;/\; R$$

for the attributable fraction, and

$$1 - R$$

for the prevented fraction. Confidence intervals are estimated by replacing R in these formulae with its upper and lower confidence limits.

If the data are *not stratified*, or refer to a single stratum, *the attributable, prevented and preventable fractions in the population* are calculated from the rate ratio (R) and the proportion of cases exposed to the risk or protective factor in the population (E). By default, E is computed from the numerators entered for the two groups, on the assumption that together they represent the cases in the population (or population stratum). Optionally (if this assumption is incorrect), E can be entered at the keyboard, as a percentage. The formula for the population attributable fraction (AFp) is

$$E(R - 1) / R,$$

and (if AFp is negative) the population prevented fraction (PFp) is computed as

$$1 - 1 / (1 - AFp).$$

For a risk factor, the preventable fraction (if appropriate) is the same as the attributable fraction; for a protective factor, it is calculated in the same way as the attributable fraction, after relabelling the exposed as "unexposed" and the unexposed as "exposed". Confidence intervals are estimated by the same formulae, but replacing R with its lower and upper confidence limits (Daly 1998). These intervals should be regarded as approximate, since uncertainty of the exposure proportion E is not taken into account (Greenland 1999); E is regarded as a fixed value.

If stratified data are entered, attributable, prevented and preventable fractions in the total population are computed from the Mantel-Haenszel rate ratio and the proportion of cases (i.e., individuals enumerated in the numerators of the exposed and unexposed groups) exposed to the risk or protective factor. These proportions are computed by combining the numbers entered for the two groups in each stratum, without reference to any values entered for E (see above), on the assumption that the combined groups represent the population. Formula 16-24 of Rothman and Greenland (1998, p. 296) is used for the attributable fraction in the population (AF_p); if its value is negative, it is converted to the corresponding preventive fraction (see conversion equation, below). For the preventable fraction, the same formula is used as for the attributable fraction, after relabelling the exposed and unexposed. For stratified data, confidence intervals are computed from the variance of $\ln(1 - AF_p)$, using formula 16-25 of Rothman and Greenland (1998, p. 296).

If the lower confidence limit of an attributable fraction (AF) is negative, the prevented fraction (PF) equivalent to this negative value is shown; and vice versa. The conversion equations used for this purpose are:

$$PF = 1 - 1 / (1 - AF) \text{ and}$$

$$AF = 1 - 1 / (1 - PF).$$

In some instances confidence intervals are not shown because zero values interfere with their computation.

Number needed to avoid one case

The number needed is the reciprocal of the rate difference. In a stratified analysis, the reciprocal of the Mantel-Haenszel rate differences is used. The 95% confidence limits for the number needed are the reciprocals of the 95% confidence limits for the rate difference.

A formula is provided for estimating the "number needed to avoid one event" in a different sample or population, on the assumption (not necessarily valid) that the rate ratio observed in the study sample remains appropriate (Chatellier et al 1996; Smeeth et al. 1999). The formula is:

Number needed = X / P,

where X = 1 / (1 / (1 - RR) / Base)
P = rate per 1000 person-time units in group A (if RR > 1) or B (if RR < 1).
RR = the rate ratio or its reciprocal (whichever is < 1)

Fail-safe N

The fail-safe N is computed by the following formula, which is derived from Orwin's effect-size formula (Orwin 1983; Hedges and Olkin 1985, formula 9, p. 306; or Iyengar and Greenhouse 1988, formula 5):

Fail-safe N = k[abs(R) - abs(C)] / abs{C}

where k = No. of studies included in the analysis
R = log of the overall rate ratio
C = log of the chosen "negligible value" [0.8, 0.9, 1.1 or 1.2).

Tests for a skewed funnel plot

The *regression asymmetry test* (Egger *et al.* 1997) uses linear regression. It regresses the standard normal deviate (SND), defined as the effect measure divided by its standard error) against precision (the inverse of the standard error of the effect measure). In both this test and the adjusted rank correlation test, the measure of effect is the log of the rate ratio.. The regression equation is

SND = intercept + b x precision.

In the absence of bias, an intercept of zero is expected. The program reports the intercept and its 90% confidence interval, and tests its difference from zero; two-tailed P is displayed. The usual formulae for least-squares linear regression are used (e.g. Woolson and Clarke 2002: 309-311; Zar 1998: formula 17.21).

The *adjusted rank correlation test* (Begg and Mazumdar 1994) uses Kendall's rank correlation (Siegel and Castellan 1988: 245-54) to appraise the association between the sizes of the effects in the component studies (after first standardizing these effect measures) and their standard errors. In the absence of bias, a *tau* of zero is expected. Allowance is made for ties in the computation. If there are 30 or fewer component studies, tables of critical levels for one-tailed P = 0.05, 0.025, 0.01, and 0.005 (Siegel and Castellan 1988: Tables RI and RII) are used. If two-tailed P exceeds 0.01 according to these tables, and for larger samples, a Z test (making allowance for ties) is used (Armitage *et al.* 2002: 290). The two-tailed P value is displayed.

Comparison of two numbers of events

If the sum of the two values is under 60, a binomial test is used (Siegel and Castellan 1988: 38-44, formula 4.2), with a binomial probability of 0.5. If the sum is 60 or more, the program uses a chi-square goodness-of-fit test (Siegel and Castellan 1988: 45-61, formula 4.5). Approximate confidence intervals for the ratio of Poisson variates a to b are estimated by regarding $a / (a + b)$ as a binomial parameter (Ederer and Mantel 1974; Armitage *et al.* 2002: 157) and computing its confidence intervals.

E. COMPARISON OF BINARY ("YES-NO") DATA IN A STUDY USING CLUSTER SAMPLES

This module provides procedures for the analysis of clustered binary ("yes-no") data. It compares the findings observed in two independent groups in a study (a trial, case-control study, or other comparison) in which clusters of individuals or observations are used as sampling units. It might be used, for example, in a comparison of two samples whose sampling units are clusters of individuals, or in a study in which a cluster of observations is made on each member of each sample, at different times or at different body sites, e.g. in each eye or on various tooth surfaces. The clusters can vary in size, and there can be different numbers of clusters in the two groups.

Before entering the data, one category (e.g. "cases", "exposed", "died", or "carious") must be defined as "yes". For each cluster in each group, the size of the cluster (the number of observations) and the number of "yes" observations in the cluster) is then entered.

For *stratified data*, enter the table for each stratum in turn, and then click on "*All strata*" to obtain the combined results, which permit appraisal of the association while controlling for confounding effects of the stratifying variable or variables, and assessment of heterogeneity.

For a *meta-analysis*, enter the table for each study in turn (as a separate stratum), and then click on "*All strata*" to compare and combine the results in separate studies.

The program uses the **Rao-Scott and Donald-Donner procedures** to compare the groups while adjusting for intracluster correlations.

Foe *each comparison*, each of the two procedures provides a *chi-square tes*t and an *odds ratio*, with approximate 90%, 96% and 99% confidence intervals. The Rao-Scott procedure also computes the *design effect* (the *variance inflation factor*) in each group, and the *effective sample sizes* (the number of observations divided by the design factor). The Donald-Donner procedure provides estimators of the *intraclass correlation coefficients* in the two groups. An *analysis of variance* table is displayed.

For a *series of tables*, the program displays *Mantel-Haenszel chi-square tests* and *overall odds ratios* (with approximate 90%, 96% and 99% confidence intervals) computed by both methods, estimates of the *intraclass correlation coefficients* in the two groups, and estimates of the **fail-safe N**.

For comparison, **unadjusted results** (ignoring the clustering of observations) are also displayed.

Rao-Scott and Donald-Donner procedures

The Rao-Scott procedure (Rao and Scott (1992) and the Donald-Donner procedure (Donald and Donner 1987) compare the groups while adjusting for intracluster correlations. Each procedure has its advantages (Ahn and Odom-Maryon 1995, Donner *et al*. 1994). The Donald-Donner procedure may be preferred if the samples were selected by randomization, since it uses a pooled estimate of the intraclass correlation, based on the assumption that the clustering effect is the same in the two groups. The Rao-Scott procedure does not require this assumption, and estimates design effects

separately for each group. The Donald-Donner procedure tends to make a conservative adjustment to the chi-square test and to provide unduly wide confidence intervals for the odds ratio.

Fail-safe N

Estimates of the fail-safe N are provided for use in meta-analyses, as rough guides to the possible importance of the "file drawer problem", i.e. the exclusion of studies that were not published or not found for other reasons. The program computes the numbers of new "null" studies (with an odds ratio of 1) that will suffice to bring the overall Rao-Scott or Donald-Donner odds ratio (in turn) to a negligible level (0.8, 0.9, 1.1 or 1.2).

Fail-safe N estimates based on the P-values in the various studies are provided by module I of this program.

METHODS

If there are no "yes" observations in a group, computation is made possible by changing the number of "yes" observations in its largest cluster from 0 to 1. Similarly, if there are "yes" observations only, the number of "yes" observations in its largest cluster is reduced by 1. In such instances the results underestimate the difference between the two groups.

Rao-Scott and Donald-Donner procedures

The Rao-Scott procedure is described by Rao and Scott (1992) and Shoukri and Pause (1999: 65-85); *n* in Rao and Scott's equation 3 (design effect) has been changed to (*n*-1), to conform with the binomial-variance formula 24.21 of Zar (1998). The Donald-Donner procedure is described by Donald and Donner (1987) and Shoukri and Pause (1999: 65-85).

In the Donald-Dunner procedure, negative intraclass correlation coefficients are truncated to 0.

The analysis can handle up to 40 strata (with up to 200 clusters per stratum).

Fail-safe N

The fail-safe N is computed separately (*see page 29*) for each of the adjusted common odds ratios.

Unadjusted results

The usual chi-square test is performed for a single comparison (Zar 1998: formula 23.6}. The unadjusted results for stratified data are based on Hauck's variance formula and the usual Mantel-Haenszel test (Donald and Donner 1987: formulae 3 and 5).

F1. COMPARISON OF CATEGORICAL DATA (THREE OR MORE NOMINAL CATEGORIES)

This module can be used to analyse any simple 2 x *k* contingency table (derived from an observational study or from a trial) that compares two independent groups with respect to a nominal variable that has three or more categories, or a series of such tables representing the findings in different strata or in different studies. Module F2 of this program should be used if the categories fall into a natural order. Module G is preferable for comparisons of cases and controls with respect to their exposure to a risk or protective factor that has three or more levels.

The categories may be entered in any order; but if there is a reference category it should be entered first.

For *stratified data*, enter the table for each stratum in turn, and then click on "All strata" to obtain the combined results, which permit appraisal of the association while controlling for confounding effects of the stratifying variable or variables, and assessment of heterogeneity.

For a *meta-analysis*, enter the table for each study in turn (as a separate stratum), and then click on "All strata" to compare and combine the results in separate studies.

For *each table entered*, the program provides **exact probabilities** (Fisher's and mid-P), **chi-square tests of association** (with adjusted residuals, multiple comparisons with the first category, and optional partitioning), **Goodman and Kruskal's *tau***, **Theil's uncertainty coefficient *U***, **and *phi***, compares the **distribution of the categories** in the two groups, and computes **Shannon's index of the diversity of distribution** among the categories.

For a *series of tables* with the same number of categories in each, the program provides an extended **Mantel-Haenszel test for nominal categories**.

Exact probabilities

Fisher's and mid-P exact probabilities are computed. They are based on the conditional probabilities, given the marginal frequencies, of all possible constellations of values, on the assumption that the variables are independent. *Fisher's P* is the sum of the probabilities of all constellations with probabilities lower than, or as low as, the probability of the observed set of values. The *mid-P* estimate is similar, except that probabilities that are identical to the probability of the observed values are halved before they are added to the sum. A mid-P value "does not guarantee that the Type I error rate falls below a fixed value. However, it usually performs well and is less conservative than Fisher's exact test" (Agresti 1996: 43). Armitage *et al.* (2002: 120) recommend that both the Fisher P and the mid-P value should be given.

Since all possible constellations are taken into account, the probabilities are essentially two-tailed.

Computation may be slow if numbers are large or there are many categories. In such instances computation may pause, and can be optionally terminated if the interim P (which grows by accretion during the analysis) is sufficiently informative.

Chi-square tests of association

The program performs both conventional (Pearson) and log-likelihood-ratio chi-square (G2) tests. In addition, Haldane's large-table chi-square test (Maxwell 1961: 41-44) is performed if there are over 30 categories.

Pearson and log-likelihood-ratio chi-square tests generally lead to the same conclusions. When they do not, many statisticians prefer the log-likelihood-ratio test (Zar 1998: 506). Whenever Williams's criterion for preferring the log-likelihood-ratio chi-square to the Pearson chi-square is met – i.e. if any expected frequency (under the null hypothesis) is less than its difference from the observed frequency (Williams 1976) – the program displays a message to this effect.

Chi-square tests may be misleading if the expected frequencies (under the null hypothesis) are too small. Cochran (1954) recommended that fewer than one-fifth of the cells should have expected frequencies of less than 5, and none should have an expected frequency of less than 1. The program displays a warning if these conditions are not met. A warning is also shown if the mean frequency per cell is under 5, since the likelihood-ratio test may then be of low validity; the P-value tends to be too high if most expected values are less than 0.5, and too low if most expected values are between 0.5 and 5 (Agresti 1996: 194).

The validity of *Haldane's large-table chi-square test* is not affected by zero or small cell frequencies. Two alternative methods of appraisal are provided: a Z value and a suggested improvement, Bartlett's modified chi-square.

Adjusted residuals are displayed. These show which cells contribute most to the chi-square, and may be helpful in determining the sources of a significant association. The residuals have been converted to Z scores so as to indicate their statistical significance. An adjusted residual over 1.96 or under -1.96, for example, indicates significance at the $P < 0.05$ level.

Multiple comparisons are performed, based on a series of 2 x 2 tables in which each category in turn is compared with the first one. This should be kept in mind when entering the data – if there is a reference category (for example, a control group in a trial or observational study), it should be entered first. Odds ratios are displayed as well as chi-squares.

Partitioning of chi-square may help to determine just where the differences are. It is generally recommended that chi-square should be partitioned only if a significant association has been found. If the option for partitioning chi-square is selected, categories that are believed to be similar, *a priori* or after inspection of the data, must be indicated.. The program then performs likelihood-ratio chi-square tests on two new tables: (a) a table confined to (and hence comparing) these selected categories, and (b) the whole table, but with the selected categories combined (so as to ignore the variation of these categories), on the assumption that they are indeed similar. The sum of the two

chi-squares is the chi-square for the original table. Two P values are reported for each test – one for testing an *a priori* hypothesis, and one for testing a hypothesis suggested by the data.

Mantel-Haenszel test for nominal categories

This overall test of association controls for confounding effects of the stratifying variable or variables – e.g. of age and sex if the strata represent different age-sex groups. In a meta-analysis, it is an overall test, controlling for the differences between the studies.

The test is not done if the number of categories varies in different strata or if there are over 40 categories or over 40 strata.

Goodman and Kruskal's *tau*

Goodman and Kruskal's *tau*, a proportional-reduction-in-errors measure of the association between two nominal-scale variables (here, the "yes-no" variable and the other variable), expresses the extent to which knowledge of the distribution of one variable enhances the accuracy with which the other can be predicted (Blalock 1979: 307-310; Jacobson 1976: 430-434; Agresti 1990: 24-25). It varies from 0, which means that the one variable is no help in predicting the other, to 1, which means that the one variable perfectly specifies the other. *Tau* is calculated for predictions in each direction. A symmetric (nondirectional) version is also computed.

Tau is influenced by the marginal totals, and is therefore generally of use only if the data come from a total group or population of interest, or a representative sample of such a group or population. The results may be misleading if the marginal totals are determined arbitrarily, as in case-control or cohort studies in which samples of arbitrary sizes are compared.

Theil's uncertainty coefficient *U*

Theil's uncertainty coefficient *U*, like Goodman and Kruskal's *tau*, is a proportional-reduction-in-errors measure of the association for between two nominal-scale variables (here, the "yes-no" variable and the other variable) (Agresti 1990: 25; Press *et al.* 1989: 527-552). It indicates the extent to which knowing the distribution of one variable enhances accuracy in predicting the distribution of the other. The possible range is from 0 (no association) to 1 (complete dependence).

U coefficients are computed for predictions in both directions, as well as a symmetric (nondirectional) weighted average.

Like Goodman and Kruskal's *tau* (see above), *U* is influenced by the marginal totals.

Phi

Phi (Siegel and Castellan 1988: 232-235), a measure of association based on chi-square, can vary from 0 to 1. In a 2 x *k* table, it is equivalent to Cramer's *V* (Siegel and Castellan 1988: 225-232).

Like Goodman and Kruskal's *tau* (see above), *phi* is influenced by the marginal totals.

Distribution of the categories

The program displays the percentage distribution of the categories in each of the two groups. For each category, it shows the difference between the percentages observed in the two groups, and the ratio of the percentages observed in the two groups..

Shannon's index of the diversity of distribution

Shannon's index (Shannon 1948), which expresses the distribution of observations among the categories, is computed for each of the two groups, and the difference between the indices in the two groups is tested.

The Shannon index is high (close to 1) if the distribution is even, and low (close to 0) if it is uneven. Since it is affected by the number of categories, relative diversity is also displayed, expressing the index as a percentage of the maximum possible value for the given number of nonzero categories.

Especially in a small sample, Shannon's index is an underestimate of the diversity in the sampled population. The relative diversity is typically an overestimate (Zar 1998: 50-42).

The test comparing the indices is not meaningful if the sizes of the groups are determined arbitrarily, as in case-control or cohort studies in which samples of arbitrary sizes are compared.

METHODS

Exact probabilities

The computation of exact probabilities is explained by Maxwell (1961: 46-50). The probability of every possible combination of frequencies, taking the marginal frequencies as fixed, is computed in turn. If the probability is lower than the probability of the observed frequency table, it is included in the cumulative estimate, both for Fisher's P and for the mid-P estimate. If the probability of the combination is the same as the probability of the observed table, it is included in Fisher's P, and half its value is added to the mid-P estimate.

Chi-square tests of association

Formulae for chi-square are provided by most statistics textbooks, (e.g. Zar 1998: formula 23.1 for Pearson's chi-square and 22.11 for the likelihood ratio test). The computation of likelihood-ratio chi-squares when there is a zero frequency is made possible by changing the zero to 0.0000001; an appropriate message is displayed.

Haldane's large-table chi-square test is based on the exact mean and variance of chi-square. Formulae are provided by Maxwell (1961: 41-44).

*Adjusted residual*s are the discrepancies between the observed frequencies and the values expected under the null hypothesis, converted to Z scores. The procedure is described by Everitt (1977:46-48; formulae 3.6 to 3.8) and Agresti (1996: 31-32; formula 2.4.4).

The *multiple comparisons* with the first category are based on likelihood-ratio chi-square tests. Since these tests are not independent, the P-values are modified so as to make them appropriate for multiple tests (Everitt 1977: 44-46; Golbeck 1994). A Bonferroni adjustment is applied, by multiplying P by the number of tests performed, i.e. the number of categories minus 1 (Golbeck 1994)

Chi-square partitioning is described by (among others) Everitt (1977: 41-44), Agresti (1990: 50-54 and 1996: 32-33), and Siegel and Castellan (1988: 118-123). The P-value for testing an *a priori* hypothesis is based on the degrees of freedom in the table tested. The alternative (conservative) P-values provided for testing *a posteriori* hypotheses are

based on the degrees of freedom in the total (original) table, so that "it is in no way necessary to decide a priori (i.e. before seeing the data), what combinations are to be tested and one may be guided by the data themselves in deciding what to test" (Gabriel 1966).

Mantel-Haenszel test for nominal categories

The extended (generalized) Mantel-Haenszel test for nominal categories is described by Agresti (1990: 234-5). Matrix calculations are required.

Goodman and Kruskal's *tau*

Goodman and Kruskal's *tau* (Agresti 1990: 24) is computed twice, with fixed marginal totals for the row variable and the column variable in turn; a symmetric version is also computed. For detailed formulae, see Jacobson 1976 or Gahlinger and Abramson 1995: 47.

Theil's uncertainty coefficient *U*

Theil's uncertainty coefficient is computed by formulae 12.6.15, 13.6.16, and 13.6.16 of Press *et al.* (1989: 530), using an adaptation of the cstab2 procedure (Press *et al.* 1989: 530-531).

Phi

The formula for *phi* is

$$phi = \sqrt{(\text{chi-square} / N)}$$

where N = total number of observations.

Shannon's index of the diversity of distribution

Formulae for Shannon's index and relative diversity are provided by Zar (1998: formulae 4.18 to 4.20). The test for the difference between two diversity indices (Hutcheson 1970) is described by Zar (1998: 156-158). Logs to base 10 are used in the computations.

F2. COMPARISON OF CATEGORICAL DATA (THREE OR MORE ORDERED CATEGORIES)

This module can be used to analyse any simple 2 x *k* contingency table (derived from an observational study or from a trial) that compares two independent groups with respect to an ordinal variable that has three or more categories, or a series of such tables representing the findings in different strata or in different studies. Module G of this program is preferable for comparing cases and controls with respect to their exposure to a risk or protective factor with three or more levels.

The categories must be entered in the correct order, starting at either end of the scale; if there is a reference category it should be entered first. In a follow-up study that compares the changes observed in two independent groups, with categories ranging from extreme change in one direction to extreme change in the other (based on paired before-after ratings), the "no change" category must be specified.

By default, the categories are given scores of 1, 2, 3 etc., for use in some of the tests and measures. If these scores are inappropriate, they can be changed to others that better express relative magnitude or degree. Appropriate scores for age categories, for example, night be the mid-points of age ranges, and for smoking categories, they might be the median numbers of cigarettes per day

For *stratified data*, enter the table for each stratum in turn, and then click on "*All strata*" to obtain the combined results, which permit appraisal of the association while controlling for confounding effects of the stratifying variable or variables, and assessment of heterogeneity.

For a *meta-analysis*, enter the table for each study in turn (as a separate stratum), and then click on "*All strata*" to compare and combine the results in separate studies.

For *each table entered*, the program provides an **extended Mantel-Haenszel test**, a **Mann-Whitney test**, **Kolmogorov-Smirnov and Cramer-von Mises tests**, an optional **Strickland-Lu test**, **ridit analysis**, **odds ratios,** and **regression and correlation coefficients**.

For a *series of tables,* the program provides an **extended Mantel-Haenszel procedure**; a **Mann-Whitney test** (van Elteren procedure); **tests and measures of heterogeneity**; **odds ratios,** and **regression and correlation coefficients.**

Extended Mantel-Haenszel test

The extended Mantel-Haenszel test, which is based on the scores allocated to the categories (see above), is regarded as a test for trend, since it is much more capable of detecting linear and other monotonic relationships than other associations; but a significant result can also be produced by a non-monotonic association (Rothman and Greenland 1998: 314).

The test is applied both to a single table and to stratified data (provided that the number of categories is the same in each stratum). Two-tailed and (for single tables) one-tailed P-values are shown.

The test is appropriate for sparse data, but (in a single table) at least two of the cell frequencies for each of the two groups must be large.. For stratified data, at least two of the cell frequencies for each group, summed across the strata. must be large (Rothman and Greenland 1998: 315).

Mann-Whitney test

The Mann-Whitney test (the Wilcoxon-Mann-Whitney test, or Wilcoxon rank-sum test) is based on the ranks of the observations; it does not require scores for the categories.. The null hypothesis is that the ranks are similar in both groups, and the alternative hypothesis is that the observations in one group tend to have higher ranks than those in the other.

If stratified data are entered each stratum is analysed separately, and the strata are then combined for testing by the *van Elteren procedure* so as to control for possible confounding effects of the stratifying variables. Caution should be used if the strata are small, or if the results in the various strata are very different.

One-tailed and two-tailed P values are shown. The program may (if numbers are small) display, but not explain, five values (*m*, *n*, *U1*, *U2*, and *Wx*) that can be employed to obtain more exact P values (see "Methods", below).

Kolmogorov-Smirnov and Cramer-von Mises tests

The Kolmogorov-Smirnov two-sample test and the Cramer-Von Mises test are tests for general differences between the distributions in the two groups. They are sensitive to differences in central tendency, spread, and symmetry or skewness, as well as in trend.

For each test, a two-tailed P-value is displayed, indicating whether the two sets of frequencies can be supposed not to come from the same unspecified distribution. The Cramer-von Mises test is often more powerful than the Kolmogorov-Smirnov test (Sprent 1993: 128).

Strickland-Lu test

The Strickland-Lu test is performed only if a "no change" category is specified (see above). It is a test for the difference between the changes observed in two independent groups, in a follow-up study in which change, appraised by comparing paired "before" and "after" ratings for each individual, is expressed in an ordinal degree-of-change scale whose categories range from extreme change in one direction to extreme change in the other (Strickland and Lu 2003). The degree-of-change data for each group must be entered, and the category representing "no change" must be specified.

Two-tailed and one-tailed P values are computed using two alternative logit models – a proportional-odds model and an adjacent-category-odds model (which, in effect, gives the same weight to any change in a particular direction). When there is a large difference between the groups, the test may slightly overestimate significance.

Test and measures of heterogeneity

Two heterogeneity tests are performed - one for the linear components of the trends in the strata, and one for the generalized odds ratios in the strata. The former test is done only if the number of categories is the same in each stratum.

The *heterogeneity tests* have a low power, and the results should be interpreted with caution. If the result is significant at the 0.05 level, the hypothesis of homogeneity can be rejected; but "a high pvalue ... does not show that the measure is uniform, it only means that heterogeneity ... was not detected by the test" (Rothman and Greenland 1998: 276); the larger the strata, the more valid the test.

Each heterogeneity test is accompanied by two *measures of heterogeneity*. These are *H* and *I-squared*. An *H* value of less than 1.2 suggests absence of noteworthy heterogeneity, whereas a value exceeding 1.5 suggests its presence, even if the heterogeneity test is not significant. *I-squared* expresses the proportion of variation that can be attributed to heterogeneity (in a meta-analysis, to interstudy variation) rather than to sampling error.

Estimates of the supposed common underlying measure, such as the generalized odds ratio, are of questionable value if the findings in the various strata are very disparate. If the results are not uniform, explorations of possible causes – e.g. associations with study design or quality or with the sizes or other characteristics of the samples – may be revealing.

The uniformity or heterogeneity of the measures in the different strata can be appraised not only by these tests and measures, but by plotting the values and their confidence intervals graphically.

Ridit analysis

Ridit analysis (Fleiss et al. 1003: 198-205) is used to estimate the probability that a randomly selected subject in group B will have a more extreme value (i.e., will fall into a category nearer to the last-entered category) than a randomly selected subject in group A. The standard error of this probability is reported, but significance is not tested, since ridit analysis is a version of the Mann-Whitney test (Selvin 1996: 178). The odds in favour of a more extreme value in group B is reported.

Ridit analysis is based on the ranks of the observations, and does not use the category scores.

Odds and odds ratios

As indicators of the difference between the two groups, the program provides two odds ratios - a cumulative odds ratio and a generalized odds ratio - that summarize the difference between the distributions in the two groups.

The *cumulative odds ratio* is based on a proportional odds model, which assumes that when the 2 x *k* table is converted to a 2 x 2 table by collapsing categories the odds ratio is the same whatever cut-point is used; 95% confidence intervals are displayed. For stratified data, a common cumulative odds ratio is computed by a Mantel-Haenszel-type procedure, appropriate even when data are very sparse. The common odds ratio is a weighted average of the stratum-specific cumulative odds ratios, and provides a useful summary of the association even if the common cumulative odds ratio assumption does not hold (provided that heterogeneity is not too severe and the directions of the odds ratios are the same). Its confidence intervals may be inaccurate if the true odds ratios are heterogeneous within or between strata.

The *generalized odds ratio* (*GOR*) - which is not based on the proportional odds model - summarizes the difference between the distributions in the two groups. It expresses the odds in favour of a higher score in one specified group (A or B) than in the other; it is the ratio of two probabilities - the

probability that a randomly selected observation from one specified group (A or B) has a higher score than a randomly selected observation from the other group, and the probability that it has a lower score. Two *GOR* values are displayed - one for a higher score in A, and one for a higher score in B, with their 90%, 95%, and 99% confidence intervals. The *GOR* is the *alpha* measure proposed by Agresti 1980; it is also described by Lui (2004: 119-122). If stratified data are entered, the assumed common values of the *GOR* are displayed (with their confidence intervals); these are weighted averages of the stratum-specific *GOR* values, and are of questionable value is there is marked heterogeneity.

The program also displays odds ratios contrasting each category with the first (reference) category. For stratified data these are Mantel-Haenszel overall odds ratios.

Regression and correlation coefficients

Regression and correlation coefficients are computed, based on the *scores* of the categories (and scores of 1 and 2 for the two groups). They are not appropriate in all study designs. The regression of the score on the group is the mean difference in scores between the two groups..

In addition, two coefficients based on *ranks* are computed: *Kendall's rank correlation coefficient,* and *gamma.*

METHODS

Extended Mantel-Haenszel test

The test is described by Mantel (1963) and explained by (among others) Rothman (1986: 346-348), Breslow and Day (1980: 146-154), and Rothman and Greenland (1998 : 214-315). The one-sided test uses the square root of chi-square (Agresti 1996: 35).

Mann-Whitney test

The test is described by (among others) Siegel and Castellan (1988: 128-137) and Zar (1998: 146-155). Allowance is made for ties, each of the tied observations being given the mean value of the relevant ranks.

If either sample contains over 10 observations, one- and two-tailed Z tests based on a normal approximation are used; the program uses the improved normal approximation procedure of Hodges *et al.* (Zar 1998. formulae 8.58–8.61).

If there are 10 or fewer observations in each sample, a table of critical values of the *U* distribution is used for P < .1, <.05, < .025, < .01, <.005, and <.0005 for one-tailed tests, and these probabilities are doubled for two-tailed tests (Zar 1998: App89: Table B.11). If the size of the smaller group does not exceed 20, the program displays values that may be employed to determine P more exactly, using tables such as those in Zar (1998: App89) and Siegel and Castellan (1988: 339). These values are *m* and n (the sizes of the smaller and larger samples respectively), two values of *U* (*U1* and *U2*), and *Wx* (the sum of the ranks for the smaller sample). When using the tables in Zar (1998) for a one-tailed test, *U2* should be used if the alternative to the null hypothesis is that the values in A are higher than those in B, and *U1* if the hypothesis is in the opposite direction; the higher of U1 and U2 should be used for a two-tailed test.

If stratified data are entered, the van Elteren procedure (van Elteren 1960) is used.

Kolmogorov-Smirnov and Cramer-von Mises tests

The *Kolmogorov-Smirnov two-sample test* is described by Siegel and Castellan (1988: 144-151). The test is performed if both sample sizes exceed 2. The test statistic D is computed by formula 6.18 in Siegel and Castellan (1988: 145). If sample sizes are small, exact P-values are reported as P<0.001, <0.01, <0.05, <0.1 or >0.1. For P<0.001, if both sample sizes are 20 or less, these are based on Hollander and Wolfe (1999: 606-630: Table A.10); for the other critical values, if both sample sizes are 25 or less, the source is Siegel and Castellan (1988: 350-351: Table L.II). For larger samples,

approximate P-values are reported as <0.001, <0.005, <0.01, <0.025, <0.05, <0.1 or >0.1, using the formulae in Siegel and Castellan (1988: 352: Table L.III).

The Cramer-von Mises test is described by Sprent (1993: 127-128). It is performed if both sample sizes exceed 3, and if the total number in each category is under 10,000. If the sum of the two sample sizes is less than 18, exact P-values are reported as P<0.001, <0.01, <0.05, <0.1 or >0.1 (source: Burr 1964). In other instances the approximate values are calculated by an adaptation of a Fortran procedure from David Baird's library of goodness of fit statistics (EMPCDF.SRC). Ties are handled as follows: if the frequencies (in groups A and B) in a category are 3 and 7, the cumulative relative frequencies are compared several times - first after entry of each of the 3 pairs of observations (one in each sample), and then after entry of each of the 4 remaining observations.

Strickland-Lu test

The Strickland-Lu test is described by Strickland and Lu (2003). To obtain the test statistic (an approximate normal test statistic), an odds ratio is estimated for each group (formula 1), and the log of the ratio of the two odds ratios is divided by the square root of its variance (formula 2). For the purpose of this computation, 0.5 is first added to zero cells (categories with a zero frequency for both groups are not taken into account).

Test and measures of heterogeneity

The *heterogeneity chi-square* is the sum of the Mantel-Haenszel chi-squares in each stratum minus the overall Mantel-Haenszel chi-square (Rothman and Boice 1982: 9).

The *measures of heterogeneity*, H and *I-squared*, are described by Higgins and Thompson (2002). H is computed by Higgins and Thompson's formula 6, and increased to 1, indicating absence of heterogeneity, if it less than 1. A test-based interval is computed by Method III. *I-squared* and its 95% interval are computed from H, by formula 10.

Ridit analysis

The method is described by Fleiss *et al*. (2003: 200-202).

Odds ratios

Formulae for the *cumulative odds ratio* and its variance are provided by Liu and Agresti (1996: formulae 2 and 3). The program uses an adaptation of Fortran code made available to us by Liu and Agresti.

The *generalized odds ratio* (*GOR*) is computed by the formula provided by Lui (2004: 120), and its 95% confidence interval by the logarithmic-transformation method of formula 6.3 (Lui 2004: 121). For stratified data, the assumed common value of the *GOR* is the exponent of a weighted average of the logs of the *GOR* values in the strata, and its 95% confidence interval is computed from the estimated variance of this weighted average (Agresti 1980 : 63).

In stratified data, the *odds ratios contrasting each category with the first category* are Mantel-Haenszel odds ratios for 2 x 2 tables (Robins *et al*. 1986, Rothman 1986: 217-220); they are not computed if there are over 30 categories.

Regression and correlation coefficients

The regression and correlation analysis based on scores uses methods described by Mantel (1963).

Formulae for *Kendall's rank correlation coefficient* and *gamma* are provided by (among others) Siegel and Castellan (1988: 245, 291). Ties are allowed for in the calculation of Kendall's rank correlation coefficient (Siegel and Castellan 1988: 249: formula 9.10). The pooled values are weighted means of the coefficients in the various strata, using the number of observations in the stratum as the weight.

G. COMPARISON OF THREE OR MORE EXPOSURE LEVELS (IN A CASE-CONTROL STUDY)

This module is appropriate for the analysis of a comparison of cases and unmatched controls with respect to their exposure to a supposed risk or protective factor that has three or more ordered levels.. It might be used in the evaluation of a therapeutic or preventive procedure, by comparing the proportions who had been exposed to the procedure. It provides the same procedures as module F2 of this program, with the exception of a Strickland-Lu test, and with the optional addition of measures of the impact of exposure (attributable or prevented fractions),..

A single group of cases can be compared with a single group of controls, or a series of case-control comparisons can be made in different strata or in different studies

The exposure categories must be entered in the correct order, starting at either end of the scale; if there is a reference category (e.g. a nonexposed category) it should be entered first.

By default, the categories are given scores of 1, 2, 3 etc., for use in trend tests. If these scores are inappropriate, they can be changed to others that better express relative magnitude or degree. Appropriate scores for age categories, for example, night be the mid-points of age ranges, and for smoking categories, they might be the median numbers of cigarettes per day

For *stratified data*, enter the table for each stratum in turn, and then click on "*All strata*" to obtain the combined results, which permit appraisal of the association while controlling for confounding effects of the stratifying variable or variables, and assessment of heterogeneity.

For a *meta-analysis*, enter the table for each study in turn (as a separate stratum), and then click on "*All strata*" to compare and combine the results in separate studies.

For *each table entered*, the program provides an **extended Mantel-Haenszel test**, a **Mann-Whitney test**, **Kolmogorov-Smirnov and Cramer-von Mises tests**, **odds and odds ratios, regression and correlation coefficients,** and **measures of the impact of exposure** (attributable or prevented fractions)**.**.

For a *series of tables,* the program provides an **extended Mantel-Haenszel procedure**; a **Mann-Whitney test** (van Elteren procedure); a **test and measures of heterogeneity**; **odds and odds ratios, regression and correlation coefficients,** and **measures of the impact of exposure** (attributable or prevented fractions)**.**.

Extended Mantel-Haenszel test

The extended Mantel-Haenszel test, which is based on the scores allocated to the categories (see above), is regarded as a test for trend, since it is much more capable of detecting linear and other monotonic relationships than other associations; but a significant result can also be produced by a non-monotonic association (Rothman and Greenland 1998: 314).

The test is applied both to a single table and to stratified data (provided that the number of categories is the same in each stratum). Two-tailed and (for single tables) one-tailed P-values are shown.

The test is appropriate for sparse data, but (in a single table) at least two of the cell frequencies for each of the two groups must be large. For stratified data, at least two of the cell frequencies for each group, summed across the strata must be large (Rothman and Greenland 1998: 315).

Mann-Whitney test

The Mann-Whitney test (the Wilcoxon-Mann-Whitney test, or Wilcoxon rank-sum test) is based on the ranks of the observations; it does not require scores for the categories. The null hypothesis is that the ranks are similar in both groups, and the alternative hypothesis is that the observations in one group tend to have higher ranks than those in the other.

If stratified data are entered each stratum is analysed separately, and the strata are then combined for testing by the *van Elteren procedure* so as to control for possible confounding effects of the stratifying variables. Caution should be used if the strata are small, or if the results in the various strata are very different.

One-tailed and two-tailed P values are shown. The program may (if numbers are small) display, but not explain, five values(*m*, *n*, *U1*, *U2*, and *Wx*) that can be employed to obtain more exact P values (*see page* 49).

Kolmogorov-Smirnov and Cramer-von Mises tests

The Kolmogorov-Smirnov two-sample test and the Cramer-Von Mises test are tests for general differences between the distributions in the two groups. They are sensitive to differences in central tendency, spread, and symmetry or skewness, as well as in trend.

For each test, a two-tailed P-value is displayed, indicating whether the two sets of frequencies can be supposed not to come from the same unspecified distribution. The Cramer-von Mises test is often more powerful than the Kolmogorov-Smirnov test (Sprent 1993: 128).

Test and measures of heterogeneity

For stratified data with the same number of categories in each stratum, the program provides a heterogeneity test for the uniformity of the trends in the various strata, and two measures of heterogeneity, with their approximate 95% confidence intervals.

The *heterogeneity test* has a low power, and should be interpreted with caution. If the result is significant at the 0.05 level, the hypothesis of homogeneity can be rejected; but "a high p-value ... does not show that the measure is uniform, it only means that heterogeneity ... was not detected by the test" (Rothman and Greenland 1998: 276); the larger the strata, the more valid the test.

The *measures of heterogeneity are H* and *I-squared* An *H* value of less than 1.2 suggests absence of noteworthy heterogeneity, whereas a value exceeding 1.5 suggests its presence, even if the heterogeneity test is not significant. *I-squared* expresses the proportion of variation that can be attributed to heterogeneity (in a meta-analysis, to interstudy variation) rather than to sampling error.

Estimates of the supposed common underlying values of measures of association, such as the Mantel-Haenszel odds ratio, are of questionable value if the findings in the various strata are very disparate. If the results are not uniform, explorations of possible causes – e.g. associations with study design or quality or with the sizes or other characteristics of the samples – may be revealing.

The uniformity or heterogeneity of the measures in the different strata can be appraised not only by these tests and measures, but by plotting the values and their confidence intervals graphically, and comparing them.

Odds and odds ratios

As indicators of the difference between the two groups, the program provides estimates of the odds in favour of a higher score in one group, and the cumulative odds ratio.

The odds in favour of a higher score in one group are the odds in favour of finding that a randomly chosen observation in one group is in a category with a higher score than a randomly chosen observation in the other group. These odds are displayed for each group, with its approximate 95% confidence interval.

The cumulative odds ratio is based on a proportional odds model, which assumes that when the 2 x *k* table is converted to a 2 x 2 table by collapsing categories the odds ratio is the same whatever cut-point is used; 95% confidence intervals are displayed. For stratified data, a common cumulative odds ratio is computed by a Mantel-Haenszel-type procedure, appropriate even when data are very sparse. The common odds ratio is a weighted average of the stratum-specific cumulative odds ratios, and provides a useful summary of the association even if the common cumulative odds ratio assumption does not hold (provided that heterogeneity is not too severe and the directions of the odds ratios are the same). Its confidence intervals may be inaccurate if the true odds ratios are heterogeneous within or between strata.

The program also displays odds ratios (with their 95% confidence intervals) contrasting each category with the first (reference) category. For stratified data, these are Mantel-Haenszel overall odds ratios.

Regression and correlation coefficients

Regression and correlation coefficients are computed, based on the *scores* of the categories (and scores of 1 and 2 for the two groups). They are not appropriate in all study designs. The regression of the score on the group is the mean difference in scores between the two groups..

In addition, two coefficients based on *ranks* are computed: *Kendall's rank correlation coefficient* and *gamma.*

Measures of the impact of exposure

Optionally, the program computes the attributable or prevented fraction in the population, on the assumption that the exposure is to a factor that causes or protects against the disease or other outcome represented by the cases. If exposure is to a risk factor, the program computes the attributable fraction in the population; and if exposure is to a protective factor, it computes the prevented fraction in the population. The attributable fraction can be regarded as the proportion of

the disease rate that can be attributed to exposure, and the prevented fraction as the proportion of the hypothetical rate (in the absence of exposure) that has been prevented by exposure.

In each single comparison of cases and controls, the calculation is based solely on the sample data. The computed fraction is an approximation, based on the assumptions that the prevalence of exposure among the controls is representative of that in the population, and that the odds ratio in the sample is a good estimate of the risk ratio in the population.

But if stratified data are entered, options are provided for enhancing the accuracy of the overall values by entering, for each stratum, the probability of the disease in the stratum and a population figure or weight expressing the relative size of the stratum. The latter entries serve to neutralize the effects of variation in the sampling fractions used in the various strata, and can also be used to standardize the attributable or prevented fractions in different populations and permit comparisons while holding constant the distribution of the stratifying variable. The weighting factors used for the latter purpose might be the sizes of the strata in a standard population.

The exposure categories must be entered in their correct order, with the control (nonexposed) category first. Attributable or prevented fractions, with their 90%, 95%, and 99% confidence intervals are computed for all exposure levels except the first, separately and together; the contrasts are with the control category.. Confidence intervals are estimated by three different methods, based respectively on the maximum likelihood estimate of the attributable fraction and on log and logit transformations. On the basis of analyses of computer-simulated case-control studies, Whittemore (1983) recommends the use of logit intervals for attributable fractions in the range 21-79% and maximum-likelihood intervals for estimates outside this range, and says that the simulations suggest no advantage to using the log intervals.

All results - the attributable or prevented fraction, standard errors, and confidence intervals - are shown as percentages.

If the data are stratified, and the same number of categories were entered in each stratum, a weighted average of the values in the separate strata is calculated. If the data are stratified according to the categories of a suspected confounding variable, this controls for effects connected with this confounder. The weights may be based solely on the sample data, or may take account of the extra information entered (see above). If the disease rate in each stratum of the population and the size of each stratum in the population have been entered, their product , which is the number of cases in the population stratum, is used as the weight. A simulation study has shown that "case load weighting" of this sort is the best method of adjustment in all practical situations with relatively large sample sizes; in small samples, however, there may be a severe downward bias (Gefeller 1992). Crude attributable fractions, based on simple pooling of the case-control data in the various strata, are also displayed.

When a prevented fraction is displayed, the equivalent (negative) attributable fraction (see conversion formula in “Methods”) is also reported.

METHODS

Extended Mantel-Haenszel test

see page 48.

Mann-Whitney test

see page 48.

Kolmogorov-Smirnov and Cramer-von Mises tests

see page 48.

Test and measures of heterogeneity

see page 49.

Odds and odds ratios

see page 49.

Regression and correlation coefficients

see page 49.

Measures of the impact of exposure

The program computes attributable fractions. If the fraction is negative – that is, if the factor is a protective rather than a risk factor – the attributable fraction AF is converted to the prevented fraction (PF) by the formula

$$PF = 1 - 1 / (1 - AF)$$

In each case-control comparison, formulae 7.65 and 7.68 of Schlesselman (1982) are used; the disease rate in the population is not taken into account. Dichotomous contrasts use formula 5 of Whittemore (1983), and formula 4-11 of Kahn and Sempos (1989) is used for standard errors..

For *stratified data*, a weighted average of the attributable fractions in individual strata is computed (Whittemore 1983: formulae A1, A3), using weights normalized so that their sum = 1. The standard error is

$$\sqrt{}(SE_i . w_i)^2$$

where SE_i and w_i are the specific standard errors and weights in individual strata.

The weight allocated to a stratum is based on the number of cases in the sample stratum, or (if entered) the disease rate in the population stratum, or (if entered) the size of the population stratum, or the product of the stratum's disease rate and size (if both were entered).

The three methods of computing *confidence intervals* are explained by Whittemore (1983). An approximate chi-square test for the significance of the attributable fraction is provided by Schlesselman (1982: 223).

The *crude attributable fraction*, based on simple pooling of the case-control data in the various strata, uses formulae 7.58 and 7.65 of Schlesselman (1982).

The program can cope with up to 60 exposure levels and up to 60 strata.

H1. COMPARISON OF NUMERICAL OBSERVATIONS (NORMAL DISTRIBUTION NOT ASSUMED)

This module provides nonparametric procedures for comparing two independent sets of numerical observations (ordinal-scale or interval-scale), without assuming a normal distribution. It may be used for the analysis of trials as well as observational studies. Module H2 of this program should be used if a normal distribution can be assumed, and module H3 for comparing survival data.

Some of the procedures are based on the actual values entered, and are appropriate for interval-scale variables only. Most of the procedures are based on the ranks of the observations, and are appropriate for both ordinal-scale variables and interval-scale ones (treated as ordinal).

The findings in two groups can be compared, or a series of comparisons can be made in different strata or in different studies. For each comparison, individual values can be entered, or discrete or grouped values with their frequencies.

For *stratified data*, enter the data for each stratum in turn, and then click on "*All strata*" to obtain the combined results, which permit appraisal of the association while controlling for confounding effects of the stratifying variable or variables, and assessment of heterogeneity.

For a *meta-analysis*, enter the data for each study in turn (as a separate stratum), and then click on "*All strata*" to compare and combine the results in separate studies.

To compare the changes observed in two groups in a *before-after study* with independent "*before*" and *"after"* observations, enter the before-after data for each group in turn, and then click on "All strata" for heterogeneity tests

For *each table entered*, the program provides **means and medians**, **randomization tests** (unless the samples are large), **extended Mantel-Haenszel tests**, a **Mann-Whitney test**, a **Fligner-Policello robust rank test**, a **Conover squared-rank test for a difference between variances**, **Kolmogorov-Smirnov and Cramer-von Mises tests**, and **measures of association** (the *odds in favour of a higher value* in one group, the *difference between population medians*, *Kendall's rank correlation coefficient,* and *gamma*).

For a *series of tables,* the program provides the **extended Mantel-Haenszel procedure** (including the adjusted differences between mean values and between mean ranks, and a correlation coefficient), the **van Elteren procedure**, a combined P based on **randomization tests**, **tests and measures of heterogeneity**, and **measures of association** (the *odds in favour of a higher value* in one group, the *difference between population medians*, *Kendall's rank correlation coefficient,* and *gamma*).

Means and medians

Mean and median ranks (determined in relation to the combined observations) and mean and median values are displayed for both sets of observations. The mean values are relevant to interval-scale variables only.

Randomization tests

Randomization (permutation) tests are done only if observations are entered individually and (in order to limit the number of possible combinations to 200,000 and thereby avoid lengthy computation) if the samples are not large (see "Methods", below). An option is provided for aborting the computation if it is too time-consuming..

A randomization test computes the exact significance of the difference between the means in the two groups. It determines what proportion of the possible combinations of observations (those in both groups) would yield a difference as extreme as, or more extreme than, the difference actually observed (Siegel and Castellan 1988: 151-155)

Two randomization tests are done for each table. First, the actual values are compared; this is appropriate for an interval-scale variable only. Then the ranks of the values are compared; this is appropriate for both ordinal-scale and interval-scale variables. One-tailed P values are computed and displayed; if 0.05 or less. the value is doubled and reported as a two-tailed P.

For *stratified data*, the P-values obtained in randomization tests in the separate strata are combined to provide an overall test of significance.

Extended Mantel-Haenszel procedure

In each comparison, Mantel's extension of the Mantel-Haenszel test (Mantel 1963) is done twice. First the actual observations are compared; this is appropriate for an interval-scale variable. Then the ranks of the observations are compared; this is appropriate for an ordinal-scale variable or for an interval-scale one (treated as ordinal-scale).. The tests are performed for each table and in the combined analysis (of all strata). One-tailed and two-tailed P values are displayed.

The extended Mantel-Haenszel test is regarded as a test for trend, since it is much more capable of detecting linear and other monotonic relationships than other associations; but a significant result can also be produced by a non-monotonic association (Rothman and Greenland 1998: 314).

In an analysis of *stratified data*, the program computes the overall (adjusted) differences between the mean values and between their ranks, as well as performing overall tests. It also computes correlation coefficients (Mantel 1963); these express the relationships of the observations, and of their ranks, with their group (by allotting scores of 1 and 2, respectively, to groups A and B).

Mann-Whitney test

The Mann-Whitney test (the Wilcoxon-Mann-Whitney test, or Wilcoxon rank-sum test), which is based on the ranks of the observations and is therefore applicable to both interval-scale and ordinal-scale data, compares the locations of the two sets of observations. It can be regarded as a comparison of the median values, on the assumption that the two distributions are similar in shape (with similar variances). The null hypothesis is that the ranks are similar in both groups, and the alternative hypothesis is that the observations in one group tend to have higher ranks than those in the other.

The program reports two-tailed P values, and one-tailed P values for the alternatives (A>B, B>A) to the respective null hypotheses. It may (if numbers are small) display, but not explain, five values (*m*, *n*, *U1*, *U2*, and *Wx*) that can be employed to obtain more exact P values (*see page 49*).

The Mann-Whitney test and related rank-based procedures are not performed if over 100 discrete values are entered.

If *stratified data* are entered, the strata are combined for testing by the *van Elteren procedure* so as to control for possible confounding effects of the stratifying variables. Caution should be used if the strata are small, or if the results in the various strata are very different.

Fligner-Policello robust rank test

This test compares the locations of the two sets of observations. It can be regarded as a comparison of the median values, with no assumption that the two distributions are similar in shape (unlike the Mann-Whitney test). It is applicable to both interval-scale and ordinal-scale data.

One-tailed P values are reported for the alternatives (A>B, B>A) to the respective null hypotheses, as well as the two-tailed P value.

Conover squared-rank test for a difference between variances

The Conover test for equality of variances is based on the squared ranks of the absolute deviations of the values from the mean value in the respective sample, which is assumed to be the population mean..

One-tailed and two-tailed P values are displayed; these are appropriate for large samples.

Kolmogorov-Smirnov and Cramer-von Mises tests

The Kolmogorov-Smirnov two-sample test and the Cramer-Von Mises test are tests for general differences between the distributions in the two groups. They are sensitive to differences in trend, central tendency, spread, and symmetry or skewness.

For each test, a two-tailed P-value is displayed, indicating whether the two sets of frequencies can be supposed not to come from the same unspecified distribution. The Cramer-von Mises test is often more powerful than the Kolmogorov-Smirnov test (Sprent 1993: 128).

Tests and measures of heterogeneity

Heterogeneity tests (for stratified data) are based on the two extended Mantel-Haenszel tests (for the observations and for their ranks).

In parallel with each test, the program also provides two *measures of heterogeneity*, *H* and *I-squared*, with their approximate 95% intervals, for the overall differences between the mean values and between their ranks. An *H* value of less than 1.2 suggests absence of noteworthy heterogeneity, whereas a value exceeding 1.5 suggests its presence, even if the heterogeneity test is not significant. *I-squared* expresses the proportion of variation that can be attributed to heterogeneity (in a meta-analysis, to interstudy variation) rather than to sampling error.

Estimates of the supposed common underlying values of the differences are of questionable value if the findings in the various strata are very disparate.

Measures of association

The program displays the odds in favour of a higher score in one group, the difference between population medians, Kendall's rank correlation coefficient, and *gamma.*

The *odds in favour of a higher score in one group* are the odds in favour of finding that a randomly chosen observation in one group is higher than a randomly chosen observation in the other group. These odds are displayed for each group, with their approximate 95% confidence interval, and for stratified data..

The *difference between population medians* is the median difference between the values in the two samples; an approximate 95% confidence interval is computed. The assumption is that the data come from distributions that are similar in shape (differing only in the magnitude of the values). The difference is not computed if there are very few or very many observations.

Kendall's rank correlation coefficient and *gamma* are based on the ranks of the observations. For stratified data, they are weighted means of the coefficients in the separate strata.

For stratified data, the overall (adjusted) differences between the mean values and between their ranks, and correlation coefficients, are computed by the extended Mantel-Haenszel procedure (see above).

METHODS

Values are ranked in relation to the combined observations (both groups combined), and tied values are allotted the mean of the ranks they would have had had they differed slightly ("tied ranks").

If grouped (but not discrete)data are entered, each observation is allocated the value midway between the lower and upper borders of the group; this may, of course, affect the accuracy of the results.

In each comparison, up to 200 separate observations or sets of grouped values may be entered for each group..

Randomization tests

Randomization tests are done only if observations are entered individually and if the combined number of observations is 20 or less, or if the numbers of observations in the smaller and larger groups do not exceed 6 and 19 respectively, or 5 and 26, or 4 and 44, or 3 and 104, or 2 and 105.

The procedure is based on a BASIC program provided by Siegel and Castellan (1988: 380: Program 3).

For stratified data, the P-values obtained in randomization tests in the separate strata are combined by a logit procedure (George 1977; Mudholkar and George 1979), using formulae 8, 9, and 10 in Hedges and Olkin (1985: 40-41).

Extended Mantel-Haenszel procedure

The procedure is described by Mantel (1963) and explained by (among others) Rothman (1986: 346-348), Breslow and Day (1980: 146-154), and Rothman and Greenland (1998 : 214-315). The one-sided test uses the square root of chi-square (Agresti 1996: 35).

Mann-Whitney test and van Elteren procedure

See page 48.

Fligner-Policello robust rank test

The procedure is described by Siegel and Castellan (1988: 137-144) and Hollander and Wolfe (1999: 135-139).

If either group contains over 12 observations, P is based on a normal approximation. For smaller samples, use is made of critical values of the test statistic (*U*) for P < .1, < 0.05, 0.025. and 0.01 (one-tailed) and P <.2, < 0.1, < 0.05 and <0.02 (two-tailed), provided by Siegel and Castellan (1988: 347: Table K).

Conover squared-rank test for a difference between variances

The Conover test for equality of variances (Conover 1980) is described by Sprent (1993: 124-125). It is based on the squared ranks of the absolute deviations of the values from the mean value in the respective sample. Squared mid-ranks are used for ties (within samples or between samples). A large-sample normal approximation is used (Sprent (1993: 117: formula 5.7).

Kolmogorov-Smirnov and Cramer-von Mises tests

See page 48.

Tests and measures of heterogeneity

The *heterogeneity chi-square* is the sum of the Mantel-Haenszel chi-squares for trend in each stratum minus the overall Mantel-Haenszel chi-square (Rothman and Boice 1982: 9).

The *measures of heterogeneity*, H and *I-squared*, are described by Higgins and Thompson (2002). *H* is computed by Higgins and Thompson's formula 6, and increased to 1, indicating absence of heterogeneity, if it less than 1. A test-based interval is computed by Method III. *I-squared* and its 95% interval are computed from *H*, by formula 10.

Measures of association

The estimate of the *odds in favour of a higher value in one group* is

$$p / (1 - p)$$

where p, the probability that a randomly chosen observation in one group will have a higher value than a randomly chosen observation in the other group, is derived from the Mann-Whitney U statistic (Zar 1998: 147) by the formula

$$p = (U - Ties) / [n_1 n_2 - 2(Ties)]$$

where $Ties = \sum(a_i.b_i / 2)$

n_1 and n_2 = the sizes of the two samples

a_i and b_i = the frequencies of each tied value i in the two samples.

For *stratified data*, the values of $(U - Ties)$ and $[n_1 n_2 - 2(Ties)]$ in the various strata are summated, a pooled value of p is calculated from the summated values, and the pooled value of the odds is computed from the pooled value of p (Abramson and Peritz 1983: 169-170).
The confidence interval for the odds in favour of a higher value in one group is calculated by the method proposed by Halperin, Gilbert and Lachin (1987), as described by Hollander and Wolfe (1999: 130: formula 4.37).

The estimation of the *difference between population medians* is explained by Campbell and Gardner (1988). It is not performed if the product of the two numbers of observations exceeds 10,816, or if either group has <5 observations.

Formulae for *Kendall's rank correlation coefficient* and *gamma* are provided by (among others) Siegel and Castellan (1988: 245, 291). Ties are allowed for in the calculation of Kendall's rank correlation coefficient (Siegel and Castellan 1988: 249: formula 9.10). The pooled values are weighted means of the coefficients in the various strata, using the number of observations in the stratum as the weight.

For stratified data, the overall (adjusted) differences between the mean values and between their ranks, and correlation coefficients, are computed by the extended Mantel-Haenszel procedure (see above).

H2. COMPARISON OF NUMERICAL OBSERVATIONS (NORMAL DISTRIBUTION ASSUMED)

This module provides procedures for comparing two independent sets of numerical observations, assuming approximately normal or lognormal distributions. It may be used for the analysis of trials as well as case-control and other comparative studies. Module H1 of this program should be used if normality cannot be assumed, and module H3 should be used to compare survival data.

The findings in two groups can be compared, or a series of comparisons can be made in different strata or in different studies. For each comparison, individual values can be entered, or discrete or grouped values with their frequencies. This will provide a full set of results (as listed below).. Optionally, mean values can be entered, together with sample sizes and standard deviations, standard errors of the mean, or variances; this will provide more limited results.

For *stratified data*, enter the data for each stratum in turn, and then click on "*All strata*" to obtain the combined results, which permit appraisal of the association while controlling for confounding effects of the stratifying variable or variables, and assessment of heterogeneity.

For a *meta-analysis*, enter the data for each study in turn (as a separate stratum), and then click on "*All strata*" to compare and combine the results in separate studies.

To compare the changes observed in two groups in a *before-after study* with independent "before" and "after" observations, enter the before-after data for each group in turn, and then click on "All strata" for heterogeneity tests.

For *each table entered*, the program provides **descriptions of the frequency distributions** (including tests for normality) based on the raw and log-transformed observations, ***t*-tests**, an ***F* test**, the **difference between means** (with confidence intervals), and the **ratio of geometric means** (with confidence intervals). If means are entered, ***t*-tests**, an ***F* test**, an optional **equivalence test**, and the **difference between means** are provided.

For a *series of tables,* the program provides **overall significance tests**, **tests and measures of heterogeneity**, precision-based estimators of the **overall difference between means** (with confidence intervals), and the **overall ratio of geometric means** (with confidence intervals). If means are entered, the program does not provide the overall ratio of geometric means.

Descriptions of the frequency distributions

For each group, the mean value, its standard error and 90%, 95%, and 99% confidence intervals, and the standard deviation and coefficient of variation are displayed. If the observations are all positive, they are log-transformed, and geometric means (which may be of special interest in serological and microbiological studies) are also computed, with their 90%, 95%, and 99% confidence intervals.

Two *tests for normality* are provided; significant results point to departure from normality The *Lilliefors test*, which examines the deviation of the cumulative frequency from the standard normal cumulative distribution, is performed if there are 6 or more observations; the result is reported as P < 0.01, P < 0.05, P < 0.10, P < 0.15, P < 0.20, or P > 0.20. The *D'Agostini-Pearson test*, which is based on tests for skewness and kurtosis, is appropriate if there are 20 or more observations. If normality is in doubt, consideration should be given to the use of module H1 rather than module H2.

If the values are all positive, these tests are also applied to the log-transformed values, as tests for lognormality. If the distribution is lognormal, the geometric mean is an efficient estimator of the population median (Quan and Zhang 2003).

t-tests

The mean values are compared by two *t*-tests, one assuming equal population variances and using a pooled estimate of the variance, and the other not assuming equal population variances.

If the values are all positive. the *t*-tests are repeated on the log-transformed data.

F test

An *F* (variance ratio) test is performed to compare the variances.

Equivalence test

An equivalence test is offered, to appraise the similarity of the two means.. This may be appropriate if a statistically significant has not been found between the means, e.g. in “negative trials” comparing a new treatment with an established standard treatment, where there may be a reason to prefer the new treatment if it is at least as effective as the standard treatment.

If the test is requested (by using the “*Check here for test of equivalence*” instruction) the bounds of “equivalence” must be defined, by specifying largest difference that is to be regarded as negligible. The test also requires entry of the means and sample sizes, together with standard deviations, standard errors of the means, or variances.

The null hypothesis is that the means of the distributions differ by more than the specified largest-negligible amount, and a significant result supports the alternative that they are equivalent The result is reported as significant at P < 0.01, P < 0.05 or P < 0.1.

Overall significance tests

If stratified data are entered, the program performs overall *Z* tests for the difference between the means and the ratio of the geometric means. In each instance, two alternative estimates of the variance are used in the computation, as in the *t* tests.

Tests and measures of heterogeneity

If stratified data are entered, the heterogeneity of the differences between means (and, if reported, of the ratio of the geometric means) is tested, using both of the alternative variance estimates.

In parallel with each test, the program also provides two *measures of heterogeneity*, *H* and *I-squared*, with their approximate 95% intervals, for the overall differences between the mean values and between their ranks. An *H* value of less than 1.2 suggests absence of noteworthy heterogeneity, whereas a value exceeding 1.5 suggests its presence, even if the heterogeneity test is not significant. *I-squared* expresses the proportion of variation that can be attributed to heterogeneity (in a meta-analysis, to interstudy variation) rather than to sampling error.

Estimates of the supposed common underlying values of the differences are of questionable value if the findings in the various strata are very disparate

Difference between means

For each comparison, the difference between the means is reported with two estimates of its standard error and 90%, 95%, and 99% confidence intervals, based on the two alternative estimates of the variance, as in the *t* tests (see above).

If stratified data are entered, the *overall difference between the means*, controlling for effects connected with the stratifying variable or variables, is estimated by computing the weighted average of the differences in separate strata. The weights are based on the variances, and therefore two estimators of the overall difference, based on the two alternative estimates of the variances, are reported. with their standard errors and 90%. 95%. and 99% confidence intervals.

The results should be treated with caution if there are large differences between the findings in the various strata.

Ratio of geometric means

If the values are positive, so that they can be log-transformed, the ratio of geometric means is reported, with two estimates of its standard error and of its 90%, 95%, and 99% confidence intervals, based on the two alternative estimates of the variance, as in the t tests (see above).

If stratified data are entered, the *overall ratio of geometric means*, controlling for effects connected with the stratifying variable or variables, is estimated by weighting and combining the results in separate strata. The weights are based on the variances, and therefore two estimators of the overall ratio, based on the two alternative estimates of the variances, are reported, with their standard errors and 90%. 95%. and 99% confidence intervals.

METHODS

If grouped data are entered, each observation is allocated the value midway between the lower and upper borders of the group; this may, of course, affect the accuracy of the results.

In each comparison, up to 200 separate observations or sets of grouped values may be entered for each group..

Descriptions of the frequency distributions

For the raw and log-transformed data, the usual formulae are used to compute the mean value, its standard error and confidence intervals, and the standard deviation, variance, and coefficient of variation (Zar 1998: formulae 4.8, 4.13, 6.18 and 7.5). The mean of log-transformed values is back-transformed to the original units, to provide a geometric mean.

The *Lilliefors test* for normality (Lilliefors 1967) is explained by Sprent (1993: 77-78);the program uses critical values provided by Hollander and Wolff (1999: 741: Table A.39). The *D'Agostini-Pearson test* for normality, which is based on tests for skewness and kurtosis (D'Agostino 1986, D'Agostino and Pearson 1973); uses formula 6.19 of Zar (1998).

t-tests

The usual formulae are employed for the *t*-tests (Zar 1998: formulae 8.11 and 8.7a). The formula for determining the degrees of freedom for the test without assuming equal population variances is Zar's formula 8.12, as modified by Welch (Zar 1998: 128-129).

F test

The test is described by Zar (1998: 137).

Equivalence test

The test uses the confidence interval (two one-sided tests) approach (Westlake 1973, Schuirmann 1987). If the 90% confidence interval (for example) of the observed difference falls completely within the interval -D to D (where D is the largest difference specified as negligible), this means that the two one-sided tests are significant at $P < 0.05$, and the equivalence test is significant at $P < 0.1$.

The confidence interval of the difference is derived from the standard error of the difference, which is computed without assuming that the population variances are equal.

Overall significance tests

If stratified data are entered, the significance of the two overall estimators of the difference between means and the ratio of means (see below) is tested by *Z* tests, by dividing the estimators by their standard errors.

Tests and measures of heterogeneity

Heterogeneity tests (for stratified data) are based on formula 10.10 of Fleiss (1981).

The *measures of heterogeneity*, *H* and *I-squared*, are described by Higgins and Thompson (2002). H is computed by Higgins and Thompson's formula 6, and increased to 1, indicating absence of heterogeneity, if it less than 1. A test-based interval is computed by Method III. *I-squared* and its 95% interval are computed from *H*, by formula 10.

Difference between means

Confidence intervals for the difference between means are computed by formulae 8.14 and 8.17 of Zar (1998).

If stratified data are entered, weighted means of the stratum-specific differences, using as weights the reciprocals of the variances of the differences (computed in two ways, first by assuming equal population variances, and then by using a pooled estimate of the variance), are reported as estimators of the overall difference. For formulae for the estimators and their standard errors and confidence intervals, see Gahlinger and Abramson (1995: 86).

Ratio of geometric means

The difference between the means of the log-transformed values is the log of the ratio of the geometric means (see Altman 1991: 201-202). Confidence intervals for the difference are computed by formulae 8.14 and 8.17 of Zar (1998).

If stratified data are entered, the stratum-specific differences between the means of the log-transformed values are weighted and combined, in the same way as the means of the untransformed values (see above), providing estimators of the overall ratio of geometric means (and its standard error and confidence intervals).

H3. COMPARISON OF SURVIVAL TIMES

This module is appropriate for the analysis of trials and follow-up surveys that compare survival times in two independent groups. It can be used for stratified data and for meta-analyses.

A survival time ("time to event") is the number of time units (usually days or months) from the start of observation until the occurrence of a specified end-point event (such as death, the onset of a disease or complication, recovery from a disease, or return to work) or (if the event has not occurred) until withdrawal from observation. The main reasons for withdrawal, or *censoring*, are loss of contact, circumstances that dictate removal from the study, and conclusion of the study.

The findings in two groups can be compared, or a series of comparisons can be made in different strata or in different studies. For each comparison, survival times may be entered separately for each subject, or each survival time can be entered with its frequency. Censored survival times are entered by appending "+", e.g .by entering "37+". Up to 250 survival times may be entered for each group. If one of the groups is a reference group (e.g. controls or "unexposed", it should be entered as group B.

To obtain results that are relevant to specific periods that are of interest, these periods can be entered (e.g., 24 months, to obtain information about 2-year survival).

For *stratified data*, enter the data for each stratum in turn, and then click on "*All strata*" to obtain the combined results, which permit appraisal of the association while controlling for confounding effects of the stratifying variable or variables, and assessment of heterogeneity.

For a *meta-analysis*, enter the data for each study in turn (as a separate stratum), and then click on "*All strata*" to compare and combine the results in separate studies.

To compare the changes observed in two groups in a *before-after study*, enter the before-after data for each group in turn, and then click on "*All strata*" for heterogeneity tests.

The program provides a Kaplan-Meier life-table analysis for each group (**cumulative survival proportions** with their 95% confidence intervals, **median and mean survival times**, and the **incidence rate** of the event), **comparisons of survival proportions, tests comparing survival distributions** (logrank and Gehan-Wilcoxon test), the **ratio of median survival times** and the **hazard ratio** (with 95% confidence intervals), and the **trends in the early and later periods of follow-up**.

If two or more strata are entered, the program can pool the data to provide a Kaplan-Meier life-table analysis for each group (**cumulative survival proportions** with their 95% confidence intervals, **median and mean survival times**, and the **incidence rate** of the event). **Directly standardized survival proportions** are also computed, to control for possible confounding effects of the stratifying variable or variables. The program provides overall **logrank tests** and **hazard ratios** that control for the stratifying variable(s), and , for use in meta-analyses, estimates of the **fail-safe N**. and the **number needed to avoid one event**.

Cumulative survival proportions

For each group, the cumulative survival proportions (expressed as percentages) at each survival time entered are estimated by the Kaplan-Meier procedure. Cumulative survival proportions are also computed for any survival times that have been specified as of special interest, with their approximate 95% confidence intervals; these are large-sample limits, and Rothman and Greenland (1998: 289-90) recommend their use only if at least five events were observed and there are at least five survivors under observation at the time of the calculation; a warning is displayed if these conditions are not met.

The step-by-step survival proportions that are reported provide raw data for the construction of survival curves, consisting of horizontal lines with vertical steps whenever the survival proportion changes.

If stratified data are entered, the Kaplan-Meier procedure is applied to each stratum and to the pooled data.

Directly standardized survival proportions

If stratified data are entered, directly standardized survival proportions are computed for one group (A), based on the fiction that the distribution of the stratifying variable in this group is the same at every survival time entered, as in the other group (B, the reference group). The standardized survival proportions are displayed when the "All strata" button is clicked,. This permits a comparison with the corresponding findings in group B, controlling for the possible confounding effect of the stratifying variable or variable .

Standardized cumulative survival proportions (expressed as percentages) in group A are provided for each survival time entered , and for survival times that have been specified as of special interest. They may not, however, be available for the whole period studied; they are not calculated beyond the last time at which survivors are present in both groups in all strata.

The survival proportions are estimated by the Kaplan-Meier procedure, and then standardized by the method described by Nieto and Coresh (1996), who point out that one advantage of this method of adjustment for confounders is that it permits comparison of the shapes of the curves. The procedure can handle time-dependent covariates.

Direct standardization is not performed if there are over 26 strata.

Median and mean survival times

Where possible, median and mean survival times are reported for each group.

Whether survival times are censored or not, the median survival time is defined as the time at which the cumulative survival probability drops to 50% or below. An approximate standard error and 95% confidence interval are reported; these values may be inaccurate if the sample is small (Machin and Gardner 2000: 97)..

If the survival probability is not precisely 50% at the reported median survival time, an alternative median is also reported, based on linear interpolation between the times straddling the 50% mark.

The program also computes the median survival time expected if the distribution is exponential; if this is very different from the observed median, the assumption of exponentiality can be rejected..

The mean survival time is displayed, with its 95% confidence interval. If there are censored survival times, these values are estimates.

If stratified data are entered, median and mean survival times are reported for each stratum and for the pooled data.

Incidence rate of the event

The average rate of events and its confidence intervals are estimated from the mean survival time and its confidence limits. If any survival times are censored, the rate is an estimate.

If stratified data are entered, the incidence rate is estimated for each stratum and for the pooled data.

Comparisons of survival proportions

For specific survival times that have been specified as being of special interest, the program displays the difference between the survival proportions in the two groups, and the ratio of these proportions, with their approximate 95% confidence intervals. The confidence intervals should be used with caution if the survival times were selected *a posteriori*, after examination of the data (Altman 1991: 376).

If stratified data are entered, these comparisons are performed in each stratum. If comparisons of survival proportions are required for the pooled data, the pooled data should be entered.

Tests comparing survival distributions

Mantel's logrank test and the Gehan-Wilcoxon test compare the survival distributions in the two groups. Both tests allow for censored observations. One-tailed and two-tailed P values are shown.

The *logrank test* statistic is computed with and without a continuity correction; this correction is not recommended if subjects were randomly allocated to the two groups, but it may be appropriate in other circumstances (Peto et al. 1977). If stratified data are entered, an overall logrank test is provided, as well as the logrank tests in the separate strata..

The *Gehan-Wilcoxon test* is more heavily influenced than the logrank test by differences in the occurrence of short survival times (Selvin 1996: 384; Cox and Oakes 1984: 107).

If stratified data are entered, the logrank test is applied in each stratum and in the pooled data. The Gehan-Wilcoxon test is applied in each stratum; if it is required for the pooled data, the pooled data should be entered.

Ratio of median survival times

Approximate 95% confidence limits are computed for the ratio of the median survival times in the two groups, on the assumption that the survival times have an exponential distribution (Simon 1986;

Altman 1991, pp 384-385). To permit this assumption to be checked, the program computes the medians that would be expected if the distributions were exponential, for comparison with the observed medians.

If stratified data are entered, the ratio is estimated in each stratum; if it is required for the pooled data, the pooled data should be entered.

Hazard ratio

The hazard ratio, which is similar to a relative risk, expresses the relative survival experience of the two groups. A approximate 95% confidence interval is computed. The program also displays the values (in each group) on which the hazard ratio is based – the number of observed events and the "extent of exposure" or "expected events", and their ratio.

For stratified data, the overall hazard ratio is computed, with its approximate 95% confidence interval.

Trends in the early and later periods of follow-up

As a simple indication of possible time-related differences between the survival distributions, the program summarizes the change in the cumulative survival proportion in each group, in the early and later segments of the follow-up period (usually using the median survival period for Group A as the cutting-point). The change is expressed as the drop in the survival percentage.

Comparison of the changes may point to trends that are different in the two groups or time periods. Differences in trend in the two periods may be obscured in the overall results.

If stratified data are entered, these trends are appraised in each stratum. If this appraisal is required for the pooled data, the pooled data should be entered.

Fail-safe N

If stratified data are entered, estimates of the fail-safe N are provided for use in *meta-analyses* (on the assumption that the strata represent separate studies), as rough guides to the possible importance of the "file drawer problem", i.e. the exclusion of studies that were not published or were not found for other reasons.

The program computes the numbers of new "null" studies (those with a hazard ratio of 1) that will suffice to bring the overall hazard ratio to a negligible level (0.8, 0.9, 1.1 or 1.2). No account is taken of P-values or the size of the null studies.

Number needed to avoid one event

For use in studies in which the events are avoidable, the program reports the number of individuals who are needed in the group with a longer survival time, in order to avoid a single case.

If stratified data are entered, the number needed is reported for each stratum; if it is required for the pooled data, the pooled data should be entered.

METHODS

Cumulative survival proportions

Cumulative survival proportions are estimated by the Kaplan-Meier technique (Kaplan and Meier 1958; Armitage *et al.* 2002: 575-576; Machin and Gardner 2000: 94-96).

95% confidence intervals for survival proportions at specific selected times are computed from the estimated variance of the logit of the proportion, using Greenwood's formula (Rothman and Greenland 1998: 289-90).

Directly standardized survival proportions

Standardization is performed by the method described by Nieto and Coresh (1996), using their formulae 1, 2, and 3. Occasional anomalous rises in the standardized survival proportion are corrected by reverting to the previous value. Standardization is performed if up to 200 times re entered;

Median and mean survival times

The *median survival time* is defined as the time at which the cumulative survival probability drops to 50% or below. Its approximate standard error and 95% confidence interval are computed by the formulae provided by Machin and Gardner (2000: 97-98), based on the survival times at which the survival probabilities reach or cross the 45% and 55% levels, or if these probabilities are equal, the 40% and 60% levels. The effective sample size required for the calculation is the total sample size minus the number censored before the median survival time (Machin and Gardner (2000: 94). If the sample is small, the results are unreliable.

If the survival probability is not precisely 50% at the reported median survival time, an alternative median is also reported, based on linear interpolation between the times straddling the 50% mark (Selvin 1996: 374).

The median survival time expected if the distribution is exponential is the sum of the survival times (whether censored or not) divided by the number of events (Altman 1991: 385).

The **mean survival time** and its confidence intervals are computed in the usual way if no survival times are censored,. Otherwise, a nonparametric estimate of the mean is computed, based on formula 11.29 of Selvin (1996: 371); its standard error is computed by formula 11.31 and used for interval estimation; for this purpose, the longest survival time is treated as uncensored, even if it is censored.

A mean/median survival time is also computed, based on the assumption that the distribution is exponential (Selvin 1996, formula 11.19; Altman 1991: 385). Its standard error is computed by Selvin's formula 11.20.

Incidence rate of the event

Since (in a closed population) an incidence rate is the reciprocal of the average time until occurrence of the event (Rothman 1986: 29; Morrison 1979), the reciprocals of the mean survival time (or the estimate of the mean survival time) and its confidence limits are used as estimates of the average rate of events and its confidence limits.

Comparisons of survival proportions

For comparisons of survival proportions, the estimation of the variances and confidence intervals of the differences and ratios is described by Rothman and Greenland (1998, 291-292). Formulae 16-15 and 16-16 are used, based on. the estimated variances of the logits of the proportions (Rothman and Greenland 1998, pp 289-90).

Tests comparing survival distributions

For the *logrank test*, a variance-based formula is used (Armitage *et al.* 2002: 577: formula 17.12); but if the variance is zero, formula 17.15 is used. The test statistic is computed with and without a continuity correction. Stratified data are combined by the method described by Peto *et al.* (1977).

The *Gehan-Wilcoxon test* is performed by the procedure described by Sprent (1993: pp 118-120), using a large-sample normal approximation (Sprent 1993: 117, formula 5.7) to appraise the sum-of-scores test statistic.

Ratio of median survival times

The computation of a confidence interval for the ratio of the median survival times in the two groups (Simon 1986), on the assumption that the survival times have an exponential distribution, is described by Altman (1991: 384-385). The median survival times used for this purpose are those at which the cumulative survival probability drops to 50% or below.

Hazard ratio

The program computes the Pike hazard ratio estimator (Pike 1972), with its confidence interval based on the large-sample variance of its log (Berry *et al*. 1991, formula 1). If there are no events in the reference group the confidence interval is estimated by the method of Machin and Gardner (1988), using the Peto hazard ratio estimator (Yusuf *et al.* 1985).

Stratified data are combined by the method described by Peto *et al*. (1977). The estimation of the confidence interval of the hazard ratio is described by Machin and Gardner (1988).

Trends in the early and later periods of follow-up

Changes in the survival percentage in each group are reported, in the early and later periods of follow-up. The cutting-point used for this purpose is based on the median survival period for Group A (or, if this median is not reached, on the point at which the cumulative survival proportion drops to 60%). The longest survival time entered determines the end of the later period. Where possible, the interval defined for Group A is applied to Group B also. Linear interpolation is used where necessary.

Fail-safe N

The fail-safe N is computed by the following formula, which is derived from Orwin's effect-size formula (Orwin 1983; Hedges and Olkin 1985, formula 9, p. 306; or Iyengar and Greenhouse 1988, formula 5):

Fail-safe N = k[abs(R) - abs(C)] / abs{C}

where k = No. of studies included in the analysis
R = log of the overall hazard ratio
C = log of the chosen "negligible value" [0.8, 0.9, 1.1 or 1.2).

Number needed to avoid one event

The number of individuals who are needed in the group with a longer survival time in order to avoid a single case is computed from the difference between survival proportions and its estimated variance (Altman and Anderson 1999),

I. ANY COMPARISON, USING SUMMARY MEASURES FOR EACH STRATUM OR STUDY

This module is designed for use in meta-analyses or studies of stratified data, if a measure of association or a P-value is available for each of the component studies or strata. In such instances it is not necessary to enter detailed data for each study or stratum (as in modules A to H of this program). The module is particularly appropriate for meta-analyses based on study reports that provide measures of association or P-values without the detailed data on which these were based. It is applicable to the results of studies (trials, or cross-sectional, cohort or case-control studies) that compare two independent groups or samples.

The measure of association may be a ratio measure (odds ratio, risk ratio, rate ratio, or hazard ratio), the difference between risks or rates or means, the effect size (the standardized difference between means), or other measures with an approximately normal distribution and a zero value when there is no association (including *kappa*, the Z transformation of a correlation coefficient, a simple regression coefficient, Kendall's *tau*, and the population attributable fraction). The module can also be used to calculate a weighted mean (precision-based) of a set of simple proportions.

In most instances it is necessary to enter the value observed in each study or stratum, together with either a standard error or a confidence interval (90%, 95%, or 99%). This applies to ratio measures, differences between risks or rates, and other measures with an approximately normal distribution and a zero value when there is no association. For ratio measures, the required standard error is the standard error of the log of the ratio. The accuracy of the results depends on the accuracy of the entries; entry of rounded-off numbers will yield approximate results.

For effect sizes (standardized differences between means), the required entries (for each study or stratum) are the difference between the means, and each sample's size and standard deviation.

If measures of association are entered. the program combines and compares them. It provides **overall measures** of association, using fixed-effect and random-effects models (with standard errors, significance tests, and confidence intervals), and **tests and measures of heterogeneity.** For use in meta-analysis, it also provides estimates of the **fail-safe N**, two **tests for a skewed funnel plot** (suggesting publication bias**)**, and a **sensitivity analysis** showing the effect (on the overall measure, the tests, and heterogeneity) of excluding each study or stratum in turn

If P-values are entered, they must be one-sided P-values testing the same direction of effect. An **overall probability** is then computed. Optionally, a weight can be entered together with each P-value (e.g. the sum of the sizes of the two samples in the study or sample)., and the P-values will then be weighted both by the weights and by the square roots of the weights. The program also provides a **heterogeneity test**, the **fail-safe N** and a **sensitivity analysis** showing the effect of excluding each study or stratum in turn.

Overall measures

If measures of association are entered, two overall measures are computed, using fixed-effect and random-effects models respectively. Using the fixed-effect model, which assumes that the individual studies or strata provide estimates of the same true effect, a *precision-based* estimator is computed; this is a weighted average that uses the reciprocal of the variance in each study or stratum as the weight for the study or stratum; it is not identical with the (preferable) measures computed by modules A to H. Using the random-effects model, which assumes that the studies or strata provide estimates of randomly differing effects, a *DerSimonian-Laird* estimator is computed; this takes account of the variation between studies or strata, resulting in wider confidence intervals and a more conservative significance test. The random-effects model gives more weight to small studies, and may be inappropriate if sample sizes are very small. Some investigators use it when unexplained heterogeneity is present (Fleiss and Gross 1991, Petitti 1994, Whitehead and Whitehead 1991). "In essence," say Rothman and Greenland (1998: 668), "a random-effects model exchanges a doubtful homogeneity assumption for a fictitious random distribution of effects . The advantage ... is that the standard errors and confidence limits ... will more accurately reflect uncertainty about unaccounted-for sources of variation". The Dersimonian-Laird estimator is not displayed if this procedure does not increase the variance of the overall measure.

For each estimator, a chi-square test is performed, appraising whether the estimator differs from zero, and a standard error and 90. 95, and 99% confidence intervals are displayed..

The overall values are of questionable validity if the separate values are widely discrepant, e.g. if they demonstrate associations in opposite directions or if the heterogeneity test is significant at a conservative level of significance (Fleiss 1981: 164). The procedure should not be used for small samples.

If *effect sizes* (standardized differences between means) are used, the program provides two aids to the appraisal of the overall effect size. First, it displays the approximate percentage of members of one group whose values fall below the mean of the other group (Glass *et al.* 1981: 29), based on the assumption that the population variances in the two groups are equal. Secondly, it computes an odds ratio (Tritchler 1995) that expresses the accuracy with which individuals would be allocated to the two groups on the basis of likelihood ratios derived from a comparison of the distributions, using any arbitrary cut-point. It is the ratio of the odds in favour of correctly classifying members of either one of the groups to the odds in favour of incorrectly classifying members of the other group. Because effect sizes are "unitless", they can be used for combining the results of studies that use different measurement scales; but, as pointed out by Greenland (1987), Petitti (1994: 123) and others, this may be misleading.

If *simple proportions* are entered, a weighted mean value (precision-based is computed), using the inverse of the variance of each proportion as its weight. Most other results will be irrelevant.

Overall probability

If measures of association are entered, the overall probability is measured by chi-square tests that appraise whether the estimator of the overall measure differs from zero.

If P-values are entered the program combines them, to provide an overall test of significance, on the assumption that they are based on tests of essentially the same hypothesis. The studies from which the P-values are obtained must be independent of one another, but different tests may be used –

neither the statistic nor the outcome variable has to be identical. For example, P-values from a *t* test, chi-square test, and other tests may be combined to produce a summary P-value. A summary P-value can, however, lead to misrepresentations if the individual test statistics do not have a continuous distribution (Oldham 1968: 114-115; Mosteller and Bush 1954).

Several summary P-values are computed, using procedures suggested for this purpose by Fisher (1948), George (1977), Stouffer *et al.* (1949: 45), and Mosteller and Bush (1954). One-tailed and two-tailed summary P-values are displayed.

One-sided P-values must be entered, all testing the same direction of effect. It is generally satisfactory to halve the two-sided value, but if the direction of the observed effect is opposite to that of the study hypothesis, the halved two-sided P-value should be subtracted from 1 before entry.

Optionally, weighting factors may be entered, for use in the calculation of a weighted average of the separate test statistics (Mosteller and Bush 1954). If they are entered, the program provides three sets of results for Stouffer's and George's procedures: unweighted, weighted by the factors entered, and weighted by the square roots of the factors entered. The weighting factor may be the sample size (i.e., the combined sizes of the groups that are compared), or some measure of study quality (DeMets 1987). Which weighting system is best is uncertain. In one comparative study, weighting by the square root of the sample size yielded a summary P-value similar to those computed by the Cochran-Peto and log odds ratio procedures for combining data from independent four-fold tables.; these methods essentially weight by the square root of sample size (Canner 1987).

Hedges and Olkin (1985) compare the statistical properties of various methods. Simulation studies of power suggest that George's logit procedure (George 1977) may be nearly optimal for a variety of situations.

Tests and measures of heterogeneity

The program provides *heterogeneity tests* for the measures of association in the different studies or strata. These tests should be interpreted with caution, since their power is low; if the result is significant at the 0.05 level, the hypothesis of homogeneity can be rejected; but "a high p-value ... does not show that the measure is uniform, it only means that heterogeneity ... was not detected by the test" (Rothman and Greenland 1998: 276); the larger the strata, the more valid the test.

The program also provides two *measures of heterogeneity*, *H* and *I-squared*, with their approximate 95% intervals, for the measures of association. An *H* value of less than 1.2 suggests absence of noteworthy heterogeneity, whereas a value exceeding 1.5 suggests its presence, even if the heterogeneity test is not significant. *I-squared* expresses the proportion of variation that can be attributed to heterogeneity (in a meta-analysis, to interstudy variation) rather than to sampling error.

The estimates of the overall measure of association are of questionable value if the findings in the various studies or strata are very disparate. If the results are not uniform, explorations of possible causes – e.g. associations with study design or quality or with the sizes or other characteristics of the samples – may be revealing

The uniformity or heterogeneity of the measures in the different studies or strata can be appraised not only by these tests and measures, but by plotting the values and their confidence intervals graphically, and comparing them.

If P-values are entered, the heterogeneity test (which is based on the dispersion of the Z scores) yields a low P-value if the individual test statistics are very dissimilar. The test is not very meaningful if the sample sizes for individual tests are small.

Fail-safe N

Estimates of the fail-safe N are provided for use in *meta-analyses*, as rough guides to the possible importance of the "file drawer problem", i.e. the exclusion of studies that were not published or not found for other reasons.

If *ratio measures* (odds, risk, rate, or hazard ratios) are entered, the program computes the numbers of new "null" studies (those with a ratio measure of 1) that will suffice to bring the overall ratio measure to a negligible level (0.8, 0.9, 1.1 or 1.2).

If *effect sizes* are used, the program computes the numbers of new "null" studies (those with an effect size of 0) that will suffice to bring the overall effect size to a negligible level (0.1 or 0.2).

If *P-values* are entered, the fail-safe N is computed if the overall test is significant (i.e. if the summary P-value is 0.05 or less by the Stouffer method without weighting). The fail-safe N is an estimate of the number of nonsignificant tests that must be added in order to push the overall P above 0.05. If the overall test is significant at the 0.01 level, the number of null tests required to push P above 0.01 is also computed. At each of these two levels, the fail-safe N is computed by two methods. The first (Rosenthal 1979) is based on the assumptions that the null hypothesis is true and that all significant results at the given two-tailed P level have been included. The second method, which yields a lower fail-safe N, has been called a "worst-case" calculation (Rosenthal and Rubin 1988; Iyengar and Greenhouse 1988b). It allows for a stronger bias in the ascertainment of studies, and makes the assumption that significant results at a given one-tailed P level have been included, and missing studies are not significant or are significant in the opposite direction.

Tests for a skewed funnel plot

If measures of association are entered, two tests for a skewed funnel plot are performed, for use in meta-analysis. The tests, which examine the association between the sizes of the effects in the component studies and their precision, are the *regression asymmetry test* and the *adjusted rank correlation test.*

In each test, a low P value suggests possible *publication bias*, although the "small-study effect" (Sterne *et al.* 2000) that it indicates may have some other cause, such as the use of higher-risk subjects in smaller studies, resulting in an association between sample size and the effect under consideration.

Both tests have a low power if they are based on few component studies. The regression asymmetry test is generally more powerful (Egger *et al.* 1997; Sterne *et al.* 2000). A critical P level of at least 0.1 should be used for both tests.

Sensitivity analysis

In a meta-analysis, it may be considered unwise to draw a conclusion that hinges on a single study. Therefore, to permit examination of the possibility that the overall results are unduly affected by a

single study or stratum, the program calculates a set of "partial" overall results (using the fixed-effect model), each time excluding a different component study or stratum.

If *measures of association* are entered, each set of "partial" results comprises an overall measure of association (with its 95% confidence interval), a chi-square test, and tests and measures of heterogeneity. In addition, the program tests the significance of the difference between each component measure and the overall measure based on all other measures; two P-values are displayed for each test, one for 1 degree of freedom and one for *k*-1 degrees of freedom (*k* = total number of P-values); the latter P-value should be used if the comparison was suggested by the data. The sensitivity analysis for measures of association is omitted if there are many strata.

If *P-values* are entered, a set of overall P-values (one-tailed, George's method) is provided, each one based on all the component P-values except one.

METHODS

Up to 100 studies or strata may be entered.

If confidence intervals for measures of association are entered, standard errors are computed on the assumption that the confidence limits are equidistant from the point estimate (or, for ratio measures, that their logs are equidistant from the log of the point estimate).

During processing, zeros are converted to 0.00001 when necessary to avoid computation errors.

Overall measures of association

The precision-based procedures are described by Fleiss (1981: 161-164, 185, 302) and Kleinbaum *et al.* (1982: 341-342, 359-361). The DerSimonian and Laird procedure is described by DerSimonian and Laird (1986) . Zero ratios are changed to 0.00001. The Q statistic, which plays a central role in the analysis, is based on the data for separate strata and the precision-based estimate of the common measure. If Q + 1 is less than the number of strata the random-effects and fixed-effect models yield identical results. Results may differ slightly from those provided by modules A to D, which use the Mantel-Haenszel estimate of the common measure, not the precision-based estimate.

For *effect sizes* (standardized differences between means), the analysis follows the lines described by Petitti (1984: 119-123). Pooled standard deviations (assuming equal population variances) and approximate variances of effect sizes are computed by formulae from Hedges and Olkin (1985: 79, 80). The formula for the odds ratio (based on Tritchler 1995) is

$$\text{Odds ratio} = [a \ / \ (1 - a)]^2$$

where $a = 1 - P$
P = one-tailed probability for Z (standardized normal deviate)
$Z = |\text{Effect size}| \ / \ 2$

Overall probability

Fisher's method (Fisher 1948) of combining probabilities is based on the product of the P-values, and *Stouffer's method* (Stouffer et al. 1949: 45) on the sum of the *Z* values (standard normal deviates) derived from the P-values. The formulae for Fisher's and Stouffer's methods are described by DeMets (1987). *George's logit procedure* (George 1977; Mudholkar and George 1979) uses formulae 8, 9, and 10 in Hedges and Olkin (1979: 40-41). When combining P-values, P-values less than 0.0000001 are changed to 0.0000001, and P-values exceeding 0.9999999 are changed to 0.9999999. If weighting factors are entered, the P values or (for Stouffer's method) *Z* values are weighted before summation (Mosteller and Bush 1954).

Tests and measures of heterogeneity

If *measures of association* are entered, the heterogeneity chi-square tests are based on formula 10.5 of Fleiss (1981)

If *P-values* are entered, heterogeneity is appraised by the following formula (Wolf 1986: 23):

$$\text{chi-square } (k\text{-1 d.f.}) = \sum[Z_i - (\sum Z_i \;/\; k)^2]$$

where Z_i = the test statistic in the individual test
k = the number of tests.

The *measures of heterogeneity*, *H* and *I-squared*, are described by Higgins and Thompson (2002). H is computed by Higgins and Thompson's formula 6, and increased to 1, indicating absence of heterogeneity, if it less than 1. A test-based interval is computed by Method III. *I-squared* and its 95% interval are computed from *H*, by formula 10.

Fail-safe N

Orwin's formula is used for *effect sizes* (Orwin 1983; Hedges and Olkin 1985, formula 9, p. 306; or Iyengar and Greenhouse 1988, formula 5), and a formula derived from this is used for *ratio measures*;:

$$\text{Fail-safe N} = k[\text{abs}(R) - \text{abs}(C)] / \text{abs}\{C\}$$

where k = number of studies included in the analysis
R = log of the overall odds ratio or risk ratio
C = log of the chosen "negligible value" [0.8, 0.9, 1.1 or 1.2).

If P-values are entered and the summary P-value is 0.05 or less by the Stouffer method without weighting, the fail-safe N is computed by two methods. The first (Rosenthal 1979) is based on the assumptions that the null hypothesis is true and that all significant results at a given two-tailed P level have been included. Rosenthal's formula is

$$N = \sum(Z_i)^2 / 2.7055 \quad \text{for a critical level of } P <= 0.05$$

or $$N = \sum(Z_i)^2 / 5.4119 \quad \text{for a critical level of } P <= 0.01$$

where Zi = the Z value (standard normal deviates) derived from the specific P-value.
The second method yields a lower fail-safe N, and has been called a "worst-case" calculation (Rosenthal and Rubin 1988; Iyengar and Greenhouse 1988b). It allows for a stronger bias in the ascertainment of studies, and makes the assumption that significant results at a given one-tailed P level have been included, and omitted studies are not significant or are significant in the opposite direction. The equation (Iyengar and Greenhouse (1988a: formula 4) is:

$$N = [-b - \sqrt{(b^2 - 4ac)}] / 2a$$

where a = 0.01177 for a critical level of P <= 0.05 or 0.0007236 for a critical level of 0.01
$b = -0.217\sum Z_i - 2.70554$ for a critical level of 0.05 or $-0.0538\sum Z_i - 5.4119$ for a critical level of 0.01
$c = (\sum Z_i)^2 - 2.70554k$ for a critical level of 0.05 or $(\sum Z_i)^2 - 5.4119k$ for a critical level of 0.01
k = the number of studies included in the analysis
$\sum Z_i$ = the sum of the test statistics in the k studies

The estimates of the fail-safe N are rounded off to the nearest whole number. If N < 1 (but not zero) it is taken as 1.

Tests for a skewed funnel plot

The *regression asymmetry test* (Egger *et al.* 1997) uses linear regression. It regresses the standard normal deviate (SND), defined as the measure of association divided by its standard error) against precision (the inverse of the standard error of the effect measure). If a ratio measure is used, its log is used, in both this test and the adjusted rank correlation test. The regression equation is

$$\text{SND} = \text{intercept} + b \text{ x precision.}$$

In the absence of bias, an intercept of zero is expected. The program reports the intercept and its 90% confidence interval, and tests its difference from zero; two-tailed P is displayed. The usual formulae for least-squares linear regression are used (e.g. Woolson and Clarke 2002: 309-311; Zar 1998: formula 6.21).

The *adjusted rank correlation test* (Begg and Mazumdar 1994) uses Kendall's rank correlation (Siegel and Castellan 1988: 245-54) to appraise the association between the sizes of the effects in the component studies (after first standardizing these effect measures) and their standard errors. In the absence of bias, a tau of zero is expected. Allowance is made for ties in the computation. If there are 30 or fewer component studies, tables of critical levels for one-tailed P = 0.05, 0.025, 0.01, and 0.005 (Siegel and Castellan 1988: Tables RI and RII) are used. If two-tailed P exceeds 0.01 according to these tables, and for larger samples, a Z test (making allowance for ties) is used (Armitage *et al.*2002: 290). The two-tailed P value is displayed.

M. EFFECT OF MISCLASSIFICATION

This module appraises the effect of misclassification (nondifferential or differential) on a 2 x 2 table. It demonstrates the effect of the sensitivity and specificity of the measures on the association between two dichotomous variables. by computing the “true” findings that would give rise to the observed findings..

The observed findings, the type of study. and the sensitivity and specificity of the measures must be entered. The program labels the variables as “disease” and “exposure, but it may be applied to any pair of dichotomous variables.

The study may be a study of a population or a representative sample of a population, or a comparison of two independent samples.

The sensitivity and specificity of both measures (of disease and of exposure) must be entered. If these differ in the two groups (differential validity), separate entries are required for each group. Estimates of sensitivity and specificity in the target population, not the sample, should be entered. These may be based on a validity study in the population studied or on other information..

The procedure assumes that the probability of misclassification of one variable is independent of the probability of misclassification of the other.

The “true” results computed by the program are the cell frequencies, the odds ratio, and, in studies of a total population or comparisons of exposed and unexposed groups, the risk ratio (the ratio of the proportion with the disease among the exposed to the proportion with the disease among the unexposed, with approximate 95% confidence intervals for these ratios.

The computed “true” results are not shown if they are unrealistic (if a “true” frequency is negative). A message is displayed saying that the observed frequencies are not compatible with the sensitivity and specificity values, and that if the entries are correct, the findings may represent sampling error.

METHOD

The program constructs a 4 x 4 matrix representing four equations that express the relationship between the observed and true (correctly classified) frequencies, and solves them by calculating the inverse of the matrix and postmultiplying this by a vector composed of the observed frequencies. The procedure, a generalization of Barron's procedure for nondifferential misclassification (Barron 1977), is described by Kleinbaum, Kupper and Morgenstern (1982: 228-236) and Greenland and Kleinbaum (1983). If the matrix is not invertible an error message is displayed..

If different sample ratios were used in the two groups, the calculations are based on the frequencies in the target population (Greenland and Kleinbaum 1983) (using the entered prevalences) and the corrected frequencies are subsequently scaled down to make them correspond to the sizes of the samples..

Approximate 95% confidence intervals are computed for the modified estimates of the odds and risk ratios, based on the adjusted frequencies, after rounding them off to the nearest integer. These intervals are derived from the standard errors of the logs of the modified values of the odds and risk ratios (Morris and Gardner 2000:. 58 and 61). Uncertainty of the sensitivities and specificities is not taken into consideration.

P1. POWER OF TEST FOR COMPARISON OF PROPORTIONS

This module estimates the power of exact and chi-square tests for detecting a difference of a given magnitude between two independent proportions, given the significance level and sample sizes. For *exact tests* (Fisher and mid-P) it computes the *expected power* (also called *overall, average* or *unconditional* power) (Bennett and Hsu 1960, Hirji *et al*. 1994), which is particularly appropriate when a study is being designed.. Computation of the power of exact tests is slow if the samples are large, and can be optionally aborted; it is not done for very large samples..

Besides the significance level (a one-tailed or two-tailed value may be entered) and sample sizes, the program requires entry of one of the proportions, and the magnitude of the difference to be detected. The samples are designated A and B, and the required proportion is the known or assumed proportion in sample B. If there is a control (comparison) group (non-cases in a case-control study, or subjects not exposed to a supposed risk or protective factor, or to an experimental treatment in a trial), it should be called sample B. The magnitude of the difference to be detected can be indicated by entering an odds ratio, a risk ratio (the ratio of the proportions), or the proportion in sample A.

If losses of sample members are expected (non-inclusion in the analysis because of failure to find addresses, nonresponses, dropouts etc.), allowance should be made for this before entering the intended sample sizes, by multiplying them by $(100 - R)^2 / 10000$, where R is the percentage of expected losses (Lachin 1981). this does of course not compensate for possible bias.

The effects of changing the significance level, sample sizes or other parameters are easily examined..

The program computes the power of Fisher's and mid-P exact tests and the power of a chi-squared test, with and without a continuity correction. The continuity correction reduces the power estimate. If an odds ratio of 1 is entered, the program computes the "true" type I error proportion (Casagrande *et al*. 1978b).

The program can also be used to determine the sample sizes required for an exact test with a given power, by entering different sample sizes in a series of trial-and-error estimations of expected power, until the required power is attained (Hirji *et al*. 1994).

METHODS

For *exact tests*, power is computed by formulae provided by Casagrande *et al.* (1978a) and Bennett and Hsu (1960). It is not computed if the combined sample sizes exceeds 1754. The accuracy of the computations has been checked against a program (Hirji *et al*. 1994) kindly provided by Prof. S.E. Vollset.

A number of methods of computing power for *chi-square tests* are available, and their results differ (Sahai and Khurshid 1996). For computing power without a continuity correction, this program used a formula derived from formula 3.19 in Fleiss (1981); this is formula 24.76 in Zar (1998, p. 560) and formula 22 in Sahai and Khurshid (1996). Power with a continuity correction is computed by formula 52 in Sahai and Khurshid (1996).

P2. POWER OF TEST FOR COMPARISON OF PROPORTIONS: STRATIFIED DATA

This module estimates the power of a Mantel-Haenszel test (Mantel and Haenszel 1959) or Cochran test (Cochran 1954), on the assumption that the odds ratio is the same in all strata . It regards the relative number of members of the two groups (e.g. cases and controls in a case-control study) in each stratum as fixed (Woolson, Bean and Rojas 1986).

Besides the significance level (a one-tailed or two-tailed value may be entered), the total sample size, the odds ratio to be detected, and the number of strata, the program requires three items of information (known or assumed) for each stratum: the stratum's relative size (e.g. the number of subjects, or the percentage or proportion of the total sample); the ratio (in this stratum) of the size of sample B to the size of sample A; and the proportion (expressed as a percentage) in sample B in this stratum (i.e., the percentage of sample B, in this stratum, who have the attribute under study). If there is a control (comparison) group (non-cases in a case-control study, or subjects not exposed to a supposed risk or protective factor, or to an experimental treatment in a trial), it should be called sample B.

If losses of sample members are expected (non-inclusion in the analysis because of failure to find addresses, nonresponses, dropouts etc.), allowance should be made for this before entering the intended sample sizes, by multiplying them by $(100 - R)^2 / 10000$, where R is the percentage of expected losses (Lachin 1981). this does of course not compensate for possible bias.

The effects of changing the significance level, sample sizes or other parameters are easily examined

The program estimates power for tests with and without continuity corrections. The continuity correction reduces the power estimate

METHOD

The computation of power for a stratified case-control study is based on the Cochran-Mantel-Haenszel statistic expressed as a weighted average of the difference between two independent binomial proportions (in the two groups that are compared). The formula (without a continuity correction) is derived from formula 2.8 of Woolson, Bean and Rojas (1986). For a continuity correction, use is made of the correction factor defined in formula 2.5.

P3. POWER OF TEST FOR COMPARISON OF DISTRIBUTIONS IN ORDERED CATEGORIES

This module estimates the power of a Mann-Whitney (Wilcoxon rank sum) test used to compare two independent samples with respect to the distribution of an attribute that has ordered categories (Mann and Whitney 1967; Wilcoxon 1945).. The test might be used (for example) to compare levels of exposure to a supposed causal factor (e.g. heavy smokers, light smokers, nonsmokers) in a case-control study, or levels of outcome (e.g. severe illness, moderate illness, mild illness or no illness) in a cohort study or trial.

A proportional odds model is employed. That is, it is assumed that when the 2 x *k* table that displays the presence of the attribute in a set of *k* ordered categories is converted to a 2 x 2 table by combining adjacent categories, the odds ratio is the same, whatever cutting-point is used.

The samples are labelled A and B If there is a control (comparison) group (non-cases in a case-control study, or subjects not exposed to a supposed risk or protective factor, or to an experimental treatment in a trial), it should be called sample B.

Besides the significance level (a one-tailed or two-tailed value may be entered), the sizes of the two samples, and the number of categories, the program requires entry of the relative sizes (known or assumed) of the categories in sample B (e.g. numbers of subjects, or the percentage or proportion of sample B falling into each category) , and either the odds ratio to be detected, or the percentage of sample A that is expected or known to be in the first category.

If losses of sample members are expected (non-inclusion in the analysis because of failure to find addresses, nonresponses, dropouts etc.), allowance should be made for this before entering the intended sample sizes, by multiplying them by $(100 - R)^2 / 10000$, where R is the percentage of expected losses (Lachin 1981). this does of course not compensate for possible bias.

The procedure is based on a normal approximation, and may not be accurate for small samples.

The effects of changing the significance level, sample sizes or the number of categories are easily examined.

The program displays the power of the test and (if the odds ratio was not entered) the odds ratio computed from the percentage of sample A in the first category.

METHOD

The formula is derived from formula 10 of Whitehead (1993). Allowance is made for ties.

If the percentage of sample A that is expected or known to be in the first category is entered instead of the odds ratio, the odds ratio is calculated, assuming a proportional odds model.

P4. POWER OF TEST FOR COMPARISON OF MEANS

This module estimates the power of a *t* test for detecting a difference of a given magnitude between the means in two independent populations, given the significance level and sample sizes. The results should be used with caution if the samples are very small.

Besides the significance level (a one-tailed or two-tailed value may be entered), the sizes of the samples, and the magnitude of the difference to be detected, the program requires entry of either the known or assumed pooled variance or the known or assumed standard deviations in the two samples.

If losses of sample members are expected (non-inclusion in the analysis because of failure to find addresses, nonresponses, dropouts etc.), allowance should be made for this before entering the intended sample sizes, by multiplying them by $(100 - R)^2 / 10000$, where R is the percentage of expected losses (Lachin 1981). this does of course not compensate for possible bias.

The effects of changing the significance level, sample sizes or other parameters are easily examined..

METHOD

The program uses the method described by Lachin (1981) in formula 7, after dividing the sample size by the correction factor described.

If the pooled variance V is not entered it is computed as follows:

$$V = S_A^2 (Q_A) + S_B^2(Q_B)$$

where S_A and S_B = standard deviations of Samples A and B respectively
QA and QB -= proportions of subjects in Samples A and B respectively

S1. SAMPLE SIZES FOR COMPARISON OF PROPORTIONS

This module computes the sizes of the samples required to detect a true difference of a given magnitude between proportions in two independent samples, with a given significance level and a given power or precision.

The samples are labelled A and B If there is a control (comparison) group (e.g. subjects not exposed to a supposed risk factor or to an experimental treatment), it should be called sample B.

The desired significance level (*alpha*) for a two-sided test, the ratio of the sizes of the two samples, the known or expected proportion in Sample B, and the magnitude of the difference to be detected must be entered. If a one-tailed test is wanted, *alpha* must be doubled; e.g., 10% should be entered to obtain sample sizes for a 5% significance level. The size of the difference to be detected can be indicated by entering an odds ratio, the ratio of the proportions, or the proportion in sample A. The effects of changing the significance level or other parameters are easily examined

To stipulate the *power* of the test, it is entered as a percentage, e.g. 90%. Cohen (1988: 56) recommends an allowable Type II error (*beta*) of about four times *alpha*. Thus, for an *alpha* of 1%, *beta* might be set at, say, 0.05 (power = 95%). Note that this module does not compute sample sizes for an *exact test* with a given power; but these can be obtained by using module P1 (Power of test for comparison of proportions) and entering different sample sizes in a series of trial-and-error estimations of expected power, until the required power is attained (Hirji *et al.* 1994).

As samples that are big enough to detect a difference may not estimate its size precisely enough to permit a decision on its practical importance (see Goodman and Berlin 1994, Bristol 1989), the program permits stipulation of **precision,** in terms of the required confidence interval for either the difference between proportions or the odds ratio, instead of power.

Optionally, the program will inflate sample sizes to compensate for the probability that not all members of the selected samples will be included in the analysis, e.g. because of failure to locate addresses, refusal to participate, or missing data. This requires entry of the expected non-inclusion rate (%). This inflation does of course NOT compensate for possible selection bias.

If the required power is specified, the program computes the required sample sizes (for a test with or without a continuity correction) and reports the expected confidence interval for the difference between proportions. It may be prudent to use the continuity-corrected sample sizes in all instances.

If a confidence interval is stipulated for the odds ratio, the program computes the sample sizes needed to meet this requirement with a 95% or 90% probability, the power of tests using these sample sizes, and the expected confidence interval for the difference between proportions. Computation may be slow if sample sizes are large; an option is supplied for aborting it.

If a confidence interval is stipulated for the difference between proportions, the program computes the required sample sizes, and reports the power of a test using these sample sizes.

Use the results with caution if samples are very small.

Precision of the test

The required precision of the test can be stipulated in two ways: by specifying the required width of the confidence interval of the odds ratio, or alternatively, by entering the required width of the confidence interval of the difference between the proportions.. In each instance, the confidence interval in question is the (100 – *alpha*)% confidence interval – e.g., the 95% confidence interval if a significance level of 5% has been entered.

The required width of the *confidence interval of the odds ratio* is specified by entering the required ratio of the odds ratio's upper confidence limit to the odds ratio's lower confidence limit . To clarify the meaning of this specified ratio (of the upper confidence limit to the lower one), the program subsequently displays the required confidence interval for the odds ratio. It also displays the equivalent confidence interval for the difference between proportions (on the assumption that the entered proportion in group B is correct). If a confidence interval is stipulated for the odds ratio, sample sizes are computed for two tolerance probabilities, the probability that confidence intervals will fall within the defined range being set at 95% and 90% in turn.

The required width of the *confidence interval of the difference between the proportions.* is specified directly – i.e., the upper confidence level of the difference minus the lower confidence level of the difference.

METHODS

All sample sizes are rounded up to the next whole number.

If the required power of the test is stipulated, a number of methods are available for the computation of required sample sizes, and their results differ (Sahai and Khurshid 1996). This program uses two methods. The first, which yields smaller sample sizes, is appropriate for tests that use no continuity correction, and the second is appropriate for tests that use a continuity correction. The computation of sample sizes without a continuity correction uses an asymptotic normal method: formula 24.77 in Zar (1998: 560), formula 20 in Sahai and Khurshid (1996). The computation with a continuity correction uses formula 3.18 in Fleiss (1981); this is formula 24.76 in Zar (1998: 560) and formula 22 in Sahai and Khurshid (1996).

The approximate *expected length of the confidence interval* L* is computed by the following formula, which is an adaptation of the formula provided by Bristol (1989):

$$L^* = 2z\sqrt{[P_a(1 - P_a) / N_a + P_b(1 - P_b) / N_b]} + 1 / N_a + 1 / N_b$$

where z = is the standard normal deviate for the *alpha*/2 level of significance
P_a and P_b = the postulated proportions in groups A and B
(if necessary, P_a is computed from P_b and the odds ratio or the ratio of proportions)
N_a and N_b = the required sizes of samples A and B

If a confidence interval is stipulated for the odds ratio, the program uses a procedure described by Satten and Kupper (1990) to compute the smallest sample sizes for which the confidence interval for the log odds ratio will not exceed a specified width, with a probability of 95% or 90%. This width is the log of the square root of the ratio (of the upper to the lower confidence limit) that was entered. The program uses an adaptation of a Microsoft Basic algorithm provided by Satten and Kupper. The sample sizes are slightly larger than those required for tests using Cornfield's method (without continuity correction) of computing confidence intervals. If proportions below 0.001 or above 0.999 are entered, the program alters them to 0.001 and 0.999 respectively.

If a confidence interval is stipulated for the difference between proportions., the method depends on the relative sizes of the samples. For equal-sized samples, the procedure is that described by Bristol (1989) and Goodman and Berlin (1994). For unequal samples, approximate sizes are computed by replacing both the probabilities required in Bristol's formula for Q by a weighted average (Pbar) of the proportions in samples A (P_a) and B (P_b):

$$Pbar := (P_a + R * P_b) / (R + 1),$$

where R is the ratio of the sample sizes (B:A). The size computed by Bristol's formula for the sample size (nL) is multiplied by

$$\sqrt{([1 + 1 / R] / 2)}$$

and taken as the size of sample B. Power is then computed for the computed sample sizes, using formulae 3.19 and 3.20 of Fleiss (1981); a continuity correction is incorporated.

If a noninclusion rate is entered, the program inflates sample sizes by multiplying the computed sample sizes by

$$1 / [1 - (R / 100)]$$

where R = noninclusion rate %
before rounding them up

S2. SAMPLE SIZES FOR TESTING EQUIVALENCE OF PROPORTIONS

This module computes sample sizes for studies that aim to compare two proportions in order to determine whether they are equivalent, that is, whether they differ by more than a negligible amount. Equivalence tests are commonly performed in clinical trials that compare a new treatment with an established standard treatment, where there may be a reason to prefer the new treatment if it is at least as effective as the standard treatment.

The bounds of "equivalence" are defined by specifying the largest difference (e.g. 0.05) to be regarded as negligible. The test's significance level and power, the ratio of sample sizes, and the known or assumed proportion are also required. Optionally, the test may be one-sided or two-sided.

One-sided tests are usually used. In planning a clinical trial that aims to determine whether a new treatment (A) can be regarded as not worse than a standard or control treatment (B), the one-sided null hypothesis of interest depends on whether the proportions being compared represent a favourable or unfavourable outcome ("successes" or "failures"). For a favourable outcome, the null hypothesis of interest is that the proportion in B is more than negligibly higher than the proportion in A (the alternative, the study hypothesis, being that it is not materially higher). For an unfavourable outcome, the null hypothesis is that the proportion in A is more than negligibly higher than the proportion in B (the alternative being that it is not materially higher). If the aim of the study is to determine whether the treatments are equivalent, *both* hypotheses are of interest. The corresponding study hypotheses (the alternatives to the null hypotheses) are (for "successes") that the proportion in B is not materially higher, and (for "failures") that the proportion in A is not materially higher. The program displays the sample sizes required for one-sided tests in both directions.

The program also computes sample sizes for two-sided tests, where the alternative to the null hypothesis is that the two proportions do not differ in either direction my more than a negligible amount. This option is available only if the samples in the two groups are to be equal in size; the same proportion must be entered for A and B.

Optionally, the program will inflate sample sizes to compensate for the probability that not all members of the selected samples will be included in the analysis, e.g. because of failure to locate addresses, refusal to participate, or missing data. This requires entry of the expected non-inclusion rate (%). This inflation does of course NOT compensate for possible selection bias.

METHOD

The program uses the maximum likelihood procedure (based on the difference between proportions) proposed by Farrington and Manning (1990; formulae in Appendix); this has been reported to be generally preferable to other methods (Roebruck and Kuhn 1995).

All sample sizes are rounded up to the next whole number. *If a noninclusion rate (R%) is entered*, the program inflates sample sizes by multiplying the computed sample sizes, before rounding them up, by $1 / [1 - (R / 100)]$.

S3. SAMPLE SIZES FOR COMPARISON OF PROPORTIONS (STRATIFIED DATA)

This module computes the sizes of the samples required to detect a true difference of a given magnitude between proportions in two stratified samples, with a given significance level and a given power or precision. It is appropriate for a Mantel-Haenszel (Mantel and Haenszel 1959) or Cochran test (Cochran 1954), and assumes that the odds ratio is the same in all strata. The data may come from a trial or observational study comparing two independent groups or samples.

The samples are labelled A and B If there is a control (comparison) group (e.g. subjects not exposed to a supposed risk factor or to an experimental treatment), it should be called sample B.

The program requires entry of the desired significance level for a two-tailed test, power, and number of strata, and the odds ratio to be detected. If a one-tailed test is wanted, *alpha* must be doubled; e.g., 10% should be entered to obtain sample sizes for a 5% significance level. Power is entered as a percentage, e.g. 90%. Cohen (1988: 56) recommends an allowable Type II error (*beta*) of about four times *alpha*. Thus, for an *alpha* of 1%, *beta* might be set at, say, 0.05 (power = 95%). The effects of changing the significance level or other parameters are easily examined

In addition, three items of information (known or assumed) are needed for each stratum: the stratum's relative size (e.g. the number of subjects, or the percentage or proportion of the total sample); the ratio (in this stratum) of the size of sample B to the size of sample A (in a case-control study, for example, this is the number of controls per case); and the proportion under study (expressed as a percentage) in sample B in this stratum (i.e., the percentage of sample B, in this stratum, who have the attribute under study).

Optionally, the program will inflate sample sizes to compensate for the probability that not all members of the selected samples will be included in the analysis, e.g. because of failure to locate addresses, refusal to participate, or missing data. This requires entry of the expected non-inclusion rate (%). This inflation does of course NOT compensate for possible selection bias.

The program computes the required sample sizes in the two groups, for a test with or without a continuity correction. It may be prudent to use the continuity-corrected (larger) sample sizes in all instances. Use the results with caution if samples are very small.

METHOD

The procedure described by Woolson, Bean, and Rojas (1986) is used;. The numbers of members of the two groups (e.g. cases and controls in a case-control study) in each stratum are regarded as fixed.

All sample sizes are rounded up to the next whole number. *If a noninclusion rate (R%) is entered* , the program inflates sample sizes by multiplying the computed sample sizes, before rounding them up, by

$$1 / [1 - (R / 100)]$$

S4. SAMPLE SIZES FOR COMPARISON OF PROPORTIONS (MULTIPLE LOGISTIC REGRESSION)

This module computes sample sizes for comparisons of proportions in two samples using a logistic regression analysis.

The program requires entry of the information required for computing sample sizes for a simple comparison of proportions, and of the multiple correlation coefficient (*R*) relating the variable (the one to be compared) with all other covariates in the model. This coefficient can be provided by statistical programs for multiple regression analysis.

As well as *R*, the desired significance level (*alpha*) for a two-sided test, power (entered as a percentage, e.g. 90%.), the ratio of the sizes of the two samples, the known or expected proportion in Sample B, and the magnitude of the difference to be detected must be entered. If a one-tailed test is wanted, *alpha* must be doubled; e.g., 10% should be entered to obtain sample sizes for a 5% significance level. The size of the difference to be detected can be indicated by entering an odds ratio, the ratio of the proportions, or the proportion in sample A. The effects of changing the significance level or other parameters are easily examined.

Optionally, the program will inflate sample sizes to compensate for the probability that not all members of the selected samples will be included in the analysis, e.g. because of failure to locate addresses, refusal to participate, or missing data. This requires entry of the expected non-inclusion rate (%). This inflation does of course NOT compensate for possible selection bias.

The program computes the required sample sizes, and estimates an approximate confidence interval for the difference between proportions. It also reports the maximum number of covariates that can be safely handled with a sample of the computed size.

METHODS

Sample sizes for a simple comparison are first calculated, using an asymptotic normal method without a continuity correction: formula 24.77 in Zar (1998: 560), formula 20 in Sahai and Khurshid (1996). These sample sizes are then multiplied by a variance inflation factor (VIF) calculated from the multiple correlation coefficient (R). The procedure is described by Hsieh et al. (1998). The formula is

$$\text{VIF} = 1 / (1 - R^2).$$

The "10 events per variable" rule-of-thumb (Rothman and Greenland 1998, p. 406; Peduzzi *et al*. 1996) is used to estimate the maximum number of covariates that can be safely handled with a sample of the computed size. The expected numbers of events in the two samples are computed, rounded off downwards, and summed. The expected number of non-events is computed in the same way, and the smaller of the two sums is divided by 10 to provide the maximum number of covariates.

The approximate *expected length of the confidence interval* L* is computed by the following formula, which is an adaptation of the formula provided by Bristol (1989):

$$L^* = 2z\sqrt{[P_a(1 - P_a) / N_a + P_b(1 - P_b) / N_b] + 1 / N_a + 1 / N_b}$$

where z = is the standard normal deviate for the *alpha*/2 level of significance

P_a and P_b = the postulated proportions in groups A and B

(if necessary, P_a is computed from P_b and the odds ratio or the ratio of proportions)

N_a and N_b = the required sizes of samples A and B.

The confidence interval in question is the (100 – *alpha*)% confidence interval – e.g., the 95% confidence interval if a significance level of 5% has been entered.

All sample sizes are rounded up to the next whole number. *If a noninclusion rate (R%) is entered*, the program inflates sample sizes by multiplying the computed sample sizes, before rounding them up, by

$$1 / [1 - (R / 100)] .$$

S5. SAMPLE SIZES FOR COMPARISON OF DISTRIBUTIONS IN ORDERED CATEGORIES

This module computes the sizes of the samples required to detect a true difference of a given magnitude between two independent samples, with a given significance level and a given power, with respect to the distribution of an attribute that has ordered categories, using a Mann-Whitney (Wilcoxon rank sum) test (Mann and Whitney 1967; Wilcoxon 1945).. The test might be used (for example) to compare levels of exposure to a supposed causal factor (e.g. heavy smokers, light smokers, nonsmokers) in a case-control study, or levels of outcome (e.g. severe illness, moderate illness, mild illness or no illness) in a cohort study or trial.

A proportional odds model is employed. That is, it is assumed that when the 2 x *k* table that displays the presence of the attribute in a set of *k* ordered categories is converted to a 2 x 2 table by combining adjacent categories, the odds ratio is the same, whatever cutting-point is used.

The samples are labelled A and B If there is a control (comparison) group (non-cases in a case-control study, or subjects not exposed to a supposed risk or protective factor, or to an experimental treatment in a trial), it should be called sample B.

The program requires entry of the desired significance level for a two-tailed test, power, the ratio of sample sizes, the number of categories, the known or expected relative size of the categories in group B (e.g., numbers of subjects, or percentages or proportions of all subjects in group B), and either the odds ratio to be detected, or the proportion of group A falling into the first category. If a one-tailed test is wanted, *alpha* must be doubled; e.g., 10% should be entered to obtain sample sizes for a 5% significance level. Power is entered as a percentage, e.g. 90%. The effects of changing the significance level or other parameters are easily examined

Optionally, the program will inflate sample sizes to compensate for the probability that not all members of the selected samples will be included in the analysis, e.g. because of failure to locate addresses, refusal to participate, or missing data. This requires entry of the expected non-inclusion rate (%). This inflation does of course NOT compensate for possible selection bias.

The program displays the required sample sizes in both groups.. The procedure is based on a normal approximation, and is accurate only if it generates moderate to large sample sizes.

METHODS

The formula for computing sample sizes for a Mann-Whitney test (allowing for ties) is derived from formula 10 of Whitehead (1993).

All sample sizes are rounded up to the next whole number. *If a noninclusion rate (R%) is entered* , the program inflates sample sizes by multiplying the computed sample sizes, before rounding them up, by

$$1 / [1 - (R / 100)] .$$

S6. SAMPLE SIZES FOR COMPARISON OF MEANS

This module computes the sample sizes required to detect a difference of a given magnitude between the means in two independent samples, with a given power or precision.

The desired significance level (*alpha*) for a two-sided test, the ratio of the sizes of the samples, either the pooled variance (known or assumed) or the standard deviations (known or assumed) in the samples, and the difference to be detected must be entered. If a one-tailed test is wanted, *alpha* must be doubled; e.g., 10% should be entered to obtain sample sizes for a 5% significance. The effects of changing the significance level, sample sizes or other parameters are easily examined..

To stipulate *power*, it is entered as a percentage, e.g. 90%. Cohen (1988: 56) recommends an allowable Type II error (*beta*) of about four times *alpha*. Thus, for an *alpha* of 1%, *beta* might be set at, say, 0.05 (power = 95%).

As samples that are big enough to detect a difference may not estimate its size precisely enough to permit a decision on its practical importance (see Goodman and Berlin 1994, Bristol 1989), the program permits stipulation of **precision,** in terms of the required width of the confidence interval for the difference between means, instead of power.

Optionally, the program will inflate sample sizes to compensate for the probability that not all members of the selected samples will be included in the analysis, e.g. because of failure to locate addresses, refusal to participate, or missing data. This requires entry of the expected non-inclusion rate (%). This inflation does of course NOT compensate for possible selection bias.

If the required power is specified, the program computes the required sample sizes and reports the expected confidence interval for the difference between means.

If a confidence interval is stipulated for the difference between means, the program computes the required sample sizes, and reports the power of a test using these sample sizes.

METHODS

Sample sizes for a *t*-test are computed by formula 6 of Lachin (1981). The computed total size N is multiplied by a correction factor, (N + 1) / (N - 1). The computation of the approximate expected width of the confidence interval is explained by Goodman and Berlin (1994: Appendix). If the pooled variance V is not entered it is computed as follows:

$$V = S_A^2 Q_A + S_B^2 Q_B$$

where S_A and S_B = standard deviations of Samples A and B respectively
Q_A and Q_B -= proportions of subjects in Samples A and B respectively

If he width of the required confidence interval is entered, sample sizes are computed by the formula (for nL) provided by Bristol (1989). Power is then computed for the new sample sizes, using formula 7 of Lachin (1981); a continuity correction is incorporated.

All sample sizes are rounded up to the next whole number. If a noninclusion rate (R%) is entered , the program inflates sample sizes by multiplying the computed sample sizes, before rounding them up, by 1 / [1 - (R / 100)] .

S7. SAMPLE SIZES FOR TESTING EQUIVALENCE OF MEANS

This module computes approximate sample sizes for tests that compare two means in order to determine whether they differ by more than a negligible amount. These tests are commonly used to determine whether two treatments can be regarded as having an equal effect (tests of average bioequivalence). Sample sizes are computed for a two-sided test, based on the two one-sided tests (confidence interval) approach (Schuirmann 1987).

The bounds of "equivalence" are defined by specifying the largest difference to be regarded as negligible. Other required entries are the significance level for the two-sided test, its power, the ratio of sample sizes, and either the pooled variance (known or assumed) or the standard deviations (known or assumed) in the two samples.. Optionally, the expected means may be entered; if they are not entered they are assumed to be equal; this will provide minimal sample sizes for the equivalence tests – if the means differ, an equivalence test requires larger samples.

Optionally, the program will inflate sample sizes to compensate for the probability that not all members of the selected samples will be included in the analysis, e.g. because of failure to locate addresses, refusal to participate, or missing data. This requires entry of the expected non-inclusion rate (%). This inflation does of course NOT compensate for possible selection bias.

The effects of changing the significance level, power, or other parameters are easily examined..

METHOD

Sample sizes are computed by Westlake's procedure (Westlake 1973, formulae 1 and 2); the initial computation uses the normal distribution (Kim 1997), and the result is then modified by iterations that use the t distribution, as suggested by Westlake. Approximations are used in the computation if sample sizes are unequal or if the means in the two samples are known or assumed to differ; the sample sizes tend to be slightly higher than those computed by the PASS 2000 power-and-sample-size program of NCSS Statistical Software.

If the pooled variance V is not entered it is computed as follows:

$$V = S_A^2 Q_A + S_B^2 Q_B$$

where S_A and S_B = standard deviations of Samples A and B respectively
Q_A and Q_B -= proportions of subjects in Samples A and B respectively

---

S8. SAMPLE SIZES FOR COMPARISON OF MEANS (MULTIPLE LINEAR REGRESSION)

This module computes sample sizes for comparisons of means in two samples using a multiple linear regression analysis.

The program requires entry of the information required for computing sample sizes for a simple comparison of means, proportions, and of the multiple correlation coefficient (R) relating the variable (the one to be compared) with all other covariates in the model. This coefficient can be provided by statistical programs for multiple regression analysis.

As well as R, the desired significance level (*alpha*) for a two-sided test, power (entered as a percentage, e.g. 90%.), the ratio of the sizes of the two samples, either the pooled variance (known or expected) or the standard deviations (known or expected) in the two samples and the difference to be detected must be entered. If a one-tailed test is wanted, *alpha* must be doubled; e.g.,10% should be entered to obtain sample sizes for a 5% significance level

Optionally, the program will inflate sample sizes to compensate for the probability that not all members of the selected samples will be included in the analysis, e.g. because of failure to locate addresses, refusal to participate, or missing data. This requires entry of the expected non-inclusion rate (%). This inflation does of course NOT compensate for possible selection bias.

The program computes the required sample sizes and reports the approximate confidence interval expected for the difference between means.

The effects of changing the significance level or other parameters are easily examined.

METHOD

Sample sizes are first calculated for a simple comparison, using formula 6 of Lachin (1981). The computed total sample size N is multiplied by a correction factor, $(N + 1) / (N - 1)$; this has a negligible effect on large samples. If the pooled variance V is not entered it is computed as follows:

$$V = S_A^2 Q_A + S_B^2 Q_B$$

where S_A and S_B = standard deviations of Samples A and B respectively
Q_A and Q_B -= proportions of subjects in Samples A and B respectively

These sample sizes are then multiplied by a variance inflation factor (VIF) calculated from the multiple correlation coefficient (R). The procedure is described by Hsieh et al. (1998). The formula is

$$VIF = 1 / (1 - R^2).$$

The computation of the approximate confidence interval is explained by Goodman and Berlin (1994:Appendix).

All sample sizes are rounded up to the next whole number. *If a noninclusion rate (R%) is entered* , the program inflates sample sizes by multiplying the computed sample sizes, before rounding them up, by $1 / [1 - (R / 100)]$.

S9. SAMPLE SIZES FOR COMPARISON OF NUMBERS OF EVENTS (E.G. DISEASE ONSETS/SPELLS)

This module computes sample sizes for comparisons of the rates of occurrence of events that are assumed to have a Poisson distribution. It may be helpful in the planning of cohort studies or trials that compare the occurrence of new cases of a rare disease, or numbers of disease episodes. in two groups.

The desired significance level (*alpha*) and power (entered as a percentage), the ratio of the sizes of the two samples and the ratio of event rates that it is wished to detect must be entered. Optionally, the known or assumed rate of events in one of the samples can also be entered; the required sample sizes will then be computed in terms of person-time units as well as in terms of numbers of events.

The statistical test can be regarded as a test of a two-sided hypothesis, although it is based on one tail of the chi-square or Poisson distribution. For a very approximate indication of the sample sizes required for a one-tailed test, the required significance level can be doubled (e.g. by entering 10% for a one-tailed test at 5%).

Optionally, the program will inflate sample sizes to compensate for the probability that not all members of the selected samples will be included in the analysis, e.g. because of failure to locate addresses, refusal to participate, or missing data. This requires entry of the expected non-inclusion rate (%). This inflation does of course NOT compensate for possible selection bias.

If the rate in one sample is not entered, the program provides an estimate of the number of events required in each sample, based on a normal approximation to the Poisson distribution. .

If the rate in one sample is entered, the program provides two estimates of the number of events required in each sample; both estimates are approximations, and are generally not identical.. The number of person-time units required in each sample is also reported.

METHODS

The method based on a normal approximation to the Poisson distribution, which uses a chi-square test, is described by Breslow and Day (1987: formula 7.3 and 7.4) and by Lui (2000, formula 11); a continuity correction is applied.

If the assumed rate of events is entered, the computation is based on the test described by Shiue and Bain (1982), unless the samples are unequal and the larger sample is assumed to have the higher rate, when the computation is based on the test described by Thode (1997), which is more powerful. The basic formula is Thode's formula 2, from which the sample sizes for both tests are derived.

All sample sizes are rounded up to the next whole number. *If a noninclusion rate (R%) is entered* , the program inflates sample sizes by multiplying the computed sample sizes, before rounding them up, by $1 / [1 - (R / 100)]$.

S10. SAMPLE SIZES FOR COMPARISON OF SURVIVAL (TIME TO EVENT)

This module computes sample sizes for comparisons of survival time in two groups, e.g. in clinical trials and cohort studies. (Survival time, or "time to event", refers to the time-lapse until the occurrence of a specified end-point event, such as death or the onset of a disease.)

Sample sizes are computed for two tests: the logrank test and a score test based on the log hazard ratio The results, which are approximations, are valid only if the hazard ratio is constant over time; an exponential curve is assumed.

The program requires entry of the desired significance level (*alpha*) for a two-sided test, power, and the ratio of the sizes of the two samples. If a one-tailed test is wanted, *alpha* must be doubled; e.g.,10% should be entered to obtain sample sizes for a 5% significance level. Power is entered as a percentage, e.g. 90%. Cohen (1988: 56) recommends an allowable Type II error (*beta*) of about four times *alpha*. Thus, for an *alpha* of 1%, *beta* might be set at, say, 0.05 (power = 95%). The effects of changing the significance level or other parameters are easily examined

To obtain only the required numbers of events (and not of subjects), the hazard ratio that it is wished to detect must be entered..

To obtain the required numbers both of events and of subjects, there are four options:

(a) enter the hazard ratio to be detected, and an estimate of the average probability of survival to the end of the follow-up period (a weighted average of the probabilities in the two groups);
(b) enter the known or assumed probability of survival to the end of the follow-up period, in each group;
(c) enter the known or assumed median survival rates in each group, and the study's follow-up period;
(d) enter the known or assumed median survival rates in each group, the accrual period (the time from the study's start to the entry of the last subject), and the follow-up period of the last subject.

The first three of these options are appropriate for a study in which all subjects are enrolled at the same time and followed up for the same period, and the last option is appropriate for a study in which the entry of subjects is staggered over a period.

Optionally, the program will inflate sample sizes to compensate for the probability that not all members of the selected samples will be included in the analysis, e.g. because of failure to locate addresses, refusal to participate, or missing data. This requires entry of the expected non-inclusion rate (%). This inflation does of course NOT compensate for possible selection bias.

The program reports the total sample size required and the sample sizes required in each group, for both the log-rank test and the score test. The hazard ratio and probabilities of survival (if not entered) may also be reported.

METHODS

The method for the log-rank test is described by Freedman (1982) ,and the method for the score test by Schoenfeld (1983). The total numbers of *events* required for the two tests are computed by formula 4 of Freedman (1982) and formula 1 of Schoenfeld (1983), respectively. The hazard ratio required by these formulae is calculated (if it is not entered) as the ratio of the natural logs of the survival probabilities in the two groups or, if survival probabilities are not entered, as the ratio of the hazard functions H, calculated for each group as

$$H = -\ln(0.5) / (\text{median survival time}).$$

For each test, the total number of subjects required is then computed by dividing the total number of events by the probability of an individual having an event in the study population, which is the complement of the average probability of survival. If the latter is not entered, the probability of an event is calculated from the probabilities in the two groups (Schoenfeld 1983, p. 500); the relative sample sizes are used as weights. If these probabilities are not entered, but median survival times and the follow-period are, the probabilities are calculated as

$$1 – \exp[-(\text{hazard ratio}) \bullet (\text{follow-up period}).]$$

For a study whose subjects are accrued over a period, the probability of an event is computed by Simpson's rule, as explained by Liu (2000: 787); the survival probabilities used for this purpose are averages of the survival probabilities in the two groups, weighted by the relative sample sizes; these survival probabilities S at time t (S_t) are estimated by the formula (Armitage e*t al*. 2002: formula 17.20)

$$S_t = \exp(-H_t).$$

where H_t = hazard function at time t.

All sample sizes are rounded up to the next whole number. *If a noninclusion rate (R%) is entered* , the program inflates sample sizes by multiplying the computed sample sizes, before rounding them up, by $1 / [1 - (R / 100)]$.

S11. SAMPLE SIZES FOR STUDY OF CHANGE (USING BEFORE-AFTER ORDINAL-SCALE RATINGS)

This module computes sample sizes for tests that compare the changes in two independent groups followed up in a cohort study or trial, where the changes are based on a comparison of paired "before" and "after" ratings that use an ordinal scale, such as "never"–"once a day"–"2 or 3 times a day"–"more than 3 times a day".

The samples are labelled A and B If there is a control (comparison) group (e.g. subjects not exposed to a supposed risk factor or to an experimental treatment), it should be called group B.

One direction of change (e.g. "less frequent") must be arbitrarily designated as the "right" direction of change. This should be the direction of change that is more probable in group A (e..g., "less often" or "less pain").

The program requires entry of the desired significance level (*alpha*) for a two-sided test, and power. If a one-tailed test is wanted, *alpha* must be doubled; e.g.,10% should be entered to obtain sample sizes for a 5% significance level. Power is entered as a percentage, e.g. 90%. Cohen (1988: 56) recommends an allowable Type II error (*beta*) of about four times *alpha*. Thus, for an *alpha* of 1%, *beta* might be set at, say, 0.05 (power = 95%).

In addition. it is necessary to enter four probabilities: the probability (known or assumed) of a changed rating (in either direction) in each group; the probability (known or assumed) of a change in the right direction in group B, and the probability of changes in the right direction that it is desired to detect in group A (this cannot be less than the probability entered for group B).

Optionally, the program will inflate sample sizes to compensate for the probability that not all members of the selected samples will be included in the analysis, e.g. because of failure to locate addresses, refusal to participate, or missing data. This requires entry of the expected non-inclusion rate (%). This inflation does of course NOT compensate for possible selection bias.

The program uses a procedure described by Strickland and Lu (2003), which is based on a comparison of the odds ratios (in the two groups) in favour of change in the right direction. These odds ratios, which are estimated from the above entries, are displayed together with the required sample sizes.

Occasional problems occur with the procedure. Computation is not possible if one of the groups has no changes in the "wrong" direction; this can be overcome by adding a fictitious observation showing a change in the "wrong" direction . Very high sample sizes may be reported if the ratio of odds ratios is very large. As a precaution, if the ratio of odds ratios is over 20 the program checks whether the computed sample sizes increase when the ratio is reduced by 10%; if they do not, a warning is shown, saying that the sample sizes may be exaggerated.

METHODS

The program uses the procedure described by Strickland and Lu (2003) in section 3.2 ("ordinal response - adjacent category odds") of their paper. The sample sizes required for a test using the adjacent-category-odds model are larger than those required for a test using the proportional-odds model. The working formula, which is equivalent to formula 3 in the paper, is:

$$N = [(Z_a + Z_b) / delta]^2 * (X + Y)$$

where

N = required sample size in each group

Z_a = z(1 - *alph*a)

Z_b = z(1 - *beta*)

delta = $\ln(OR_1 / OR_2)$;

$X = [T_1 / (T_1)^2 + (1 - T_1) / (1 - T_1)^2] / Pr_A$

$Y = [T_2 / (T_2)^2 + (1 - T_2) / (1 - T_2)^2] / Pr_B$

$T_1 = Pr_A / Pc_A$

$T_2 = Pr_B / Pc_B$

Pc_A = probability of a changed rating in group A

Pc_B = probability of a changed rating in group B

Pr_A = probability of a change in the right direction in group A

Pr_B = probability of a change in the right direction in group B

The odds ratios in favour of change in the right direction are

$OR_1 = T_1 / (1 - T_1)$ [in group A] and

$OR_2 = T_2 / (1 - T_2)$ [in group B].

If the ratio of odds ratios is over 20 the program checks whether the computed sample sizes increase when the ratio of odds ratios is reduced by 10% (and *delta* is modified accordingly before insertion in the above formula).

All sample sizes are rounded up to the next whole number. *If a noninclusion rate (R%) is entered*, the program inflates sample sizes by multiplying the computed sample sizes, before rounding them up, by $1 / [1 - (R / 100)]$.

REFERENCES

Abramson JH, Gahlinger PM (2001) Computer programs for epidemiologists: PEPI version 4. Sagebrush Press: Salt Lake City.

Abramson JH, Peritz E (1983) Calculator programs for the health sciences. New York: Oxford University Press.

Agresti A (1980) Generalized odds ratios for ordinal data. Biometrics 36: 59-67.

Agresti A (1990) Categorical data analysis. New York: John Wiley & Sons.

Agresti A (1996) An Introduction to categorical data analysis. New York: John Wiley & Sons.

Ahn C, Odom-Maryon T (1995) Estimation of a common odds ratio under binary cluster sampling. Statistics in Medicine 14: 1567-1577.

Altman DG (1991) Practical statistics for medical research. London: Chapman and Hall.

Altman DG (1998) Confidence intervals for the number needed to treat. British Medical Journal 317 :1309-1312

Altman DG, Andersen PK (1999) Calculating the number needed to treat for trials where the outcome is time to an event. British Medical Journal 319: 1492-1495

Armitage P, Berry G, Matthews JNS (2002) Statistical methods in medical research, 4th edn. Oxford: Blackwell Science.

Barron BA (1977) The effects of misclassification on the estimation of relative risk. Biometrics 33:414-418.

Begg CB, Mazumdar M (1994) Operating characteristics of a rank correlation test for publication bias. Biometrics 50: 1108-1101.

Bennett, BM (1981) On the use of the negative binomial in epidemiology. Biometrical Journal 23: 69-72.

Bennett BM, Hsu P (1960) On the power function of the exact test for the 2x2 contingency table. Biometrika 147:393-398.

Berry G, Kitchin RM, Mock PA (1991) A comparison of two simple hazard ratio estimators based on the logrank test. Statistics in Medicine 10:749-755.

Bjerre LM, LeLorier J (2000) Expressing the magnitude of adverse effects in case-control studies: “the number of patients needed to be treated for one additional patient to be harmed”. British Medical Journal 320: 503-506.

Blalock HM Jr (1979) Social statistics, revised 2nd edn. New York: McGraw-Hill.

Breslow NE (1984) Elementary methods of cohort analysis. International Journal of Epidemiology 13: 112-115.

Breslow NE, Day NE (1980) Statistical methods in cancer research. vol. I. The analysis of case-control studies. Lyon: International Agency for Research on Cancer.

Breslow NE, Day NE (1987) Statistical methods in cancer research, vol. II. The design and analysis of cohort studies. Lyon: International Agency for Research on Cancer.

Bristol DR (1989) Sample sizes for constructing confidence intervals and testing hypotheses. Statistics in Medicine 6:803-811.

Burr EJ (1964) Small-sample distributions of the two-sample Cramer-von Mises' W-square and Watson's U-square, Annals of Mathematical Statistics 35: 1091-98.

Campbell MJ, Gardner MJ (1988) Calculating confidence intervals for some non-parametric analyses. British Medical Journal 296 :1454-1456.

Canner PL (1987) An overview of six clinical trials of aspirin in coronary heart disease. Statistics in Medicine 6:255-263.

Casagrande JT, Pike MC, Smith PG (1978a) The power function of the 'exact' test for comparing two binomial distributions. Applied Statistics 27:176-180.

Casagrande JT, Pike MC, Smith PG (1978b) Algorithm AS 129: The power function of the 'exact' test for comparing two binomial distributions. Applied Statistics 27:212-219.

Chatellier G, Zapletal E, Lemaitre D, Menard J, Degoulet P (1996) The number needed to treat: a clinically useful nomogram in its proper context. British Medical Journal 312: 426-429.

Cochran WG (1954) Some methods for strengthening the common chi- square tests. Biometrics 10: 417-451.

Cohen J (1988) Statistical power analysis for the behavioral sciences. 2nd edn. New York: Academic Press.

Conover WJ (1980) Practical nonparametric statistics., 2nd edn. New York: John Wiley & Sons.

Cornfield J (1956) A statistical problem arising from retrospective studies. In Proceedings of the 3rd Berkeley symposium on mathematical statistics and probability (Newman J. ed), pp 135-148. Berkeley: University of California Press.

Cox DR, Oakes D (1984) Analysis of survival data. London: Chapman & Hall.

D'Agostino RB (1990) Comment. Statistics in Medicine 9: 377-378.

Daly LE (1998) Confidence limits made easy: interval estimation using a substitution method. American Journal of Epidemiology 147 :783-790.

DeMets DL (1987) Methods for combining randomized clinical trials: strengths and limitations. Statistics in Medicine 6:341-348.

DerSimonian R, Laird N (1986) Meta-analysis in clinical trials. Controlled Clinical Trials 7:177-188.

Donald A, Donner A (1987) Adjustments to the Mantel-Haenszel chi-square statistic and odds ratio variance estimator when the data are clustered. Statistics in Medicine 6: 491-499.

Donner A, Eliasziw M, Klar N (1994) A comparison of methods for testing homogeneity of proportions in teratologic studies. Statistics in Medicine 13: 1253-1264.

Dupont WD (1986) Sensitivity of Fisher's exact test to minor perturbations in 2x2 contingency tables. Statistics in Medicine 5: 629-635.

Ederer F, Mantel M (1974) Confidence limits on the ratio of two Poisson variables. American Journal of Epidemiology 100:165-167.

Egger M, Smith GD, Schneider M, Minder C (1997) Bias in meta-analysis detected by a simple graphical test. British Medical Journal 315: 629-634.

Everitt BS (1977) The analysis of contingency tables. London: Chapman and Hall.

Farrington CP, Manning G (1990) Test statistics and sample size formulae for comparative binomial trials with null hypothesis of non-zero risk difference or nun-unity relative risk. Statistics in Medicine 9:1457-1494.

Feinstein AR (1995) Meta-analysis: statistical alchemy for the 21st century. Journal of Clinical Epidemiology 48: 71-79.

Fisher RA (1948) Combining independent tests of significance. American Statistician 2:30.

Fleiss J (1979) Confidence intervals for the odds ratio in case-control studies: the state of the art. Journal of Chronic Diseases 32:69-77.

Fleiss JL (1981) Statistical methods for rates and proportions, 2nd edn. New York: John Wiley & Sons.

Fleiss JL, Gross AJ (1991) Meta-analysis in epidemiology, with special reference to studies of the association between exposure to environmental tobacco smoke and lung cancer: a critique. Journal of Clinical Epidemiology 44: 127-139.

Fleiss JL, Levin B, Paik MC (2003) Statistical methods for rates and proportions, 3rd edn. Hoboken, NewJersey: John Wiley & Sons.

Freedman LS (1982) Tables of the number of patients required in clinical trials using the logrank test. Statistics in Medicine 1: 121-129.

Gabriel KR (1966) Simultaneous test procedures for multiple comparisons on categorical data. Journal of the American Statistical Association 61:1081-1096.

Gahlinger PM, Abramson JH (1993) Computer programs for epidemiological analysis. Honolulu, Hawaii: Makapuu Medical Press.

George EO (1977) Combining independent one-sided and two-sided statistical tests - Some theory and applications. Doctoral dissertation, University of Rochester.

Glass GV, McGaw B, Smith ML (1981). Meta-analysis in social research. Beverly Hills: Sage Publications.

Golbeck AL (1994) A note on partitioning 2 x C contingency tables into non-independent 2 x 2 tables. Biometrics 50:305-307.

Goodman SN, Berlin JA (1994) The use of predicted confidence intervals when planning experiments and the misuse of power when interpreting results. Annals of Internal Medicine 121:200-206, appendix.

Graham PL, Mengersen K, Morton AP (2003) Confidence limits for the ratio of two rates based on likelihood scores: non-iterative method. Statistics in Medicine 22: 2071-2083.

Greenland S (1987) Variance estimators for attributable fraction estimates consistent in both large strata and sparse data. Statistics in Medicine 6: 701-708.

Greenland S (1999) Re: "Confidence limits made easy: interval estimation using a substitution method". American Journal of Epidemiology 149: 884.

Greenland S, Kleinbaum DG (1983) Correcting for misclassification in two-way tables and matched-pair studies. International Journal of Epidemiology 12:93-97.

Greenland S, Robins JM (1985) Estimation of a common effect parameter from sparse follow-up data. Biometrics 41:55-68.

Guess HA, Lydick EV, Small RD, Miller LP (1987) Epidemiological programs for computers and calculators: Exact binomial confidence intervals for the relative risk in follow-up studies with sparsely stratified incidence density data. American Journal of Epidemiology 125:340-347

Haber M (1980) A comparison of some continuity corrections for the chi-square test on 2 x 2 tables. Journal of the American Statistical Association 75: 510-515.

Halperin M, Gilbert PR, Lachin JM (1987) Distribution-free confidence intervals for Pr(X1<X2). Biometrics 43: 71-80.

Hedges LV, Olkin I (1985) Statistical methods for meta-analysis. Orlando: Academic Press.

Higgins JPT, Thompson SG (2002) Quantifying heterogeneity in a meta-analysis. Statistics in Medicine 21: 1539-1558.

Hirji KF, Tang M-L, Vollset SE, Elashoff RM (1994) Efficient power computation for exact and mid-P tests for the common odds ratio in several 2 x 2 tables. Statistics in Medicine 13: 1539-1549.

Hollander M, Wolfe DA (1999) Nonparametric statistical methods, 2nd edn. New York: John Wiley & Sons.

Hsieh FY, Bloch DA, Larsen MD (1998). A simple method of sample size calculation for linear and logistic regression. Statistics in Medicine 17: 1623-1634.

Hutcheson K (1970) A test for comparing diversities based on the Shannon formula. Journal of Theoretical Biology 29: 151-154.

Iyengar S, Greenhouse JB (1988) Selection models and the file drawer problem. Statistical Science 3: 109-117.

Jacobson PE Jr (1976) Introduction to statistical measures for the social and behavioral sciences, Hinsdale, Ill.: Dryden Press, pp 430-434.

Jewell NP (1986) On the bias of commonly used measures of association for 2x2 tables. Biometrics 42: 351-358.

Kaplan EL, Meier P (1958) Nonparametric estimation from incomplete observations. Journal of the American Statistical Association 53: 457-481.

Kim J S (1997) Determining sample size for testing equivalence. Medical Device and Diagnostic Industry Magazine. Available on the Internet at www.devicelink.com/archive/97/020.html

Kleinbaum DG, Kupper LL, Morgenstern H (1982**)** Epidemiological research: principles and quantitative methods. New York: Van Nostrand Reinhold

Lachin JM (1981) Introduction to sample size determination and power analysis for clinical trials. Controlled Clinical Trials 2: 93-113.

Liu G (2000) Sample size for epidemiologic studies. In: Gail MH, Benixhou J (eds.) Encyclopedia of epidemiologic methods, Chichester: John Wiley and Sons, pp. 777-794.

Liu I-M, Agresti A (1996) Mantel-Haenszel-type inference for cumulative odds ratios with a stratified ordinal response. Biometrics 52:1223-1234York

Lui K-J, personal communication.

Lui K-J (2004) Statistical evaluation of epidemiological risk. Chichester: John Wiley & Sons.

Machin D, Gardner MJ (1988) Calculating confidence intervals for survival time analyses. British Medical Journal 296:1369-1371.

Machin D, Gardner MJ (2000) Time to event studies. In: Altman DG, Machin D, Bryant TN, Gardner MJ, eds (2000) Statistics with confidence, 2nd edn. BMJ Books, pp 93-194.

Mann HB, Whitney DR (1947) On a test of whether one of two random variables is stochastically larger than the other. Annals of Mathematical Statistics 18: 50-60.

Mantel N (1963) Chi-square tests with one degree of freedom; extensions of the Mantel-Haenszel procedure. American Statistical Association Journal 58:690-700.

Mantel N (1990) Comment. Statistics in Medicine 9: 369-370.

Mantel N (1992) An invalid comparison of several point estimators of the odds ratio in a single 2x2 contingency table. Biometrics 48:1289-1295.

Mantel N, Haenszel W (1959) Statistical aspects of the analysis of data from retrospective studies of disease. Journal of the National Cancer Institute 22:719-748.

Martin D, Austin H (1991) An efficient program for computing conditional maximum likelihood estimates and exact confidence limits for a common odds ratio. Epidemiology 2: 359-362.

Martin DO, Austin H (1996) Exact estimates for a rate ratio. Epidemiology 7: 29-33.

Maxwell AE (1961) Analysing qualitative data. London: Methuen.

Mehta CR, Patel NR, Gray R (1985) Computing an exact confidence interval for the common odds ratio in several 2 x 2 contingency tables. Journal of the American Statistical Association 80: 969-973.

Miettinen OS (1972) Standardization of risk ratios. American Journal of Epidemiology 96: 283-388.

Miettinen OS, Nurminen M (1985) Comparative analysis of two rates. Statistics in Medicine 4:213-226.

Morris JA, Gardner MJ (2000) Epidemiological studies. In: Altman DG, Machin D, Bryant TN, Gardner MJ. Statistics with confidence, 2nd edn. BMJ Books.

Morrison AS (1979) Sequential pathogenic components rates. American Journal of Epidemiology 108: 709-718.

Mosteller FM, Bush RR (1954) Selected quantitative techniques. In: Lindzey G (ed.), Handbook of social psychology, Vol. 1. Theory and method. Cambridge, Mass: Addison-Wesley, pp 289-334.

Mudholkar GS, George EO (1979) The logit method for combining probabilities. In: Rustagi J (ed.). Symposium on optimizing methods in statistics. New York: Academic Press, pp. 345-366

Newcombe RG (1998) Interval estimation for the difference between independent proportions: comparison of eleven methods. Statistics in Medicine 17: 873-890.

Newcombe RG. Altman DG (2000) Proportions and their differences. In: Altman DG, Machin D, Bryant TN, Gardner MJ. Statistics with confidence, 2nd edn. BMJ Books.

Nieto FJ, Coresh J (1996) Adjusting survival curves for confounders: a review and a new method. American Journal of Epidemiology 13: 1059-1067.

Oldham PD (1968) Measurement in medicine: The interpretation of numerical data. Philadelphia: Lippincott.

Orwin R (1983) A fail-safe N for effect size in meta-analyses. Journal of Educational Statistics 8: 157-159.

Overall JE (1990) Comment. Statistics in Medicine 9: 379-82.

Peduzzi P, Concato J, Kemper E, Holford TR, Feinstein AR (1996) A simulation study of the number of events per variable in logistic regression analysis. Journal of Clinical Epidemiology 49:1373-1379.

Petitti DB (1994) Meta-analysis, decision analysis, and cost-effectiveness analysis. New York: Oxford University Press.

Peto R, Pike MC, Armitage P, Breslow NE, Cox DR, Howard SV, Mantel N. McPherson K, Peto J, Smith PG (1977) Design and analysis of randomized clinical trials requiring prolonged observation of each patient. II. Analysis and examples. British Journal of Cancer 35: 1-39.

Pike MC (1972) Contribution to the discussion on the paper by Peto R and Peto J: Asymptotically efficient rank invariant test procedures. Journal of the Royal Statistical Society, Series A, 135:201-203.

Press WH, Flannery BP, Teukolsky SA, Vetterling WT (1989) Numerical recipes in Pascal: The art of scientific computing. Cambridge: Cambridge University Press.

Quan H, Zhang J (2003) Estimate of standard deviation for a log-transformed variable using arithmetic means and standard deviations. Statistics in Medicine 22: 2723-2736.

Rao JNK, Scott AJ (1992) A simple method for the analysis of clustered binary data. Biometrics 48: 527-585.

Robins J, Breslow N, Greenland S (1986) Estimators of the Mantel-Haenszel variance consistent in both sparse data and large-strata limiting models. Biometrics 42: 311-23.

Rockhill B, Newman B, Weinbert C (1998) Use and misuse of population attributable fractions. American Journal of Public Health 88: 15-19.

Roebruck P, Kuhn A (1995) Comparison of tests and sample size formulae for proving therapeutic equivalence based on the difference of binomial probabilities. Statistics in Medicine 14:1583-1594.

Rosenthal R (1979) The 'file drawer problem' and tolerance for null results. Psychological Bulletin 3:638-641.

Rosenthal R, Rubin DB (1988) Comment: assumptions and procedures in the file drawer problem. Statistical Science 3:120-125.

Rothman KJ (1986) Modern epidemiology. Boston: Little, Brown & Co.

Rothman KJ, Boice JD Jr (1982) Epidemiological analysis with a programmable calculator. Boston: Epidemiology Resources Inc.

Rothman KJ, Greenland S (1998) Modern epidemiology, 2nd edn. Philadelphia: Lippincott-Raven.

Sackett DL, Richardson WS, Rosenberg W, Haynes RB (1997) Evidence-based medicine: how to practice and teach EBM. New York: Churchill Livingstone.

Sahai H, Khurshid A (1996) Formulae and tables for the determination of sample sizes and power in clinical trials for testing differences in proportions for the two-sample design: a review. Statistics in Medicine 15: 1-21/

Sato T (1989) On the variance estimator for the Mantel-Haenszel risk difference. Biometrics 45: 1323-1324.

Satten GA, Kupper LL (1990) Sample size requirements for interval estimation of the odds ratio. American Journal of Epidemiology 131:177-184.

Schoenfeld DA (1983) Sample size formula for the proportional-hazards regression model. Biometrics 39: 499-503.

Schuirmann DJ (1987) A comparison of the two one-sided tests procedure and the power approach for assessing the equivalence of average bioavailability. Journal of Pharmacokinetics and Biopharmaceutics 14: 657-580).

Selvin S (1996) Statistical analysis of epidemiologic data, 2nd edn. New York: Oxford University Press.

Shannon CE (1948) A mathematical theory of communication. Bell. System Technical Journal 27: 379-423, 623-656.

Shiue W-K, Bain, LJ (1982) Experiment size and power comparisons for two-sample Poisson tests. Applied Statistics 31: 130-134.

Shoukri MM, Pause CA (1999) Statistical methods for health sciences, 2nd edn. Boca Raton: CRC Press.

Siegel S (1956) Nonparametric statistics for the behavioral sciences. New York: McGraw-Hill.

Siegel S, Castellan NJ Jr (1988) Nonparametric statistics for the behavioral sciences, 2nd edn. New York: McGraw-Hill.

Simon R (1986) Confidence intervals for reporting results of clinical trials. Annals of Internal Medicine 105: 429-435.

Sinclair JC, Bracken MB (1994) Clinically useful measures of effect in binary analyses of randomized trials. Journal of Clinical Epidemiology 47: 881-889.

Smeeth L, Haines A, Ebrahim S (1999) Numbers needed to treat derived from meta-analyses – sometimes informative, usually misleading. British Medical Journal 318: 1548-51.

Sprent P (1993) Applied nonparametric statistical methods, 2nd edn. London: Chapman & Hall.

Sterne JAC, Gavaghan D, Egger M (2000) Publication and related bias in meta-analysis: Power of statistical tests and prevalence in the literature. Journal of Clinical Epidemiology 53: 1119-1129.

Stouffer SA, Suchman EA, De Vinney LC, Star SA, Williams RM Jr (1949) The American soldier: Adjustment during army life, vol. 1. New Jersey: Princeton University Press.

Strickland PAO, Lu S-E (2003) Estimates, power and sample size calculations for two-sample ordinal outcoms under before-after study designs. Statistics in Medicine 22: 1807-1818.

Thode HC Jr (1997) Power and sample size requirements for tests of differences between two Poisson rates. The Statistician 46: 227-230.

Tocher KD (1950) Extension of the Neyman-Pearson theory of tests to continuous variates. Biometrika 37: 130-144.

Tritchler D (1995) Interpreting the standardized difference. Biometrics 51:351-353

Upton JGC (1982) A comparison of alternative tests for the 2x2 comparative trial. Journal of the Royal Statistical Society, Series A, 145: 86-105.

Van Elteren, Ph (1960) On the combination of independent two sample tests of Wilcoxon. Bull Inst. Internat. Stat. 37: 351-361.

Walter SD, Cook RJ (1991) A comparison of several point estimators of the odds ratio in a single 2x2 contingency table. Biometrics 47:795-811.

Westlake W J (1973) The design and analysis of comparative blood-level trials. In: Swarbrick J (ed.) Current concepts in the pharmaceutical sciences: dosage form design and bioavailability. Philadelphia: Lea & Febiger.

Whitehead J (1993) Sample size calculations for ordered categorical data. Statistics in Medicine 12: 2257-2271.

Whitehead A, Whitehead J (1991) A general parametric approach to the meta-analysis of randomized clinical trials. Statistics in Medicine 10: 1665-1677.

Wilcoxon F (1945) Individual comparisons by ranking methods. Biometrics 1: 80-83.

Williams K (1976) The failure of Pearson's goodness of fit statistic. The Statistician 25:49.

Wilson EB (1927). Probable inference, the law of succession, and statistical inference. Journal of the American Statistical Association 22: 209-212.

Wolf FM (1986) Meta-analysis: Quantitative methods for research synthesis. Beverly Hills: Sage Publications.

Woolson RF, Bean JA, Rojas PB (1986) Sample size for case-control studies using Cochran's statistic. Biometrics 42: 927-932.

Woolson RF, Clarke WR (2002) Statistical methods for the analysis of biomedical data, 2nd edn, Wiley-Interscience.

Yanagawa T, Tango T, Hiejima Y (1994) Mantel-Haenszel-type tests for testing equivalence or more than equivalence n comparative clinical trials. Biometrics 50: 859-864; erratum note in Biometrics (1995) 51 :392.

Yusuf F, Peto R, Lewis J, Collins R, Sleight P (1985) Beta blockade during and after myocardial infarction: an overview of the randomized clinical trials. Progress in Cardiovascular Diseases 27:335-371.

Zar JH (1998) Biostatistical analysis, 4th edn. Prentice Hall.
