# Supplementary material for: WINPEPI (PEPI-for-Windows): computer programs for epidemiologists
Source: Epidemiol Perspect Innov. 2004 Dec 17;1:6. doi: 10.1186/1742-5573-1-6 (PMC544871; doi:10.1186/1742-5573-1-6)
Supplement: Additional File 1 — WINPEPI package. WINPEPI programs, with manuals and Pepi Finder. [file 1742-5573-1-6-S1.zip › Describe.pdf]

DESCRIBE

MANUAL

(Version 1.44)

© J.H. Abramson

Revised October 28, 2004

What DESCRIBE does

DESCRIBE is a WINPEPI (PEPI-for-Windows) program, part of the PEPI suite of computer programs for epidemiologists. ("PEPI" is an acronym for "Programs for EPIdemiologists".) It can be run in any version of Windows except Windows 3.

DESCRIBE provides procedures for use in descriptive epidemiology, including the appraisal of separate samples in comparative studies. It can handle categorical data (dichotomous, nominal or ordinal) and numerical data (including survival times). It provides a capture-recapture procedure, appraises screening and diagnostic tests, and can compute sample sizes. There are 14 modules to choose from:

How to use DESCRIBE 2

A. Appraisal of a rate or proportion 5

Confidence intervals, comparison with an expected value, appraisal of the effect of misclassification, and (for randomly recurrent events) estimation of the individual's chances of occurrence.

B. Appraisal of a sequence of rates or proportions 9

Appraisal of a sequence of rates or proportions measured at successive points along a scale. Tests for trend and serial correlation, multiple-comparison tests, regression coefficients for the raw data and log-transformed data, and the relative change per scale unit (e.g. the annual rate of increase or decrease). The program can control the effects of serial correlation and give corrected estimates of the regression coefficients and relative change per scale unit.

C. Appraisal of a frequency table with three or more categories 14

Tests of goodness of fit with an expected distribution (including frequencies based on a Poisson or binomial distribution); index of qualitative variation.

D. Appraisal of numerical data 16

Description of the characteristics of a frequency distribution (including a test for outliers, robust estimators of the mean, and tests of the raw and log-transformed data for normality); comparison of the median or mean with a selected hypothetical value, test for heterogeneity of counts.

- For a specific sequence of numbers (e.g. successive observations): tests for randomness, trend, a change-point, and centrifugality; Sen's estimator of slope; linear regression (parametric and nonparametric); rank

and Pearson's correlation coefficients; smooths the curve. (The numbers can represent nominal categories.)

E. Appraisal of seasonal variation 27

Procedures for the appraisal of seasonal variation in the incidence of disease onsets, births, deaths, consultations, hospital admissions, onsets of sickness absences, or other events.

F. Appraisal of survival data (time-to-event data) 31

Procedures for use in studies of survival; that is, in cohort studies that investigate the period of freedom from a specified event, such as death, occurrence of a complication, discharge from hospital, or return to work.

G. Direct standardization 34

Directly standardizes rates, proportions, means, or other statistics, using standard weights. Can use age intervals as weights

H. Computation of SMR or indirectly standardized rate 37

Computes a standardized morbidity or mortality rate (SMR) or indirectly standardized rate. Can also be used in other analyses of occurrences that are assumed to have a Poisson distribution.

I. Estimation of number of cases, using capture-recapture method 41

For use in an study that aims to estimate the number of individuals with a defined characteristic (usually the presence of a disease) in a population, on the basis of incomplete overlapping lists derived from 2–4 sources.

J. Estimate prevalence, using a cluster sample or stratified sample 44

K. Sample size (to estimate proportion/rate/mean, or find cases) 47

Computation of sample sizes for estimating proportions, prevalences, and means, and for finding a given number of cases; applicable to simple and stratified random samples and to cluster samples.

L1. Appraisal and use of “yes-no” screening/diagnostic tests and measures 52

L2. Comparison or use of two screening or diagnostic tests 59

L3. Meta-analysis of studies of a "yes-no" screening or diagnostic test 65

References 73

WORDS OF CAUTION

This program offers more options than most users will ever need, and will usually display more results than are needed. Ignore the options and results you don't require.

It is unwise to use a statistical procedure whose use one does not understand. This manual cannot supply this knowledge, and it is certainly no substitute for the basic understanding of statistics and epidemiological thinking that is essential for the wise choice of methods and the correct interpretation of their results.

How to use DESCRIBE

First **choose a module** (A to K)**.** Then follow the simple on-screen instructions. To return to the main menu, click on the "*Back to main menu.*" button.

Choosing a module:

If you wish to appraise findings, select an option between **A** and **F** in the main menu.

- To appraise a *rate* or a *proportion*, select option **A**.
- To appraise a *sequence of rates or proportions*, select option **B**.
- To appraise a frequency distribution *with three or more categories* (nominal or ordinal), select option **C.**
- To appraise *numerical data* (normally distributed or not), select option **D**. The numbers may or may not fall into a specific sequence. The numbers can represent a sequence of nominal categories.
- To appraise *seasonal variation* (using monthly, weekly, or daily data), select option **E**.
- To appraise *survival data* (time-to-event data), select option **F**.

To standardize the findings, select option **G** (direct standardization) or **H** (indirect standardization, SMR).

To estimate the number of cases in a population, using incomplete overlapping lists of cases (*capture-recapture method*), select option **I** in the main menu.

To estimate the prevalence of a disease or other attribute from observations in a *cluster sample* or *a stratified random sample*, select option **J** in the main menu.

To estimate the *sample size* required to estimate a prevalence, proportion, or mean, or to find a given number of cases, select option **K** in the main menu.

Easy entry of data:

- If entries are required in different boxes, pressing *Enter* or *Tab* after entering a number will generally take you to the next box; pressing *Escape* will clear the entry.
- If several entries are required in the same box, press *Enter* or *Space* after each entry.
- Optionally, data can be "pasted" into entry boxes (see next page).

Recalling results:

Click on *"View"* in the top menu to display the current session's previous results

Pasting results:

Results shown on the screen are automatically placed in the Windows clipboard**,** from which they can be pasted to other applications, at the site of the cursor (usually by pressing *Shift-Insert* or *Ctrl-V*). Click on "*Not***e**" in the top menu if you wish to add explanatory comments to be placed in the clipboard (or printed)) with the results

If the current session's previous results are recalled (by *clicking on* "View"*)*, text can be marked (drag the mouse over it with button pressed) and copied to the clipboard (by pressing *Ctrl-Insert* or *Ctrl-C)* for pasting elsewhere.

Saving results:

By default, all results of Pepi-for-Windows programs are saved in C:\PEPI.TXT, with a warning if it exceeds 500K. Results also go to C:\PEPI.TMP (for display in the 'View' option); this file may be overwritten unless it is renamed on

quitting DESCRIBE. Click on ***"Save"*** (in the top menu) to see the default procedure or to change it. TXT files can be combined with JOINTEXT, available free from http://www.brixtonhealth.com

Printing results:

Click on *"Print"*. If this fails, try switching the printer off and on again. Or paste the results from the clipboard to Word or another program, and print from there. Results can also be printed from the file in which they are saved. Note: the "Print" option ejects full pages only.

If you get an "*Error opening window*" message, close and re-open DESCRIBE.

PASTING DATA

If the data are available in a text file (e.g. a TXT file created by Notepad), they can be copied to the Windows clipboard [usually by pressing *Ctrl-Insert* or *Ctrl-C*], and then "pasted" into a data-entry box [usually by pressing *Shift-Insert* or *Ctrl-V*]. This can simplify data entry in boxes that require a number of entries (in rows or columns). [Also, data can be copied from a data-entry box and pasted to a text file for future re-use; press *Ctrl-A* to mark it for copying.]

Precautions:

- The data must be pasted into the box as a single block, and not piecemeal.
- The data must be in the format required in the box, with spaces between the numbers; exact alignment of the
  columns is not necessary. For example
  ```
  45 66  1
  20  3 132
  53  11  44
  ```
- If a defined number of rows is required, this number must be entered first, e.g. in the "Number of strata" or "Number of categories" box.
- If row numbers are shown on the left (1, 2, etc.), ensure that the"1" is visible before pasting.
- The cursor must be in the top left corner of the box when the "paste" keys are pressed.

HOW TO OBTAIN PEPI PROGRAMS

All WINPEPI (PEPI-for-Windows) and other PEPI programs can be downloaded free. The latest versions of WINPEPI programs – currently COMPARE2, DESCRIBE, PAIRSetc, and WHATIS – can be downloaded from www.brixtonhealth.com; and the latest release of Version 4 of PEPI, which contains over 40 DOS-based programs (which can be used in Windows) and WHATIS, can be downloaded from www.sagebrushpress.com/pepibook.html or www.simtel.net/pub/pd/54632.html

COMPARE2, DESCRIBE, and PAIRSetc are distributed with manuals (as computer files). A printed manual is available for the DOS-based programs and WHATIS (Abramson and Gahlinger 2001.).

WINPEPI programs are provided with no liability to users and without any warranties, whether expressed or implied. They are copyrighted, but may be freely copied and distributed for personal use; they may not be exploited commercially without permission.

Wilko C Emmens's XYgraph unit (version 1.3.2) creates the graphs displayed by this program.

A. APPRAISAL OF A RATE OR PROPORTION

This module provides **confidence intervals** for a rate or proportion, and can also test for **goodness of fit** with an expected value, appraise the effect of **misclassification**, and (for randomly recurrent events) estimate the individual's **chances of occurrence**.

If the observations are based on a sample of a population of known size, this size can be entered. The program then applies a **finite population correction**, and estimates **confidence intervals for the number of cases** (individuals with the attribute under study) in the population.

Confidence intervals applicable to **inverse sampling** are also reported. These are appropriate if the sample size was not decided in advance, but randomly-selected subjects were investigated until a predetermined number of cases were identified. The program can estimate the confidence intervals of the prevalence when the first case is identified in a case-finding program.

Two basic entries are required - a *numerator* (e.g. the number of persons with a given attribute, the number of cases of a disease, or the number of deaths or other events) or a proportion or rate; and a denominator - a *count denominator* (number of individuals) or *number of person-time units* (usually person-years). If inverse sampling is used, the denominator is the number of cases plus the number of "failures". If a rate is entered, the program may adjust it to ensure that the numerator is a whole number.

In addition, the *total population size* may be entered if the observations are based on a sample; this is unnecessary if the sampling fraction is less than 5% and confidence intervals for the number of cases are not required.

Additional entries are needed if a test for goodness of fit is required., or if the effect of misclassification is to be appraised (see below).

Confidence intervals for the prevalence

For a proportion or a rate with a count denominator, exact Fisher's and mid-P confidence intervals are generally computed. If the denominator is very large, approximations to the exact intervals are displayed; the approximation to the Fisher interval is close enough to be regarded as exact. For a rate with a person-time denominator, exact Fisher's and mid-P confidence intervals are displayed if the number of events is 402 or less, and approximate intervals if it is higher.

Confidence intervals based on Wilson's score-test (Wilson 1927) are also computed if there is a count denominator. These are arguably preferable to the exact intervals in terms of closeness to the required confidence levels (Agresti and Coull 1998). Newcombe and Altman (2000: 46) recommend use of Wilson’s score intervals rather than Fisher's exact method, which is unduly conservative, adding that, especially if the proportion is very close to 0 or 1, it is reasonable to use the mid-P exact method, although it too is somewhat more conservative than the score method.

Finite population correction

If the observations are based on a sample of a population of known size, and this size is entered, a finite population correction is applied. This reduces the variance and hence makes confidence intervals narrower. The correction is necessary only if the sampling fraction is more than 5% (Cochran 1977: 25) and sampling is done without replacement, as it usually is (that is, an individual cannot be selected more than once). The correction has little effect unless the sampling fraction is large.

Confidence intervals for the number of cases

If the total population size is entered, confidence intervals are estimated for the number of cases (subjects with the attribute under study) in the population, by applying the Wilson confidence limits for the prevalence (with a finite population correction) to the population size. The numbers are rounded off to the nearest integer.

Inverse sampling

The program provides confidence intervals for the prevalence that are applicable if inverse sampling was used – that is, if randomly-selected subjects were investigated until a predetermined number of cases were identified. The computation is based on the number of cases and the number of subjects investigated (the number of cases plus the number of "failures").

Three sets of intervals are provided – Fisher's exact intervals, which may be preferred if the denominator is small (Lui 2004: 9), and intervals based on Wald's statistic (which uses a biased estimate of the prevalence) and on Finney's estimator (which uses an unbiased estimate of the prevalence), both of which are derived from large-sample theory. If the number of cases is large, the intervals provided by the three procedures are similar.

The program can estimate the confidence intervals of the prevalence if the number of cases is 1; that is, when the first case is identified in a case-finding program.

Goodness of fit

To test goodness of fit with an expected rate or proportion, the expected value must be entered.

If a *count denominator* is entered, goodness of fit is computed by an exact binomial test.

If *a person-time denominator* is entered, goodness of fit is tested by comparing the observed and (calculated) expected numbers of events, assuming a Poisson distribution. This is appropriate if the event is rare. Exact Fisher's and mid-P tests are performed if neither the observed nor the expected number of events exceeds 200, and a large-sample method in other instances. A large-sample chi-square goodness-of-fit test is also done.

Appraisal of misclassification

This module may be appropriate if the numerator represents the number of individuals with a given characteristic (e.g. the presence of a disease). It requires entry of the sensitivity and specificity of the measure of whatever is enumerated by the numerator; but if a person-time denominator is entered, a specificity of 100% (an absence of false positives) is assumed.

The program computes the true rate or proportion that (given the above sensitivity and specificity values) would have given rise to the observed rate or proportion, assuming that the sample studied is a representative one. If this calculated figure is under 0% or (when a count denominator is used) over 100%, the program displays a warning saying that the observed finding is incompatible with the sensitivity and specificity values entered, and that if the entries are correct, the findings may represent sampling error. Confidence limits are estimated for the computed true rate, based on the confidence limits for the observed rate or proportion. These intervals make no allowance for uncertainty of the sensitivity and specificity values.

If confidence intervals (say 90% or 95%) are available for sensitivity and specificity, it may be enlightening to compute a range for the estimated true rate. To compute an upper bound for the true rate or proportion (when a count denominator is used), enter the lower confidence limit of sensitivity and the upper confidence limit of specificity; to compute a lower bound, enter the upper confidence limit of sensitivity and the lower confidence limit of specificity; if incompatibility is reported, take the lower bound for the true rate or proportion to be zero.

Chances of occurrence

If a person-time denominator is entered, the program computes an individual's chances of 1, 2, 3 or more events in a unit of time (in a year, if a person-year denominator is entered). These estimates apply to randomly recurrent events that follow a Poisson distribution.

METHODS

Confidence intervals

For a proportion or a rate with a *count denominator*, exact Fisher's and mid-P binomial confidence intervals are computed by a procedure from XLIM (version SP2.5) by A. Ray Simons. If the denominator is over 30,000 (or, if the numerator is zero, over 15,000), Fisher's intervals are estimated by a method based on a relationship between the F and binomial distributions (Brownlee 1965); this provides estimates that are close enough to be regarded as exact. Zar's formulae 24.28 and 24.29 are used (Zar 1998, p. 524). If the denominator is over 30,000 or (if the numerator is zero) over 15,000, approximate mid-P intervals are computed by Vollset's procedure (Vollset 1993); for details, see the Pepi manual (Abramson and Gahlinger 2001, p. 260).

Formulae for the computation of Wilson's confidence intervals, based on a inversion of the score test for a proportion, are provided by Newcombe and Altman (2000: 46-47). If the numerator (number of cases) was not entered it is calculated from the proportion or rate, and rounded off to the nearest integer.

Confidence intervals for rates with *person-time denominators* are based on the assumption that the number of events has a Poisson distribution. If there are 40 or fewer events, exact Fisher's intervals are displayed, using tabulated values from Pearson and Hartley (1966), and for 20 or fewer events, exact mid-P intervals are displayed, using tabulated values from Cohen and Yang (1994). If there are more events, closely approximate intervals are computed by formulae 17 and 18 of Rothman and Boice (1982: 29).

Standard error

The formula (Zar 1998: formula 24.2) is

$$\sqrt{[p\,(1-p)\,/\,(n-1)]}$$

where p = proportion
n = count denominator

Finite population correction

If the total population size is entered, the finite population correction (*fpc*) factor for the variance is computed as:

$(N - n) / (N - 1)$

where N = total population size
n = size of sample.

The standard error is multiplied by $\sqrt{fpc}$.

The confidence intervals for the prevalence are corrected by multiplying the distance between each confidence limit and the point estimate by $\sqrt{fpc}$, using modified point estimates (Burstein 1975, Cochran 1977: 59) for this purpose:

$(a - 0.5) / n$ for the lower limit, and
$(a + a / n) / n$ for the upper limit

where a = numerator
n = denominator

Inverse sampling

Confidence intervals appropriate for inverse sampling are provided by Lui (2004) – formula 1.20 for the exact intervals, formula 1.15 for the intervals based on Wald's statistic, and formula 1.19 for the formula based on Finney's estimator. The Wald and Finney methods are not used ifthe proportion is 0 or 1. The computations are based only on the sample; the total population size (if entered) is ignored.

If a single case has been found, the program uses the procedure suggested by George and Elston (1993), which is a special case of formula 1.20 (Lui 2004: 9)..

Goodness of fit

The *binomial test of goodness of fit test* (Siegel and Castellani 1988: 38-42), which is performed when a count denominator is entered, uses the incomplete *beta* function to compute exact cumulative binomial functions (Press *et al.* 1989, pp 188-190). The lower of the two one-tailed probabilities is displayed as the one-tailed P, and the mid-P value is their mean (unless the observed proportion is 0 or 1, in which case the mid-P value is half the lower one-tailed P). The two-tailed P values displayed are conservative approximations, obtained by doubling the one-sided values.
If a person-time denominator is entered, the formulae for *Poisson-based goodness-of-fit tests* are those provided by Rothman and Boice (1982: 29); iterative root-finding procedures are used. The formula for the chi-square goodness-of-fit test, with 1 degree of freedom, is

$$\text{chi-sq.} = (\text{Obs} - \text{Exp})^2 / \text{Exp}$$

where Obs and Exp are the observed and expected numbers of events (Vaeth 2000, Rothman and Greenland 1998, p.235). Chi-square is also computed with a continuity correction (Zar 1998: formula 22.3):

$$\text{chi-sq.} = (|\text{Obs} - \text{Exp}| - 0.5)^2 / \text{Exp}$$

Appraisal of misclassification

To appraise the effect of misclassification, the true rate or proportion is calculated by the formula (based on formula 19-5 in Rothman and Greenland 1998):

$$\text{True proportion} = [P - (1 - Sp)] / (Se + Sp - 1)$$

where P = observed proportion
Se = sensitivity, expressed as a proportion
Sp = specificity, expressed as a proportion.

This formula is applied both to the observed rate or proportion and to its Wilson’s 95% confidence limits (if a count denominator was entered) or its Fisher’s 95% confidence limits (if a person-time denominator was entered).

Chances of occurrence

The computation of the individual's chances of 0, 1, 2, 3 or more events in a unit of time uses formula 3.18 of Armitage *et al.* (2003: 72). The rate per person-time unit is used as the Poisson mean.

B. APPRAISAL OF A SEQUENCE OF RATES OR PROPORTIONS

This module appraises a sequence of rates or proportions measured at successive points along a scale – usually along a time scale, but sometimes along some other dimension, e.g. the number of cigarettes smoked per day, or the severity of a disease (e.g. mild, moderate, severe).

It provides tests for a **trend** and for **departure from a linear trend**, and **multiple-comparison** tests that compare the rates or proportions with one another. **Regression coefficients** are computed, expressing the relationship between the rates or proportions and their positions along the scale, using both the raw data and log-transformed data, and the **relative change per scale unit** (e.g. the annual rate of increase or decrease) is reported. Tests are performed for serial correlation of residuals – i.e., correlation between the deviations of consecutive rates or proportions from the regression line. If the findings suggest the presence of positive serial correlation, the **Cochrane-Orcutt procedure** is used to control its effects and provide corrected estimates of the regression coefficients and the relative change per scale unit. The regression lines, including those based on the Cochrane-Orcutt procedure, are displayed in a **graph.**

If a change-point test is required, or smoothed values for plotting a curve, or nonparametric regression coefficients (for rates or proportions measured at equal intervals along the scale), enter the sequence of rates or proportions in this program's module D.

Three methods of data entry are offered: entry of numerators (e.g. numbers of cases of a disease) and denominators, or entry of proportions and their denominators, or entry of rates and their denominators. Either *count denominators* (numbers of individuals) or *person-time denominators* (e.g. person-years) may be entered. By default, the points along the scale are given scores of 1, 2, 3 etc., which makes them equally spaced. The scores can be changed if the points are not equally spaced; calendar years (1999, 2003, etc.) can be used as scores. For a scale of smoking, the median numbers of cigarettes smoked per day might be used as the scores for categories of smokers.

Tests for trend

Two almost equivalent tests for trend are provided: the *Cochran-Armitage chi-square test* and the *Mantel trend test*. A significant result generally indicates a linear trend, but the trend is not always linear or monotonic. Both the trend test and the accompanying test for *departure from a linear trend* may be significant. The numbers of expected frequencies that are <2 and <5 are displayed, since the Cochran-Armitage test may be uncertain if many expected frequencies are <2, and the P value for the test for departure from a linear trend may be uncertain if many are <5 (Armitage *et al.* 2003: 505). The Mantel test is valid even if the numerators are only zeroes or ones, provided that at least two of the proportions or rates have large numerators and (for pure-count data) that, for at least two rates or proportions, there are a large number of individuals not included in the numerator (Rothman and Greenland 1998: 315). Note that a trend appraisal of age-standardized rates may be misleading if not supplemented by appraisals of the trend of age-specific rates (Choi *et al.* 1999).

In addition, *Bartholomew's test for trend* (Bartholomew 1959a, 1959b) is done if three or four rates or proportions are entered. This test is recommended when the scale represents a qualitative

gradient, so that only arbitrary scores can be allocated to points along the scale – for example in a study using “mild”, “moderate”, “serious”, and “extreme” as measures of severity (Fleiss *et al.* 2003). Unlike the Cochran-Armitage and Mantel tests, this test does not use scores. One-tailed and two-tailed P values are reported, as is the direction of the trend to which the one-tailed P applies. The one-tailed tests are performed after “condensing” the series by calculating weighted averages of any adjacent values whose direction of change diverges from a monotonic upward or downwards tendency. The test assumes that after this “condensing” process each denominator is large, no proportions are very close to0 or 1, and no expected cell frequency is less than 5 (Bartholomew 1959a). It can occasionally happen that the test indicates a significant trend in the opposite direction to the slope of the linear regression line.

Multiple-comparison tests

Multiple tests are performed, comparing each pair of rates or proportions, unless there are over 100 rates or proportions. A Tukey-type (“honestly significant difference test”) multiple-comparison procedure is used.

Regression coefficients

The linear relationship between the rates or proportions and their scores (i.e., their positions along the scale) is summarized by regression equations based on both raw and log-transformed values. Standard errors, 95% confidence intervals, and 1-tailed and 2-tailed P values are displayed for the slope coefficients. The regression lines are displayed in a *graph* (see below).

If nonparametric regression coefficients are required, they can be obtained (for rates or proportions measured at equal intervals along the scale) by selecting this program’s module D.

Relative change per scale unit

The relative change per scale unit is computed. If the score units are years, this is the annual rate of increase or decrease.

Serial correlation of residuals

Two tests are performed for serial correlation of residuals, i.e., correlation between the deviations of consecutive rates or proportions from the regression line. In a study of a time trend this may be caused by factors that have an effect persisting over successive periods, and that do not find their expression in the straight regression line; they may or may not be confounders of the association under study, such as fluctuations in diagnostic criteria.

Serial correlation will produce an unduly narrow confidence interval for the slope coefficient, and its presence may throw doubt on the appropriateness of a straight regression line.

The tests are the *Durbin-Watson test*, which assumes a normal distribution for the residuals, and a *runs test*, which makes no such assumption. The Durbin-Watson D statistic is compared with the lower and upper bounds of its 5% critical level; if it is below the lower bound, this indicates positive serial correlation at the $P < 0.05$ level; if it is below only the upper bound, this is inconclusive; it indicates that there may be positive serial correlation at the $P < 0.05$ level. The runs test is based on the direction of the discrepancies; it compares the number of runs of

uninterrupted sequences in the same direction (positive or negative) with the number expected in a random sequence.

Two-tailed and one-tailed P values (testing for positive and negative correlation) are displayed, a low P value indicates serial correlation.

Cochrane-Orcutt procedure

If the findings suggest there may be positive serial correlation, the program uses the Cochrane-Orcutt procedure to control its effects and provide corrected estimates of the regression coefficients and the relative change per scale unit (e.g. the annual rate of increase or decrease). The procedure is applied to both the raw data and log-transformed values. The corrected estimates are displayed in a *graph* (see below).

Graph

A graph is displayed, showing the simple linear regression line (the regression of the rates or percentages on their scores – i.e., on their positions along the scale) and also, if the findings suggest positive serial correlation, a corrected regression line based on the Cochrane-Orcutt procedure . The regression lines are truncated at the edges of the graph. Optionally, this graph can be replaced by a similar one showing regression lines based on the log-transformed rates or proportions, to permit a visual appraisal of whether log-transformation produces a better fit.

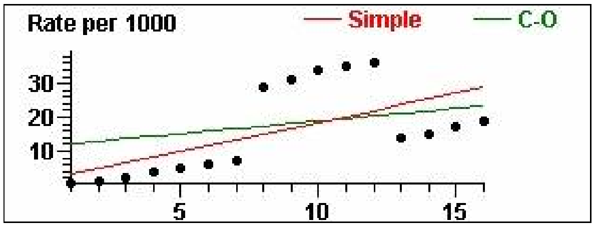

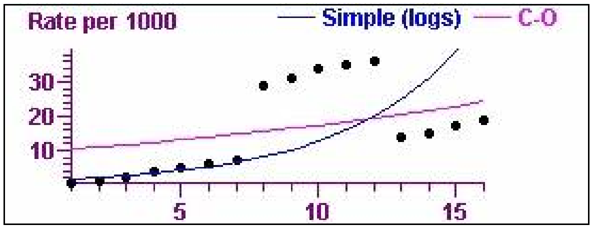

The expected values according to any of the regression equations can be read by clicking on the line. Accuracy can be enhanced by "zooming" (but not for the graph showing log-transformed data) - any segment of a curve can be magnified by pressing *Ctrl* and clicking on the graph, and then drawing a rectangle to outline the required segment. Each graph can be printed, copied to the clipboard for pasting elsewhere, or saved in a bitmap (.BMP) file.

METHODS

If rates or proportions are entered as such – i.e. if numerators are not entered – the numerators are calculated and rounded off to the nearest integer. Corrected rates or proportions (using the rounded-off numerators) are used in subsequent computations.

Tests for trend

The *Cochran-Armitage test* for a linear trend test (Altman 1991: 261-265) and the test for departure from a linear trend use formulae 24.90 and 24.91 of Zar (1998, p. 567). If person-time denominators are entered, they are multiplied by 1,000,000, as suggested by Rothman and Boice (1982: 35).

The *Mantel trend test* uses the formula provided by Rothman (1986: 346). If person-time denominators are entered the variance (in the denominator) is increased by changing the first term to

$$\Sigma\,(a_i)\ /\ \{\Sigma\,(N_i) \bullet [\,\Sigma\,(N_i) - 1]\}$$

Bartholomew's test is described by Fleiss *et al.* (2003), and in more detail by Bartholomew (1995a, 1995b). The series of rates or proportions is "condensed" by calculating weighted averages of adjacent values whose direction of change diverges from a monotonic trend. This is done twice, once for an upward and once for a downward trend, and a chi-square statistic is calculated each time by formula 9.32 of Fleiss *et al.* A one-tailed P value is derived from the higher chi-square, and reported as <0.001, <0.01, <0.025, <0.05, <0.1, and >0.1, and doubled to provide a two-tailed value. The one-tailed P values are read from Tables A.7 and A.8 of Fleiss *et al.*, after calculating values for c_1 and *c2* (formulae 9.34 –9.36 of Fleiss *et al.*), and interpolating in both c_1 and c_2 if necessary.

Multiple-comparison tests

The Tukey-type multiple-testing procedure is described by Zar (1998: 563-564). Formula 13.8 is used for the transformation, or 13.5 if person-time denominators are entered If person-time denominators are entered, rates exceeding 100% are changed to 100% for the purpose of these tests. Results are appraised in relation to critical values of the Q distribution (Zar 1998: Tables B5, B6, B7), and reported as P < 0.001, < 0.01, < 0.05, or not significant.

Regression coefficients

The regression coefficients are based on ordinary least-squares regression analysis , using both the raw data, and (unless there are negative numbers in the series) log-transformed rates or proportions (natural logarithms). If log-transformation is applied, zero proportions are taken as 0.0001, and zero rates are taken as 0.1 per base (e.g., per 1000).

Relative change per scale unit

The slope coefficient (*b*) for the log-transformed data is used to compute the relative change per scale unit, using the following formula (after the Cochrane-Orcutt procedure [see below], *b** is substituted for *b*).

$$100(\exp(b) - 0.1).$$

Serial correlation of residuals

The Durbin-Watson test (Durbin and Watson, 1951) for serial correlation is based on the magnitude of the discrepancies between the observed values and the values computed from the regression equation. The formula is

$$D = \Sigma\,[(e_i - e_{i-1})^2]\ /\ \Sigma\,[(e_i)^2],$$

where e_i = the discrepancy for a specific value (other than the first) in the series)
e_{i-1} = the discrepancy for the previous value in the series.
D is compared with tabulated critical values (the lower bound [DL] and the upper bound [DU]) for P < 0.05 (University of Manchester School of Economic Studies).

The runs test for serial correlation is based on the direction of the discrepancies between the observed values and the values computed from the regression equation. It compares the number of runs of uninterrupted sequences in the same direction (positive or negative) with the number expected in a random sequence. The runs test is described in numerous texts (e.g. Siegel and Castellan 1988: 58-64; Zar 1998: 583-585; Sprent 1993: 82-84). If there are <21 values in the sequence, P is reported as <0.05, <0.1, <0.2 or >0.2 (or, for one-tailed tests, <0.025, <0.05, <0.1 or >0.1), using the table of critical values supplied by Zar (1998: App171-App179). In other instances an approximate P is computed by formulae 25.14 to 25.16 in Zar (1998: 584).

Cochrane-Orcutt procedure

The program performs the Cochrane-Orcutt regression procedure (Cochrane and Orcutt 1949, Johnston and DiNardo 1997) if the original Durbin-Watson D value is below the upper critical level for P < 0.05, or if the runs test for serial correlation yields a one-tailed P < 0.05). For a detailed description of the procedure, see SPSS (2003). The procedure is based on the serial correlation coefficient (*rho*) between the residuals (the deviations from the regression line, e_i, and the residuals of the immediately preceding values in the sequence, e_{i-1}. *Rho* is estimated by dividing $\Sigma\,(e_i \bullet e_{i-1})$ [for $i = 2$ to n] by $\Sigma\,(e_i^2)$ [for $i = 1$ to n], where n is the number of values in the sequence. This estimate of *rho* is used to modify the regression equation between X (score) and Y (rate or proportion) so as to remove the serially correlated

error term. This is done by transforming the X and Y values to X* and Y*, using the formulae

$$X^*_i = X_i - (rho \bullet X_{i-1})$$

and

$$Y^*_i = Y_i - (rho \bullet Y_{i-1}).$$

New regression coefficients a* (intercept) and b* (slope) are then computed, by regressing Y* against X*; a*/(1 - *rho*) is taken as the best estimate of the true intercept, and b* as the best estimate of the true slope. This process is repeated, computing new residuals from the original X and Y values and the new coefficients, and then computing a new *rho*, until the new value of *rho* is within 0.001 of the previous value, or until 10 iterations have been completed. The newest regression coefficients are displayed. The rates or proportions are then log-transformed, and the Cochrane-Orcutt procedure is repeated. Natural logs are used, after changing any zeros to 0.1 (rates) or 0.00001 (proportions). The percentage change per scale unit is computed from the slope coefficient *b** for the log-transformed data, as

$$100[\exp(b^*) - 0.1].$$

C. APPRAISAL OF A FREQUENCY TABLE WITH THREE OR MORE CATEGORIES

This module tests the **goodness of fit** of a distribution in three or more categories with an expected distribution. It is applicable to tables showing frequencies in three or more **nominal or ordinal categories**, including tables whose categories represent **numbers of "events" per "entity**" (0, 1, 2 etc. accidents per person, or children per family, or deaths per month, etc.) and the corresponding numbers of "entities" (persons, families, months, etc.). The expected distribution may be an even one (the same expected number in each category), any specified uneven distribution, or (for categories representing numbers of events) the expected frequencies based on a *Poisson* or *binomial distribution*. . Low P-values indicate nonconformity with the expected distribution.

An **index of qualitative variation** is also provided.

Goodness of fit

Goodness of fit is tested by the **Kolmogorov-Smirnov test for discrete data**, which is appropriate only for ordinal categories (categories that have a meaningful sequence), and by **chi-square tests**, which take no account of the order of the categories. Three chi-square tests are done: *Pearson's goodness-of-fit test* and the *log-likelihood-ratio test*, each of which is preferred by some statisticians (Zar 1998: 475), and the *Cressie-Read test*, which has been recommended as a compromise between the former two. The program reports whether Williams's criterion for preferring the likelihood-ratio chi-square to the Pearson chi-square is met. Since it is generally advised that the chi-square tests should not be used if more than 20% of the expected frequencies used in the analysis are below 5 or if any expected frequency is less than 1 (Siegel and Castellan 1988: 49), the number of such frequencies is reported. If a Poisson or binomial distribution has been met, the program combines categories before doing chi-square tests, to avoid expected frequencies below 1. This is not done for the Kolmogorov-Smirnov test, which is not invalidated by small expected frequencies.

The expected frequencies based on a **Poisson or binomial distribution** (only one of which will generally be of interest) are calculated from the observed mean number of events per entity (for the Poisson distribution) or the probability of an event in the population (for the binomial distribution: the binomial success rate); these values are calculated from the data or (optionally) entered by the user. Use of the Poisson distribution assumes that the event is a random occurrence with a low probability. This limitation does not apply to the binomial distribution. But a binomial distribution can be fitted only if the ceiling score (the maximum number of possible events per person, family, month, etc.) is known. For example, if the table shows numbers of families with 0, 1, 2, etc. diseased members, the maximum score is the family size, and a binomial distribution can be fitted only if the families are of the same size. To fit a binomial distribution, the frequency of each score, from 0 to the ceiling; must be entered. For a Poisson distribution, zero frequencies at the end of the distribution need not be entered; scores at the end of the distribution can be grouped; for example, if the top number of events entered is 5, it can refer to "5 or more".

The program can be used to test the significance of *clustering in time or space* if the "entities" are uniform time periods or equally-sized areas, by appraising conformity with a Poisson (chance)

expectation; a low P value points to clustering (a poor fit with a random temporal or spatial distribution). To test for monthly variation, month-by-month data extending over a number of years should be used. A frequency distribution is required, showing the numbers of months with 0, 1, 2, etc. events. If the test is based on calendar months (which differ in length) it is of course approximate. Clustering in space can be appraised by comparing the numbers of occurrences in equal-sized non-overlapping areas with the chance expectation. The areas may be defined by applying a grid to a map; a frequency distribution is prepared, showing the numbers of areas with 0, 1, 2, etc. events in a given period.

Index of qualitative variation

The index of qualitative variation is computed for both the observed and expected distributions. It ranges from 0 (the most uneven distribution possible) to 1 (an even distribution). It is not computed if the categories represent numbers of events.

METHODS

Binomial and Poisson distributions

The methods of estimating expected frequencies consistent with binomial and Poisson distributions are described by (*inter alia*) Zar (1998: 520-522 and 571-574) and Maxwell (1961: 102-109).

Kolmogorov-Smirnov one-sample test

The Kolmogorov-Smirnov one-sample test for discrete data is described by Siegel and Castellan (1988: 51-56). The appraisal of P is based on the values for two-tailed P = 0.01, 0.05, 0.1 and 0.2 in Table 14.3.3.1 of Zwillinger and Kokoska (1999). The program displays the Kolmogorov-Smirnov statistic D, which is the largest difference detected between the cumulative observed and expected frequencies (as proportions of the total number of observations).

Chi-square tests

Pearson and *log-likelihood chi-square goodness-of-fit tests* are described by (*inter alia*) Zar (1998: 462-464, 473-475).

Williams's criterion for preferring the likelihood-ratio chi-square to the Pearson chi-square is the presence of a difference between any pair of observed and expected frequencies that is not less than the expected frequency (Williams 1976).

The Cressie-Read test is described by Cressie and Read (1984; the formula is on p. 463). Lambda is set at the recommended value of ⅔. If a zero expected frequency was entered, it is changed to 0.0000001 to avoid division by zero during the computation of chi-square. When calculating the log likelihood-ratio and Cressie-Read chi-squares, 0.0000001 is added to the observed frequencies if any of them is zero. The degrees of freedom are the number of categories used in the analysis, minus 1, or (if the Poisson or binomial parameter is computed from the data) minus 2.

For chi-square tests of goodness of fit with a binomial or Poisson distribution, the program combines categories at the end of the distribution if this is necessary to avoid an expected frequency of less than 1.

Index of qualitative variation

The formula for the index of qualitative variation (Healey 1984) is:

$$[1 - \Sigma (P_i^2)] \; / \; \{1 - [1 - (1 / k)]\}$$

where P_i = the fraction of observations in category i

k = the number of categories

D. APPRAISAL OF NUMERICAL DATA

This module appraises a set of numerical data. It describes a *frequency distribution* in terms of its **central tendency** and **dispersion**, displays **box-and-whisker diagrams**, and (optionally) performs **comparisons of the median or mean with a selected hypothetical value**. If the observations are based on a sample of a population of known size, this size can be entered. The program then applies a **finite population correction** when estimating confidence intervals for the mean. It provides a **heterogeneity test** for counts. Some of the results are relevant to all distributions, others only to normal or near-normal distributions. The values may be entered individually, or discrete or grouped values may be entered with their frequencies.

In addition, this module provides a variety of tests and measures that are appropriate only if the numbers constitute a *specific sequence*, e.g. if they are numerical observations made at successive points in time or space. The numbers in the sequence can represent nominal categories, e.g. the two sexes, or different diseases (whole numbers must be used for this purpose, e.g. "1" for male and "2" for female). A warning is displayed if a result is not applicable to numbers that represent nominal categories.

The tests and measures applicable to a specific sequence include three tests for **randomness**, tests and measures of **trend** and **slope**, **correlation coefficients** and **linear regression analysis** expressing the association between the value and its rank in the sequence, a **change-point test**, and a **test for centrifugality**. If the sequence is a time series extending over two or more years, the program can appraise trend while **controlling for seasonal variation**, and examine the similarity of the trends in different seasons**.** **Smoothed values** are computed to facilitate the drawing of a curve. The values are plotted in a **graph**, with regression lines and smoothed curves.

Module B of this program should be used if a sequence of rates or proportions is to be appraised.

Central tendency

The program displays the *mean*, with its standard error and 90%, 95% and 99% confidence intervals, the *median*, with its 95% confidence interval, and three *robust estimators of the mean*, which (like the median) are relatively unaffected by outliers or long tails of the distribution. These are a *trimmed mean*, (which ignores observations below the 1st decile or above the 9th decile), *Huber's m-estimator* (which reduces the influence of these observations), and *a mean that excludes outliers*. The *geometric mean* and *harmonic mean* are also displayed.

Finite population correction

If the observations are based on a sample of a population of known size, and this size is entered, a finite population correction is applied. This reduces the variance and hence makes confidence intervals narrower. The correction is necessary only if the sampling fraction is more than 5% (Cochran 1977: 25) and sampling is done without replacement, as it usually is (that is, an individual cannot be selected more than once). The correction has little effect unless the sampling fraction is large.

Dispersion

The program displays *quantiles* (quartiles, quintiles, octiles and deciles, when warranted by the number of observations), the *standard deviation* and *variance*, the *mean deviation from the mean*, and the *median absolute deviation (MD)* from the median. It reports the number of outliers at each end of the distribution (defined as values further than 5 x MD from the median), and performs *Grubbs' test for outliers*; a low P value indicates the presence of one or more values further from the mean than might be expected in a normal distribution with the given standard deviation.

The *shape of the frequency distribution* is appraised in terms of its *symmetry or skewness* and its *peakedness or flatness*, and two *tests for normality* are performed for both the raw data and log-transformed data. Symmetry or skewness is expressed by *Bowley's quartiles-based skewness coefficient*, which ranges from -1 (extreme skewness to the left) to +1 (extreme skewness to the right), and tested by the *Randles-Fligner-Policello-Wolfe test* or the *Wilcoxon signed-rank test of symmetry* around the sample median. The former procedure tests the hypothesis that the values are generated from a symmetrical distribution with an unknown median, and it has satisfactory power for samples greater than about 20 (Siegel and Castellan 1988: 55-58); since computation is slow, it is not done if the total number of individual values exceeds 150, and the Wilcoxon signed-rank test is done instead. Peakedness or flatness is summarized by *Moors' octiles-based kurtosis coefficient*, which can range from zero to infinity. A value of under 1.233 indicates that the distribution is less peaked than a normal distribution, and a value of over 1.233 indicates that it is more peaked. A *Kolmogorov-Smirnov test for an even distribution* (flatness of the curve) is performed. A low P value indicates that the distribution curve departs from flatness, in the range extending from the lowest value entered to the highest value entered. (To apply the test to a wider range, use the “Enter discrete values” option and enter each required new limit with a frequency of zero.) The test is not done if grouped data are entered. The *Lilliefors test* for normality examines the deviation of the cumulative frequency from the standard normal cumulative distribution; it is performed if there are 6 or more observations; the result is reported as $P < 0.01$, $P < 0.05$, or 'not significant'. The *D'Agostini-Pearson test* is based on tests for skewness and kurtosis; it is appropriate if there are 20 or more observations (Zar 1998: 80).

The shape of the frequency distribution is pictured in *box-and-whisker diagrams* (see below).

Box-and-whisker diagrams

Two box-and-whisker diagrams are displayed, to permit a visual appraisal of the shape of the frequency distribution.. Each of these pictures the range from the 95th to the 5th percentile (shown as a vertical line [two “whiskers”]) and the range from the 75th to the 25th percentile (shown as a solid box), in relation to the median or geometric mean (shown as a horizontal line). The diagrams are not displayed if there are fewer than 20 values.

Each diagram permits appraisal of the symmetry or skewness of the distribution (by comparing the ranges above and below the median or mean), and its peakedness or flatness (by comparing the height of the box with the length of the vertical “whiskers” line).

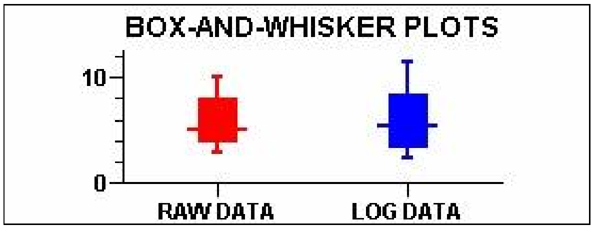

The first plot, which is based on the raw data, shows the observed median and 5th, 25th, 75th, and 95th percentiles. The second plot is based on the log-transformed data, and is not displayed if there are negative or zero values. It shows the geometric mean and the computed 5th, 25th, 75th, and 95th percentiles of the log values (back-transformed to raw units); these percentiles assume a normal distribution of the log values.

Comparison of the two plots may be helpful in a decision on whether to use log-transformed data in analyses.

Comparison of the median or mean with a hypothetical value

Two tests are provided to determine whether the mean or median differs significantly from a specified hypothetical value: a *t-test*, which assumes a normal distribution, and *Wilcoxon's signed-ranks test,* which assumes a symmetric distribution. One-tailed and two-tailed P values are displayed.

Test for heterogeneity

The test for heterogeneity (the Poisson dispersion test) is appropriate if the values that were entered are counts. It indicates whether the counts are more variable, or less variable, than might be expected by chance, by testing whether the values could reasonably have been drawn from Poisson distributions with the same mean (Armitage *et al.* 2002: 234). This might be helpful in, for example, a study of possible clustering , based on counts of occurrences in the cells of a geographical grid, or in a reliability study where undue variability of successive counts (e.g. of micro-organisms), or an excessive similarity of successive counts, might indicate imperfection of the study methods. The program reports a one-sided P value, and if this is 0.05 or less it states whether there is evidence of more-than-chance variation (overdispersion) or less-than-chance variation (underdispersion).

Randomness

There may be interest in appraising the conformity of a sequence of numbers with random expectation, for example when the sequence represents observations whose mutual independence is in question, or when it is a set of purportedly random numbers. The program offers four tests: two *runs tests*, an *up-and-down-runs test* (which is more powerful), and the *mean square successive difference test.* For all of these, a low P-value points to departure from randomness; if the result is significant the hypothesis that the sequence is random should be rejected.

Two *runs tests* are provided. The first, which is done if the sequence contains 25 or more values, and these are whole numbers (possibly representing nominal categories), is based on the number of runs of identical numbers; a two-tailed P-value is reported. The second, done in all instances, is based on the number of runs of "high" (above-median) and "low" (median or lower) values; a two-tailed P-value and two one-tailed P-values are reported. The one-sided alternatives to the null

hypothesis (randomness) are the presence of clustering (fewer runs than would occur at random) and a tendency toward a uniform distribution (more runs than would occur at random).

The *up-and-down-runs test* defines a run as an unbroken sequence of increasing or decreasing observations. A two-tailed P value is reported. The test is not done if the sequence contains only two alternative values (e.g. “1” and “2”, representing “male” and “female”).

The *mean square successive difference test*, which is done if the sequence contains three or more different numbers, is appropriate only if normality can be assumed in the underlying distribution; it is not meaningful if the numbers represent nominal categories. A one-tailed P value is shown; a low value indicates nonrandom variability and serial correlation (of consecutive measurements).

Tests for trend

Two nonparametric tests for a monotonic upward or downward trend are provided: the *Mann-Kendall test for trend* and the *Cox-Stuart test for trend*. One-tailed and two-tailed P-values are displayed. If a time sequence with seasonal data is entered, the Mann-Kendall test is performed twice, once without controlling and once controlling for seasonal variation (see below).

The tests of the significance of correlation and regression coefficients (see below) are tests for a linear trend.

Slope

If the numbers in the sequence represent equally-spaced observations along some dimension – for example, if they are measurements made daily, weekly, monthly, or annually – *Sen's estimator of slope* is appropriate. It estimates the median change per interval (e.g. per day, per week, per month, or per year). The estimator is computed if 4 or more values are entered, and its confidence interval if 5 or more are entered. If a time sequence with seasonal data is entered, Sen's estimator is computed twice, once without controlling and once controlling for seasonal variation (see below).

Slope is also measured by the *b* coefficients provided by linear regression analysis (see below).

Correlation coefficients

Spearman's and *Kendall's rank correlation coefficients* (*rho* and *tau*), which range from -1 to +1, express the linear association between the rankings of the numbers and their ranks in the sequence. Two-tailed and one-tailed P-values are shown; a low P indicates the presence of the association. Approximate 95% confidence intervals are computed for *tau*.

Pearson's correlation coefficient (r), which ranges from -1 to +1, expresses the linear association between the numbers and their ranks in the sequence. Two-tailed and one-tailed P values are shown, as is the coefficient of determination (r^2), which expresses the proportion of the variation that is accounted for by this association. If there are no zero or negative numbers in the series, the numbers are log-transformed and the computation is repeated.

Linear regression analysis

The linear relationship between the numbers and their rank in the series is summarized by regression equations based on *least-squares regression analysis* and *nonparametric regression analysis*; 95% confidence intervals are displayed for the slope coefficients. Least-squares regression analysis is done using the raw numbers and also (unless there are zero or negative numbers in the series) using log-transformed values. The latter findings are used to compute the *relative change*, from one number in the series to the next; this may be helpful if the numbers represent observations made at equal intervals, along say a time scale. The nonparametric procedure does not assume a normal distribution, and has the advantage of robustness - i.e. discrepant 'outlier' observations have a reduced effect; two estimators of the intercept may be shown; the second is recommended if deviations from the regression line can be assumed to be symmetrical.

The simple regression line and the nonparametric regression line are displayed in a graph, together with smoothed curves (see below).

Change-point test

The change-point test appraises whether there is a point at which there is a change in values during the sequence; specifically, whether there is a shift in the median of the distribution. Two-tailed and one-tailed P-values are shown. If a significant change is found (one-tailed $P < 0.05$), the point at which it occurs is reported. If the sequence contains only two alternative whole numbers, the *change-point test for binomial variables* is used; in other instances the *change-point test for continuous variables* is used.

Test for centrifugality

If the sequence contains only two alternative values (probably representing two nominal categories), its conformity with a centrifugal pattern is tested. A low P indicates a good fit with a centrifugal pattern, i.e. a tendency for one of the values to occur near the beginning and end of the sequence, and for the other value to appear near the middle. The appropriate P-value is one-tailed, testing a specific hypothesized centrifugal pattern against all alternatives; the program also doubles this value and displays it as two-tailed P.

Controlling for seasonal variation

If a time series extending over two or more full years is entered, an option is provided for the control of seasonal variation when performing the Mann-Kendall test for trend – the "*seasonal Mann-Kendall test*" (Hirsch *et al.* 1982) – and when computing Sen's estimator of slope (see above), which is computed twice, once without controlling and once for seasonal variation. A *heterogeneity test* is performed, comparing the season-specific slopes. Also, two *measures of heterogeneity*, *H* and *I-squared* are provided, with their approximate 95% intervals. An *H* value of less than 1.2 suggests absence of noteworthy heterogeneity, whereas a value exceeding 1.5 suggests its presence, even if the test is not significant. *I-squared* expresses the proportion of variation that can be attributed to heterogeneity rather than sampling error.

This option is applied if commas are placed between the values for successive seasons when entering the data, and semicolons between the values for successive years. Each year must have the same number of seasons (optionally, 2, 3, 4, 6 or 12), but the number of values per season can vary.

If 2 or more values are entered for a season, the median of these is used for the Mann-Kendall test and Sen's slope estimator (other analyses are based on all the numbers entered). A space can be left if there is no value for a specific season.

Smoothed values

Two procedures are used for computing “objectively” smoothed values, one based on *running medians*, and one on *Fourier transforms*. The running-median procedure is less affected by isolated extremely discrepant numbers. *Residual values* are displayed, for use in pinpointing “out-of-line” numbers. Smoothed values are not computed if the sequence contains only two alternative numbers. Curves smoothed by both methods are shown in the graph (see below).

Graphs

The values in the sequence are plotted in a graph that shows the simple linear regression line (labelled *R1*), the nonparametric regression line (*R2*), curves smoothed by the running medians (*S1*) and Fourier-transforms methods (*S2*), and the change-point (if significant), marked by a red triangle.

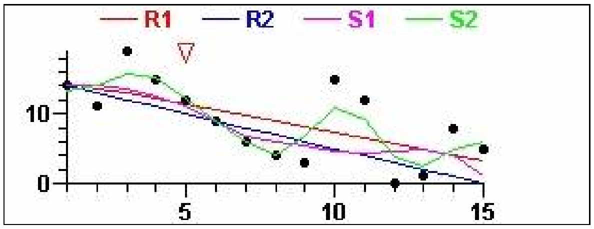

The charted values can be read by clicking on the line. Accuracy can be enhanced by “zooming” – any segment of a curve can be magnified by pressing *Ctrl* and clicking on the graph, and then drawing a rectangle to outline the required segment. The graph can be printed, copied to the clipboard for pasting elsewhere, or saved in a bitmap (.BMP) file.

METHODS

When appraising a frequency distribution, observations in a class containing a range of values are attributed to the middle of the range, except when computing quantiles.

Central tendency

Formulae for the *standard error of the mean* and *confidence intervals for the mean* are provided by Zar 1998 (formulae 6.18 and 7.5).

The *robust estimators of the mean* are a *trimmed mean* (which ignores observations below the 1st decile or above the 9th decile), *Huber's m-estimator*, and a *mean that excludes outliers* (values further than 5 median absolute deviations from the median). Huber's m-estimator is a maximum-likelihood estimator of the mean, computed by an iterative procedure (described by Sprent 1993: 280-282), in which observations beyond a defined distance from the mean are allotted weights (dependent on their distance from the mean) that reduce their effect. The program defines this distance as half the interval between the 1st and 9th deciles of the distribution.

G*eometric and harmonic means* are computed if there are no negative or zero values(the geometric mean if there are at least 20 values, the harmonic mean if individual values are entered).

The median is determined in the same way as other quantiles (see below). For 6 to 100 observations, an exact confidence interval (as close to 95% as possible) is displayed for the median, and an approximate 95% interval is computed if there are more observations (Campbell and Gardner 2000: 37-39 and Table 18.4).

Finite population correction

If the total population size is entered, the finite population correction (*fpc*) factor for the variance is computed as:

$$(N - n) / (N - 1)$$

where N = total population size
n = size of sample.

The standard error of the mean is multiplied by $\sqrt{\text{fpc}}$, which reduces the width o the confidence intervals for the mean.

Dispersion

Each quantile is determined by computing an appropriate index Q, e.g.

$$Q = (N + 1) * 0.5$$

for the median and

$$Q = (N + 1) * 0.75$$

for the upper quartile, and then locating the Qth-lowest item in a sequential array of the N observations (see Zar 1998: 26-27). Q is first rounded off to the nearest integer; if it is half-way between two integers, the quantile is the midpoint between the relevant observations. If the frequencies of ranges of values are entered, the assumption is made that the observations are equally spaced within each group (Cook 1987); the class interval is divided into *k* segments, where *k* is the number of observations in the class, and the observations are allocated to the midpoints of the segments.

Formulae for the standard deviation and variance are provided by Zar 1998 (formulae 4.8 and 4.13).

The *mean deviation from the mean* and the *median absolute deviation (MD) from the median* are self-explanatory.

Outliers are defined as values further than 5 x MD from the median.

Grubbs' test for outliers (Grubbs 1969) is performed by finding the value furthest from the mean, and dividing its absolute distance from the sample mean by the sample standard deviation. If there are between 10 and 50 values, the critical value at a 0.05 significance level for this statistic G is obtained from a table (US Army Corps of Engineers 2001, Table f-1). If the sample is larger (N > 50) an approximate *t* value is computed by the formula

$$t = \sqrt{\{[(N(N - 2)G^2] \;/\; [(N - 1)2 - NG^2]\}}$$

and finally the P value corresponding to this *t* value, at (N - 2) degrees of freedom, is multiplied by N.

Shape of the frequency distribution

Bowley's quartiles-based coefficient of skewness and *Moors' octiles-based coefficient of kurtosis* are computed by formulae 6.10 and 6.12 of Zar (1998: 71-72).

The *Randles-Fligner-Policello-Wolfe test for distributional symmetry* (Randles *et al*. 1980) is based on the examination of skewness in each possible set of three consecutive values, after arranging the values in monotonically ascending order. It is described by Siegel and Castellan (1988: 55-58) and Hollander and Wolfe (1999: 87-94)

The *Wilcoxon signed-ranks test for symmetry* (Zar 1998: 119-120; Siegel and Castellan 1988: 87-95) is based on the discrepancies between the values and the sample median. Nondiscrepant values are ignored. If there are fewer than 20 pairs significance is appraised by using critical levels for one-tailed P = .05, .025, .01, .005,.0025, and (derived from Siegel and Castellan 1988: Table H; and Zar 1998: Table B.12). If the sample is larger a normal approximation is used, with allowance made for ties. The test uses the formula provided by Siegel and Castellan (1988: 92, formula 5.5), but allowing for the effect of ties on the variance by replacing the denominator (as suggested by Sprent 1993: 53 and Mehta and Patel 1991: 7-10) by

$$\sqrt{(\Sigma S_i / 4)},$$

where S_i = the square of the rank of the difference between paired observations.

The *Kolmogorov-Smirnov test* for an even distribution is the goodness-of-fit test for continuous data described by Zar (1998: 478-481), over a given range. At each successive level, the cumulative observed frequency is compared both

with the cumulative expected frequency at that level (expected on the assumption of a continuous distribution over that range) and with the cumulative expected frequency at the next level, and the largest absolute discrepancy in both sets of comparisons is then compared with critical values computed by a formula given by Zar (p. App85) [the second of the formulae cited from Miller (1956)].

The *Lilliefors test for normality* (Lilliefors 1967) is explained by Sprent (1993: 77-78); it uses the critical values provided in Table IV. The *D'Agostini-Pearson test for normality* (D'Agostino 1986, D'Agostino and Pearson 1973) uses formula 6.19 of Zar (1998). To test for normality of log-transformed data, the program uses logs to base 10; value X is transformed to log(X + 1) (Zar 1998: 275); this transformation is not possible if there are negative values.

Box-and-whisker diagrams

The median and quantiles required for the diagram based on *raw data* are determined by the method described above.

The geometric mean shown in the diagram based on *log data* is the mean of the logs of the values, back-transformed to raw units by taking its antilog. The percentiles are computed from the mean and S.D. of the logs of the values (Altman 1992: 60-63), back-transformed to raw units. The formulae are

$\text{Mean} \pm 1.15 \bullet \text{S.D.}$ for the 25th and 75th percentiles,

and $\text{Mean} \pm 1.96 \bullet \text{S.D.}$ for the 5th and 95th percentiles.

Comparison of the median or mean with a hypothetical value

The *t test* used for testing against a hypothetical mean is described by Zar (1998: 91-98, and the *Wilcoxon signed-rank test* used for testing against a hypothetical median is described by Hollander and Wolffe (1999: 79-83). Significance is appraised as for the Wilcoxon signed-ranks test for symmetry (see above).

Test for heterogeneity

The test for heterogeneity (the Poisson dispersion test) is described by Armitage *et al.* (2002: 234-235) and Cuzick (2000). The test is not performed if non-integers are entered, or if there are fewer than 5 counts, or – to avoid misleading use of the chi-square approximation (Armitage *et al.* 2002: 235) − if the mean count is under 2, or if it is under 5 and there are fewer than 15 values.

The test is based on a comparison of the observed variance, $\Sigma\,[(x_i - X)^2]$, with the predicted variance X of a Poisson distribution. The chi-square test statistic is

$\Sigma\,[(x_i - X)^2 / X]$, which is equivalent to

$(n-1).s^2 / X$ (Brown and Zhao 2002)

where x_i = an individual count

X = mean = $\Sigma\,(x_i) / n$

n = number of counts

s = standard deviation of the observed distribution.

P is derived from this chi-square value, at n-1 degrees of freedom, and reported as a one-tailed P value; but if P is 0.95 or more and the observed variance is less than the expected variance, 1–P is reported as the one-tailed P value. If the reported P value is 0.05 or less, the program reports whether the findings indicate overdispersion (when the observed variance exceeds the expected variance) or underdispersion (when the observed variance is less than the expected variance).

Randomness

The *runs test* is described in numerous texts (e.g. Siegel and Castellan 1988: 58-64; Zar 1998: 583-585; Sprent 1993: 82-84). If there are two alternative values in the series, and there are <21 of each value, or if there are more than two alternative values, with <21 “high” (above-median) values and < 21 “low” (median-or-below) values, P is reported as <0.05, <0.1, <0.2 or >0.2 (or, for one-tailed tests, <0.025, <0.05, <0.1 or >0.1), using the table of critical values supplied by Zar (1998: App171-App179). In other instances an approximate P is computed by formulae 24.14 to 24.16 in Zar (1998: 584). The runs test for a series containing more than two alternative whole numbers uses formulae 24.16 to 24.18 in Zar (1998: 584-585); it is not done if the series contains <25 values.

For the *up-and-down-runs test*, the numbers of runs of increasing and decreasing observations are counted twice. First the runs are defined as either monotonically upward or downward (i.e. a pair of equal observations breaks an upward run, but not a downward run) and then as either upward or monotonically downward (i.e. equal observations break a downward run only). If these two methods yield different total numbers of runs, they are averaged (Zar 1998: 588-589). If the sequence contains 20 or fewer values, P values (reported as <0.001, <0.01, <0.02, <0.05, <0.1, <0.2 or >0.2) are obtained from Table B.31 of Zar (1998: App182-App183). For larger numbers, use is made of formulae 25.23, 25.24 and 25.16 in Zar (1998: 584 and 588).

The test statistic for the *mean square successive difference test*, Young's C (Young 1941) is calculated by formula 25.20 in Zar (1998: 587. If the number of observations is between 8 and 50, use is made of the critical values for P = 0.25, 0.10 and 0.05 in Zar's Table B.30 (Zar 1998: App180-App181; for larger numbers, the program uses formula 25.22 in Zar (1998: 587).

Tests for trend

The *Mann-Kendall test for trend* (Mann 1945, Hollander and Wolfe 199: 376) is an application of Kendall's sign-based test for trend (Hollander and Wolfe 199: 363-381). The method is described briefly by McBride (2000) and Salmi *et al*. (2002). The Kendall sample correlation statistic *K* is computed by summing the scores obtained from pairwise comparisons of all values, the score being +1 if the later value is higher, -1 if the earlier value is higher, and 0 if they are equal. If the sequence contains up to 20 values, the one-tailed P value (reported as <0.001, <0.01, <0.025, <0.05, <0.1, or >0.1) and corresponding two-tailed value are obtained from Table A.30 of Hollander and Wolffe (1999: 724-731). If ties are present, this P value is approximate. If there are more than 20 values a large-sample approximation is used; the variance of *K* is computed by formula 4 of Salmi *et al*., making allowance for ties, and the test statistic Z (from which the P value is derived) by formula 5, which includes a continuity correction for *K*.

If a time sequence with seasonal data is entered, the Mann-Kendall test is performed twice, once without controlling for seasonal variation and once controlling for seasonal variation (see below).

The *Cox-Stuart test for trend* is described by Cox and Stuart (1955) and Sprent (1993: 37-39). The test is based on comparisons of the numbers in the first and second halves of the set. The *i*th number (in the first half) is compared with the (i + N/2)th number (in the second half) if the number of values (N) is even, and with the $\Sigma(i + 1 + N/2)$th number if N is odd. A binomial test with probability 0.5 is then applied to the numbers of comparisons showing downward and upward differences between the two halves of the set.

Slope

Sen's estimator of slope (Sen 1968) is computed by comparing each possible pair of values and dividing their difference (subtracting the earlier value from the later value) by the difference between their ranks in the sequence (Salmi *et al*. 2002). The median of these results is the Sen estimator. For 5 to 14 values, an exact confidence interval is displayed, and an approximate 95% interval is computed if there are more values (Campbell and Gardner 2000: 37-39 and Table 18.4).

If a time sequence with seasonal data is entered, Sen's estimator is computed twice, once without controlling for seasonal variation and once controlling for seasonal variation (see below)

Correlation coefficients

Kendall's tau is computed from the Kendall sample correlation statistic *K* (see above) by formula 8.34 of Hollander and Wolfe (1999: 382), taking account of tied ranks, and its approximate 95% confidence interval by formulae 8.37 to 8.39. The significance of *tau* is appraised by the Mann-Kendall test (see above).

Spearman's rho (Siegel and Castellan 1988: 235-244) is computed by a procedure that takes account of tied ranks (an adaptation of the SPEAR procedure in Press et al. 1989: 538-539). An approximate 95% confidence interval (Zar 1998: 398) is estimated if N is 10 or more and *rho* is 0.9 or less, based on the Fisher z transformation. The confidence limits are

$$\exp[2(z \pm 1.96 \bullet SEz) - 1] / \exp[2(z - 1.96 \bullet SEz) + 1]$$

where $SEz = \sqrt{[1.06 / (N - 3)]}$ as recommended by Fieller, Hartley and Pearson (1957, 1961), and

$$z = 0.5 \bullet \ln[(1 + rho) / (1 - rho)]$$

If there are 30 or fewer numbers, the significance of *rho* is appraised by the use of critical levels for one-tailed P = 0.10, 0.05, 0.025, 0. 01, 0.005, and 0.001 (Siegel and Castellan 1988: Table Q). If there are over 30 numbers, a *t*-test is used (Siegel and Castellan 1988: 243, footnote; Press *et al*. 1989: formula 13.8.2, p. 537).

A modification of the PEARSN procedure in Press *et al*. (1989: 535) is used to compute Pearson's correlation coefficient and its significance (Press *et al*. 1989: formula 13.7.5, p. 533). Natural logs are used to log-transform the numbers.

Linear regression analysis

Linear regression coefficients are computed by formula 14.2.6 of Press *et al* (1989, p. 554), and standard errors, confidence intervals and significance by formulae 16.20 and 16.21 of Zar (1998: 337). Natural logs are used to log-transform the numbers. If log-transformed values are used, the percentage change from one number in the series to the next is computed from the *b* (slope) coefficient by the formula

$$(-1 + \exp(b)) \cdot 100.$$

The *nonparametric regression analysis* procedures are described by Daniel (1995: 622-625), Sprent (1993: 195-202) and Sen (1968). The analysis is not done if there are over 146 values in the sequence. Three alternative ways of estimating *beta* (the slope coefficient) are used.

If up to 30 numbers are entered, Theil's estimator (Theil 1950) is computed by a method described by Sprent (1993: 195-198). If more than 30 numbers are entered, Sen's method (Sen 1968) is used ; but if there are more than 146 different values the program employs the abbreviated Theil method (Sprent 1993: 198-202), which uses a systematic sample of the data. For the Sprent and abbreviated Theil methods, which (unlike Sen's method) assume distinct values of the independent variable, the program treats tied observations as if they were not identical by imputing differences of (alternately) 0.000001 or -0.000001.

The point estimate of *beta* (β) is the median value of β_{ij}, where

$$\beta_{ij} = (y_j - y_i) / (x_j - x_i)$$

for each pair of values of the independent variable x (x_i and x_j) and the corresponding values of the dependent variable y (y_i and y_j). Using Sprent's method, β_{ij} is calculated for all of the N(N-1)/2 possible pairs of values; zero values of (x_j - x_i) are changed to 0.000001 or -0.000001 (alternately). In Sen's procedure β_{ij} is calculated only if (x_j - x_i) is not zero. In the abbreviated Theil procedure, each of the first N/2 pairs in the sequence is then linked with the pair situated N/2 positions further along the array; β_{ij} is computed only for these linked observations; zero values of (x_j - x_i) are changed to 0.000001 or -0.000001.

Alpha is estimated by two alternative formulae. The first is the median of the (y_i - $\beta.x_i$) terms for the N pairs of observations, and the second (Daniel 1995: 623-624) is the median of the averages of the (y_i - $\beta.x_i$) terms calculated for each of the pairwise combinations of observations. . Both estimators are shown if they differ. The first estimator is recommended if deviations from the regression model cannot be assumed to be symmetrical; the second estimator of *alpha* (which is not calculated if the abbreviated Theil procedure is used) is recommended if the symmetry assumption is tenable. The regression line shown in the graph (see above) uses the first estimator of *alpha*.

Confidence intervals for beta are obtained from an array of values of b_{ij} in order of increasing magnitude. Sen's method (Sen 1968) uses critical values provided by a large-sample formula based on a variance estimate corrected for ties, and Sprent's method (Sprent 1993: 199-202), based on Theil's, uses critical values based on the critical value for Kendall's *tau* for significance at the nominal 5% level in two-tailed tests, obtained from Siegel and Castellan (1988: 363, Table RII) and Sprent (1993: Table IX). Approximate confidence intervals are estimated in a similar way in the abbreviated Theil procedure, using critical values based on formula 2.3 in Sprent (1993: 34).

Change-point test

The change-point test (Siegel and Castellan 1988: 64-70) uses a method appropriate for binomial variables (Siegel and Castellan 1988: 65-67) if the sequence contains only two alternative whole numbers, and in other instances it uses a method appropriate for continuous variables (Siegel and Castellan 1988: 67-70), with allowance for ties. For the *binomial-variable* test, if each of the alternative whole numbers occurs 25 times or fewer, two-tailed P values are obtained from Table Lii of Siegel and Castellan (1988: 350-351) for P<0.01, <0.05 and <0.1; for larger numbers, Table Liii of Siegel and Castellan (1988: 352) is used; half the two-tailed P value is reported as the one-tailed P value. For the *continuous-variable* test, if both the number of values below the change-point and the number after the change-point are 10 or less, one-tailed and two-tailed P values are derived from Table B10 of Altman (1991: 532-533); formula

4.12 of Siegel and Castellan (1988: 68) is used for larger numbers, after allowing for ties by changing its denominator to the expression for the variance shown in formula 6.12 (p. 134).

Test for centrifugality

For the test of centrifugality (Ghent 1993), the program first constructs a table with 2 rows (for the two alternative values) and *k* columns (for the ranks in the sequence), showing the ranks of the two values; it then changes the ranks in the right-hand part of the table (beyond the median rank) by numbering them from the right-hand end, and 'folds' the right-hand part over the left-hand part, combining cells with the same rank. A Mann-Whitney test is then applied to this table. For small samples the one-tailed P-values are reported as <0.0005, <0.005, <0.01, <0.025, <0.05, <0.10 or >0.10. If there are 10 or fewer in each row, use is made of critical values provided by Zar (1998: App89-App100: Table B11); otherwise a normal approximation is used.

Controlling for seasonal variation

If a time sequence with seasonal data is entered, the Mann-Kendall test and computation of Sen's slope estimator (see above) are repeated, in such a way as to control for seasonal variation. If more than one value is entered for a specific season, the program uses the median of these values; seasons with missing values are omitted from the calculations.

For the *seasonal Mann-Kendall test* (Helsel and Hirsch 2002: 338-341), *K* and its variance (see above) are computed separately for each season, by making pairwise comparisons of the values (in that season) for different years. Ties are not taken into account when computing these variances. The seasonal *K* values and variances are then summated, and the corresponding P value is computed by using the large-sample approximation.

For *Sen's estimator of slope*, (Helsel and Hirsch 2002: 340), separate sets of comparisons are made, each one limited to pairwise comparisons of the values for a specific season; the median of these results (for all seasons combined) is the Sen estimator. Confidence intervals are obtained as described above.

The *heterogeneity test* comparing the season-specific slopes uses formula 10.11 of Fleiss (1981: 163), taking y_i as the slope estimator for a specific season, and w_i as the inverse of its variance. The *measures of heterogeneity* (*H and I-squared*) are computed by the methods described by Higgins and Thompson (2002). *H* is computed by Higgins and Thompson's formula 6, and increased to 1 (indicating absence of heterogeneity) if it less than 1. A test-based interval is computed by Method III. *I-squared* and its 95% interval are computed from *H*, using formula 10.

Smoothed values

Smoothed values are computed by adaptations of *the running-medians* and *Fourier-transforms* procedures described by Hartwig and Dearing (1979: 36-42) and Press *et al.* (1989) respectively.

Smoothing by running medians is done in several stages. First, 5-point moving medians are calculated: each number except the first two and the last two is replaced by the median of the set of five successive numbers of which it is the centre. The first and second numbers are replaced by the median of the first three numbers in the original series, and the last and second-last numbers are replaced by the median of the last three numbers in the original series. Three-point moving medians of the adjusted values are then calculated, copying the modified first and last values unchanged, and this process is repeated until no further changes occur. Two-point running medians are then calculated, and this step is repeated once; this may modify all but the first and last values. The residuals are displayed, i.e., the discrepancies between the original numbers and the values computed by this method.

The *Fourier-transform smoothing method* uses an adaptation of the SMOOFT procedure in Press *et al.* (1989: 544-545), who say "It removes any linear trend, and then uses a Fast Fourier Transform to low-pass filter the data. The linear trend is re-inserted at the end". The program arbitrarily defines the width of the window used when smoothing neighbouring points as 0.2 times the total number of values in the sequence.

E. APPRAISAL OF SEASONAL VARIATION

This module provides procedures for the appraisal of seasonal variation in the incidence of disease consultations, hospital admissions, onsets of sickness absences, or other events, using *monthly*, *weekly*, or *daily* data.

If monthly or daily data are entered, the program applies **Freedman's tests** (using monthly totals or daily data, respectively) and three tests based on the monthly totals: **Edwards's test** (as modified by Roger), the **ratchet circular scan test**, and **Hewitt's test** (as extended by Rogerson). If weekly data are entered, **Pococks's harmonic-analysis procedure** is used. The data and results are summarized in **graphs**.

Data are required for a full year or for a set of full years. If monthly totals are entered, data for separate years must be combined before entry, on the assumption that there is no long-term trend (a rising trend, for example, will mean a higher incidence at each year's end than at its start, spuriously suggesting seasonal variation). If events are entered by weeks, the number of events in each separate week (e.g., 208 weeks in a 4-year period) must be entered in turn; any odd days at the end of the last year are dropped. If events are entered by days, each event must be entered separately by entering its date (day and month) of occurrence; the entries in separate years must be combined before entry, on the assumption that there is no long-term trend.

As a default, the lengths of the months are taken into account in the tests that use monthly or daily data. If the numbers of individuals at risk vary in different months, they can be entered, and appropriate correction factors will be used in Edward's and Hewitt's tests. Optionally, these corrections can be applied in addition to month length, or instead of month length. The latter option is appropriate if the number at risk is influenced by the length of the month (e.g. monthly numbers of births, in a study of congenital anomalies). If data for two or more years are entered, the annual numbers at risk for each month should be combined before entry.

Freedman's tests

Freedman's tests (for monthly and daily data) detect departures from a uniform occurrence throughout the year. The test based on monthly totals may be safely used if the total number of events is 50 or more; for smaller samples, exact dates should be entered (Freedman 1979).

Edwards's test

Edwards's test (Edwards 1961) tests the null hypothesis against the occurrence of a sinusoidal curve with a 12-month period, i.e. a single annual peak and a single trough, with six months between the two. The program computes the amplitude of the curve (as a percentage of the peak frequency), an angle that indicates the time of the peak, and the date that corresponds to this angle. The significance test uses Roger's modified procedure (Roger 1977), and is appropriate even for total sample sizes as small as 20. If the test is significant, ($P < 0.05$), the time of the peak is indicated in a *graph* (see below). The number of individuals at risk in each month may be entered if they need to be taken into account (see above).

Ratchet circular scan test

The ratchet circular scan test (Wallenstein, Weinberg, and Gould 1989) is based on the maximum number of events in two or three consecutive months. It is sensitive to a relatively sharp increase in incidence for a season, superimposed on a constant incidence over the entire year. The test is used if the total number of events is 8 or more. Significant peak periods ($P < 0.05$) are indicated in a *graph* (see below).

Hewitt's rank-sum test

Hewitt's rank-sum test detects a 6-month seasonal peak (Hewitt et al. 1971), and, as suggested by Rogerson (1996) a 5-month or 4-month peak. It is a conservative test. Significant peak periods ($P < 0.05$) are indicated in a *graph* (see below); a set of high-incidence months that includes December and January is split into two segments in the graph.. Rogerson points out that the length of the pulse should be hypothesized in advance, since the simultaneous testing of multiple hypotheses may lead to misleading P values.

Pococks's harmonic-analysis procedure

For Pococks's harmonic-analysis procedure (Pocock 1974), the population at risk should be a fixed cohort of individuals at risk throughout the period (if the population is a changing one, this may affect the occurrence of events).

The number of events must be entered for each week in turn (e.g. 208 entries in a 4-year period), starting with Jan. 1st to 7th of the first year, and dropping any odd days at the end of the last year.

The mean weekly number per week and the variance of the weekly numbers are reported, and a *test for weekly variation* is done; a low P value indicates for more-than-chance variation. *Tests for seasonality and non-seasonal cyclic variation* are then performed, and the seasonal, non-seasonal and random *components of variation* are computed. For these purposes, *seasonal variation* is defined as the occurrence of cyclic trends (with periods ranging from 2 to 52 weeks) that have an exact number of cycles (1 to 26) in a 52-week period, and *non-seasonal variation* as the occurrence of other cyclic trends. The *ratio of seasonal to random components of variation* is reported. Since this ratio is influenced by the mean number of events per week, a *standardized ratio* is computed (for a standard mean of 10 events per week); this permits comparisons with the relative importance of seasonal variation in other sets of data (e.g. for other populations or events, or at other times).

The program computes the statistical significance of seasonal harmonics with cycles of different lengths, and the proportion of variance that is attributable to the harmonic is reported if $P < 0.05$ and the proportion of variance is at least 1%. Interest will usually centre on the harmonic whose cycle has a period of 52 weeks. If this harmonic is significant ($P < 0.05$) and accounts for at least 5% of the variance; the *peak month* is reported and shown in the *graph*; this is an approximation, based on the peaks in different years, and is not displayed if the peaks in any two years differ by more than six weeks.

Graphs

If *monthly or daily data* are entered, the monthly totals are plotted in a graph, which also shows any statistically significant peak periods of two, three, four, five or six consecutive months, marked as P2 to P6 respectively, and (if Edwards's test is positive) the peak date of a 12-month sinusoidal curve, marked by a red triangle.

MONTHLY DATA

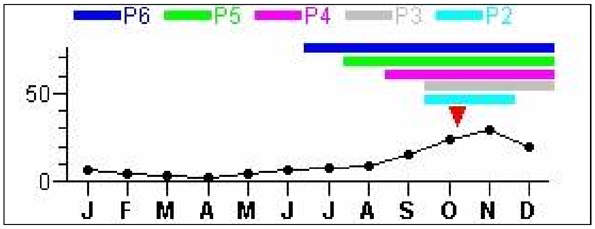

WEEKLY DATA

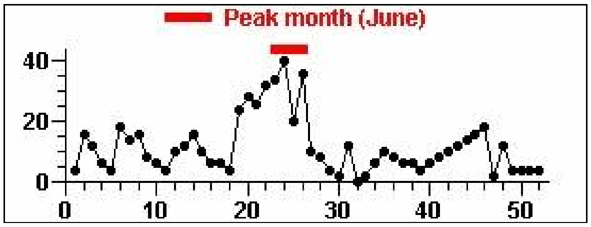

If *weekly data* are entered, the weekly totals (pooling the data for the separate years, on the basis of the fiction that each year contains precisely 52 weeks) are plotted in a graph. If Pococks's harmonic-analysis procedure finds that the harmonic whose cycle has a period of 52 weeks is statistically significant ($P < 0.05$) and accounts for at least 5% of the variance, the approximate month in which its peak occurs is indicated in the graph. This is an approximation, based on the peaks in different years, and it is not reported if the peaks in any two years differ by more than six weeks.

METHODS

Freedman's tests

Freedman's test for seasonal variation using exact dates provides a test statistic derived from $V(N)$, which is a Kolmogorov-Smirnov type statistic (see Freedman 1979). If monthly totals rather than exact dates are entered, the test employs a step distribution similar to a Kolmogorov-Smirnov type statistic for a discrete distribution.
The critical values are:

For exact dates: $P < 0.1$, 1.620; $P < 0.05$, 1.747; $P < 0.025$, 1.862; $P < 0.01$, 2.001.
For monthly totals: $P < 0.1$, 1.29; $P < 0.05$, 1.41; $P < 0.01$, 1.66.

Edwards' test

Edwards' test (Edwards 1961) provides the amplitude of the curve, the angle that indicates the time of the peak, and a chi-square test, using Roger's modification of Edwards' test (Roger 1977). An approximate date corresponding to the peak angle is calculated by determining the proportion of the year equal to

$$d = 365.25 / 360$$

where d is the number of days since beginning of the year.
Correction factors are applied before Edwards's test is performed (Walter and Elwood 1975). Each value of N_i (the number of events in month i) is multiplied by a correction factor

$$\sum M_i / M_i$$

to produce N_i', and each value of N_i' is then multiplied by

$$\Sigma N_i / \Sigma N_i'$$

to obtain a final adjusted value. If numbers at risk are not entered (see above), M_i is the length of the month. If numbers at risk are entered, M_i is either the number at risk (instead of the length of the month) or a person-time denominator (the number at risk multiplied by the length of the month).

Ratchet circular scan test

For the ratchet circular scan test (Wallenstein *et al.* 1989), *peak periods* (of 2 and 3 consecutive months, respectively) are identified, by comparing the numbers of events in each possible period of that length.

If the total number of events is between 8 and 35, P-values are based on critical levels ($P < .05$, $<.025$, and $< .01$) for *n*, the maximum number of events in (respectively) 2 or 3 months. These critical levels are derived from Table 1 in Wallenstein *et al.* (1989). For larger numbers of events, a test statistic R is calculated by the formula

$$R = (n - 1 - N) / \sqrt{[Nw(1 - w)]}$$

where n = number of events in the peak period
N = total number of events
w = the number of days in the peak period, divided by 365.

Critical levels of R (for $P < 0.1$, < 0.05, < 0.025, < 0.01 and < 0.005) are read from the asymptotic distributions shown in Figs. 2 and 3 in Wallenstein *et al.* (1989). These are applicable for N of 50 or more and, as conservative estimates, for N of 36 to 49 (the program displays an appropriate warning).

Hewitt's test

For Hewitt's test (Hewitt *et al.* 1971), the monthly numbers of events are ranked. For each period (4, 5, and 6 months), all possible rank sums based on consecutive months are examined, the set of months with the highest rank sum is defined as the peak period, and the significance level is based on this rank sum. Tied ranks are reduced by first adjusting the monthly frequencies according to the length of the month and any correction factors entered; if ties occur, an average rank is used (Walter 1980). The program uses exact significance levels provided by Walter (1980) and Rogerson (1996), with interpolation for non-integer values (Walter 1980).

Pococks's harmonic-analysis procedure

The harmonic-analysis procedure that is applied to weekly data, which is based on the theory of Fourier analysis, is described in detail by Pocock (1974). The weekly numbers are treated as Poisson random variables, on the assumption that events are rare.

The tests for non-chance variation, seasonal variation, and non-seasonal variation are based on (respectively) the index of dispersion (Pocock's formula 7) and Pocock's formulae 9 and 14. Measures and tests of seasonal variation are based on the combination of harmonics that have a cycle that appears an exact number of times (from once to 26 times) in each 52 weeks (formula 3). The seasonal sum of squares is defined by formula 6 and tested by formula 9, and the non-seasonal sum of squares is tested by formula 14. The percentages of variation attributable to random and seasonal variation are computed by formulae 10 and 12; if the sum of these percentages exceeds 100% it is reduced to 100%, keeping their ratio the same (Pocock p. 109). The standardized ratio of seasonal to random components of variation is computed by formula 17.

Tests for individual seasonal harmonics are based on formula 8, and their contributions to sample variance are defined by formula 13 (negative values being taken as 0%). If the sum of the individual seasonal components diverges from the total seasonal component (formula 6), the individual components are rescaled to the total seasonal component.

The approximate peak week in a one-year cycle is estimated by averaging the peak weeks in each 52-week period. The month in which this week falls is reported as the approximate peak month if the one-year cycle is significant ($P < 0.05$) and accounts for at least 5% of the variance, and the peaks in any two years do not differ by more than six weeks.

F. APPRAISAL OF SURVIVAL DATA (TIME-TO-EVENT DATA)

This module provides procedures for use in studies of survival; that is, in cohort studies that investigate the period of freedom from a specified event, such as death, occurrence of a disease complication, onset of pregnancy, discharge from hospital, or return to work.

The program estimates **median** and **mean survival times** (with their confidence intervals) and the **incidence rate of the event**, and computes **cumulative survival proportions** (the percentage of subjects who are still free of the event after a given period) at each survival time entered, and also for any selected periods of special interest (e.g. 2-year or 5-year survival proportions). A **survival curve** is displayed.

Either survival times or life-table data may be entered. A *survival time* is defined as the number of time units (usually days or months) from the start of observation until occurrence of the event, or (if the event has not occurred) until withdrawal from observation. The main reasons for withdrawal, or *censoring*, are loss of contact, circumstances that dictate removal from the study, and conclusion of the study. Censored survival times are entered by appending "+" to the survival time, e.g. "37+". Survival times may be entered separately for each subject, or the number of subjects with each survival time may be entered; optionally, dates may be entered (the date at which the observation of each subject started and the date of the event or withdrawal from observation), and the program will compute the survival time (inclusive of both dates, to avoid zero survival times). If survival times are entered, the Kaplan-Meier life table procedure is used. Alternatively*, life-table data* may be entered, i.e. the numbers of events and withdrawals in successive periods (e.g. in the first year, the second year, etc.) after the start of follow-up; these periods can differ in length; if life table data are entered, a simple cohort life table computation procedure is used

The procedures assume that there is no **withdrawal bias**. Other assumptions are that (if subjects entered the study at different times) the probability of the end-point event did not greatly alter with time, and (if life-table data are used) that within each follow-up period, both end-point events and withdrawals are evenly spaced.

Median survival time

If survival times are entered and none are censored, their median is reported. If there is censoring or if life table data are entered, the median survival time is estimated by the longest observed survival time for which the cumulative survival probability is 50% or less. Exact or approximate confidence intervals are reported. If the survival probability is not precisely 50% at the reported median survival time, an alternative median is also reported, based on linear interpolation between the times straddling the 50% mark

Mean survival time

The mean survival time is displayed if survival times are entered, with its confidence intervals. If any survival times are censored, the mean is an estimate. The program also computes a mean/median survival time based on the assumption that the distribution is exponential; if this is very different from the observed median, this assumption can be rejected.

Incidence rate of the event

The average rate of events and its confidence intervals are estimated from the mean survival time and its confidence limits. If any survival times are censored, the rate is an estimate.

Cumulative survival proportions

If survival times are entered, cumulative survival proportions are estimated by the Kaplan-Meier procedure. Standard errors and 95% confidence intervals are displayed for periods in which the user has indicated special interest (e.g. 2-year and 5-year survival proportions).

If life table data are entered, the program uses the usual life table technique, and survival proportions are displayed with their standard errors and 95% confidence intervals...

Extreme bounds are displayed for the cumulative survival proportion, based on assumptions of maximal withdrawal bias (see below).

In a comparison of two samples, confidence intervals for the difference between their cumulative survival at a given time can be estimated from the standard error of the difference (*SED*) (the square root of the sum of the squares of the two standard errors); the approximate 95% confidence limits are 1.96(*SED*) above and below the observed difference.

Survival curve

The cumulative survival percentages are plotted by the time interval since the start of follow-up. If any survival times are censored, the graph also displays the *extreme bounds* of the survival percentages, based on assumptions of maximal withdrawal bias (see below).

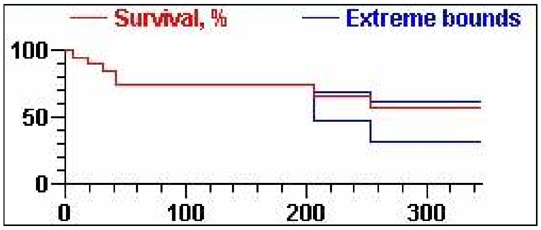

The charted values can be read by clicking on the line. Accuracy can be enhanced by "zooming" - any segment of a curve can be magnified by pressing *Ctrl* and clicking on the graph, and then drawing a rectangle to outline the required segment. The graph can be printed, copied to the clipboard for pasting elsewhere, or saved in a bitmap (.BMP) file.

Withdrawal bias

The procedures assume that the survival of people withdrawn from follow-up is the same as that of people followed up. This may not be so; it has been suggested that the results may be questionable if *the proportion of withdrawals* reaches 10% before the point at which the survival proportion is computed (Axtell, 1963). If survival times are entered, this proportion is reported at each stage (unless over 50 survival times are entered).

As a guide to the possible impact of withdrawal bias, the program also computes *extreme estimates of the cumulative survival proportions*, assuming maximal bias (first in one direction and then the other) of withdrawn subjects. These extreme bounds are shown in the graph.

METHODS

Median survival time

If survival times are entered and none are censored, their median is reported; an exact confidence interval (as close to 95% as possible) is displayed if there are 6 to 100 subjects, and an approximate 95% interval is computed if there are more than 100 subjects (Campbell and Gardner 2000: 37-39 and Table 18.4).

If there is censoring or if life table data are entered, the median is estimated by the longest observed survival time for which the cumulative survival probability is 50% or less. If the survival probability is not precisely 50% at the reported median survival time, an alternative median is also reported, based on linear interpolation between the times straddling the 50% mark (Selvin 1996: 374). The standard error and 95% confidence intervals of the median are computed by the formulae provided by Machin and Gardner (2000: 97-98), based on the survival times at which the survival probabilities reach or cross the 45% and 55% levels, or if these probabilities are equal, the 40% and 60% levels. The *effective sample size* required for the calculation is the total sample size minus the number censored before the median survival time (Machin and Gardner (2000: 94). If the sample is small, the results are unreliable

Mean survival time

If survival times are entered and none are censored, the mean and its confidence intervals are computed in the usual way. Otherwise, a nonparametric estimate of the mean (not assuming an exponential distribution of survival times) is computed, based on formula 11.29 of Selvin (1996: 371); its standard error is computed by formula 11.31, and used for interval estimation; for this purpose the longest survival time is treated as uncensored, even if it is censored

A mean/median survival time is also computed, based on the assumption that the distribution is exponential (Selvin 1996, formula 11.19; Altman 1991: 385). Its standard error is computed by Selvin's formula 11.20.

Incidence rate of the event

The reciprocals of the mean survival time (or the estimate of the mean survival time) and its confidence limits are used as estimates of the average rate of events and its confidence limits.

Cumulative survival proportions

If *survival times* are entered, cumulative survival proportions are estimated by the Kaplan-Meier technique (Kaplan and Meier 1958; Armitage *et al.* 2003: 575-576; Machin and Gardner 2000: 94-96). Standard errors are calculated by Greenwood's formula (Altman 1991; p. 379), and used to estimate 95% confidence intervals (Altman 1991; p. 378). If *life table data* are entered, the program uses the basic formulae provided by (among others) Rothman and Boice (1983: 39). The standard error of the cumulative probability of survival is calculated by formula 17.7 of Armitage *et al*. (2003) and confidence intervals by formulae provided by Rothman (1978).

Withdrawal bias

The *extreme estimates of the cumulative survival proportions*, assuming maximal bias (first in one direction and then the other) of withdrawn subjects., are based on the alternative assumptions that withdrawn subjects (except those with survival times exceeding the longest uncensored survival time) all incurred the event (low bound), and that all withdrawn subjects remained under observation and were free of the event (high bound).

The formulae for the extreme probability of an event at each point (Kaplan-Meier procedure) or in each period (cohort life table method) are

$$(E + W) / N$$

and E / N,

where E = number of events at this point or during this period
W = number of withdrawals at this point or during this period
N = number at risk, including withdrawals.

G. DIRECT STANDARDIZATION

This module combines rates (or other statistics) in two or more strata of a group or population, using standard weights for the various strata, so as to provide a standardized rate (or other statistic) for use in comparisons with other groups (using the same weights). This neutralizes the possible confounding effect of differences between the groups in the relative sizes of the strata, e.g. (for age-standardized rates) in age composition, although it may be less informative than performing a separate comparison in each stratum.

The statistics to be standardized include rates, proportions, and means, and differences between rates, proportions or means; but not ratio measures (such as rate or risk ratios). Direct standardization is most commonly used for morbidity and mortality rates.

The *weights* may be proportions, percentages, or absolute numbers (the sizes of the strata in a selected standard population, or any other appropriate figures). To give equal weight to each stratum, for example, "1" might be entered for each. Age standardization is often based on the age distribution of the population of a given country at a given time, or of a hypothetical standard population. For convenience, the program displays weights based on the age composition of three **hypothetical standard populations**.

For age-standardization, the program can also use **age intervals as weights**, dispensing with the need for a standard population. For this purpose, the required weights are the numbers of years in successive age intervals −for example, a weight of 5 for a 20-24 years stratum, and 10 for a 25-34 years stratum.

If confidence intervals are not needed, the only entries required are the weights and the values. The computation of confidence intervals requires extra information – standard errors or denominators. Numerators (numbers of cases) and denominators can be entered instead of rates or proportions. Separate provision is made for the entry of count ("number-of-persons") denominators and person-time denominators. In the program instructions the former are termed "denominators". and the latter "PT denominators"; the term "rate" refers to a measure with either kind of denominator.

Unless age intervals are used as weights, the standardized value is a *weighted average* of the values entered for the various strata. The program computes a standard error and confidence intervals. If denominators are entered, the overall crude rate or proportion is also displayed.

If age intervals are used as weights, the standardized rate is the *weighted sum* of the age-specific rates, i.e. the overall or *cumulative rate* during the age-span covered. The program also computes the *risk* during this age-span, the approximate standard error of the age-standardized rate, and approximate 95% confidence intervals for the rate and for the corresponding risk.

If over eight strata are entered, the display of weights or values may sometimes fall out of alignment with the stratum numbers. This does not affect the computation, but a warning may be shown, to avoid confusion.

Hypothetical standard populations

Optionally, the program displays tables of standard weights representing the age distributions of idealized world, Africa, and European populations. The world population is a new WHO standard, representing the estimated age-structure of the world population in 2000-2025 (Ahmad *et al.).* The standard African population is relatively young, and the standard African population is relatively old (Waterhouse et al. 1976, Breslow and Day 1987: 54).

STANDARD POPULATIONS

Standard population WORLD (WHO)

Age	Weight
0	2.4
1-4	9.6
5-9	10
10-14	9
15-19	9
20-24	8
25-29	8
30-34	6
35-39	6
40-44	6
45-49	6
50-54	5
55-59	4
60-64	4
65-69	3
70-74	2
75-79	1
80-84	0.5
85+	0.5

Standard population AFRICAN

Age	Weight
0	2
1-4	8
5-9	10
10-14	10
15-19	10
20-24	10
25-29	10
30-34	10
35-39	10
40-44	5
45-49	5
50-54	3
55-59	2
60-64	2
65-69	1
70-74	1
75-79	0.5
80-84	0.3
85+	0.2

Standard population EUROPEAN

Age	Weight
0	1.6
1-4	6.4
5-9	7
10-14	7
15-19	7
20-24	7
25-29	7
30-34	7
35-39	7
40-44	7
45-49	7
50-54	7
55-59	6
60-64	5
65-69	4
70-74	3
75-79	2
80-84	1
85+	1

The list of standard weights can be truncated at either end (or at both ends) if the study deals with a restricted age-span. If an age-stratum is wider than those shown in the table, the weights in the table should be combined: if in the table the 5-9 year and 10-14 year age-groups have weights of 10 and 9 respectively, the weight for the 5-14 year age-group is 19.

Use of age intervals as weights

This method of age-standardization (Day 1976; Breslow and Day 1980: 49-53; Breslow and Day 1987: 57-61; Selvin 1996: 360-362; Abramson 1995) is appropriate for incidence and mortality rates. The procedure assumes that the incidence or mortality density is at least approximately constant within the age intervals for which specific rates are entered; the narrower these intervals, the more valid the results.

The adjusted rate is the sum of the age-specific rates, weighted by the number of years in each age interval; in effect, this gives each single year of age the same weight; the procedure may be seen as use of a hypothetical standard population in which each single-year age-group has the same size (Hill and Benhamou 1995). The weighted sum of the age-specific rates is the overall or *cumulative rate* for the age-span covered by the data. It meets the basic purpose of age-standardization, since differences observed between the rates calculated in different populations cannot be attributed to differences in their age distribution. The overall rate should be regarded as a rate having a person-time denominator.

The program also computes the approximate *risk* during the age-span covered. This is the probability of occurrence (of the disease, death, etc.) for an individual who is at risk during the total age-span (i.e., assuming no deaths of competing causes). Reservations have been expressed about

the use of this method of standardization when comparing populations with widely different all-cause mortality rates (Inskip 2000).

METHODS

If rates with person-time denominators are entered, the number of cases (numerator) in each stratum is calculated and rounded off to the nearest integer, for use in the computation; this may modify the rate.

Unless age intervals are used as weights (see below), the standardized values are simple weighted averages:

Standardized proportion = $\Sigma\,(W_iP_i)$

Standardized rate = $\Sigma\,(W_iR_i)$

Standardized mean (or other statistic) = $\Sigma\,(W_iM_i)$

where R_i = rate in stratum i
P_i = proportion in stratum i
M_i = mean (or other statistic) in stratum i
W_i = weight allotted to stratum i, divided by $\Sigma\, W_i$.

The standard error of a standardized proportion and (if count denominators are entered) of a standardized rate (after converting the rates to proportions) is

$\sqrt{}\ \Sigma\,[W_i^2P_i(1 - P_i) / D_i$ if standard errors are not entered

and $\sqrt{}\ \Sigma\,(W_i^2S_i^2)$ if standard errors are entered

where D_i = denominator in stratum i
S_i = standard error of the proportion in stratum i

If person-time denominators are entered, the approximate standard error of a standardized rate is computed by a formula using the Poisson model (Breslow and Day 1987: 59, formula 2.2):

$\sqrt{}\ \Sigma\,(W_i^2C_i / D_i^{2)}$

where Ci = number of cases (numerator – entered or computed – in stratum i.

The standard error of a standardized mean (or other value) is

$\sqrt{}\ \Sigma\,(W_i^2S_i^{2)}$

where S_i = standard error of the mean (or other value) in stratum i.

The 90%, 95%, and 99% confidence intervals of standardized means, proportions, and rates are computed from the standard error in the usual way, using a normal approximation. For person-time rates, alternative confidence intervals (appropriate for small number of events) are computed by a procedure explained by Dobson *et al.* (1991).

Using age intervals as weights

The age-standardized rate *ASR* is computed as

$ASR = \Sigma\,(w_iP_i)$

where Ri = rate in stratum i (computed from numerator and denominator if not entered)
w_i = weight (number of years) in stratum i.

Its approximate standard error is computed using a Poisson model (Breslow and Day 1987: 59, formula 2.2):

S.E. = $\sqrt{}\ \Sigma\,(w_i^2R_i / D_i^2)$

and its Its approximate 95% confidence limits are computed as

$ASR \pm 1.96(\text{S.E.})$

The same formulae are used, whether or not a person-time denominator is entered.

The risk during the age-span under study is derived from the age-standardized rate by the formula

Risk = 1 - exp(-*ASR*).

Its confidence limits are estimated by substituting the confidence limits of the rate for *ASR* in this formula.

H. COMPUTATION OF SMR OR INDIRECTLY STANDARDIZED RATE

This module performs **indirect standardization.** It computes a standardized morbidity or mortality ratio (SMR) and (optionally) an indirectly standardized rate, with confidence intervals. It can also be used for other purposes, in studies of occurrences that are assumed to have a Poisson distribution.

The SMR is the ratio of observed to expected cases (events); more specifically, it is the ratio of the number of observed cases in a study population to the number that would be expected if the rates in its various strata were the same as those in the strata of a selected *standard (reference) population*. Use of the SMR permits comparisons in which a possible confounder is controlled by using it as the stratifying variable, e.g. by basing the expected number on the rates in the age categories of the standard population.

The *indirectly standardized rate* is a fictional (and usually unnecessary) rate computed by multiplying the SMR by the rate in the standard population.

The observed and expected numbers can be either entered or computed by the program. Instead of the observed number, the observed rate and the size of the study population can be entered, or the number of cases in each stratum, or the rate and denominator in each stratum. Instead of the expected number, the rate or the number of cases and denominator size in each stratum of the standard population can be entered, together with the size of each stratum of the study population. If the observations cover *y* years and annual data are entered for the standard population, a *correction factor* of *y* must be entered.

The program computes exact and approximate **confidence intervals for the SMR and the standardized rate**, and for the number of cases. Optionally, it computes alternative confidence intervals that take account of random variation of the number of expected cases as well as that of observed cases; this may be advisable if the expected numbers are based on rates that were measured in small samples of the standard population. It can also take account of correlation between the observed and expected numbers, as occurs when the study population is part of the standard population.

The program may also be used in **other comparisons of observed and expected numbers of occurrences**, assuming a Poisson distribution, e.g. in studies of space-time clustering.

It also estimates **confidence intervals for an observed number of events** (without entry of an expected number), e.g. in the instance of a rare disease whose occurrence can be assumed to have a Poisson distribution.

The program displays the SMR (the ratio of observed to expected numbers, expressed as a percentage), exact and approximate **significance tests** for the departure of the ratio from 100%, the indirectly standardized rate, and 90%, 95%, and 99% confidence intervals for the SMR, for the standardized rate, and for the number of events.

Indirect standardization

Indirect standardization provides an SMR or a standardized rate for use in comparisons with other study populations or groups (using the same weights) with the aim of neutralizing the possible confounding effect of differences in composition, e.g. in age distribution. The essential feature is the application to the study population of rates observed in the strata of a standard population, in order to determine the expected number of cases. The selection of strata (age, ethnic group, etc.) depends on what possible confounder it is wished to control. Two or more confounders can be controlled simultaneously; for example, by stratifying by both age and ethnic group - this requires a known rate in each age-ethnic category of the standard population, and knowledge of the size of each age-ethnic category of the study population.

Indirect standardization does not require information on the rate in each stratum of the study population. It is the lack of such information, or its uncertainty because of small sample sizes, that often leads to the choice of indirect rather than direct standardization.

The *standard (or reference) population* should preferably be the population with which it is wished to compare the study population (Anderson *et al*. 1980). If several study populations are to be compared, the use of one of them as the standard is unlikely to produce substantially misleading results. Less advisedly, any other population can be used as the standard. Use is often made of the combined study populations, or of a broad population that contains all the study populations that it is wished to compare with one another. The SMR in the standard population is of course 100%.

Confidence intervals for the SMR and standardized rate

Confidence intervals for the SMR are computed on the assumption that the number of events is subject to random variation in accordance with a Poisson distribution (appropriate if the event is rare), whereas the expected number of events is an error-free constant. The estimates may be inaccurate if the denominators are very small. Exact Fisher's and mid-P confidence intervals are computed if there are 70 or fewer events, and approximate Fisher's confidence intervals in other instances. Cohen and Yang (1994) point out that, unlike the conservative Fisher's intervals, the narrower mid-P intervals do not guarantee the nominal confidence level in all instances, but these authors suggest that the discrepancies are of little practical importance.

The program can also estimate alternative confidence intervals that take account of random variation of the number of expected cases (as well as of observed cases); this may be advisable if the expected numbers are based on rates that were measured in small samples of the standard population. The procedure is based on the normal (not the Poisson) distribution, using Fieller's theorem for obtaining confidence limits for the ratio of two normal variables). The confidence intervals are wider than those computed by the Poisson method. They are approximate, and the method may be very inexact or unworkable if numbers are very small. As suggested by Silcocks (1994), the procedure is offered only if both the observed and expected numbers are 10 or more.

The program also provides an extension of the Fieller-based procedure, adjusting the confidence intervals to allow for the effect of the correlation between observed and expected numbers that occurs when the study population is part of the standard population. This may be important if the study population group forms a large proportion of the standard population. The adjustment, which makes the confidence intervals narrower, requires information, for each stratum, on the number of observed events and the number of individuals in each population. The program displays the

adjustment factor, which is the weighted percentage of the standard population that is in the study population (using the numbers of observed cases in the strata as weights).

If a *standardized rate* is computed, its confidence intervals are calculated by multiplying the confidence limits of the SMR by the rate in the standard population.

It may be helpful to know that an approximate confidence interval can be computed for the *ratio of two SMRs or standardized rates* (that use the same standard) by the formula

C.I. = $A_L.E_2$ / (1 - A_L)E_1 to $A_U.E_2$ / (1 - A_U)E_1 (Morris and Gardner 2000):

where A_L and A_U are the lower and upper confidence limits (which can be calculated by Option A of this program) of the proportion O_1 / (O_1 + O_2);

O_1 and O_2 are the observed numbers of events in populations 1 and 2 respectively;

E_1 and E_2 are the expected numbers of events in populations 1 and 2 respectively.

For a significance test, $z = |SMR_1 – SMR_2| / \sqrt{(SE_1^2 + SE_2^2)}$.

Other comparisons of observed and expected numbers of occurrences

The program can also be used for other purposes, in studies that compare observed and expected numbers of occurrences that are assumed to have a Poisson distribution, the expected number being based either on theoretical considerations or on empirical observations.

It could be used, for example, for a simple *test of space-time clustering* (Knox 1964; Selvin 1991: 126-128) by defining ‘closeness in space’ and ‘closeness in time of occurrence’, and then classifying every possible pair of observations as close in both space and time, or not so. If there are n cases the number of possible pairs (N) is $n(n - 1) / 2$. The observed and expected numbers of pairs that are close in both time and space are then entered in the program; if S pairs are close in space and T pairs are close in time, the expected number (under the null hypothesis) is ST / N.

In viewing the results of such analyses, the SMR, divided by 100, would be read as "the ratio of observed to expected numbers".

Confidence intervals are computed on the assumption that the number of events is subject to random variation in accordance with a Poisson distribution (appropriate if the event is rare), whereas the expected number of events is an error-free constant. The estimates may be inaccurate if the denominators are very small. Exact Fisher's and mid-P confidence intervals are estimated if there are 70 or fewer events, and approximate Fisher's confidence intervals in other instances. Cohen and Yang (1994) point out that, unlike the conservative Fisher's intervals, the narrower mid-P intervals do not guarantee the nominal confidence level in all instances, but these authors suggest that the discrepancies are of little practical importance.

Confidence intervals for an observed number of events

The program can estimate confidence intervals for an observed number of events (without entry of an expected number), e.g. in the instance of a rare disease whose occurrence can be assumed to have a Poisson distribution. Exact Fisher's and mid-P confidence intervals are estimated if there are 70 or fewer events, and approximate Fisher's confidence intervals in other instances.

Significance tests

One-tailed tests are done for the significance of the SMR's departure from 100%, or the observed:expected ratio's departure from unity (displayed as the departure of the SMR from 100%), or the standardized rate's departure from the rate in the standard population. If the observed and expected numbers of events are 88 or less, exact Fisher's and mid-P probabilities are shown; otherwise a large-sample test is used. Fisher's P expresses the probability of occurrence, under the null hypothesis, of the observed or a more extreme number of events, and the mid-P value expresses the probability of a more extreme number plus half the probability of the observed number. The one-tailed P-values are doubled to provide two-tailed P-values.

METHODS

If observed numbers are calculated from rates, they are rounded off to the nearest integer. The total may diverge from the true number if the rates were rounded off. The expected number is multiplied by the correction factor (if entered).

Standard error

The standard error of the SMR (the ratio of observed to expected numbers of events) is calculated as

$$\text{S.E.} = \sqrt{\Sigma \{Fi[1 - (Fi / Ni)]\}} \;/\; \Sigma (PiNi)$$ if separate values of F_i are entered, and

$$\text{S.E.} = \sqrt{\Sigma Fi} \;/\; \Sigma (PiNi)$$ if separate values of F_i are not entered data are not entered

where F_i = number of events in stratum i of study population
N_i = size of sratum i of study poulation
P_i = rate in stratum i of standard population.

Significance tests

Formulae for the computation of approximate and exact Fisher and mid-P probabilities are provided by Rothman and Boice (1982: 29: formulae 9-12). Rothman and Boice's formula 19 is used for the large-sample test.

Confidence intervals

Exact Fisher's and mid-P confidence intervals for the SMR, the standardized rate, and the number of events, based on the Poisson distribution, are displayed if there are 70 or fewer events, using tabulated values from Pearson and Hartley (1966) and Cohen and Yang (1994). In other instances, or if an exact confidence interval cannot be computed, Fisher's confidence interval is computed by a large-sample method (Rothman and Boice 1982: 29: formulae 17-18). Confidence intervals for an observed number of cases are based on a Poisson distribution with mean and variance of 1.

The Fieller-based procedure is explained by Silcocks (1994). The confidence limits for the observed:expected ratio, when the expected number is subject to random variation, are the two solutions for x of the quadratic equation:

$$e(e - c)x^2 - (2oe - 2oqc)x + o(o - c) = 0$$

where o and e = observed and expected events
c = chi-square (2.7055 for 90%, 3.84146 for 95%, or 6.6349 for 99% CI).
q = 0 if the effect of correlation between observed and expected numbers is not taken into account
$q = \Sigma (rn / N) \;/\; \Sigma r$ if the effect of correlation between observed and expected numbers is taken into account
r = observed events in a specific stratum
n = size of that stratum in index group
N = size of that stratum in standard population.

For an observed number of 0, the lower confidence limit is zero and the exact upper confidence limit is

$$-2.302585093(\log[a])$$ (Diem 1970: 137, formula 129).

where a = 0.05 for the 90% interval, 0.025 for the 95% interval, and 0.005 for the 99% interval.

I. ESTIMATION OF NUMBER OF CASES, USING CAPTURE-RECAPTURE METHOD

This procedure is appropriate in an epidemiological study that aims to estimate the number of individuals with a defined characteristic (usually the number of cases of a disease) in a population, on the basis of incomplete overlapping lists derived from two to four sources.

The individuals in each list must be identifiable, so that it is possible to determine in which lists they appear. The numbers of cases appearing solely in each of the lists, and in each combination of lists, must be entered.

The program then displays the **estimated total number of cases**, including those not appearing in any list, together with approximate 95% confidence intervals. Optionally, the total population size may be entered, to permit display of the results in “rate” format.

If there are two sources of data, it is assumed that the sources are independent of one another; that is, that the probability of appearing in one list is not increased or decreased by inclusion in the other list. The estimated total number of cases is an underestimate if the two sources are positively correlated, and an overestimate if they are negatively correlated. Two alternative confidence intervals are displayed, one based on the standard error of the estimator, and one on the Poisson distribution. Following a recommendation by Seber (1982), the latter interval may be preferred if the overlap between the lists is small (as defined below), and the former in other instances.

If there are three or four sources of data, the program provides two estimators of the total number of cases, with their confidence intervals. One of these estimators assumes that the sources are independent, and the other does not. .The program also displays **coefficients of covariation** that indicate the direction and degree of the dependencies between lists.

For each list, **percent ascertained (exhaustiveness)** is reported, with its 95% confidence interval. This is the number of cases that appear in the list, expressed as a percentage of the estimated total number of cases.

Estimated total number of cases

For two sources of data, the program uses Chapman's low-bias modification of Peterson's estimator of the total number of cases. Two sets of confidence intervals are displayed – one based on the standard error, and a Poisson confidence interval – with a recommendation as to which is to be preferred (see below). The estimated total may be an underestimate if the two sources are positively correlated, and an overestimate if they are negatively correlated.

For three or four sources of data, the program uses the sample-coverage approach described by Chao and her colleagues. Two estimators of the total number of cases are computed, with their confidence intervals. One is to be preferred if the sources are independent, and the other if there is dependence.

Coefficients of covariation

The coefficients of covariation indicate the direction of the dependencies between lists.

To simplify their interpretation, "standardized" coefficients that can range from -1 to +1 are also presented. These provide a guide to the degree as well as the direction of the dependencies between lists..

Percent ascertained (exhaustiveness)

Percent ascertained (exhaustiveness) is the percentage of the estimated total number of cases that appear in a specific list. As an estimate of its 95% confidence limits, the number of listed cases is expressed as a percentage of the upper and lower confidence limits of the total. The percent ascertained is calculated separately for each estimator of the total number of cases, if two are displayed.

If there are two sources of data, the percent ascertained may be an overestimate if the sources are positively correlated, and an underestimate if they are negatively correlated.

The percent ascertained may be interpreted as *sensitivity* (the percentage of all ascertainable true cases who are included in the list) if all listed cases are true cases or if all lists have the same positive predictive value.

METHODS

Estimated total number of cases

If there are two sources of data, the program uses Chapman's low-bias modification (1948, 1951) of Peterson's estimator of the total number of cases(N). The formula is:

$$N = [(n1 + 1)(n2 + 1) / (m + 1)] - 1$$

and the formula for its S.E. is

$$SE = \sqrt{} \{[(n1 + 1)(n2 + 1)(n1 - m)(n2 - m)] / [(m + 1)2(m + 2)]\}$$

where n1 and n2 are the numbers of cases in lists 1 and 2 respectively.
m is the number of cases appearing in both list 1 and list 2.

Two alternative confidence intervals are displayed, one based on the standard error of the estimator, and one on the Poisson distribution. Following a recommendation by Seber (1982), the latter interval may be preferred if the overlap between the lists is small (if $m < 50$) and, as well, m is less than 0.1(n1) or less than 0.1(n2), and the former interval in other instances.

The 95% confidence interval based on the standard error is computed by a log transformation method (Chao 1987, formula 11):

$$[F + (N - F)/C] \text{ to } [F + (N - F)C].$$

where $F = n1 + n2 - m$
$C = \exp\{1.96 * \sqrt{}[\log(1 + SE^2 / (N - F)^2)]\}$.

For numbers over 100, the Poisson confidence interval is an approximate interval computed by the formulae provided by Rothman and Boice (1982: 29: formulae 17 and 18). For smaller numbers, exact Fisher's intervals are provided, using tabulated values (Altman *et al.* 2000: Table 18.3)).

If there are three or four sources of data, the program uses the sample-coverage approach to capture-recapture analysis proposed by Chao and Tsay (1998) and described in detail by Chao *et al.* (2001).

For three sources, formula 10 in the latter paper provides an estimator for independent sources, and formulae 12 and 13 provide estimators for dependent sources. The program uses formula 12 if the sample size is adequate, and formula 13

if it is not. For this purpose, a sample coverage of 55% or more is regarded as adequate, as suggested by simulation studies by Chao *et al*. (1996). The sample coverage is estimated by computing the proportion of cases (in each list separately) that also appear in one or more other lists, and averaging these proportions (formula 7). Formula 13 provides a "one-step" estimator that can be regarded as a lower bound for positively dependent samples, and an upper bound for negatively dependent samples. Since simple methods of accurately computing standard errors are not available for these estimators, approximate confidence intervals are displayed, arrived at by treating the estimated number of cases as a Poisson variate (using a formula or exact intervals: see above); this is appropriate if the event (the disease) can be regarded as relatively rare.

If there are four sources of data, the computation is similar, using formulae 14 to 17 (Chao *et al*. 2001).

Coefficients of covariation

Coefficients of covariation, based on whichever estimator (Chao *et al*.: 2001: formula 12 or formula 13) is displayed, are computed by formula 13a.

"Standardized" coefficients that can range from -1 to +1 are also displayed. For this purpose, the negative coefficients remain unchanged, since their lower bound is -1. The positive coefficients are divided by the upper bound for the pair of sources under consideration; this upper bound is based on the arbitrary assumption that whenever a case is found in only one of the two lists, it would be found in the other one also. The "standardized" coefficients may be easier to interpret, although with the caveat (Chao, personal communication) that since this upper bound depends on the data, it cannot serve as a standard basis from a statistical point of view; the coefficients can become extremely large, without a universal bound.

Percent ascertained (exhaustiveness)

Percent ascertained (exhaustiveness) is the percentage of the estimated total number of cases that appear in a specific list. If there are two sources of data, the estimation of its confidence interval uses the S.E.-based estimator of the total, and if there are three or four sources of data its confidence interval is computed separately for each estimator of the total.

J. ESTIMATE PREVALENCE, USING A CLUSTER SAMPLE OR STRATIFIED SAMPLE

This module estimates the prevalence of a disease or other attribute from observations in a cluster sample or a stratified sample.

The clusters in a *cluster sample* may be groups of subjects or groups of two or more observations made on each subject. The sizes of the clusters, and the numbers with the attribute under study (the number of "hits" in each cluster) must be entered. Clusters may be entered separately, or clusters with similar findings can be entered together. Optionally, the size of the population to which the study refers can be entered, to permit application of a finite population correction that reduces the width of the confidence intervals; this is unnecessary if the sample includes less than 5% of the population (Cochran 1977: 66: 25).

The program displays the estimated prevalence, with 90%, 95%, and 99% confidence intervals computed by three methods, those of Cochran, Lui, and Fleiss *et al.* The standard error and *design effect* (see below) based on each procedure is reported. The *rate of homogeneity*, or *intraclass correlation coefficient*, which can range up to 1, is also displayed; this is a measure of the similarity of the elements in a cluster.

If *stratified data* are entered, the prevalence in the total population from which the samples were drawn is estimated, with its standard error and 90%, 95% and 99% confidence intervals. For each stratum, the required entries are the size of the sample, the prevalence, and the size of the stratum in the population. The use of approximate population data (e.g., based on an old census) will usually have little effect on the results. If standard errors for the prevalences in the samples cannot be computed from the above data (e.g. if cluster samples were used), standard errors should be entered.

Cluster sample

The clusters in a cluster sample may be randomly-selected groups of subjects, or groups of two or more observations made on the same subject at different times or at different body sites, e.g. in each eye or on various tooth surfaces.

The procedure may be used for a random or near-random sample of clusters selected in more than one stage, e.g. clusters of households selected randomly from communities chosen by a systematic procedure in which the probability of selection is proportional to the community's size, as in EPI (Expanded Program on Immunization) surveys (Bennett *et al.* 1991).

The prevalence of a history of the occurrence of a given disease in the members of a cluster sample in a given period can, if the information is sufficiently valid, be used as an indication of the incidence during that period (Rothenberg *et al.* 1985).

The program displays the estimated prevalence (as a proportion, or per 1,000, etc.) with 90%, 95%, and 99% confidence intervals computed by three methods, those of Cochran, Lui, and Fleiss *et al.*, and the standard error and design effect based on each procedure. The *design effect*, or *variance inflation factor*, is the ratio of the variance to what the variance would be if this were a simple random sample, and it provides an indication of the loss of precision due to the use of a cluster sample. The *rate of homogeneity*, or *intraclass correlation coefficient*, which can range up to 1, is also displayed; this is a measure of the similarity of the elements in a cluster. In unusual circumstances there may be anomalous results; for example when many of the clusters contain a single observation, the rate of homogeneity may be negative, and the design effect less than 1. If the cluster sizes vary, the mean cluster size is displayed, together with the adjusted mean used in calculating the rate of homogeneity.

Stratified sample

If stratified data are entered, the program estimates the prevalence in the total population from which the samples were drawn (with 90%. 95%, and 99% confidence intervals), using weights based on the relative sizes of the strata in the total population. Both the prevalence per 1000 (etc.) and the estimated total number of cases are displayed.

If standard errors (per 1000 etc.) are entered, these (rather than the prevalence data and numbers in the strata) are used as the basis for the computation of a standard error and confidence intervals for the overall prevalence.

METHODS

Cluster sample

The point estimate of the prevalence is the ratio of the total number with the attribute under study to the combined number in the clusters.

Using *Cochran's procedure*, the variance of the prevalence is computed by formula 3.34 of Cochran (1977: 66); if the clusters are equal in size, this is equivalent to formula 3.30. If the size of the population is entered, a finite population correction, $1-f$, is applied in these formulae; f is the ratio of the sample size to the size of the population. If $f = 1$, the variance is zero (Armitage *et al.* 2002: 96).

The 90%, 95%, and 99% confidence limits are computed as

$$p \pm t(\mathrm{SE})$$

where p = prevalence
t = the two-tailed critical value of Student's t at *alpha* = 0.1, 0.05, or 0.01, with (C-1) degrees of freedom.
C = number of clusters
SE = standard error

Lui's procedure uses a logarithmic transformation for estimating confidence intervals (Lui 2004: formula 1.12). If the size of the population is entered, the variance used in this formula is multiplied by the finite population correction (see above), to avoid unduly conservative estimates. If the sample encompasses the whole population, the variance is zero (Armitage *et al.* 2002: 96). The standard error is derived from Lui's formula 1.8.

In the procedure described by *Fleiss et al.* (2003: 441-444: formulae 15.2 and 15.3), the intraclass correlation coefficient is estimated by formula 15.4 . If the size of the population is entered, the program multiplies the variance (formula 15.3) by the finite population correction (see above), to avoid unduly conservative estimates. If the sample encompasses the whole population, the variance is zero (Armitage *et al.* 2002: 96).

For each procedure, the program reports the standard error (the square root of the variance calculated by that procedure) and the design effect. The *design effect* is the ratio of the variance to the variance of a simple random sample of the

same size (Cochran 1977: formula 3.11, p. 52), which is computed as

$[(N - n) / N] [p(1 - p) / (n - 1)]$ if N was entered, or

$p (1 - p) / (n - 1)$ if N was not entered

where N = size of population
p = proportion with the attribute under study
n = size of sample

In the Fleiss procedure, n is used in the denominators, not $(n - 1)$.
If $n = N$, the variance is zero (Armitage *et al.* 2002: 96), and the design effect is not computed.

The *rate of homogeneity (intraclass correlation coefficient)* is computed from the one-way ANOVA components MSB and MSW (the between-cluster and within-cluster mean squares) by the formula (Ridout *et al.* 1999)

$$(\text{MSB-MSW}) / [(\text{MSB} + \text{MSW}) (M - 1)]$$

where M is either the cluster size or, if the clusters vary in size, an adjusted mean cluster size, computed for this purpose by the formula for *n0* in Ridout *et al.* (1999).

Stratified sample

The overall prevalence (the prevalence proportion in the population) is

$$\Sigma (N_i p_i / N)$$

where N_i = number in stratum i of the population
p_i = prevalence in stratum i
$N = \Sigma N_i$

If standard errors are not entered, the variance of the overall prevalence is computed by formula 5.53 of Cochran (1977), and its square root is reported as the standard error. The last term in this formula is replaced by $p_h q_h / (n_h - 1)$, as recommended by Cochran (p. 108), except in the improbable instance that n_h (the sample size in stratum h) is 1, when $p_h q_h / n_h$ is used.

If standard errors are entered for the strata, the standard error of the overall prevalence is computed as

$$\sqrt{\Sigma (W_i^2 s_i^2)}$$

where $W_i = N_i / N$
s_i = standard error in stratum i

Confidence intervals for the overall prevalence are estimated by the formula

$$P \pm zS$$

where P = overall prevalence
S = standard error of overall prevalence
z = 1.6449, 1.96, or 2.5758 for 90%, 95% and 99% intervals, respectively.

The overall prevalence proportion and its confidence limits are multiplied by the total population size to provide estimates of the overall number of cases; these numbers are rounded off to the nearest integer.

K. SAMPLE SIZE (TO ESTIMATE PROPORTION/RATE/MEAN, OR FIND CASES)

This module computes the sample size required for estimating a proportion, prevalence rate, or mean; it is applicable to **simple random samples**, **stratified random samples**, and **cluster samples**. It also computes the size of the random sample required in **case-finding**, in order to identify a given number of subjects with a given disease or other attribute.

The data required for estimating a proportion, prevalence rate, or mean include the desired confidence level (e.g. 95%) and the maximum acceptable difference, which is half the total width of the desired confidence interval. The assumed true proportion, rate, or (for a mean) S.D., and the maximum acceptable difference, must be entered. The program then computes the sample size needed to obtain an estimate with the chosen probability of being within the chosen distance from the true value.

If the size of the population from which the sample is to be chosen is entered, a finite population correction (Cochran 1977: 24) is made; this reduces the required sample size; it has little effect if the sampling fraction is below 5%.

Optionally, the expected percentage of selected subjects expected to be lost because of refusal to participate or other reasons can also be entered. The computed sample size is adjusted by first inflating it (if necessary) to allow for losses (which of course does not compensate for possible selection bias), and then rounded up to the nearest whole number (which may produce apparent small inconsistencies in the results).

In some instances **graphs** are displayed as well as numerical results.

If a single sample is to be used for several purposes, e.g. for estimating the prevalence of different diseases, separate size computations should be done, and the largest sample size selected.

Simple random samples

For a random sample to estimate a *proportion* or *prevalence*, the assumed true value in the population is required; if this cannot be guessed, 0.5 (or 500 per 1,000) should be entered (this is a 'worst-case scenario' that maximizes the sample size).

For estimating a *mean*, the assumed true S.D. in the population is required; the maximum acceptable difference may be entered as a number of units, or as a percentage of the mean (together with the assumed mean in the population).

In addition to reporting the sample size appropriate for the true proportion, rate or S.D. that was entered, the program displays a *graph* showing the sample sizes required for a range of

true values under the specified conditions (confidence level, maximum acceptable difference, and maybe percentage of losses and population size). For proportions, this is a full range, from 0 to 1:

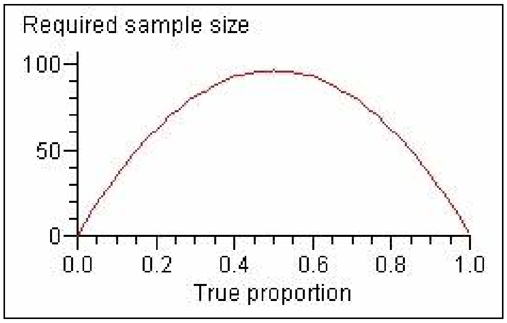

For rates and S.D.s, the graph shows the sample sizes required for true values ranging from half to double the assumed value, under the specified conditions. An assumed rate of 20 per 1.000 produced the following graph, appropriate for true rates from 10 to 40 per 1000:

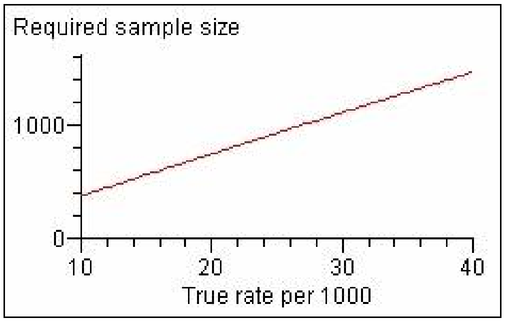

Stratified random samples

If a stratified random sample is to be used, the population size in each stratum is required, together with the assumed proportion, rate or S.D. in each stratum.

Three sample sizes are computed, based respectively on *proportional allocation* (i.e., using the same sampling fraction in each stratum), presumed *optimal (Neymann) allocation*, and (optionally) *low-cost optimal allocation*. Neymann allocation reduces the variance of the estimate by increasing the sampling fraction in a stratum where the variance is larger, and decreasing it where it is smaller (Cochran 1977: 99). Low-cost optimal allocation takes account of the average cost per subject in each stratum (which must be entered if this option is required) as well as the variance; it reduces the sampling fraction in a stratum where the cost is high.

For each method of allocation, the required number of subjects in each stratum is reported as well as the total sample size; if costs are entered, the total cost is also computed.

If the aim is to estimate proportions, rates or means in different population subgroups, a separate sample size computation should be conducted for each subgroup.

Cluster samples

For a cluster sample, it is assumed that the clusters of subjects are equal-sized and randomly selected, as in the cluster surveys advocated by WHO's Expanded Programme on Immunization (Bennett *et al*. 1991), which use clusters drawn from communities whose probability of selection is proportional to their size. The required sample size is larger than for a simple random sample.

In addition to the desired confidence level (e.g. 95%) and maximum acceptable difference, an estimate of the *design effect* or *rate of homogeneity* must be entered. The program then computes the sample size needed to obtain an estimate of the proportion, prevalence or mean with the chosen probability of being within the chosen distance from the true value; the sample size is rounded up to the nearest whole number. (The design effect is the ratio of the variances of the values estimated from a cluster sample and from a simple random sample of the same size. The rate of homogeneity, *or intracluster correlation coefficient* [Bennett *et al*. 1991; Cochran 1977: 241], expresses the degree of similarity of cluster members. If a rate of homogeneity is entered, the program computes the design effect.)

It is generally necessary to guess the design effect or rate of homogeneity, on the basis of published values observed in similar studies (using the same cluster size) in similar populations. Design effects of 2 or more are not uncommon. Rates of homogeneity generally range from close to zero (say 0.02) to about 0.4. To be on the safe side, enter a high value.

Optionally, the size of the population can also be entered, to permit a finite population correction.

The program reports the sample size required, and also (as appropriate) the required number of clusters or cluster size.

In addition, two *graphs* are displayed, showing the required number of clusters or cluster size. The left-hand graph shows the number of clusters or the cluster size required under the specified conditions (confidence level, maximum acceptable difference, maybe percentage of losses, and design effect or rate of homogeneity) if the true value of the proportion, prevalence or S.D in the population is anywhere from half to double the assumed value. The right-hand graph shows the required number of clusters or cluster size under the specified conditions (confidence level, maximum acceptable difference, and maybe percentage of losses) if the assumed proportion. prevalence or S.D. is correct, and the design effect is anywhere between half and double the value entered (or derived from the rate of homogeneity).

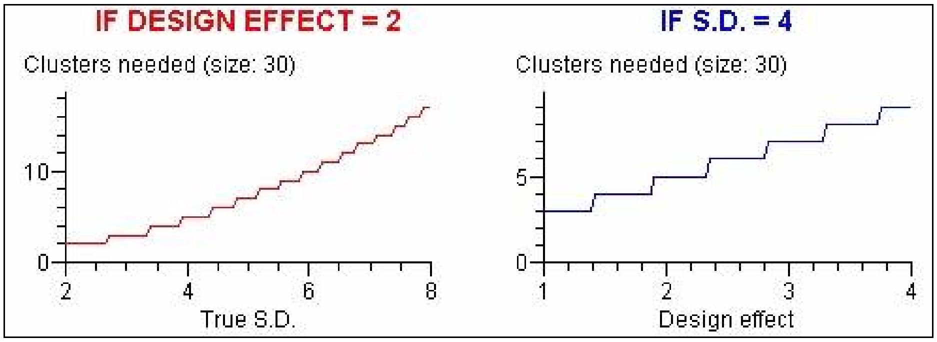

For a stratified cluster sample, a separate sample size calculation for each stratum is recommended (Bennett *et al*. 1991). The precision of the overall study (after weighting the results in accordance with the sizes of the strata) will then be somewhat greater than in individual strata.

Case-finding

To estimate the size of the random sample needed in order to identify a given number of subjects with a specific attribute (Lee 1993), choose a confidence level (e.g. 95%) and enter the estimated prevalence of the attribute and the required number of subjects. The sample size is rounded up to the nearest whole number.

Graphs

If a graph is displayed, the charted values can be read by clicking on the line. Since the curves use interpolation, these values may not coincide perfectly with the reported results. Accuracy can be enhanced by “zooming” - any segment of a curve can be magnified by pressing *Ctrl* and clicking on the graph, and then drawing a rectangle to outline the required segment. The graph can be printed, copied to the clipboard for pasting elsewhere, or saved in a bitmap (.BMP) file.

METHODS

Simple random samples

Formulae for the size of a simple random sample are provided by (among others) Zar (1998: formula 24.35) for a proportion or for a rate (converted to a proportion), and formula 7.7 for a mean; or (using a finite population correction) formulae 24.36 and 7.12 respectively. An iterative procedure is used to estimate *t* for use in formula 7.7 (Zar 1998: 105). The required number is rounded up to the nearest whole number, after making allowance (if necessary) for the percentage of expected losses (L%), by multiplying the number by

$$1 / [1 - (L / 100)].$$

Stratified random samples

The sample *size for estimating a proportion or rate* using a stratified random sample (by *proportional or optimal [Neymann] allocation*, with a finite population correction) is computed by formulae 5.65 and 5.66 [with *Wn* changed to *Wh*] of Cochran (1977). The desired variance *V* is fixed at

$$(d/z)^2,$$

where *d* is the maximum acceptable difference and
z is the 2-tailed normal deviate corresponding to the desired confidence level.

For *optimal allocation*, the sample size in each stratum is estimated by Cochran's formula 5.60; and for *proportional allocation*, by apportioning the total sample size in accordance with the relative sizes of the strata. The required number in each stratum is then rounded up to the nearest whole number, after making allowance (if necessary) for expected losses (see above). The numbers required in the strata are then summed to provide revised estimates of the total sample size.

A similar approach is used for estimating a *proportion or rate* using low-cost optimal allocation; Cochran's formula 5.66 is used, but replacing the numerator of the formula for n0 with

$$\Sigma [W_i \bullet \sqrt{}(p_i q_i) / \sqrt{}(Cost_i)] \bullet \Sigma [W_i \bullet \sqrt{}(p_i q_i) * \sqrt{}(Cost_i)]$$

where W_i, p_i, q_i, and $Cost_i$ refer to the values in a specific stratum.

The proportion of the low-cost sample allocated to a specific stratum is

$$[W_i \bullet \sqrt{}(p_i q_i) / \sqrt{}(Cost_i)] / \Sigma (W_i \bullet \sqrt{}(p_i q_i) / \sqrt{}(Cost_i)$$

In addition, the data entered for the strata are combined to compute the overall population size and the overall proportion or rate, which are then used to estimate the required size (with a finite population correction) of a simple random sample, ignoring the stratification.

The sample size *for estimating a mean* using a stratified random sample (*by proportional or optimal [Neymann] allocation*, with a finite population correction) is computed by formulae 5.47 and 5.48 of Cochran (1977). The desired variance V is fixed at

$$(d/z)2,$$

where d is the maximum acceptable difference
z is the 2-tailed normal deviate corresponding to the desired confidence level.

For *optimal allocation*, the sample size in each stratum is estimated by Cochran's formula 5.26; and for *proportional allocation*, by apportioning the total sample size in accordance with the relative sizes of the strata. The required number in each stratum is then rounded up to the nearest whole number, after making allowance (if necessary) for expected losses (see above). The numbers required in the strata are then summed to provide revised estimates of the totals.

A similar approach is used for *low-cost optimal allocation*, but using the formulae on pages 91 and 92 of Moser (2002).

In addition, the data entered for the strata are combined to compute the overall population size and the overall mean, which are then used to estimate the required size (with a finite population correction) of a simple random sample, ignoring the stratification; this is done by Zar's formula 7.7 (Zar 1998: 105), using a value of t estimated by an iterative procedure; the S.D. (in the total population) that is used is the square root of the pooled variance:

$$\text{pooled variance} = \Sigma\,[(SD_i)^2(Ni - 1] \,/\, \Sigma\,[Ni - 1]$$

where SD_i = S.D. in stratum i
N_i = size of stratum i

Cluster samples

For a cluster sample (without a finite population correction), the sample size is the required size for a simple random sample (computed by the above formulae), multiplied by the design effect E. If the rate of homogeneity H is entered, E is calculated as follows, using the cluster size U (Bennett *et al.* 1991):

$$E = 1 + (U - 1)H$$

To apply a finite population correction for estimating a proportion or rate, the sample size S is calculated as follows (Baras M, personal communication):

$$S = BN \;/\; [D^2(N - 1) + B]$$

where $B = Z^2 \bullet P(1 - P) \bullet E$
P = estimated proportion with the attribute
N = population size
D = maximum acceptable difference

Case-finding

To identify a given number R of subjects with a specific attribute (Lee 1993), the sample size S is:

$$S = [(V - U) / 2P]^{2}$$

where $U = -Z\{\sqrt{[P(1 - P)]}\}$
$V = \sqrt{(U^2 + 4PR)}$
P = assumed proportion in population
Z = standard normal variate corresponding to (1 - confidence level); e.g. 1.645 for 95%.

L1. APPRAISAL AND USE OF “YES-NO” SCREENING AND DIAGNOSTIC TESTS AND MEASURES

This module is applicable to “yes-no” screening and diagnostic tests, and to measures of the presence or absence of any attribute, not necessarily a disease. It is applicable to tests that yield a range of results, if a selected cutting-point is used. The module can also appraise a risk marker (indicative of an increased probability that a disease or other outcome will occur, rather than an increased probability that it is present). [The term “disease” may refer to whatever attribute or outcome the test or measure aims to indicate.]

The required entries are sensitivity, specificity, and (if confidence intervals are required) the sizes of the samples in which they were measured; or, alternatively, the numbers of positives and negatives in samples with and without the disease.

The program appraises the validity of the test or measure by computing **sensitivity and specificity** (if not entered), **chance-corrected sensitivity and specificity**, **false positive and negative rates**, **Youden's index**, the **diagnostic odds ratio**, **likelihood ratios** for positive and negative tests, and **Kullback-Leibler distances**.

The program also provides results that apply to use of the test or measure in a group or population with a known prevalence of the disease or other target attribute. If these results are required, the prevalence can be entered or (optionally) computed from the sizes of the samples in which validity was appraised; the latter option assumes that the combined samples are representative of the population. The additional results are the **post-test probabilities, predictive value**, several measures of **gain in certainty** (measures of the change that the test can be expected to make in the clinical estimate of the probability that the disease is present or absent, the **numbers of tests per case** identified, the **percentage agreement**, and a series of alternative ***kappa*s** that take account of the relative importance attached to false negatives and to false positives. A **graph** shows the relationship of the predictive values of positive and negative results to the prevalence of the disease or other target attribute.

An option is offered for the *computation of post-test probabilities from the likelihood ratio,* without entering sensitivity or specificity . This is for use by clinicians who know a test’s likelihood ratio and wish to decide whether the test is likely to improve the certainty of diagnosis enough to warrant its performance. The pretest probability must be entered. The program computes the **post-test probability** and the **gain in certainty**; a **graph** shows their relationship to the pretest probability.

An option is also offered for estimating the **prevalence of the disease** (or other outcome attribute) from the frequency of positive test results. If confidence intervals are required, this requires entry of the size of the sample in which the test was used., as well as the sizes of the samples in which validity was measured. A **graph** shows the relationship between prevalence and the frequency of positive test results.

Sensitivity, specificity, and false positive and negative rates

Sensitivity and the *false negative rate* refer to the frequency of positive and negative results, respectively, in subjects who (according to a “gold standard) have the disease (or other attribute). S*pecificity* and the *false positive rate* refer to the frequency of negative and positive results, respectively, in subjects without the disease.

In some instances the program revises the entered sensitivity or specificity value to make it consistent with a whole-number numerator. This is done if the entered value differs from the sensitivity or specificity obtained by rounding its numerator (calculated from the entered value and the size of the sample in which it was determined) to the nearest integer.

“Chance-corrected” measures of sensitivity and specificity

“Chance-corrected” measures of sensitivity and specificity (proposed by Brenner and Gefeller 1994) make allowance for the occurrence of chance agreement between the test result and the true status. In a test for the presence of a disease (or other attribute), chance-corrected sensitivity is defined as the proportion of positive results, among people with the disease, that can be attributed to the presence of the disease rather than to chance, chance agreement being estimated by the false positive rate. Similarly, chance-corrected specificity is the proportion of negative results, among people without the disease, that can be attributed to the absence of the disease rather than to chance (estimated by the false negative rate). A value below zero means that sensitivity or specificity is lower than might be expected by chance. These measures do not take disease prevalence into account.

Youden’s index

Youden's index is the sum of sensitivity and specificity (expressed as proportions) minus one. If expressed as a percentage it has been termed the "per cent gain in certainty" (Connell and Koepsell 1985). It is the expected total net gain in certainty, as a proportion of the maximum possible gain (see below).

Diagnostic odds ratio

The diagnostic odds ratio is a measure of the discriminatory power of a test, taking account of both sensitivity and specificity, and without distinguishing between the effects of sensitivity and specificity. It is the ratio of the odds in favour of a positive result in subjects with the disease (or other attribute) to the odds in favour of a positive result in subjects without the disease. It is equivalent to the ratio of the likelihood ratios for positive and negative test results.

An odds ratio of 1 or less means that the test has no discriminatory value

If the four cell frequencies (true and false positives and negatives) are known, and any is zero, an adjusted odds ratio is shown, after adding 0.5 to each cell frequency.

Likelihood ratios

The likelihood ratio is the ratio of the prevalence of a specific result in subjects with the disease (or other attribute) to its prevalence in people without the disease. Likelihood ratios are calculated for positive and negative test results.

A value greater than 10 or less than 0.1 indicates that the test will provide convincing diagnostic evidence, and a value greater than 5 or below 0.2 provides strong diagnostic evidence, whereas if the likelihood ratio is 2 and 0.5 and 2 the test has little or no effect on the certainty of diagnosis (Jaeschke *et al.* 1994).

Kullback-Leibler distances

Kullback-Leibler distances, which are measures of the discrepancy between two probability distributions, can be used as measures of the extent to which performing a test can be expected to alter the odds in favour of correct decisions in "ruling in" positives or in "ruling out" negatives. As suggested by Lee (1999), this may be helpful in situations where a choice must be made between tests. The computation does not require information about the pretest probability.

Post-test probabilities and predictive value

If the prevalence of the disease or other target attribute (i.e., the pretest probability of its presence) is entered, the program estimates the post-test probability % of its presence, conditional on a positive or negative test result.

The indices displayed in graphs are the *predictive value* of a positive test, or positive predictive value, which is the post-test probability of the disease, and the predictive value of a negative test, or negative predictive value, which is the post-test probability of absence of the disease.

Gain in certainty

If the prevalence of the disease (or the pretest probability of its presence) is entered, the program provides several measures of the gain in certainty resulting from performance of the test (Connell and Koepsell 1985). The gain in certainty is the change that the test can be expected to make in the clinical estimate of the probability that the disease is present or absent. Where possible, confidence intervals are provided.

The gain in certainty is the difference between the pre-test probability – that is, the likelihood, based only on the known or assumed prevalence, that the patient has or does not have the disease – and the post-test probability, based on information derived from the test. Gain in certainty is computed separately for positive and negative test results. In each instance it is expressed both as a net difference and as a percentage of the pre-test probability of the presence or absence of the disease. The total gain in certainty is also computed, taking account of the probabilities of positive and negative results. It is expressed both as a net difference and as a percentage of the maximum possible gain in certainty. The separate contributions of positive and negative results to this percentage are displayed.

The gain in certainty is determined by sensitivity, specificity, and the prevalence of the disease in the subgroup in which the test is to be used (usually a specific clinic population, or patients with a specific pattern of clinical manifestations); the entry of an erroneous estimate of prevalence may yield very misleading results. Only the total net gain is uninfluenced by prevalence.

The confidence intervals for post-test probabilities and measures of gain in certainty are based on the assumption that the estimates of sensitivity and specificity are subject to random error but

prevalence is a known fixed quantity. A negative value for the lower confidence limit of any of the measures of gain in certainty indicates a decrease in certainty.

Numbers of tests per case identified

As a guide to the potential cost of a screening program (e.g. in terms of inconvenience or resources), DESCRIBE reports two numbers: the number of tests required to identify one case, and the number of positive tests per case identified. These numbers are valid only if the test is applied in a population with the prevalence that is entered or computed from the sample sizes entered.

The total economic cost of a projected screening program is the first number multiplied by the cost of a screening test, plus the second number multiplied by the cost of the confirmatory investigations required when a screening test is positive, plus setting-up costs and overhead expenses.

Percentage agreement

The percentage of subjects whose test result coincides with their true status is sometimes called the “index of validity” (Taube 1986). It is valid only if the test is applied in a population with the prevalence that is entered or computed from the sample sizes entered.

Unlike *kappa* (see below), the percentage agreement makes no allowance for chance agreement.

Kappa

A set of alternative weighted *kappa*s is computed, showing the agreement between the test result and the true status, after allowing for chance agreement. The weights express the relative importance (“clinical cost”, undesirability) attached to false negatives and false positives (Bloch and Kraemer 1989, Kraemer *et al.* 2002). At one extreme, false positives are regarded as being 100 times more undesirable than false positives; this might be an appropriate *kappa* for a diagnostic test that aims to provide definitive proof that a disease is present. At the other extreme, false negatives are regarded as being 100 times more undesirable than false positives; this might be appropriate for a screening test that aims to find all possible cases.

The *kappa* values apply only to a target population with the disease prevalence that is entered or computed from the sample sizes entered.

Prevalence of the disease

The *prevalence of the disease* (or whatever other attribute the test aims to indicate) in a target population is estimated from the prevalence of positive results in the population, on the assumption that the sensitivity and specificity values are applicable to this population, whether these values were measured in samples of diseased and nondiseased subjects from this population or from similar populations. The expected prevalences of true and false positive results are reported.

The prevalence of the disease is not computed if the prevalence of positive tests is less than the false positive rate, or if the false positive rate exceeds the test’s sensitivity.

Two approximate 95% confidence intervals are computed, one that takes the sensitivity and specificity to be known constant values , and one (computed if the sizes of the samples in which

sensitivity and specificity were appraised) that takes account of uncertainty in the estimation of sensitivity and specificity.

Graphs

If the prevalence of the disease (or other attribute) is entered, a graph is displayed, showing the predictive values of positive and negative results. in relation to prevalence, for prevalences ranging from half to (if possible) double the value that is entered (or derived from the sample sizes entered). Here is an example:

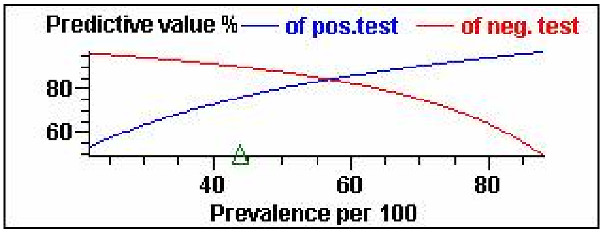

If the frequency of positive test results is entered, a graph is displayed, showing the computed prevalence of the disease (or whatever other attribute the test points to) and the computed prevalence of true positive results (i.e. the prevalence of cases detected by the test), in relation to the frequency of positive test results. The gap between the curves represents the cases missed by the test (false negative results). The range displayed for the prevalence of positive test results extends from half the value entered by the user (or the highest value associated with a disease prevalence of zero, whichever is higher) to twice the value entered by the user (or the lowest value associated with a disease prevalence of 100%, whichever is the lower). Here is an example:

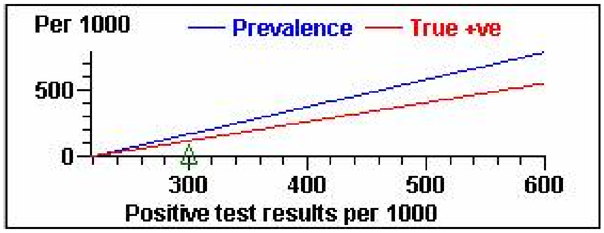

If the option for the *computation of post-test probabilities from the likelihood ratio* is selected, a graph is displayed, showing the post-test probability % of the disease (or whatever other attribute the test points to) and the net gain in certainty (the absolute difference between the pretest and post-test probabilities %), in relation to the pretest probability %. The range displayed for the pretest probability extends from half the value entered by the user to (if possible) twice the value entered by the user. Here is an example:

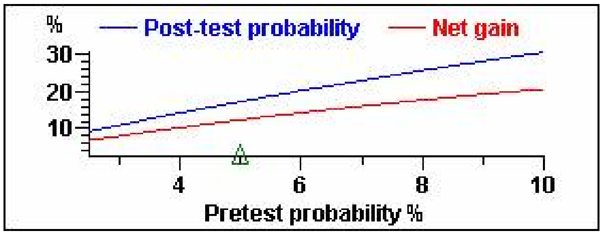

In each of these graphs, the charted values can be read by clicking on the relevant line. Accuracy can be enhanced by "zooming" – any segment of a curve can be magnified by pressing *Ctrl* and

clicking on the graph, and then drawing a rectangle to outline the required segment. The graph can be printed, copied to the clipboard for pasting elsewhere, or saved in a bitmap (.BMP) file.

METHODS

Sensitivity, specificity, and false positive and negative rates

The formulae are:

sensitivity = $a / (a + c)$
specificity = $d / (b + d)$
false positive rate = $b / (b + d)$
false negative rate = $c / (a + c)$

where a = positive result in subjects in whom the disease [or other attribute] is present
b= positive result in subjects in whom the disease [or other attribute] is absent
c = negative result in subjects in whom the disease [or other attribute] is present
d = negative result in subjects in whom the disease [or other attribute] is absent

Confidence intervals are based on the Wilson procedure (Newcombe and Altman 2000: 46-47).

If the sample sizes on which sensitivity and specificity are based are entered, the sensitivity or specificity that is entered is revised, to make it consistent with a whole-number numerator, if the entered value differs from the sensitivity or specificity obtained by rounding its numerator (calculated from the entered value and the size of the sample in which it was determined) to the nearest integer. The revised value is the sensitivity or specificity computed from the rounded-off numerator.

Chance-corrected measures of sensitivity and specificity

The formulae for *chance-corrected measures of sensitivity (Se*) and specificity (Sp*)* (Brenner and Gefeller 1994) are:

$Se^* = 1 + [(Se - 1) / Sp]$, or $1 - LRN$
$Sp^* = 1 + [(Sp - 1) / Se]$, or $1 - 1 / LRP$

where Se and Sp = sensitivity and specificity (expressed as proportions).
LRN and LRP = likelihood ratios (see below) for, respectively, negative and positive results

Negative corrected values (indicating less than chance expectation) are reported as zero.

Confidence intervals are derived from the confidence intervals of the likelihood ratios.

Youden's index

The formula for *Youden's index* is

$Se + Sp - 1$

where Se and Sp = sensitivity and specificity (expressed as proportions).

A formula for its confidence interval is provided by Salmi (1986).

Diagnostic odds ratio

The formula is

$$Se * Sp / (1 - Se) / (1 - Sp), \text{ or } ad / bc.$$

The 95% confidence limit is estimated from the standard error of the log odds ratio (Newcombe and Altman 2000,: 60-62).

If a, b, c or d is zero, 0.5 is added to each before calculating rhe odds ratio and its standard error (formulae 5.20 and 5.33 of Fleiss 1981).

Likelihood ratios

For a positive result, the likelihood ratio is $Se / (1 - Sp)$
and for a negative result it is $(1 - Se) / Sp$
where *Se* and Sp = sensitivity and specificity (expressed as proportions).
Formulae for confidence intervals for the likelihood ratios are provided by Sullivan (1990). They use the standard error of the log of the ratio (formula 27 of Fleiss 1993).

Kullback-Leibler distances

Formulae are provided by Lee (1999).

Post-test probabilities and predictive value

The predictive value % of a positive test (the post-test probability % of the disease, after a positive result) is

$$(Se.DP) / [Se.DP + (1 - Sp)(1 - DP)] \text{ x } 100$$

and the predictive value % of a negative test (the post-test probability % of absence of the disease, after a negative result) is

$$(Sp.DP) / [(1 - Se)DP) + Sp(1 - DP)] \text{ x } 100$$

where *DP* is the disease prevalence (known or assumed), expressed as a proportion.
Se and *Sp* = sensitivity and specificity (expressed as proportions).

The post-test probability % of the disease after a negative result is

$$100 - \text{predictive value \% of a negative test.}$$

Formulae for confidence intervals for post-test probabilities are provided by Monsour *et al.* (1991).

Gain in certainty

Gain in certainty is computed by formulae provided by Connell and Koepsell (1985). Confidence intervals are estimated by substituting the confidence limits for the relevant post-test probability or Youden's index in the formulae for gain in certainty.

Kappa

The formula for alternative weighted *kappa*s is in the footnote to Table III of Kraemer *et al.* (2002).

Prevalence of the disease

The formula for p*revalence of the disease* (or other target attribute) (Rogan and Gladen 1978) is:

$$\text{Prevalence} = (t + Sp - 1) / (Se + Sp - 1)$$

where t = prevalence of positive tests
Se and *Sp* = sensitivity and specificity (expressed as proportions).
A computed prevalence of less than 0 is changed to 0, and one of more than 100% is changed to 100%.

Confidence intervals for the prevalence are based on the two variance formulae provided by Rogan and Gladen (1978: 75).

The *prevalence of true positive results* is computed as Prevalence x Sensitivity, and this is subtracted from the total prevalence of positive results to provide the *prevalence of false positive results*.

L2. COMPARISON OR USE OF TWO SCREENING OR DIAGNOSTIC TESTS

This module is applicable to “yes-no” screening and diagnostic tests for the presence or absence of a disease (or any other attribute) that have been appraised against a “gold standard” that defines the subject as a “case” or a “noncase”. It is applicable to tests that yield a range of results, if a single cutting-point is used. The program compares the tests, and appraises the validity of the combined tests. [The term “disease” may refer to whatever attribute the test or measure aims to indicate.]

The basic entries are the test results in samples of cases and noncases. If the tests were used in the same subjects, the paired results (e.g., “Test A positive, Test B negative”) are required.

If the tests were used in the same subjects and “gold standard” tests were used for all subjects, the prevalence of the disease in the target group or population can be entered (otherwise, the combined samples are taken to be representative of the target group). Also, the weight to be given to false negatives (FNs), relative to the weight given to false positives (FPs), can be entered. The weights express the relative undesirability (“costs”) of FNs and FPs. FNs should be given a large weight if the aim is to detect all cases, and a small weight if the aim is to establish a definitive diagnosis. If a weight is not entered, equal weight is given to FNs and FPs.

If the two tests were used in the same subjects and “gold standard” tests were used for all subjects, the program reports the **characteristics of the test** (sensitivity, specificity, false positive rate, Youden’s index, diagnostic odds ratios, likelihood ratios, and post-test probabilities) and provides a **comparison of sensitivities, false positive rates, and specificities.** Two indices that take account of the weights given to FNs and FPs are provided**: risk (“expected loss”) and *kappa,*** with significance tests for the differences between the tests. An **equivalence test** is offered, comparing the proportions of correct results in relation to the “gold standard”; this requires paired data (e.g., “Test A correct, Test B incorrect”); the bounds of “equivalence” must be defined, by specifying the largest difference that is to be regarded as negligible (e.g. 0.05). Sensitivity, specificity and post-test probability are computed for the **combined tests,** done in parallel or in either sequence.

If the two tests were used in the same subjects but “gold standard” tests were restricted to subjects with positive results, the program provides a **comparison of sensitivities, false positive rates, and specificities.** If these findings do not clearly indicate that one test is preferable, **FP:TP ratios** (false-positive:true-positive ratios**)** are provided. Relative sensitivity and the relative false positive rate are computed for the **combined tests,** done in parallel or in sequence.

If the two tests were used in different subjects, the **characteristics of the tests** that are reported are sensitivity, specificity,. chance-corrected sensitivity and specificity, Youden's index, diagnostic odds ratios, and likelihood ratios. The program provides a **comparison of sensitivities, false positive rates, and specificities.**

Characteristics of the tests

Sensitivity refers to the frequency of positive results in cases, and *specificit*y and the *false positive rate* refer to the frequency of negative and positive results, respectively, in noncases.

If the tests were appraised in different samples, sensitivity and specificity must be entered, and the program may revise the entered value to make it consistent with a whole-number numerator. This is done if the entered value differs from the sensitivity or specificity obtained by rounding its numerator (calculated from the entered value and the size of the sample in which it was determined) to the nearest integer.

"Chance-corrected" sensitivity and specificity (measures proposed by Brenner and Gefeller 1994) make allowance for the occurrence of chance agreement between the test result and the true status. It is the proportion of positive results, among cases, that can be attributed to the presence of the disease rather than to chance, chance agreement being estimated by the false positive rate. Similarly, chance-corrected specificity is the proportion of negative results, among noncases, that can be attributed to the absence of the disease rather than to chance (estimated by the false negative rate). A value below zero means that sensitivity or specificity is lower than might be expected by chance. These measures do not take disease prevalence into account.

Youden's index is the sum of sensitivity and specificity (expressed as percentages) minus 100. It has been termed the "per cent gain in certainty" (Connell and Koepsell 1985).

The *diagnostic odds ratio* is the ratio of the odds in favour of a positive result in cases to the odds in favour of a positive result in noncases. It is equivalent to the ratio of the likelihood ratios for positive and negative test results. A high odds ratio means that the odds of a positive test are relatively high in cases. An odds ratio of 1 means that the test has no discriminatory value

The *likelihood ratio* is the ratio of the prevalence of a specific result in cases to its prevalence in noncases. Likelihood ratios are calculated for positive and negative test results.

The *post-test probability* of the disease, which is conditional on the pre-test probability (i.e., the disease prevalence in the target group or population), refers to the probability if the test is positive (unless otherwise stated) or negative. The predictive value of a positive test, or positive predictive value, is the post-test probability of the disease; and the predictive value of a negative test, or negative predictive value, is the post-test probability of absence of the disease.

Comparison of sensitivities, false positive rates, and specificities.

If the tests were applied to the same subjects (and paired results are entered), the program computes *relative sensitivity, relative specificity, and the relative false-positive rate* - that is, the ratios of the values for Test A to the values for Test B, with 95% confidence intervals for the former two indices. A test that has a higher sensitivity than the other, without having a higher false-positive rate than the other, can be regarded as preferable (ignoring considerations of cost, convenience, etc.). Also, the program does *significance tests* for the differences between the sensitivities and between the specificities.

According to computer simulations (Cheng et al. 2000), the relative sensitivity estimate is unbiased if the true number of cases (diseased subjects) exceeds 30, and the relative false-positive rate if the

number of noncases exceeds 200. The confidence intervals are adequate if the number of cases or noncases exceeds 150.

If the tests were applied to different subjects, the program reports the differences between the two sensitivities, between the two specificities, between the two false-positive rates, and between the chance-corrected versions of the indices, with their 95% confidence intervals.

Risk ("expected loss") and *kappa*

If the two tests were used in the same subjects and "gold standard" tests were used for all subjects, two indices that take account of the weights given to FNs and FPs are provided (Bloch 1997): *risk* ("*expected loss*") and *kappa*, with significance tests for the differences between the tests.

"Risk" expresses the probability that a test will yield false results (positive or negative), whereas *kappa* expresses the test's desirable properties (a low probability of false results). *Kappa* ranges from 1 (no expected loss), through 0 (the loss expected by chance alone) to -1 (more loss than expected by chance).

Both indices apply to use of the test in a group or population with a specific defined disease prevalence; this might be 40%, for example, in a selected tertiary hospital population, and only 1% in a primary care setting. The assumed disease prevalence should be entered, unless the combined samples of cases and noncases are representative of the target population.

The comparisons based on the two indices may be expected to yield similar conclusions, unless the probabilities of positive findings are very different for the two tests. The significance tests are based on normal approximations, and should be treated with caution if samples are small; this applies especially to the tests for *kappa*.

FP:TP ratios

If the tests were applied to the same subjects, but "gold standard" tests were done only if results were positive, FP:TP ratios (false-positive:true-positive ratios) may be computed. They are not provided if a comparison of sensitivities and false positive rates clearly suggests that (ignoring considerations of cost, convenience, etc.) one test is superior to the other, e.g. with a higher sensitivity and a lower false positive rate, or if the total number of subjects tested is not entered.

The FP:TP ratio (Chock *et al.* 1997) is an estimate of the number of extra false positives that would be detected in a target population with a defined disease prevalence when the test with a higher sensitivity is used, for each extra true positive found, in comparison with the other test. Estimates are provided for disease prevalences of 50, 20, 10, 5 and 1 per 1000, and a formula is provided for making estimates for other prevalences.

The FP:TP ratio may be helpful if a test attains a higher sensitivity at the expense of a higher false-positive rate; the ratio then expresses the trade-off to be considered when deciding which test to use.

The estimates of FP:TP ratios assume that the tests are independent, and that all subjects who are negative on both tests are non-diseased.

Equivalence test

This test compares the two tests with respect to their proportions of results (positive or negative) that accord with the "gold standard" (Liu *et al.* 2002), after entry of the largest difference that is to be regarded as negligible (e.g. 0.05). Paired data are required (e.g., "Test A correct, Test B incorrect").

Two null hypotheses are tested. These are the hypotheses that there is more than a specified "negligible" difference in each direction – i.e., that the first proportion is more than negligibly higher than the first, and that the second is more than negligibly higher than the first. If both tests yield significant results, this supports the alternatives to the null hypotheses, namely that both these one-sided differences are negligible – that is, the proportions are equivalent. If only one test is significant, this indicates that one proportion is at least as high as the other. The larger of the two P values is displayed as the P value for the equivalence test (Liu *et al.* 2002).

Combined tests

If the two tests were used in the same subjects, the program appraises a combination of the tests.

If "gold standard" tests were used for all subjects, sensitivity, specificity and the post-test probability of positive results for a combination of the tests are computed, conditional on whether the overall result is seen as positive if either test is positive, or only if both tests are positive. These are the approaches sometimes termed "*SPIN*" ("*SP*ecific: *P*ositive result rules *IN* disease") or "Believe the positive", and "*SNOUT*" ("*S*e*N*sitive: *N*egative result rules *OUT* disease") or "Believe the negative". For the first approach, alternative results are presented, depending on whether both tests are always done, or whether performance of Test A depends on the result of Test B or *vice versa*. The second approach is conditional on performance of both tests

If "gold standard" tests were restricted to subjects with positive results, the program computes the relative sensitivity and relative false positive rate of the combined tests (taking the result as positive if either test is positive) , in comparison with each test separately. The results apply to performance of both tests in parallel, or to performance of both tests only if the first is negative.

METHODS

Characteristics of the tests

The formulae are:

Sensitivity = $a / (a + c)$

Chance-corrected sensitivity = $100 + [(Se - 100) / Sp]$

Specificity = $d / (b + d)$

Chance-corrected specificity = $100 + [(Sp - 100) / Se]$

False positive rate = $b / (b + d)$

Youden's index = $Se + Sp – 100$

Diagnostic odds ratio = $Se * Sp / (1 – Se) / (1 – Sp)$

Likelihood ratio for a positive result = $Se / (100 - Sp)$

Likelihood ratio for a negative result = $(100 - Se) / Sp$

Post-test probability (positive test) = $(Se.DP) / [Se.DP + (1 - Sp)(1 - DP)]$

Post-test probability (negative test) = $(Sp.DP) / [(1 - Se)DP) + Sp(1 - DP)]$

where *a* = positive result in subjects in whom the disease [or other attribute] is present
b= positive result in subjects in whom the disease [or other attribute] is absent
c = negative result in subjects in whom the disease [or other attribute] is present
d = negative result in subjects in whom the disease [or other attribute] is absent
S*e* and *Sp* = sensitivity and specificity (expressed as percentages).
DP is the disease prevalence per 100
Negative chance-corrected values (indicating less than chance expectation) are reported as zero.

If the tests were done in different samples, the sensitivity or specificity that is entered may be revised, to make it consistent with a whole-number numerator. This is done if the entered value differs from the sensitivity or specificity obtained by rounding its numerator (calculated from the entered value and the size of the sample in which it was determined) to the nearest integer. The revised value is the sensitivity or specificity computed from the rounded-off numerator.

Comparison of sensitivities, false positive rates, and specificities.

If the tests were done on the same subjects, McNemar tests are used to appraise their differences in sensitivity and specificity (Chock et al. 1997).

Relative sensitivity and the relative false positive rate, and their 95% confidence intervals, are computed by formulae 1 to 3 of Cheng et al. (2000). Relative specificity is derived from the relative false positive rate.

Confidence intervals for the differences in sensitivity, in false positive rates, and in specificity are based on the Wilson procedure (Newcombe and Altman 2000: 46-47).

Risk ("expected loss") and *kappa*

Formulae are provided by Bloch (1997).

Estimation and comparison of *risk* are based on formulae 3-8 if the combined samples are taken to be representative of the study population, and on formulae 9-15 if separate samples of cases and non cases are used.

Estimation and comparison of *kappa* are based on formulae 18-20 or (if separate samples of cases and non-cases are used) formulae 21-26. There are two misprints: in formula 25 the multiplier $(pi - 1 + r)/(1 - pi)$ should be changed to $(pi - 1 + r)/pi$; and in the formula for P2 (page 81), $Sp2$ should be changed to (1 - *Sp*2).

FP:TP ratios

FP:TP ratios for target populations with various prevalences are estimated by formula 3.9 of Chock et al. (1997. This formula requires estimates of the numbers of cases and noncases with two negative findings in the sampled population (d^* and D^*, respectively).

The number of cases with two negative findings is estimated by the formula

$$d^* = |b - c| / a$$

where *a* = cases with both tests positive
b = cases with only Test A positive
c = cases with only Test B positive.
This is based on the assumption that the two tests are independent when applied to the cases.

The number of noncases with two negative findings, D^*, is estimated by subtracting the sum total of subjects whose results are entered, plus d^*, from the total number of subjects tested.

Equivalence test

The program uses a test based on restricted maximum likelihood estimation (RMLE), without a continuity correction. This method, described by Nam (1997), has been evaluated and recommended by Liu *et al.* (2002), who explain how to replace the standard errors in the basic formulae (formulae 4 and 5) with RMLE-based values.

Combined tests

The relative sensitivity of the combined tests, in comparison to Test A or Test B, is calculated by dividing the number of true positives for the combined tests with the number of true positives for test A or B. Different numbers of true positives for the combined tests are used, depending on whether both tests are used or whether performance of the second test is conditional on the result of the first.

Relative false-positive rates are calculated in the same way, using the numbers of false positives.

L3. META-ANALYSIS OF STUDIES OF A "YES-NO" SCREENING OR DIAGNOSTIC TEST

This module is for use in meta-analyses of studies of the sensitivity and specificity of a "yes-no" screening or diagnostic test. It compares and combines the study findings. It can be applied to measures of the presence or absence of any attribute, not necessarily a disease, and can be used for a test that yields a range of results, if a selected cutting-point is used.

Optionally, the studies can be divided into categories, e.g. in accordance with differences in the test procedure, the characteristics of the subjects, or the quality of the study. The program then analyses each group of studies separately, as well as analysing the total set of studies. The program can also appraise the effect of a covariate on the test’s accuracy.

The required entries are sensitivity, specificity, and the sizes of the samples in which they were measured; that is, the number of "cases" (subjects who truly have the disease or attribute, according to some "gold standard") in whom sensitivity was measured, and the number of "noncases" in whom specificity was measured. Optional extras are the study category and a covariate.

The program compares the studies with respect to the test's **sensitivity**, **specificity**, **likelihood ratios** for positive and negative results, and **diagnostic odds ratio**. It displays forest plots for sensitivity, specificity, and the diagnostic odds ratio, permitting visual appraisal of heterogeneity, as well as providing tests and measures of heterogeneity for each index.

Overall values of the measures of test performance are computed, for use if the differences are deemed small enough to justify this. For sensitivity and specificity, it provides pooled and precision-weighted estimates; for likelihood ratios (for positive and negative results) it provides pooled, precision-weighted.,Mantel-Haenszel (fixed-effect) and Dersimonian-Laird (random-effects) estimates; and for diagnostic odds ratios it provides pooled, Mantel-Haenszel, and Dersimonian-Laird estimates.

The **relationship between sensitivity and specificity** is demonstrated in a scattergram, and a *summary ROC curve* (summarizing this relationship) is fitted to the data. The discriminative capacity of the test is summarized by the ***Q**** index, which is the value of sensitivity and specificity at the point where (according to the summary ROC curve) these two proportions are equal. If there are two or more groups containing 10 or more studies, the significance of differences between their *Q** values is tested.

To permit appraisal of **the effect of a covariate,** the program computes a linear regression equation that includes the covariate, and uses it to provide a graph comparing diagnostic odds ratios predicted from regression equations that do and do not include the covariate, as well as a graph comparing separate ROC curves computed for each study (taking the covariate into account).

Sensitivity and specificity

To permit appraisal of the heterogeneity of the sensitivities and specificities reported in different studies, the ranges of their values are reported, forest plots are displayed (see example, below), and *tests and measures of heterogeneity* (see below) are provided. The graphs show 95% confidence

intervals, and results based on small samples (under 30) and larger samples are shown in different colours. Here is an example:

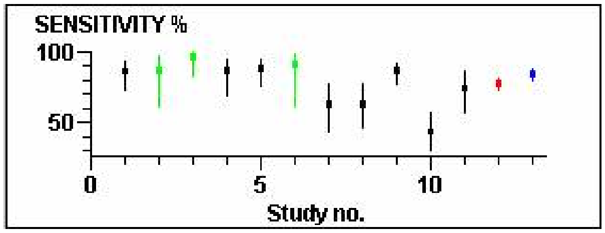

Optionally, a list of sensitivities and specificities with their 95% confidence limits is displayed. The sensitivities and specificities may differ slightly from the entered values, as the program recalculates them to reduce the effect of rounding-off.

Two overall summary sensitivity and specificity values are computed and displayed in the graphs (on the right, in red and blue), with their 95% confidence intervals. One overall value is obtained by pooling the studies' results (which is equivalent to weighting them by sample size), and the other by weighting them by the inverse of their variances (precision-weighting).

The overall values may be useful if there is little heterogeneity, but otherwise their appropriateness has been questioned, especially in view of the possibility that different studies may use different criteria (e.g., different degrees of abnormality) for the presence of the disease or other attribute under study (Irwig *et al.* 1995). Sensitivity and specificity are generally inversely correlated, and meta-analytic procedures that take account of both sensitivity and specificity, such as the use of likelihood ratios, diagnostic odds ratios, or a summary ROC curve, are recommended.

Relationship between sensitivity and specificity

The program reports the correlation between sensitivity and specificity (Spearman's *rho*), and provides a scattergram (see example, below) demonstrating the relationship between sensitivity and the false positive rate (100% minus specificity). In the scattergram, results based on samples of different sizes are shown in different colours.

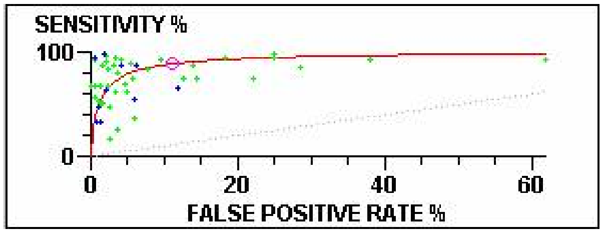

A *summary ROC curve* is superimposed on the scattergram. This is a curve, fitted to the data, that demonstrates the trade-off between sensitivity and specificity. It is based on the principle that there is a linear relationship between the logit of sensitivity and the logit of the false positive rate; the program uses an equation based on ordinary unweighted least-squares regression (Moses *et al.* 1993), a method that generally provides similar (but not identical) results to those using other regression equations; the unweighted analysis has the disadvantage that more importance is not given to larger studies, but it avoids a possible bias of a weighted analysis, which tends to give more weight to less to studies reporting less accuracy (Irwig *et al.* 1995). The regression

coefficients and their standard errors are reported, and the significance of the *beta* (slope) coefficient is tested.

The closer the curve comes to the top left corner of the graph, where both sensitivity and specificity are 100%, the better the test. For comparison, the graph also displays a dotted line representing the curve of a completely nondiscriminatory test. A poor test will have a ROC curve close to this line.

The ROC curve is a useful way of combining heterogeneous test results, on the assumption that the differences are due to variation in the thresholds used to define positive and negative results; unless this is so, its advantage is open to question (Deeks 2001a).

*Q** index

The *Q** index, which is the value of sensitivity and specificity at the point where (according to the summary ROC curve) these two proportions are equal, may be used as a summary measure of the discriminative capacity of the test (Moses *et al*. 1993: appendix). Its value and estimated standard error are reported, and it is shown in the scattergram, as a small circle (see example, above). Unless the ROC curve is anomalous, Q* is the point at which the curve shoulders most closely to the desirable top left corner, although this may not be readily apparent because of the use of different scales in the X and Y axes of the graph.

Q* should be used with caution, since in some situations it may lead to misinterpretation (Stengel *et al.* 2003); it hardly distinguishes highly sensitive but unspecific tests from worthless procedures, and tests with poor sensitivity may yield similar Q* values, regardless of their specificity.

If there are two or more groups containing 10 or more studies, the significance of the differences between their *Q** values is tested.

Likelihood ratios

The likelihood ratio is the ratio of the probability of a specific result in cases to its probability in noncases. The program computes likelihood ratios for positive and negative tests (positive and negative likelihood ratios) for each study, and reports the overall ranges. Tests and measures of heterogeneity (see below) are provided. Optionally, a list of the positive and negative likelihood ratios is displayed.

Four overall summary measures are reported for each likelihood ratio, with their 95% confidence intervals. Their use may be considered justifiable if there is little heterogeneity.

The first overall value is obtained by simply pooling the studies' primary results; the second is based on precision-weighting (weighting by the inverse of the variance), and is not recommended if there are studies with small numbers of subjects or if the likelihood ratio is close to zero (Lui 2004: 69); the third is a Mantel-Haenszel (fixed-effect) estimate, and the fourth is a Dersimonian-Laird (random-effects) estimate. The fixed-effect estimate is based on a homogeneity assumption, whereas the random-effects estimate takes account of unexplained sources of between-study heterogeneity. But in situations where the random-effects estimate leads to important changes in inferences, there is often so much heterogeneity that the value of both the fixed-effect and random-effects estimates is questionable (Rothman and Greenland 1998: 667).

A likelihood ratio greater than 10 or less than 0.1 indicates that the test will provide convincing diagnostic evidence, and a value greater than 5 or below 0.2 provides strong diagnostic evidence, whereas if the likelihood ratio is between 0.5 and 2 the test has little or no effect on the certainty of diagnosis (Jaeschke *et al.* 1994).

Diagnostic odd ratios

The diagnostic odds ratio is a measure of a test’s discriminatory power that takes account of both sensitivity and specificity, but without distinguishing between the effects of sensitivity and specificity. It is the ratio of the odds in favour of a positive result in subjects with the disease (or other attribute) to the odds in favour of a positive result in subjects without the disease, and is equivalent to the ratio of the likelihood ratios for positive and negative test results. An odds ratio of 1 means that the test has no discriminatory value; the further the value is from 1 (in either direction), the more its discriminatory capacity.

The program computes the diagnostic odds ratio for each study, reports the overall ranges, displays a forest plot (see example, below), and provides *tests and measures of heterogeneity.* Optionally, a list of the diagnostic odds ratios is displayed. The graphs show 95% confidence intervals, and results based on samples of different sizes are shown in different colours. Mantel-Haenszel and random-effects estimates are shown in red and blue.

Reasonable consistency of the diagnostic odds ratios suggests that they could have originated from the same ROC curve (Deeks 2001a).

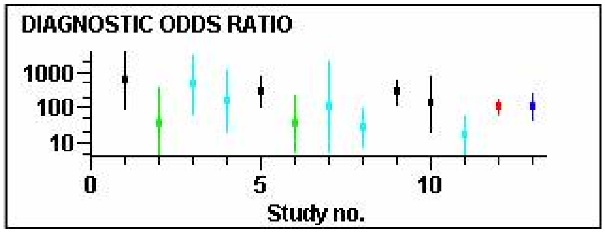

Effect of a covariate

The program permits entry of a covariate that may influence the test’s accuracy. This might be (or example) a numerical measure of some characteristic of the subjects or of the quality of the study. The program then fits a regression equation that includes the covariate to the data (using least-squares regression, without weighting). This equation is used to predict a diagnostic odds ratio for each study, for comparison with the diagnostic odds ratio predicted from a regression equation that does not include the covariate. The exponential of the regression coefficient for the covariate is reported, as an indication of the multiplicative effect of the covariate on the diagnostic odds ratio (Deeks 2001b) .

The pairs of odds ratios are shown in a graph; if the values do not coincide, as in the following figure, this indicates that the covariate affects the test’s accuracy. The odds ratios based on models that do and do not include the covariate are shown in purple and yellow respectively. The graph also shows the observed diagnostic odds ratios (colour-coded according to sample size) if they differ from the predicted values, and (on the right) Mantel-Haenszel and random-effects estimates.

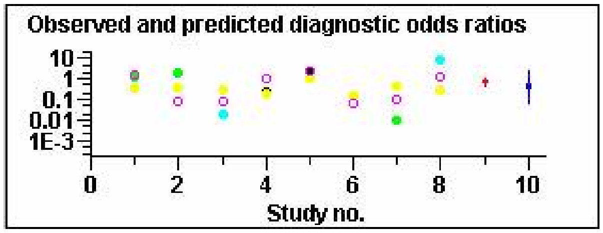

As suggested by Moses *et al.*(1993), the regression equation is also used as a basis for a separate ROC curve for each study, taking account of the value of the covariate in the study together with the information in the summary ROC curve. These curves, which are displayed in a graph (colour-coded in accordance with sample size), will be close or superimposed if the covariate has little or no effect on accuracy. In the following example, the covariate is seen to have a strong effect.

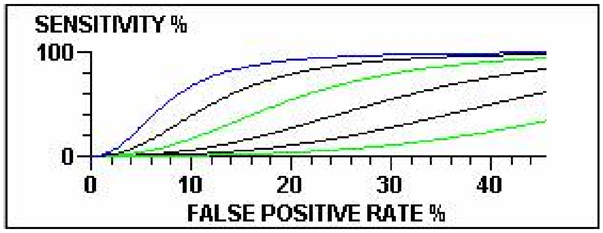

Heterogeneity tests and measures

The heterogeneity tests (for sensitivity, specificity, likelihood ratios, and diagnostic odds ratios) should be interpreted with caution, since their power is low; if the result is significant at the 0.05 level, the hypothesis of homogeneity can be rejected; but "a high p-value ... does not show that the measure is uniform, it only means that heterogeneity ... was not detected by the test" (Rothman and Greenland 1998: 276).

Two measures of heterogeneity are provided: H and I-squared, with their approximate 95% intervals. An H value of less than 1.2 suggests absence of noteworthy heterogeneity, whereas a value exceeding 1.5 suggests its presence, even if the heterogeneity test is not significant. I-squared expresses the proportion of variation that can be attributed to heterogeneity rather than to sampling error.

Graphs

The three forest plots and the scattergram described above are displayed for the total set of studies and (if the studies have been grouped) for each group of studies. The graphs comparing predicted diagnostic ratios and comparing ROC curves are displayed only if a covariate has been entered.

It is not possible to "zoom" or read the values in these graphs by clicking. The graphs can be printed, copied to the clipboard for pasting elsewhere, or saved in bitmap (.BMP) files.

A graph may disappear if the program is minimized and then restored. To recover it, click on "Next graph" or "Back", and then return to the required graph.

METHODS

To remove the effect of rounding-off of the entered sensitivities and specificities, they are recalculated after rounding their numerators (calculated from the entered data) off to the nearest integer.

Sensitivity and specificity

Confidence intervals for the individual sensitivities and specificities, and for the overall values calculated from the studies' pooled numbers of true and false positives and negatives, are computed by the Wilson score-test method (Wilson 1927), as described by Newcombe and Altman (2000:46-7).

The precision-weighted overall values are calculated by the formula

$$P_w = \Sigma (W_i . P_i) / \Sigma W_i$$

and its 95% confidence intervals are

$$P_w - (1.96 / \sqrt{\Sigma W_i}) \text{ and } P_w + (1.96 / \sqrt{\Sigma W_i})$$

where $W_i = 1$ / variance of $P_i = N_i / P_i Q_i$

$P_i =$ sensitivity $= a / (a + c)$ (calculated after adding 0.5 to a and c if either is 0)

or specificity $= b / (b + d)$ (calculated after adding 0.5 to b and d if either is 0)

$Q_i = 1 - \text{Pi}$

a = positive result in subjects in whom the disease [or other attribute] is present
b = positive result in subjects in whom the disease [or other attribute] is absent
c = negative result in subjects in whom the disease [or other attribute] is present
d = negative result in subjects in whom the disease [or other attribute] is absent
$N_i = a + c$ (for sensitivity) or $b + d$ (for specificity).

Heterogeneity is tested by contingency table analysis (Zar 1998; 488-489). If the sensitivities or specificities are all zero, or al 100, 0.0000001 is added to a zero denominator to avoid division by zero.

The correlation between sensitivity and specificity is measured by Spearman’s rank correlation coefficient *rho*, allowing for ties (Siegel and Castellan 1988: 235-244), using an adaptation of the SPEAR procedure in Press *et al.* 1989: 538-539. If there are 30 or fewer studies, the significance of rho is appraised by the use of critical levels for one-tailed P = 0.10, 0.05, 0.025, 0. 01, 0.005, and 0.001 (Siegel and Castellan 1988: Table Q). If there are over 30 numbers, a t-test is used (Siegel and Castellan 1988: 243, footnote; Press *et al.* 1989: formula 13.8.2, p. 537).

Summary ROC curve and *Q**

The summary ROC curve is based on an ordinary least-squares regression equation (Irwig *et al.* 1995: formula 1). . Before computation, 0.5 is added to each of the four cells in the table to deal with the possibility of zero cells, as suggested by(Moses *et al.* (1993 and Deville *et al.* (2002); this correction biases the curve conservatively, reducing the test's discriminatory capacity. In rare instances, the ROC curve cannot be computed..

The regression equation is:

$$D = \alpha + \beta S$$

where D = log(diagnostic odds ratio) = logit(TPR) - logit(FPR)
S = logit(TPR) + logit(FPR)
TPR = sensitivity
FPR = false positive rate
α, β = regression coefficients

Using the regression coefficients, a value of TPR is then computed for each value of FPR (Moses *et al.* 1993: formula 1), and the results are plotted on a graph whose axes are sensitivity and the false positive rate. The significance of β (the slope coefficient) is tested by formula 7.18 of Armitage *et al.* (2003).

The ROC curve does not extend beyond the highest observed false positive rate.

*Q** and its standard error are computed by the formulae provided by Moses *et al.* (1993: 1314)

Likelihood ratios

The likelihood ratios for a positive test (*LRP*) and for a negative test *(LRN*) are ratios of proportions, and the estimation of confidence intervals for overall values and heterogeneity tests are based on procedures applicable to ratios of proportions (risk ratios).

LRP = sensitivity / (1 - specificity)

LRN = (1 - sensitivity) / specificity

If sensitivity or specificity is 0 or 100%, 0.5 is added to the numbers of positive and negative tests before computation.

The *pooled LRP and LRN* are based on the totality of the cumulated primary results.
Their 95% confidence intervals use the log of the likelihood ratio (Fleiss 1993, formula 29-35; Lui 2004, formula 4.2):

$$95\%\ \text{CI} = \exp(\log(LR) - 1.96S) \text{ to } \exp(\log(LR) + 1.96S)$$

where S = standard error of $\log(LR) = \sqrt{}[1 / a - 1 \ /(a + c) + 1 / \text{b} - 1 / (b + d)]$

$LR = LRP$ or LRN
a = true positives (for *LRP*) or false negatives (for *LRN*)
b = false positives (for *LRP*) or true negatives (for *LRN*)
c = false negatives (for *LRP*) or true positives (for *LPN*)
d = true negatives (for *LRP*) or false positives (for *LRN*)

For the *precision-weighted estimates* and their confidence intervals, the logs of each study's LR values are weighted by the inverse of their variances (Fleiss 1993, formulae 1, 2, 4; Lui 2004, formula 4.9). The *Mantel-Haenszel estimates* of the overall likelihood ratios are computed by formulae 4.10 to 4.12 of Lui (2004), and the *Dersimonian-Laird estimate* by the formulae provided by Deeks (1999) and Deeks *et al.* (2001).

The *heterogeneity tests* are based on a comparison with the Mantel-Haenszel estimate (Deeks 1999, Deeks *et al*. 2001), and use the adjustment suggested by Lipsitz *et al.* (1998) (formula 4.14 of Lui (2004). The heterogeneity index and *I-squared* are based on the unadjusted test statistic (Lui 2004: formula 4.13).

Diagnostic odds ratios

The diagnostic odds ratio is each study is calculated as ad / bc
where a = positive results in cases, plus 0.5
b= positive results in noncases, plus 0.5
c = negative results in cases, plus 0.5
d = negative results in noncases, plus 0.5
Its standard error and 95% confidence interval are estimated by the logit method (Morris and Gardner 2000: 61).

The *pooled diagnostic odds ratio* is based on the totality of the cumulated data. The *Mantel-Haenszel estimate* and its 95% confidence interval are computed by formulae provided by Deeks (1999) and Deeks *et al.* (2001), and the *Dersimonian-Laird estimate* and its 95% confidence interval by formulae 70-79 of Fleiss (1993). *Heterogeneity* is tested by formula 22 of Fleiss (1993).

Effect of a covariate

The regression equation that includes the covariate (Moses *et al*. 1993) is:

$$D = \alpha + \beta S + \gamma C$$

where D = log(diagnostic odds ratio) = logit(TPR) - logit(FPR)
S = logit(TPR) + logit(FPR)
C = covariate
TPR = sensitivity
FPR = false positive rate
α, β, γ = regression coefficients
Before computing TPR and LPR, 0.5 is added to each of the four cells in the table.
The equation is computed by an adaptation of Abdusamad Salih's Fortran MULREG multiple-regression program (Salih 2003), and standard errors of the regression coefficients by formula 17.5.3 of Snedecor and Cochran (1980).

Using this equation, a ROC curve is computed for each study, as described by Moses *et al*. (1993: 1310). A value of *TPR* is computed for each value of *FPR* (Moses *et al*. 1993: formula 1), substituting $(\alpha + \gamma C)$ for A, and β for B, in the

formula, and the results are plotted on a graph whose axes are sensitivity and the false positive rate. Occasionally the curves are not shown because the computation requires logs of a negative number.

Heterogeneity measures

The *measures of heterogeneity*, *H* and *I-squared*, are described by Higgins and Thompson (2002). *H* is computed by Higgins and Thompson's formula 6, and increased to 1, indicating absence of heterogeneity, if it less than 1. A test-based interval is computed by Method III. *I-squared* and its 95% interval are computed from *H*, by formula 10.

REFERENCES

Abramson JH (1885) Age-standardization in epidemiological data. International Journal of Epidemiology 24: 238-239.

Abramson JH, Gahlinger PM (2001) Computer programs for epidemiologists: PEPI version 4. Sagebrush Press: Salt Lake City.

Agresti A, Coull BA (1998) Approximate is better than "exact" for interval estimation of binomial proportions. The American Statistician 52: 119-126.

Ahmad OB, Boschi-Pinto C, Lopez AD, Murray CJL, Lozano R, Inoue M. (1976) Age standardization of rates: a new WHO standard. Global Programme on Evidence for Health Policy Discussion Paper Series: No. 31. World Health Organization. Available on Internet at www3.who.int/whosis/discussion_papers/pdf/paper31.pdf

Altman DG (1991) Practical statistics for medical research. London: Chapman and Hall.

Altman DG, Machin D, Bryant TN, Gardner MJ (2000) Statistics with confidence, 2nd edn. BMJ Books.

Anderson S, Auguier WW, Hauck WW, Oakes D, Vandaele W, Weisberg HI (1980) Statistical methods for comparative studies. New York, Wiley.

Armitage P, Berry G, Matthews JNS (2003) Statistical methods in medical research, 4th edn. Oxford: Blackwell Science.

Axtell LM (1963) Computing survival rates for chronic disease patients. JAMA 186:1125-1128.

Bartholomew DJ (1959a) A test of homogeneity for ordered alternatives. Biometrika 46: 36-48.

Bartholomew DJ (1959b) A test of homogeneity for ordered alternatives. II. Biometrika 46: 328-335.

Bennett S, Woods T, Liyanage WM, Smith DL (1991) A simplified general method for cluster-sample surveys of health in developing countries. World Health Statistics Quarterly 44:98-106.

Bloch DA (1997) Comparing two diagnostic tests against the same “gold standard” in the same sample. Biometrics 53: 73-85.

Bloch DA, Kraemer HC (1989) 2 x 2 kappa coefficients: measures of agreement or association. Biometrics 45: 269-287.

Brenner H, Gefeller O (1994) Chance-corrected measures of the validity of a binary test. Journal of Clinical Epidemiology 47: 627-633.

Breslow NE, Day NE (1980) Statistical methods in cancer research. vol. I. The analysis of case-control studies. Lyon: International Agency for Research on Cancer.

Breslow NE, Day NE (1987) Statistical methods in cancer research, vol. II. The design and analysis of cohort studies. Lyon: International Agency for Research on Cancer.

Brown LD, Zhao L H (2002) A test for the Poisson distribution. The Indian Journal of Statistics 64 (series A): 611-625.

Brownlee KA (1965) Statistical theory and methodology in science and engineering, 2nd edn. New York: John Wiley & Sons.

Burstein H (1975) Finite population correction for binomial confidence limits. Journal of the American Statistical Association 70: 67-69.

Campbell MJ, Gardner MJ (2000) Medians and their differences. In: Altman DG, Machin D, Bryant TN, Gardner MJ, eds (2000) Statistics with confidence, 2nd edn. BMJ Books, pp 36-44.

Chao A (1987) Estimating the population size for capture-recapture data with unequal catchability. Biometrics 43: 783-791.

Chao A, Tsay PK (1998) A sample coverage approach to multiple-system estimation with application to census undercount. Journal of the American Statistical Association 93: 283-293.

Chao A, Tsay PK, Lin S-H, Shau W-Y, Chao DY (2001) Tutorial in biostatistics: The applications of capture-recapture models in epidemiological data. Statistics in Medicine 30: 3123-3157.

Chao A, Tsay PK, Shau W-Y, Chao DY (1996) Population size estimation for capture-recapture models with application to epidemiological data. Proceedings of Biometrics Section, American Statistical Association, pp 108-117.

Chapman RM (1948) A mathematical study of confidence limits of salmon populations calculated from sample tag ratios. International Pacific Salmon Fisheries Commission Bulletin 2: 69-85.

Chapman RM (1951) Some properties of the hypergeometric distribution with applications to zoological sample censuses. University of California Publication in Statistics, 1: 131-159.

Cheng H, Macaluso M, Hardin JM (2000) Validity and coverage of estimates of relative accuracy. Annals of Epidemiology. 2000;10(4):251-260.

Chock C, Irwig L, Berry G, Glasziou P (1997) Comparing dichotomous screening tests when individuals negative on both tests are not verified. Journal of Clinical Epidemiology 50: 1211-1217.

Choi BC, de Guia NA, Walsh P (1999) Look before you leap: stratify before you standardize. American Journal of Epidemiology 149: 1087- 1096.

Cochran WG (1977) Sampling techniques, 3rd edn. New York: John Wiley & Sons.

Cochrane D, Orcutt GH (1949) Application of least squares regression to relationships containing autocorrelated error terms. Journal of the American Statistical Association 44: 32-61.

Cohen G, Yang S-Y (1994) Mid-P confidence intervals for the Poisson expectation. Statistics in Medicine 13: 2189-2203.

Connell FA, Koepsell TD (1985) Measures of gain in certainty from a diagnostic test. American Journal of Epidemiology 121: 744-753.

Cook I (1987) Estimation of the median from grouped data. Teaching Statistics 9: 26-29.

Cox DR, Stuart A (1955) Some quick tests for trend in location and dispersion. Biometrika 42: 80-95.

Cressie N, Read TRC (1984) Multinomial goodness-of-fit tests. Journal of the Royal Statistical Society, Series B, 46: 440-464.

Cuzick J (2000) Clustering. . In: Encyclopedia of epidemiologic methods (Gail MH, Benichou J, eds.), Chichester: Wiley, pp 192-201.

.D'Agostino RB (1986) Tests for the normal distribution. In: D'Agostino RB, Stephens MA (eds) Goodness-of-fit techniques. New York: Marcel Dekker, pp 367-419.

D'Agostino RB, Pearson ES (1973) Tests of departure from normality: empirical results for the distribution of b2 and ub1. Biometrika 60: 613-622.

Daniel WW (1995) Biostatistics: a foundation for analysis in the health sciences, 6th edn. New York: John Wiley & Sons.

Day NE (1976) A new measure of age standardized incidence, the cumulative rate. In: Waterhouse JAH, Muir CS, Correa P, Powell J (eds.) Cancer incidence in five continents, vol. III. Lyon: International Agency for Research on Cancer, pp 443-452.

Deeks J (1999) Statistical methods programmed in MetaView. Oxford, Cochrane Library. Internet document http://www.a3.san.gva.es/mbe/statisticalmethods4.pdf

Deeks J (2001a) Systematic reviews of evaluations of diagnostic and screening tests. British Medical Journal 323: 157-162.

Deeks J (2001b) Systematic reviews of evaluations of diagnostic and screening tests. In: Systematic reviews in health care: Meta-analysis in context, 2nd edn. London: BMJ Books pp 248-202.

Deeks JJ, Altman DG, Bradburn MJ (2001) Statistical methods for examining heterogeneity and combining results from several studies in meta-analysis. In: Systematic reviews in health care: Meta-analysis in context, 2nd edn. London: BMJ Books pp 285-312.

Deville WI, Buntinx F, Bouter LM, Montori VM, de Vet HCW, van der Windt DAWM, Bezemer PD (2002) Conducting systematic reviews of diagnostic studies: didactic guidelines. BMC Medical Research Methodology 2: 9.

Diem K (1970) Documenta Geigy: Scientific Tables, 7th edn. Basle: J.R.Geigy S.A.

Dobson AJ, Kuulasma K, Eberle E, Scherer J (1991) Confidence intervals for weighted sums of Poisson parameters. Statistics in Medicine 10:457-462.

Durbin, J. and G.S. Watson (1951), Testing for serial correlation in least squares regression II, Biometrika, 38: 159-178.

Edwards JH (1961) Seasonal incidence of congenital disease in Birmingham. Annals of Human Genetics 25: 89-93.

Fieller EC, Hartley HO, Pearson ES (1957) Tests for rank correlation coefficients. I. Biometrika 44: 470-481.

Fieller EC, Hartley HO, Pearson ES (1961) Tests for rank correlation coefficients. II. Biometrika 48: 29-40.

Fleiss JL (1981) Statistical methods for rates and proportions, 2nd edn. New York: John Wiley & Sons.

Fleiss JL (1993) The statistical basis of meta-analysis. Statistical Methods in Medical Research 2: 121-145.

Fleiss JL, Levin B, Paik MC (2003) Statistical methods for rates and proportions, 3rd edn. Hoboken, New Jersey: John Wiley & Sons.

Freedman LS (1979) The use of a Kolmogorov-Smirnov type statistic in testing hypotheses about seasonal variation. Journal of Epidemiology and Community Health 33:223-228.

George VT, Elston RC (1993) Confidence intervals based on the first occurrence of an event. Statistics in Medicine 12: 685-690.

Ghent A W (1993) An exact test and normal approximation for centrifugal and centripetal patterns in line and belt transects in ecological studies. American Midland Naturalist 130: 338-355.

Grubbs, Frank (1969), Procedures for detecting outlying observations in samples, Technometrics 11 [1]: 1-21.

Hartwig F, Dearing, BE (1979) Exploratory data analysis. Beverly Hills, Calif.: Sage Publications.

Healey JG (1984) Statistics: a tool for social research. Belmont, CA: Wadsworth.

Helsel DR, Hirsch RM (2002) Statistical methods in water resources. U.S. Department of the Interior, U.S. Geological Survey, Techniques of Water-Resources Investigations Book 4, Chapter A3. Internet document http://water.usgs.gov/pubs/twri/twri4a3/pdf/chapter12.pdf

Hewitt D, Milner J, Csima A, Pakula A (1971) On Edwards' criterion of seasonality and a non-parametric alternative. British Journal of Preventive and Social Medicine 25: 174-176.

Higgins JPT, Thompson SG (2002) Quantifying heterogeneity in a meta-analysis. Statistics in Medicine 21: 1539-1558.

Hill C, Benhamou E (1995) Age-standardization in epidemiological data. International Journal of Epidemiology 24: 238-242.

Hirsch RM, Slack JR, Smith RA (1982). Techniques of trend analysis for monthly water quality data. Water Resource Research 18: 107-121.

Hollander M, Wolfe DA (1999) Nonparametric statistical methods, 2nd edn. New York: John Wiley & Sons.

Inskip H (2000) Standardization methods. In: Encyclopedia of epidemiologic methods (Gail MH, Benichou J, eds.), Chichester: Wiley, pp 871-884.

Irwig L, Macaskill P, Glasziou P, Fahey M (1995) Meta-analytic methods for diagnostic test accuracy. Journal of Clinical Epidemiology 48: 119-130.

Jaeschke R, Guyatt GH, Sackett DL (1994). Users' guides to the medical literature. III. How to use an article about a diagnostic test. Journal of the American Medical Association 271: 703-709.

Johnston J, DiNardo J (1997) Econometrics methods, 4th edn, McGraw-Hill.

Kaplan EL, Meier P (1958) Nonparametric estimation from incomplete observations. Journal of the American Statistical Association 53:457-481.

Knox EG (1964) The detection of space-time interactions. Applied Statistics 13:25-29.

Kraemer HC, Periyakoil VS, Noda A (2002) Tutorial in biostatistics: kappa coefficients in medical research. Statistics in Medicine 21: 2109-2129.

Lee J (1993) Sample size and power considerations to procure the required number of subjects with a certain attribute. British Journal of Industrial Medicine 50:765.

Lee W-C (1999) Selecting diagnostic tests for ruling out or ruling in disease: the use of the Kullback-Leibler distance. International Journal of Epidemiology 28: 521-525.

Lipsitz SR, Dear KBG, Laird NM, Molenberghs G (1998). Tests for homogeneity of the risk difference when data are sparse. Biometrics 54: 148-160.

Lilliefors HW (1967) On the Kolmogorov-Smirnov test for normality with mean and variance unknown. Journal of the American Statistical Association 62:399-402.

Liu J-P. Hsueh H-M. Hsieh E. Chen JJ (2002) Tests for equivalence or non-inferiority in paired binary data. Statistics in Medicine 21: 231-245.

Lui K-J (2004) Statistical evaluation of epidemiological risk. Chichester: John Wiley & Sons.

Machin D, Gardner MJ (2000) Time to event studies. In: Altman DG, Machin D, Bryant TN, Gardner MJ, eds (2000) Statistics with confidence, 2nd edn. BMJ Books, pp 93-194.

Mann HB (1945) Nonparametric tests against trend. Econometrica 13: 245-259.

Maxwell AE (1961) Analysing qualitative data. London: Methuen.

McBride G (2000) Anomalies and remedies in non-parametric seasonal trend tests and estimates. Internet document www.niwa.cri.nz/rc/prog/stats/anom.pdf

Mehta C, Patel N (1991) StatXact statistical software for exact nonparametric inference: User Manual Version 2. Cambridge MA: Cytel Software Corporation.

Miller LH (1956) Table of percentage points of Kolmogorov statistics. Journal of the American Statistical Association 51: 111-121.

Monsour MJ, Evans AT, Kipper LL (1991) Confidence intervals for post-test probability. Statistics in Medicine 10:443-456.

Morris JA, Gardner MJ (2000) Epidemiological studies. In: Altman DG, Machin D, Bryant TN, Gardner MJ, eds (2000) Statistics with confidence, 2nd edn. BMJ Books, p. 69.

Moser EB (2002) Fundamental sampling techniques; stratified random sampling. Baton Rouge, Louisiana: Department of Experimental Statistics, Louisiana State University. Internet document: http://www.stat.lsu.edu/faculty/moser/exst7012/strata.pdf

Moses LE, Shapiro D, Littenberg B (1993) Combining independent studies of a diagnostic test into a summary ROC curve: data-analytic approaches and some additional considerations. Statistics in Medicine 12: 1293-1316.

Nam J-M (1997) Establishing equivalence of two treatments and sample size requirements in matched-pairs design. Biometrics 53: 1422-1430.

Newcombe RG, Altman DG (2000) Proportions and their differences. . In: Altman DG, Machin D, Bryant TN, Gardner MJ, eds (2000) Statistics with confidence, 2nd edn. BMJ Books, pp 45-56.

Pearson ES, Hartley HO (1966) Biometrika tables for statisticians, Vol. 1, 3rd edn. Cambridge: Cambridge University Press.

Pocock SJ (1974) Harmonic analysis applied to seasonal variations in sickness absence. Applied Statistics 23: 103-120.

Press WH, Flannery BP, Teukolsky SA, Vetterling WT (1989) Numerical recipes in Pascal: The art of scientific computing. Cambridge: Cambridge University Press.

Randles RH, Fligner MA, Policello GE III, Wolfe DA (1980) An asymptotically distribution-free test for symmetry versus asymmetry. Journal of the American Statistical Association 75: 168-172.

Ridout MS, Demetrio CGB, Firth D (1999) Estimating intraclass correlation for binary data. Biometrics 55: 137-148.

Rogan WJ, Gladen B (1978) Estimating prevalence from the results of a screening test. American Journal of Epidemiology 107:71-76.

Roger JH (1977) A significance test for cyclic trends in incidence data. Biometrika 64(1): 152-156.

Rogerson PA (1996) A generalization of Hewitt's test for seasonality. International Journal of Epidemiology 25: 644-648.

Rothenberg RB, Lobanov A, Singh KB, Stroh G Jr (1985) Observations on the application of EPI cluster survey methods for estimating disease incidence. Bulletin of the World Health Organization 63: 93-99

Rothman KJ (1978) Estimation of confidence limits for the cumulative probability of survival in life table analysis. Journal of Chronic Diseases 31:557-560.

Rothman KJ (1986) Modern Epidemiology. Boston: Little, Brown & Co.

Rothman KJ, Boice JD Jr (1982) Epidemiological analysis with a programmable calculator. Boston: Epidemiology Resources Inc.

Rothman KJ, Greenland S (1998) Modern epidemiology, 2nd edn. Philadelphia: Lippincott-Raven.

Salih A (2003) Numerical recipes in Fortran. Internet document: http://www.sali.freeservers.com/engineering/fortran.html#recipe

Salmi LR (1986) Re: Measures of gain in certainty from a diagnostic test (letter). AmericanJournal of Epidemiology 123: 1121-1122.

Salmi T, Maatta A, Anttila P, Ruoho-Airola T, Amnell T (2002). Detecting trends of annual values of atmospheric pollutants by the Mann-Kendall test and Sen's slope estimate - the Excel Template Application Makesens. Publications on air quality no. 31, Finnish Meteorological Institute, Helsinki. Internet document www.emep.int/assessment/MAKESENS_MANUAL.pdf

Seber GAF (1982) The estimation of animal abundance and related parameters, 2nd edn. London: Charles Griffin & Co.

Selvin S (1996) Statistical analysis of epidemiologic data, 2nd edn. New York: Oxford University Press.

Sen PK (1968) Estimates of the regression coefficient based on Kendall's tau. Journal of the American Statistical Association 63: 1379-1389.

Siegel S, Castellan NJ Jr (1988) Nonparametric statistics for the behavioural sciences, 2nd edn. New York: McGraw Hill International.

Silcocks P (1994) Estimating confidence limits on a standardized mortality ratio when the expected number is not error free. Journal of Epidemiology and Community Health 48: 313-317.

Snedecor GW, Cochran WG (1980) Statistical methods, 7th edn. Iowa State University Press, Ames, Iowa.

Sprent P (1993) Applied nonparametric statistical methods, 2nd edn. London: Chapman & Hall

SPSS (2003) AREG. Internet document www.spss.com/tech/stat/algorithms/11.0/areg.pdf

Stengel D, Bauwens K, Sehouli J, Ekkernkamp A, Porzsolt F (2003) A likelihood ratio approach to meta-analysis of diagnostic tests. Journal of Medical Screening 10: 47-51.

Sullivan, KM (1990) Documentation of EPI_PAK program. Division of Nutrition, Centers for Disease Control, Atlanta, GA.

Taube A (1986) Sensitivity, specificity and predictive values: a graphical approach. Statistics in Medicine 5: 585-591.

Theil H (1950) A rank-invariant method of linear and polynomial regression analysis. III. Koninklijke Nederlandse Akademie Van Wetenschappen, Proceedings, Series A, 53: 1397-1412.

Vaeth M (2000) Expected number of deaths. In: Encyclopedia of epidemiologic methods (Gail MH, Benichou J, eds.), Chichester: Wiley, pp. 394-396.

Vollset SE (1993) Confidence intervals for a binomial proportion. Statistics in Medicine 12: 809-824.

Wallenstein S, Weinberg CR, Gould M (1989) Testing for a pulse in seasonal event data. Biometrics 45: 817-830.

Walter SD (1980) Exact significance levels for Hewitt's test for seasonality. Journal of Epidemiology and Community Health 34: 147-149.

Walter SD, Elwood JM (1975) A test for seasonality of events with a variable population at risk. British Journal of Preventive and Social Medicine 29: 18-21.

Wilson EB (1927) Probable inference, the law of succession, and statistical inference. Journal of the American Statistical Association 22: 209-212.

Waterhouse J, Muir C, Corea P, Powell J, eds. (1976) Cancer incidence in five continents, vol. III (IARC Scientific Publications no. 15), Lyon: International Agency for Research on Cancer.

Williams K (1976) The failure of Pearson's goodness of fit statistic. The Statistician 25: 49.

University of Manchester School of Economic Studies. Statistical tables. Internet document www.ses.man.ac.uk/clark/es561/tables.pdf

US Army Corps of Engineers 2001. Performance Evaluation (PE) Program: Engineer manual. Internet document: http://www.usace.army.mil/publications/eng-manuals/em200-1-7/basdoc.pdf

Young LC (1941) On randomness in ordered sequences. Annals of Mathematical Statistics 12:293-300.

Zar JH (1998) Biostatistical analysis, 4th edn: Prentiss Hall.

Zwillinger D. Kokoska S (1999) CRC standard probability and statistics tables and formulae. Boxa Raton: Chapman & Hall/CRC.
