# Supplementary material for: WINPEPI (PEPI-for-Windows): computer programs for epidemiologists
Source: Epidemiol Perspect Innov. 2004 Dec 17;1:6. doi: 10.1186/1742-5573-1-6 (PMC544871; doi:10.1186/1742-5573-1-6)
Supplement: Additional File 1 — WINPEPI package. WINPEPI programs, with manuals and Pepi Finder. [file 1742-5573-1-6-S1.zip › Pairsetc.pdf]

PAIRSetc

MANUAL

(Version 1.15)

© J.H. Abramson

Revised Nov 8, 2004

PAIRSetc is a WINPEPI (PEPI-for-Windows) program, part of the PEPI suite of computer programs for epidemiologists. ("PEPI" is an acronym for "Programs for EPIdemiologists".) The program can be run in any version of Windows.

PAIRSetc provides procedures for use in comparisons of paired and other matched observations. The "etc" in its name indicates its ability to deal with sets of matched observations larger than pairs.

The program appraises differences, and also provides *kappa* and other measures of agreement. It may be used for analyses and meta-analyses of cross-sectional, cohort or case-control studies and of trials, and in reliability studies that compare replicate observations or methods of measurement. It can analyse stratified data, compute power and sample sizes, and appraise the effect of misclassification. There are 29 modules to choose from:

How to use PAIRSetc 3

Analysis of paired observations

A. "Yes-no" (dichotomous) variable 5

B. Three or more categories, not ordered 14

C. Three or more ordered categories 19

D. Numerical observations: compare 2 matched samples or replicates

- **D1. Normal distribution assumed** 26
- **D2. Lognormal distribution assumed.** 32
- **D3. Normality not assumed** 37
- **D4. Analysis of paired survival data** 43

Analysis of sets of 3 or more matched observations

E. "Yes-no" variable: compare cases with 2 or more controls 47

F. "Yes-no" variable: compare 3-10 matched samples 50

G. Compute *kappa* for 3 or more ratings 53

H. Numerical observations: compare two groups or two measures 54

I. Numerical observations: compare 3-10 matched samples or replicates 58

Analysis of sets of varying numbers of matched observations

J. "Yes-no" variable (compare cases and controls) 63

K. "Yes-no" variable: compute *kappa* only 65

L. Numerical observations: compare two sets of varying numbers of observations

- **L1. Compare two groups** 66
- **L2. Compare two methods of measurement** 68

M. Numerical observations: compare replicate measurements 70

Effect of misclassification on paired dichotomous data:

Mis1. Comparison of cases and controls 72

Mis2. Comparison of exposed and nonexposed 73

Mis3. Comparison of any two matched groups 74

Power

P1. Difference between proportions (matched pairs) 75

P2. Difference in distribution of an ordinal-scale variable (matched pairs) 76

P3. Difference between means (matched pairs) 77

Sample sizes

S1. "Yes-no" data: Difference (McNemar test) 78

S2. "Yes-no" data: Agreement (*kappa*) 80

S3. "Yes-no" data: Equivalence test 81

S4. Ordered categories: Difference between pairedbservations 82

S5. Numerical data: Difference (paired *t* test) 83

S6. Numerical data: Agreement (intraclass correlation coefficient) 84

S7. Numerical data: Equivalence of paired observations 86

References.. 88

WORDS OF CAUTION

This program offers more options than most users will ever need, and will usually display more results than are needed. Ignore the options and results you don't require.

It is unwise to use a statistical procedure whose use one does not understand. This manual cannot supply this knowledge, and it is certainly no substitute for the basic understanding of statistics and epidemiological thinking that is essential for the wise choice of methods and the correct interpretation of their results.

HOW TO USE PAIRSetc

First **choose a module.** Then follow the simple on-screen instructions**.** **T**o return to the main menu, click on the *"Return to main menu"* button, or on "*Main menu"* in the top menu.

Choosing a module:

Modules **A** to **L** are listed in the main menu. The other options are accessed by clicking on "*Misclass*", *Power*", or :"*Sample size*" in the top menu.

The choice of a module depends on:
(a) whether the matched sets are *pairs* (options **A** to **D**), have a *fixed size of 3 or more* (options **E** to **I**), or *vary in size* (options **J** to **M**)
(b) whether the dependent variable is *categorical* (*dichotomous [*options **A**, **E**, **F**, **J**, **K]**, *nominal* [option **B]**, or *ordered* [option **C**]), or *numerical* (options **D**, **H**, **I**, **L1, L2,M**); and (in option **D)** whether the distribution is *normal or lognormal*, or *normality is not assumed.*
(c) the procedures required:

- to *compare two groups*: **A**, **B**, **C**, **E** or **J** (*categorical variable)* or **D**, **H** or **L** (*numerical variable*);
- to *compare paired survival data:* **D4***;*
- to *compare 3–10* matched observations: **F** or **I**;
- to compute *kappa*: **A**, **B**, **C**, **E** or **J,** or (for *kappa* only) **G** or **K**;
- *to compare replicate numerical measures:* **D** (2 replicates), **I** (3–10 replicates) or **M** (variable numbers);
- to *compare two methods of measuring a numerical variable*:
  - using one measurement by each method: **D**;
  - using replicate measurements: **H** (fixed number of replicates) or **L2** (variable number of replicates);
- to appraise the effect of *misclassification*: click on "*Misclass*" in the top menu;
- to estimate *power* or *sample size*: click on "*Power*" or :S*ample size*" in the top menu;

Entry of data

The required entries are the findings in each member of the matched pair or set, and/or the number of pairs or sets with a given pattern of findings.

If the data are stratified, enter each stratum in turn. For meta-analyses, enter each study as a separate stratum. Click on "All strata" whenever combined results are required.

Easy entry of data:

- If entries are required in different boxes, pressing <Enter> or <Tab> will generally take you to the next box; pressing <Escape> will clear the entry.
- If several entries are required in the same box, press <Enter> or <Space> after each entry.
- Optionally, data can be "pasted" into entry boxes (see below).

Recalling results:

Click on *"View***"** in the top menu to display the current session's previous results

Pasting results:

Results shown on the screen are automatically placed in the Windows clipboard**,** from which they can be pasted to other applications, at the site of the cursor (usually by pressing *Shift-Insert* or *Ctrl-V*). Click on "*Not***e**" in the top menu if you wish to add explanatory comments to be placed in the clipboard (or printed)) with the results

If the current session's previous results are recalled (by *clicking on* "View"*)*, text can be marked (drag the mouse over it with button pressed) and copied to the clipboard (by pressing *Ctrl-Insert* or *Ctrl-C)* for pasting elsewhere.

Saving results:

By default, all results of Pepi-for-Windows programs are saved in C:\PEPI.TXT, with a warning if it exceeds 500K. Results also go to C:\PEPI.TMP (for display in the 'View' option); this file may be overwritten unless it is renamed on quitting PAIRSetc. Click on "Save" (in the top menu) to see the default procedure or to alter it. TXT files can be combined by using JOINTEXT, available free from www.brixtonhealth.com

Printing results:

Click on *"Print"*. If this fails*,* try switching the printer off and on again. Or paste the results from the clipboard to Word or another program, and print from there. Results can also be printed from the file in which they are saved. Note: the "Print" option ejects full pages only.

If you get an "*Error opening window*" message, close and re-open PAIRSetc.

PASTING DATA

If the data are available in a text file (e.g. a TXT file created by Notepad), they can be copied to the Windows clipboard [usually by pressing *Ctrl-Insert* or *Ctrl-C*], and then "pasted" into a data-entry box [usually by pressing *Shift-Insert* or *Ctrl-V*]. This can simplify data entry in boxes that require a number of entries (in rows or columns). [Also, data can be copied from a data-entry box and pasted to a text file for future re-use; press *Ctrl-A* to mark it for copying.]

Precautions:

- The data must be pasted into the box as a single block, and not piecemeal.
- The data must be in the format required in the box, with spaces between the numbers; exact alignment
. of the columns is not necessary. For example 45 66 1
20 3 132
53 11 44
- If a defined number of rows is required, this number must be entered first, e.g. in the "Number of strata" or "Number of categories" box.
- If row numbers are shown on the left (1, 2, etc.), ensure that the"1" is visible before pasting.
- The cursor must be in the top left corner of the box when the "paste" keys are pressed.

HOW TO OBTAIN PEPI PROGRAMS

All WINPEPI (PEPI-for-Windows) and other PEPI programs can be downloaded free. The latest versions of WINPEPI programs – currently COMPARE2, DESCRIBE, PAIRSetc, and WHATIS – can be downloaded from www.brixtonhealth.com; and the latest release of Version 4 of PEPI, which contains over 40 DOS-based programs (which can be used in Windows) and WHATIS, can be downloaded from www.sagebrushpress.com/pepibook.html or www.simtel.net/pub/pd/54632.html

COMPARE2, DESCRIBE, and PAIRSetc are distributed with manuals (as computer files). A printed manual is available for the DOS-based programs and WHATIS (Abramson and Gahlinger 2001.).

WINPEPI programs are provided with no liability to users and without any warranties, whether expressed or implied. They are copyrighted, but may be freely copied and distributed for personal use; they may not be exploited commercially without permission.

A. PAIRED OBSERVATIONS: “YES-NO” (DICHOTOMOUS) VARIABLE

This module is appropriate for the analysis of paired observations (in different subjects or the same subject), where the dependent variable is a dichotomy (”yes-no”"). It appraises differences and agreement between the two sets of observations . It can be used to analyse matched-control trials and matched case-control studies, before-after studies, and other comparisons of paired subjects or observations, such as comparisons of husbands and wives, and diagnoses of the same individuals by two different observers or diagnostic techniques. It can handle clustered and stratified data and (see below) data collected by *inverse sampling*.

The numbers of pairs with each combination of findings are entered in a 2 x 2 table in which A and B are the paired sets of observations, and “yes" refers to the presence of the disease or other characteristic under study; in a case-control-study, “yes” usually refers to exposure to a risk factor or protective factor. Numbers of pairs are entered, not numbers of observations.

The controls in a case-control study or trial, and the unexposed in a cohort study should be designated “B”. To test for *equivalence*, the bounds of “equivalence” must be defined, by specifying the largest difference between the proportions of “yes” in A and B that is to be regarded as negligible (e.g. 0.05).

If the data are stratified, enter each stratum in turn; for *meta-analyses*, enter each study as a separate stratum. If there are *clusters* of paired observations that may not be independent (e.g. various pairs of observations of the same person, or by the same observer), enter each cluster as a separate stratum. Click on “All strata” whenever combined results are required.

For each table, the program provides **tests for the difference** between the paired observations, a **test of equivalence** (optional), the **odds ratio** (with a low-bias estimator), the **proportions** (of “yes”) **and their difference and ratio,** the **relative difference**, and the **number needed to avoid one event** (for studies that contrast exposure and nonexposure to a factor or treatment), **attributable or prevented fractions (**for paired case-control studies), ***kappa* and related results**, and a measure of the **distinguishability of categories**.

For *stratified data*, the program provides overall **tests for the difference**, **heterogeneity tests and measures**, the overall **odds ratio**, and ***kappa* and related results**. Three sets of tests and measures are provided for **clustered data**. For studies using **inverse sampling,** a test and confidence intervals for the odds ratio are provided.

Tests for the difference between paired observations

For each table, and (if stratified data are entered) for the combined (pooled) data, the program provides Fisher’s and mid-P exact tests (unless the user aborts their computation or numbers are very large) and McNemar tests (with and without a continuity correction) for differences between A and B. Lui (2001b) recommends use of the McNemar test, uncorrected for continuity, rather than Fisher's exact test, which (like the corrected McNemar test) "can be quite conservative and hence lose much efficiency". The uncorrected McNemar

test is more powerful, and performs well even when the number of discordant pairs is as low as 6.

Heterogeneity tests and measures

For stratified data (i.e., a series of tables), the program provides *heterogeneity tests* that compare the odds ratios in the different strata, and the *kappa* values in the different strata. These permit appraisal of the modifying effect of the stratifying variable. The greater the similarity, the higher the P-value. The tests should be interpreted with caution, since their power is low; if the result is significant at the 0.05 level, the hypothesis of homogeneity can be rejected; but "a high p-value ... does not show that the measure is uniform, it only means that heterogeneity ... was not detected by the test" (Rothman and Greenland 1998: 276); the larger the strata, the more valid the test.

The program also provides two *measures of heterogeneity*, *H* and *I-squared* (Higgins and Thompson 2002), with their approximate 95% intervals. An *H* value of less than 1.2 suggests absence of noteworthy heterogeneity, whereas a value exceeding 1.5 suggests its presence, even if the heterogeneity test is not significant. *I-squared* expresses the proportion of variation that can be attributed to heterogeneity (in a meta-analysis, to interstudy variation) rather than to sampling error.

Estimates of the supposed common underlying value of the odds ratio or *kappa* are of questionable value if the findings in the various strata are very disparate. If the results are not uniform, explorations of possible causes - e.g. associations with study design or quality or with the sizes or other characteristics of the samples - may be revealing

Test of equivalence

The program offers an equivalence test for the proportions of "yes" in two matched samples. This test may be appropriate if no statistically significant difference has been found, e.g. in "negative trials" that compare a new treatment with an established standard treatment, where there may be a reason to prefer the new treatment if it is at least as effective as the standard treatment.

To use the test, the bounds of "equivalence" must be defined by specifying the largest difference between proportions (e.g., 0.05) that is to be regarded as negligible.

Two null hypotheses are tested: these are the hypotheses that there is more than a specified "negligible" difference in each direction – i.e., that the first proportion is more than negligibly higher than the first, and that the second is more than negligibly higher than the first. If both tests yield significant results, this supports the alternatives to the null hypotheses, namely that both these one-sided differences are negligible - that is, the proportions are equivalent. If only one test is significant, this indicates that one proportion is at least as high as the other. The larger of the two P values is displayed as the P value for the equivalence test (Liu *et al*. 2002).

Odds ratio

The odds ratio is computed with its exact Fisher's and mid-P confidence intervals, unless numbers are very large or the computation is interrupted by the user, in which case Poisson-

based confidence intervals are substituted. Jewell's low-bias estimator of the odds ratio (Jewell 1984) is shown. (Alternative confidence intervals are computed for studies using inverse sampling: see below.)

If stratified data are entered, a pooled odds ratio is computed for the combined data, with exact Fisher's and mid-P confidence intervals or Poisson-based confidence intervals.

Proportions, difference between proportions, ratio of proportions

The proportions of "yes" observations in the two samples, their absolute difference, and their ratio are displayed, all with their 90%, 95%, and 99% confidence intervals.

Relative difference

The program computes the relative difference between the proportions, with its confidence intervals. This measure (Fleiss 1981: 118-119) is defined as the difference between the numbers of "yes" responses in the samples, divided by the number of controls with "no" responses in sample B. It may be useful in the analysis of clinical trials in which a group receiving a new treatment (entered as sample A) is compared with a control group receiving a standard treatment (sample B). If "yes"' indicates a favourable response to treatment, the relative difference is a measure of the relative value of the new treatment, based on the assumption that the new treatment can benefit only those patients who fail to improve under the standard treatment. It is the proportion of subjects who are expected to respond to the new treatment, among those who fail to respond to the standard treatment (Lui 2004: 56).

Number needed to avoid one event

The program reports the number of individuals who are needed in the group with a lower rate in order to avoid a single case, with its approximate 95% confidence interval. These results apply to studies that compare the proportions of cases (of disease, etc.) in paired subjects exposed and not exposed to a risk or protective factor or treatment, and to two-period crossover trials.

In a clinical trial the number needed has been called the "number needed to treat"' or "number needed to treat (benefit)" (Altman 1998), i.e. the number of patients who must be treated in order to prevent one event (Sinclair and Bracken 1994, Feinstein 1995). In an observational study of a supposed cause of disease, it indicates the number of people whose exposure must be prevented in order to prevent one event (assuming that the findings reflect a cause-effect relationship and that the causal factor and its effect are modifiable).

The number is the reciprocal of the risk difference, and the 95% confidence limits for the number needed in a group to avoid one case are the reciprocals of the 95% confidence limits for the risk difference. Since the confidence interval for the rate difference may straddle zero, the confidence interval for the number needed to avoid one case may straddle infinity. A confidence interval of 5.5 to -2.2 is reported as "5.5 to infinity (in the one group), then up to 2.2 in the other group".

Attributable or prevented fractions

Attributable and prevented fractions in the exposed and in the population are computed, with their confidence intervals. These are appropriate for case-control studies where the cases are randomly selected and the disease is rare. Confidence intervals based on large-sample standard errors are provided; they should be used with caution if numbers are small.

The computation of the fractions and their standard errors is based on the methods described by Kuritz and Landis (1987, formulae 4 to 9). If the attributable fraction AF is negative the cases and controls are reversed for the purposes of computation, and the calculated attributable fraction is reported as the prevented fraction PF. If a lower confidence limit for an AF is negative, the equivalent PF is displayed in parentheses.

The confidence intervals of the attributable and prevented fractions in the exposed are computed by Kuritz and Landis's formulae 10 and 11. The confidence intervals of these fractions in the population are generally based on the quadratic-equation method proposed by Lui (2001a, method 5). If the odds ratio is 4 or more or 0.25 or less, however, or the proportion of cases who are exposed is 50% or more, use is instead made of logit-transformed estimators, as recommended by Lui (2001a, method 3).

Kappa and related results

Kappa is generally used to measure the agreement between two “yes”-“no” ratings (by different observers or tests, or by the same observer on different occasions) of the same individuals. In addition to this use as a measure of reliability, it may be used to measure concordance in other situations where paired samples are compared (Fleiss 1981: 232-233). In a matched case-control study or matched-control trial, *kappa* may serve as an indication of the effectiveness of a matching procedure – it indicates the extent to which the findings in matched pairs are more similar than findings in individuals from different pairs (Fleiss 1981: 233). Uses and misuses of *kappa* in epidemiology are discussed by (among others) MacLure and Willett (1987), Thompson and Walter (1988a, 1988b) and Kraemer and Bloch (1988).

The probability of chance agreement is taken into account in the calculation of *kappa*. A value of 1 indicates perfect agreement (after allowing for this probability of chance agreement) between ratings; 0 indicates no agreement other than what can be attributed to chance, and a negative value indicates less than chance agreement. Fleiss (1981: 218) suggests that a value of 0.75 or more indicates excellent agreement, and 0.40 or less indicates poor agreement. Alternative guidelines are: over 0.80, very good agreement; 0.61-0.80, good; 0.41-0.60, moderate; 0.21-0.40, fair; and 0.20 or less, poor agreement (Altman 1991). These levels may be taken into account in the appraisal of confidence intervals, e.g. by seeing whether the lower confidence limit lies above 0.40 (Basu and Basu 1995).

A one-tailed test is done, indicating whether *kappa* is significantly higher than zero. If *kappa* is 0.4 or more, a second test is done, indicating whether it is significantly higher than 0.4; and if it is 0.6 or more, a third test is done, indicating whether it is significantly higher than 0.6.

Confidence intervals are estimated both from the standard error and by a goodness-of-fit approach (Donner and Eliasziw 1992). The latter intervals are more accurate than those based on the standard error, especially in small samples; if any of the expected frequencies is <1, the intervals are labelled as approximate.

Paradoxical values of *kappa* may occur because of bias (systematic one-sided variation between two ratings) – indicated by the McNemar test (see above) - or a skewed distribution (inequality between the prevalences of the categories in the two samples), indices of bias and skewed prevalence are displayed, and two adjusted values of kappa – BAK (*bias-adjusted kappa*) and PABAK (*prevalence-adjusted bias-adjusted kappa*) – are computed (Byrt *et al.* 1993*)*. These adjusted values are conditional on the observed percentage agreement. BAK is the value that *kappa* would take if there were no systematic one-sided variation between the ratings; it is equivalent to Scott's *pi* coefficient of agreement (Scott 1955). Low *kappa* values are likely to be affected by such bias. PABAK is the value that *kappa* would take if, in addition, the prevalence of each category (as expressed by the mean of the two raters' totals for the category) was equal. PABAK may be useful in appraising agreement when the percentage agreement is high and *kappa* is paradoxically low; it approximates to the highest possible *kappa* if the percentage agreement is above about 50% (Lantz and Nebenzahl 1996). PABAK is called *kappa-no*r by Lantz and Nebenzahl (1996), and is equivalent to Maxwell's *RE* (random error) coefficient of agreement (Maxwell 1977) and Bennett's S coefficient (Bennett et al. 1954). It should be noted that simulation studies have suggested that PABAK may substantially overestimate agreement (Hoehler 2000).

The program also displays the *maximum attainable kappa* consistent with the marginal totals, i.e. consistent with the observed level of bias.

The *percentage agreement* is also shown. This is the percentage of individuals who are placed in the same category by both ratings, and (unlike *kappa*) it is not corrected for chance agreement. In clinical practice, the percentage of agreement for a positive rating (the proportion of positive agreement) represents the probability that, if a subject has been given a positive rating by a typical observer, another typical observer will concur. Similarly, the proportion of negative agreement expresses the probability of concurrence with a negative rating (Samsa 1996). This requires the assumption that the two observers have a similar tendency to rate subjects as “yes” or “no”; the program estimates confidence intervals for these two proportions of agreement only if there is no significant difference between the proportions of “yes” observations in the two samples (i.e., if P =>.05 by the NcNemar test for bias). The program displays separate probabilities that a second rating will agree with a first “yes” or “no” rating, depending on whether rating A or B is made first.

If *stratified data* are entered (e.g. observations of individuals in different age groups), the heterogeneity of the *kappa* values in the different strata is tested, measures of heterogeneity (see above) are provided, three estimates of the overall *kappa* are computed, with their confidence intervals, and overall values of the percentage agreement and of the percentage agreement for each category. are reported. . The first estimate of the overall *kappa* is precision-based; it is produced by weighting each *kappa* by the inverse of its variance (Fleiss 1981: 222). The second uses the methods of Donner and Klar (1996); computation of the overall kappa is based on the common correlation model (in which the expected responses for each pair of observations are based on the overall prevalence of the two possible responses). The associated heterogeneity test (which appraises compatibility of the stratum-specific estimates with the overall *kappa*) and estimation of confidence intervals are based on a goodness-of-fit approach, which has been shown to provide satisfactory confidence intervals for combined samples with as few as 50 subjects (Donner and Eliasziw 1992). The third estimate is obtained by weighting the *kappa* values by the sizes of the samples in the

strata. A simulation study suggests that this is preferable to the precision-based method if *kappa* is not zero (Barlow *et al.* 1991).

Distinguishability of categories

A measure of the *distinguishability of the categories* (Darroch and McCloud 1986) is computed. This may be useful in a methodological study in which the matched observations represent separate ratings. The measure ranges from 100% if there are no disagreements, to zero if disagreements outnumber agreements.

Inverse sampling

Inverse sampling refers to the addition of pairs to the sample until a prespecified number of pairs with a specific combination of attributes has been found. The computation is based on the assumption that it is the number of pairs with an A: "no", B: "yes" combination that was specified in advance (the two sets of observations should be labelled accordingly when entering the findings). This method of sampling is appropriate only if subjects are accrued sequentially and their attributes can be determined rapidly

The program provides an appropriate test for the difference between the observations, and exact 90%, 95%, and 99% *confidence intervals for the odds ratio*.

Clustered data

Some studies are based on clusters of paired "yes"-"no" observations that may not be independent, e.g. pairs of observations of the same person, or by the same observer. The study might, for example, be a clinical trial of the effects of treatment applied to the eyes of patients with early signs of cataract, based on before-after appraisals of visual acuity ("impaired" or "normal"). Since a person's two eyes may be similar, the findings may not be independent, and a simple McNemar test based on the pooled data might yield a spuriously high level of significance. Clustering may similarly occur in a study in which paired observations are made on multiple teeth belonging to the same person, or on multiple blood or tissue samples, or in a study in which different observers participate. In such studies the data comprise clusters of related observations, one cluster per subject or per observer. Clusters may contain different numbers (one or more) of pairs of observations.

To analyse clustered data, all that is required is to enter each cluster as a separate stratum. When the combined strata are analysed, the effect of clustering is appraised and allowed for.

Three procedures are provided: those described by Eliasziw and Donner (1991), by Obuchowski (1998), and by Durkalski *et al.* (2003). The Eliasziw-Donner procedure adjusts the McNemar test and estimates adjusted confidence intervals for the odds ratio, and the latter two provide significance tests and adjusted 95% confidence intervals for the difference between the proportions of "yes" responses in the sets of paired observations. The relative value of the three tests varies in different circumstances. The Obuchowski test is slightly less powerful than the Eliasziw-Donner test (Obuchowski 1998), and is more powerful than the Durkalski test if cluster size is very variable (Durkalski *et al.* 2003).

METHODS

Tests for the difference between paired observations

The Fisher’s and mid-P exact tests use an efficient algorithm for calculating the coefficients of the conditional distribution (Martin and Austin 1991, 1996), using code from David O. Martin's public-domain EXACTBB program. The McNemar tests use formulae 4.3 and 4.4 of Siegel and Castellan (1988: 43).

Heterogeneity tests and measures

The test for the heterogeneity of odds ratios in different strata is based on a multiple-sample goodness-of fit test (Sokal and Rohlf 1981: 711-716; Zar 1996: 471-473), using log-likelihood chi-squares (without corrections for continuity) in each stratum; 0.0000001 is added to cells with frequencies of zero. The tests are for goodness of fit with an equal distribution of pairs with discrepancies in different directions.

The.test for the heterogeneity of *kappa* values is based on the method of Donner and Klar (1996).

The *measures of heterogeneity* (Higgins and Thompson 2002) are *H* and *I-squared*. *H* is computed by Higgins and Thompson's formula 6, and increased to 1 (indicating absence of heterogeneity) if it less than 1. A test-based interval is computed by Method III. *I-squared* and its 95% interval are computed from *H*, using formula 10.

Test of equivalence

The program uses a test based on restricted maximum likelihood estimation (RMLE), without a continuity correction. This method, described by Nam (1997), has been evaluated and recommended by Liu *et al*. (2002), who explain how to replace the standard errors in the basic formulae (formulae 4 and 5) with RMLE-based values.

Odds ratio

The odds ratio is b/c or c/b, where *b* and *c* are the numbers of discrepant pairs. The low-bias estimator of the odds ratio (Jewell 1984) is $b \,/\, (c + 1)$ or $c \,/\, (b + 1)$.

Confidence intervals for odds ratios are estimated by treating the two values as Poisson variates, with their ratio (the odds ratio) distributed binomially (Morris and Gardner 2000: 65) Exact probabilities and confidence intervals are computed with an efficient algorithm for calculating the coefficients of the conditional distribution (Martin and Austin 1991, 1996), using code from David O. Martin's public-domain EXACTBB program.

Proportions, difference between proportions, ratio of proportions

Confidence intervals for the *proportions* (of “yes”) are computed by the method described by Newcombe and Altman (2000: 46-47), confidence intervals for the *difference between proportions* by the method recommended by Newcombe and Altman (2000: 52) [which is method (10) of Newcombe (1998b)], and confidence intervals for the *ratio of proportions* by means of formulae 16-2 and 16-3 of Rothman and Greenland (1998).

Relative difference

The relative difference is calculated by formula 8.16 of Fleiss (1981: 118), and its confidence intervals by the log-transformation method described by Lui (2004: 57: formula 3.22).

Number needed to avoid one event

The number is the reciprocal of the risk difference, and the 95% confidence limits for the number needed in a group to avoid one case are the reciprocals of the 95% confidence limits. The program uses the method described by Walter (2001) for a crossover design with discrete data (formulae 2 and 3).

Attributable and prevented fractions

The computation of the fractions and their standard errors is based on the methods of Kuritz and Landis (1987, formulae 4 to 9). If the attributable fraction AF is negative the cases and controls are reversed for the purposes of computation, and the calculated attributable fraction is reported as the prevented fraction PF. If a lower confidence limit for an AF is negative, the equivalent PF is displayed (and vice versa), using the formulae

$$PF = 1 - 1 / (1 - AF)$$
$$AF = 1 + 1 / (PF - 1)$$

The confidence intervals of the attributable and prevented fractions in the exposed are computed by Kuritz and Landis's formulae 10 and 11. The confidence intervals of these fractions in the population are generally based on the quadratic-equation method proposed by Lui (2001a, method 5). If the odds ratio is 4 or more or 0.25 or less, however, or the proportion of cases who are exposed is 50% or more, use is instead made of logit-transformed estimators, as recommended by Lui (2001a, method 3).

Kappa and related results

The basic formulae are provided by Fleiss (1981: chapter 13). *Kappa* is calculated by formula 13.12. For tests of the null hypothesis that *kappa* is zero (formulae 13.14 and 13.35), the standard error (for an underlying zero value of *kappa*) is calculated by formula 13.13. For tests of the hypothesis that *kappa* has an underlying value other than zero, and for confidence intervals, the standard error appropriate for non-zero values is calculated by formulae 13.15 to 13.18

Confidence intervals are estimated by two methods: by using the standard error (if the upper confidence limit exceeds 1, it is reduced to 1), and by the goodness-of-fit approach explained by Donner and Eliasziw (1992), which uses a model in which the expected frequencies of "yes"-"yes", "yes"-"no", and "no"-"no" observations are computed from the overall prevalence of "yes" responses.

Bias is appraised by the McNemar chi-square test (see above) . The *bias index* and *skewed-prevalence index* are the indices of symmetry in disagreement and agreement proposed by Lantz and Nebenzahl (1996), calculated as percentages. BAK (*bias-adjusted kappa*) and PABAK (*prevalence-adjusted bias-adjusted kappa*) are computed by the methods described by Byrt *et al*. (1993).

In the combined analysis of several samples or strata, the estimate of the supposed *common or overall value* of *kappa* is calculated in three ways: by computing a weighted mean, using the inverse of the variance of each *kappa* as its weight (Fleiss 1981: formula 13.21); by the methods of Donner and Klar (1996), which use the common correlation model (the expected responses for each pair of observations are based on the overall prevalence of the two possible responses); and by computing a weighted mean, using the size of the stratum as the weight. The confidence intervals of the common kappa are estimated by Fleiss's formula 13.23 and by the goodness-of-fit approach of Donner and Klar (1996).

The *heterogeneity* tests are based on Fleiss's formula 13.22 and the goodness-of-fit approach of Donner and Klar (1996). The measures of heterogeneity (Higgins and Thompson 2002) are described above.

The overall values of the *percentage agreement* are based on the pooled data; this is equivalent to weighting the stratum-specific values by sample sizes.

Distinguishability of categories

This measure is computed by the method described by Darroch and McLeod (1986).

Inverse sampling

The difference between the observations is tested by the formula (Lui 1996) :

$$\text{chi-square (1 d.f.)} = (b - c)^2 / 2c$$

where b = A: “yes”, B: “no”
c = A: “no”, B: ”yes”

Exact confidence intervals for the odds ratio are computed by formula 5.58 of Lui (2004: 112).

Clustered data

The Eliasziw-Donner procedure to adjust for the presence of clusters of non-independent paired observations estimates a weighted average within-cluster intraclass correlation coefficient, *rho*, using information on both concordant and discordant pairs, by the methods described in Section 4 of the paper by Eliasziw and Donner (1991). The program reports the value of r*ho*. Using the methods described in Section 2 of the paper, a correction factor for the McNemar test is then computed (the program divides the McNemar chi-square by this factor). An adjusted variance is computed for the prevalence of discrepancies in one direction, b / (b+c). Confidence intervals are estimated for this prevalence, and converted to adjusted confidence intervals for the pooled odds ratio.

The Obuchowski procedure for comparing correlated proportions in clustered data uses formula 6 of Obuchowski (1998) to compute a chi-square test statistic; for this purpose the estimator of the variance of the difference between the proportions of "yes" responses in the sets of paired observations is computed by formula 4, after substituting the pooled (mean) proportion for the specific proportions in formula 2, and replacing the covariance estimator computed by formula 3 with that provided by formula 7. A 95% confidence interval for the difference between proportions is based on the variance estimator in formula 2; the square root of this variance is displayed as the standard error of the difference.

The procedure described by Durkalski *et al.* (2003) for the analysis of clustered matched-pair data computes chi-square by formula 15. A 95% confidence interval for the difference between proportions (formula 18) is based on the variance estimator in formula 17; the square root of this variance is displayed as the standard error of the difference.

B. PAIRED OBSERVATIONS: THREE OR MORE CATEGORIES, NOT ORDERED

This module is appropriate for the analysis of paired observations (in different subjects or the same subject) where the dependent variable is a nominal-scale one (i.e., with categories that are not ordered). It appraises differences and agreement between the two sets of observations. It can be used to analyse matched-control trials and case-control studies, before-after studies, and other comparisons of paired subjects or observations, such as comparisons of husbands and wives, and diagnoses of the same individuals by two different observers or diagnostic techniques.

The number of categories must be entered, and then the numbers of pairs with each combination of findings are entered in a k x k table in which the paired sets of observations are arbitrarily designated A and B. The numbering and sequence of the categories is arbitrary, except that if there is a reference category it should be given the highest number. Numbers of pairs are entered, not numbers of observations.

If the data are stratified, enter each stratum in turn; for *meta-analyses*, enter each study as a separate stratum. Click on “All strata” whenever combined results are required.

For each table, the program provides **tests for the difference** between the two sets of observation **(**extended McNemar test, Stuart-Maxwell test**)** , showing the sources of disagreement (if there are up to seven categories), and computes **odds ratios and related tests**, ***kappa* and related results**, and a measure of the **distinguishability of categories.**

For *stratified data*, the program provides overall **tests for the difference** (based on the pooled data) and ***kappa* and related results.**

Tests for the difference between paired observations

For each table, the program provides extended McNemar tests for off-diagonal symmetry and (if there are up to 20 categories) the Stuart-Maxwell test for marginal heterogeneity. If stratified data are entered, extended McNemar tests are done on the combined (pooled) data.

The *extended McNemar (“symmetry”) test* (Bowker's test for off-diagonal symmetry) tests the symmetry of the findings; e.g. for categories 1 and 2 (and similarly for each other pair of categories) it tests whether the probability that the observation will be in category 1 in one set of observations and in category 2 in the second is the same as the probability of the reverse combination, namely category 2 in the first set and category 1 in the second. Ordinary (Pearson's) and log-likelihood chi-squares are computed

As a guide to the sources of disagreement (Maxwell 1970), the contribution that each pair of categories makes to a significant McNemar chi-square ($P < 0.05$) is reported (if there are up to seven categories).

The *Stuart-Maxwell test* for marginal heterogeneity tests the hypothesis that the probabilities of the various categories are the same in the two sets of observations.

Odds ratios and related tests

The program provides odds ratios based on the contrast between each pair of categories (if there are up to 10 categories). If the odds ratio based on the contrast between two categories, e.g. 1 and 2 (displayed as "1:2") is above 1, this means that the odds in favour of 1 rather than 2 are higher in sample A than in sample B.

The consistency of these odds ratios based on pairs of categories is tested. For example, if the odds ratio for category 1 versus category 2 is 3.0 and the odds ratio for category 2 versus category 3 is 4.0, the odds ratio for category 1 versus category 3 would be expected to be 12.0. Inconsistency with such expectations suggests that the odds ratios may be modified by the matching variables (Pike, Casagrande, and Smith 1975). A low P value is indicative of inconsistency.

Maximum-likelihood estimates of mutually consistent odds ratios based on the contrast between each pair of categories are computed; these estimates are not very meaningful if the test points to mutual inconsistency.

The program also computes odds ratios based on a comparison of each category with all other categories combined, and does McNemar tests to appraise their significance; alternative P-values are provided for tests of hypotheses formulated before and after seeing the results.

Confidence intervals are displayed for odds ratios contrasting each category with the reference category (the category with the highest category number), assuming mutual consistency.

*Kapp*a and related results

The program computes an overall *kappa* value (for the complete set of categories), and a separate *kappa* values for each category. In each instance, a one-tailed test is done, indicating whether *kappa* is significantly higher than zero. If *kappa* is 0.4 or more, a second test is done, indicating whether it is significantly higher than 0.4; and if it is 0.6 or more, a third test is done, indicating whether it is significantly higher than 0.6. Confidence intervals for *kappa* are estimated from its standard error.

Paradoxical values of *kappa* may occur because of bias (systematic one-sided variation between two ratings) – indicated by the extended McNemar test (see above) – or a skewed distribution (inequality between the prevalences of the categories in the two samples). Two adjusted values of the overall kappa – BAK (*bias-adjusted kappa*) and PABAK (*prevalence-adjusted bias-adjusted kappa*) – are therefore computed (Byrt *et al*. 1993*)*. These adjusted values are conditional on the observed percentage agreement. BAK is the value that *kappa* would take if there were no systematic one-sided variation between the ratings; it is equivalent to Scott's *pi* coefficient of agreement (Scott 1955). Low *kappa* values are likely to be affected by such bias. PABAK is the value that *kappa* would take if, in addition, the prevalence of each category (as expressed by the mean of the two raters' totals for the category) was equal. PABAK may be useful in appraising agreement when the percentage

agreement is high and *kappa* is paradoxically low; it approximates to the highest possible *kappa* if the percentage agreement is above about 50% (Lantz and Nebenzahl 1996). PABAK is called *kappa-no*r by Lantz and Nebenzahl (1996), and is equivalent to Maxwell's *RE* (random error) coefficient of agreement (Maxwell 1977) and Bennett's S coefficient (Bennett et al. 1954). It should be noted that simulation studies have suggested that PABAK may substantially overestimate agreement (Hoehler 2000).

The program also displays the *maximum attainable overall kappa* consistent with the marginal totals, i.e. consistent with the observed level of bias.

Kappa is generally used to measure the agreement between two ratings (by different observers or tests, or by the same observer on different occasions) of the same individuals. In addition to this use as a measure of reliability, it may be used to measure concordance in other situations where paired samples are compared (Fleiss 1981: 232-233). In a matched case-control study or matched-control trial, *kappa* may serve as an indication of the effectiveness of a matching procedure – it indicates the extent to which the findings in matched pairs are more similar than findings in individuals from different pairs (Fleiss 1981: 233). Uses and misuses of *kappa* in epidemiology are discussed by (among others) MacLure and Willett (1987), Thompson and Walter (1988a, 1988b) and Kraemer and Bloch (1988).

The probability of chance agreement is taken into account in the calculation of *kappa*. A value of 1 indicates perfect agreement (after allowing for this probability of chance agreement) between ratings; 0 indicates no agreement other than what can be attributed to chance, and a negative value indicates less than chance agreement. Fleiss (1981: 218) suggests that a value of 0.75 or more indicates excellent agreement, and 0.40 or less indicates poor agreement. Alternative guidelines are: over 0.80, very good agreement; 0.61-0.80, good; 0.41-0.60, moderate; 0.21-0.40, fair; and 0.20 or less, poor agreement (Altman 1991). These levels may be taken into account in the appraisal of confidence intervals, e.g. by seeing whether the lower confidence limit lies above 0.40 (Basu and Basu 1995).

The *percentage agreement* is also shown. This is the percentage of individuals who are placed in the same category by both ratings, and (unlike *kappa*) it is not corrected for chance agreement. In a study in which the same individuals are rated by two observers, this is the percentage of subjects who are placed in the same category by both raters). The percentage agreement is also shown separately for each category (if there are up to six categories). In clinical practice, the percentage of agreement for a specific rating represents the probability that, if a subject has been given that rating by a typical observer, another typical observer will concur.

If *stratified data* are entered (e.g. observations of individuals in different age groups), the heterogeneity of the overall kappa values in the different strata is tested, measures of heterogeneity (see above) are provided, two estimates of the overall kappa are computed, with their confidence intervals. The first estimate of the overall kappa is precision-based; it is produced by weighting each kappa by the inverse of its variance (Fleiss 1981: 222). The second estimate is obtained by weighting the kappa values by the sizes of the samples in the strata. A simulation study suggests that this is preferable to the precision-based method if kappa is not zero (Barlow et al. 1991). A heterogeneity test is done, and supplemented by two measures of heterogeneity, H and I-squared (Higgins and Thompson 2002), with their approximate 95% intervals. An H value of less than 1.2 suggests absence of noteworthy heterogeneity, whereas a value exceeding 1.5 suggests its presence, even if the heterogeneity

test is not significant. I-squared expresses the proportion of variation that can be attributed to heterogeneity (in a meta-analysis, to interstudy variation) rather than to sampling error. Overall values of the percentage agreement are reported. These are based on the pooled data; this is equivalent to weighting the stratum-specific values by sample sizes.

Distinguishability of categories

A measure of the distinguishability of pairs of categories is computed. This may be useful in a methodological study in which the matched observations represent separate ratings. The value is 100% if there are no disagreements, and zero if disagreements outnumber agreements.. The average of the values for all pairs of categories is displayed. and the least distinguishable pair of categories is identified.

METHODS

Tests for the difference between paired observations

The *extended McNemar ('symmetry') test* is described by Bowker (1948), Everitt (1977: 114-115) and Zar (1998: formula 9.22). Corresponding cells that both have zero values are omitted from the calculation of this chi-square. The contributions that specific pairs of categories make to a significant chi-square ($P < 0.05$) are computed by formula 6 of Maxwell (1970). In McNemar tests for single categories, the degrees of freedom are defined as $k-1$ (where k = number of categories) for testing *a posteriori* hypotheses (Fleiss 1981: 121).

The *Stuart-Maxwell chi-square test* is computed by Fleiss and Everitt's method (Fleiss 1981: 120-122; Everitt 1977: 115-116).

Odds ratios and related tests

If there is a zero observed frequency of pairs in any cell, adjusted odds ratios are computed, by adding 0.5 in each cell.

The test for the consistency of odds ratios between pairs of categories, the maximum-likelihood estimation of mutually consistent odds ratios, and the estimation of confidence intervals are described by Pike, Casagrande and Smith (1975).

Kappa and related results

The basic formulae are provided by Fleiss (1981: chapter 13). Kappa for single categories and for the total distribution (*overall kappa*) are calculated by formulae 13.10 to 13.12. For tests of the null hypothesis that *kappa* is zero (formulae 13.14 and 13.35), the standard error (for an underlying zero value of *kappa*) is calculated by formula 13.13. For tests of the hypothesis that *kappa* has an underlying value other than zero, and for confidence intervals, the standard error appropriate for non-zero values is calculated by formulae 13.15 to 13.18. Confidence intervals are estimated from the standard error (if the upper confidence limit exceeds 1, it is reduced to 1).

Bias is appraised by the extended McNemar (symmetry) test (see above). BAK (*bias-adjusted kappa*) and PABAK (*prevalence-adjusted bias-adjusted kappa*) are computed by the methods described by Byrt *et al.* (1993).

In the combined analysis of several samples or strata, the estimate of the supposed *common or overall value* of *kappa* is calculated in two ways: by computing a weighted mean, using the inverse of the variance of each *kappa* as its weight (Fleiss 1981: formula 13.21); and by computing a weighted mean, using the size of the stratum as the weight. The confidence intervals of the common kappa are estimated by Fleiss's formula 13.23.

The *heterogeneity test* is based on Fleiss's formula 13.22. The *measures of heterogeneity* (Higgins and Thompson 2002) are *H* and *I-squared*. *H* is computed by Higgins and Thompson's formula 6, and increased to 1 (indicating absence of heterogeneity) if it less than 1. A test-based interval is computed by Method III. *I-squared* and its 95% interval are computed from *H*, using formula 10.

Some computations are omitted if division by zero or other problems are encountered. In some instances, zero values are changed to 0.00001 to permit computation.

Distinguishability of categories

This measure is computed by the method described by Darroch and McLeod (1986).

C. PAIRED OBSERVATIONS: THREE OR MORE ORDERED CATEGORIES

This module is appropriate for the analysis of paired observations (in different subjects or the same subject) where the dependent variable has three or more categories that fall into a sequence. It appraises differences and agreement between the two sets of observations. It can be used to analyse matched-control trials and case-control studies, before-after studies, and other comparisons of paired subjects or observations, such as comparisons of husbands and wives, and diagnoses of the same individuals by two different observers or diagnostic techniques.

The number of categories must be entered, and then the numbers of pairs with each combination of findings are entered in a *k* x *k* table in which the paired sets of observations are arbitrarily designated A and B. The categories must be entered in the correct sequence; if there is a reference category it should be given the highest number. Numbers of pairs are entered, not numbers of observations. S*cores* of 1, 2, 3, etc. are allotted to the categories (for use in computing a weighted *kappa*), but these default scores can optionally be changed to numbers that are believed to better express the relative closeness of the categories.

If the data are stratified, enter each stratum in turn; for *meta-analyses*, enter each study as a separate stratum. Click on "All strata" whenever combined results are required.

For each table, the program provides **tests for the difference** between the two sets of observation , including tests appropriate for ordered categories (Mann-Whitney test, Fleiss-Everitt test, Wilcoxon signed-rank test, permutation test) and tests that ignore the sequence of the categories (extended McNemar test, Stuart-Maxwell test), and computes **odds ratios and related tests**, ***kappa* and related results**, a measure of the **distinguishability of categories,** and **rank correlation coefficients and other measures of ordinal association.**

For *stratified data*, the program provides overall **tests for the difference**, a **heterogeneity test**, a **generalized odds ratio**, and ***kappa* and related results.**

Tests for the difference between paired observations

The Mann-Whitney test, Fleiss-Everitt test for three ordered categories, Wilcoxon signed-rank test, and permutation test take account of the sequence of the categories, whereas the extended McNemar test and Stuart-Maxwell test ignore their sequence.

The *Mann-Whitney test for paired data* (Agresti 1984: 208-209) is a large-sample test that compares the frequencies in two sets of paired observations; if the data are arranged in the format of a square contingency table these are the marginal distributions. A two-tailed P-value is shown, labelled as "approximate" if there are under 50 pairs of observations. If stratified data are entered, the test is also done on the combined data, after weighting the test statistics in the strata in three different ways – equally, by the sample sizes in the strata, and by the square roots of the sample sizes – as well as a simple test on the pooled data.

The *Fleiss-Everitt test*, which is done if there are three categories, tests whether in one set of observations there tend to be more values at one end of the scale and fewer at the other, compared with the other set of observations (Fleiss 1981: 122-123). If stratified data are entered, the test is also done on the combined (pooled) data.

The *Wilcoxon signed-ranks test* (Siegel and Castellan 1988: 87-95) tests whether the median discrepancy between paired observations is zero. It is done only if the differences between each pair of adjacent categories can be assumed to be equal, that is, if the scores allotted to adjacent categories are equally spaced. The test is appropriate if the differences between paired observations are an acceptable basis for ranking the differences in the characteristic that is measured.

The *permutation test for matched pairs* (Siegel and Castellan 1988: 95-100 is appropriate for interval-scale variables, and is therefore done only if the scores allotted to adjacent categories are equally spaced. It is not done if there are more than 20 pairs. The test provides exact P-values; one-tailed P-values are displayed if $P < .05$; the one-tailed value is doubled to provide a two-tailed value.

The *extended McNemar ("symmetry") test* (Bowker's test for off-diagonal symmetry) tests the symmetry of the findings; e.g. for categories 1 and 2 (and similarly for each other pair of categories) it tests whether the probability that the observation will be in category 1 in one set of observations and in category 2 in the second is the same as the probability of the reverse combination, namely category 2 in the first set and category 1 in the second. Ordinary (Pearson's) and log-likelihood chi-squares are computed. The test is equivalent to the test for goodness of fit with a symmetry model described by Agresti (1984: 202). If stratified data are entered, the test is also done on the combined (pooled) data.

As a guide to the sources of disagreement (Maxwell 1970), the contribution that each pair of categories makes to a significant McNemar chi-square ($P < 0.05$) is reported (if there are up to seven categories).

The *Stuart-Maxwell test* for marginal heterogeneity tests the hypothesis that the probabilities of the various categories are the same in the two sets of observations.

Heterogeneity test

If stratified data are entered, goodness of fit with a symmetry model is tested twice, once using the pooled data, and once using the sum of the goodness-of-fit chi-squares in the separate strata. The difference between the two goodness-of-fit chi-squares is an indication of the effect of the stratifier variable(s), and is displayed as a heterogeneity test. The result should be interpreted with caution, since test has a low power. The symmetry model is based on the assumption that the probability of discrepant pairs in which the case is in category 1 and the control in category 2 is the same as the probability of pairs in which the case is in category 2 and the control in category 1 (and similarly for other pairs of categories); i.e. the odds ratio (as generally computed for paired data) is 1 (Agresti 1984: 202).

The heterogeneity of *kappa* values is also tested (see below).

Odds ratios and related tests

The *generalized odds ratio* or GOR (the odds ratio for ordinal data) is displayed. This is the odds in favour of a higher score in one sample than in the other, i.e. the ratio of pairs with a higher score in one sample to pairs with a higher score in the other sample. It is assumed that this odds ratio is the same for each pair of categories (Agresti 1984: 203). The ratios in both directions are displayed, with their 90%, 95% and 99% confidence intervals. If stratified data are entered, the assumed common values of the GOR are displayed (with their 96% confidence intervals); these are weighted averages of the stratum-specific GOR values, and are of questionable value is there is marked heterogeneity.

The program provides odds ratios based on the contrast between each pair of categories (if there are up to 10 categories). If the odds ratio based on the contrast between two categories, e.g. 1 and 2 (displayed as “1:2”) is above 1, this means that the odds in favour of 1 rather than 2 are higher in sample A than in sample B.

The consistency of these odds ratios based on pairs of categories is tested. For example, if the odds ratio for category 1 versus category 2 is 3.0 and the odds ratio for category 2 versus category 3 is 4.0, the odds ratio for category 1 versus category 3 would be expected to be 12.0. Inconsistency with such expectations suggests that the odds ratios may be modified by the matching variables (Pike, Casagrande, and Smith 1975). A low P value is indicative of inconsistency.

Maximum-likelihood estimates of mutually consistent odds ratios based on the contrast between each pair of categories are computed; these estimates are not very meaningful if the test points to mutual inconsistency.

The program also computes odds ratios based on a comparison of each category with all other categories combined, and does McNemar tests to appraise their significance; alternative P-values are provided for tests of hypotheses formulated before and after seeing the results.

Confidence intervals are displayed for odds ratios contrasting each category with the reference category (the category with the highest category number), assuming mutual consistency.

Kappa and related results

As measures of the agreement between the matched observations, the program provides a *weighted kappa* (which takes account of the sequence of the categories), an ordinary *overall kappa* (for the complete set of categories, but ignoring the sequence), and (if there are up to six categories) separate kappa values for each category. In each instance, a one-tailed test is done, indicating whether kappa is significantly higher than zero. If kappa is 0.4 or more, a second test is done, indicating whether it is significantly higher than 0.4; and if it is 0.6 or more, a third test is done, indicating whether it is significantly higher than 0.6. Confidence intervals are estimated from the standard error.

In the computation of weighted kappa, the weight given each pair of observations depends on the size of the difference between the categories in which the pair-mates fall. Default scores

of 1, 2, 3, etc. are allotted to the categories for this purpose; but these scores can optionally be changed to numbers that are believed to better express the relative closeness of the categories.

Paradoxical values of *kappa* may occur because of bias (systematic one-sided variation between two ratings) – indicated by the extended McNemar test (see above) – or a skewed distribution (inequality between the prevalences of the categories in the two samples). Two adjusted values of the overall kappa – BAK (*bias-adjusted kappa*) and PABAK (*prevalence-adjusted bias-adjusted kappa*) – are therefore computed (Byrt *et al.* 1993*).* These adjusted values are conditional on the observed percentage agreement. BAK is the value that *kappa* would take if there were no systematic one-sided variation between the ratings; it is equivalent to Scott's *pi* coefficient of agreement (Scott 1955). Low *kappa* values are likely to be affected by such bias. PABAK is the value that *kappa* would take if, in addition, the prevalence of each category (as expressed by the mean of the two raters' totals for the category) was equal. PABAK may be useful in appraising agreement when the percentage agreement is high and *kappa* is paradoxically low; it approximates to the highest possible *kappa* if the percentage agreement is above about 50% (Lantz and Nebenzahl 1996). PABAK is called *kappa-no*r by Lantz and Nebenzahl (1996), and is equivalent to Maxwell's *RE* (random error) coefficient of agreement (Maxwell 1977) and Bennett's *S* coefficient (Bennett *et al.* 1954). It should be noted that simulation studies have suggested that PABAK may substantially overestimate agreement (Hoehler 2000).

The program also displays the *maximum attainable overall kappa* consistent with the marginal totals, i.e. consistent with the observed level of bias.

Kappa is generally used to measure the agreement between two ratings (by different observers or tests, or by the same observer on different occasions) of the same individuals. In addition to this use as a measure of reliability, it may be used to measure concordance in other situations where paired samples are compared (Fleiss 1981: 232-233). In a matched case-control study or matched-control trial, *kappa* may serve as an indication of the effectiveness of a matching procedure – it indicates the extent to which the findings in matched pairs are more similar than findings in individuals from different pairs (Fleiss 1981: 233). Uses and misuses of *kappa* in epidemiology are discussed by (among others) MacLure and Willett (1987), Thompson and Walter (1988a, 1988b) and Kraemer and Bloch (1988).

The probability of chance agreement is taken into account in the calculation of *kappa*. A value of 1 indicates perfect agreement (after allowing for this probability of chance agreement) between ratings; 0 indicates no agreement other than what can be attributed to chance, and a negative value indicates less than chance agreement. Fleiss (1981: 218) suggests that a value of 0.75 or more indicates excellent agreement, and 0.40 or less indicates poor agreement. Alternative guidelines are: over 0.80, very good agreement; 0.61-0.80, good; 0.41-0.60, moderate; 0.21-0.40, fair; and 0.20 or less, poor agreement (Altman 1991). These levels may be taken into account in the appraisal of confidence intervals, e.g. by seeing whether the lower confidence limit lies above 0.40 (Basu and Basu 1995).

The *percentage agreement* is also shown. This is the percentage of individuals who are placed in the same category by both ratings, and (unlike *kappa*) it is not corrected for chance agreement. In a study in which the same individuals are rated by two observers, this is the percentage of subjects who are placed in the same category by both raters. The percentage agreement is also shown separately for each category (if there are up to six categories). In

clinical practice, the percentage of agreement for a specific rating represents the probability that, if a subject has been given that rating by a typical observer, another typical observer will concur.

If *stratified data* are entered (e.g. observations of individuals in different age groups), the heterogeneity of the overall kappa values in the different strata is tested, measures of heterogeneity (see above) are provided, and two estimates of the overall kappa are computed, with their confidence intervals. The first estimate of the overall kappa is precision-based; it is produced by weighting each kappa by the inverse of its variance (Fleiss 1981: 222). The second estimate is obtained by weighting the kappa values by the sizes of the samples in the strata. A simulation study suggests that this is preferable to the precision-based method if kappa is not zero (Barlow *et al*. 1991). A heterogeneity test is done, and supplemented by two measures of heterogeneity, *H* and *I-squared* (Higgins and Thompson 2002), with their approximate 95% intervals. An *H* value of less than 1.2 suggests absence of noteworthy heterogeneity, whereas a value exceeding 1.5 suggests its presence, even if the heterogeneity test is not significant. *I-squared* expresses the proportion of variation that can be attributed to heterogeneity (in a meta-analysis, to interstudy variation) rather than to sampling error. Overall values of the percentage agreement are reported. These are based on the pooled data; this is equivalent to weighting the stratum-specific values by sample sizes.

Distinguishability of categories

A measure of the distinguishability of pairs of categories is computed. This may be useful in a methodological study in which the matched observations represent separate ratings. The value is 100% if there are no disagreements, and zero if disagreements outnumber agreements.. The average of the values for all pairs of categories is displayed. and the least distinguishable pair of categories is identified.

Rank correlation coefficients and other measures of ordinal association

Kendall's and Spearman's rank correlation coefficients (*tau b* and *rho*, respectively) are computed. These have different numerical values but are similar in their ability to detect associations (Siegel and Castellan 1988: 251). The other measures of ordinal association that are provided are Goodman and Kruskal's *gamma* and Somers' asymmetric *D*, which may be regarded as measures of how effectively the order of a pair of observations with respect to one observation can be predicted from their order with respect to the other observation (see Hildebrand, Laing, and Rosenthal 1977). The Somers' *D* statistics are appropriate when one of the observations is clearly the dependent one, e.g. one that comes later in time; Somers' *Dxy* is appropriate when A is dependent, and *Dyx* when B is dependent.

Tau, Kruskal's *gamma*, and Somers' *D* depend on a comparison of the ranks of the paired observations. All possible pairs are taken into account in the computation of *tau*, whereas pairs that tie are disregarded in the calculation of *gamma*, and pairs that tie with respect to one (the independent) observation are omitted from the computation of Somers' *D*. *Tau* is the geometric average of *Dxy* and *Dyx*.

METHODS

Maximum categories = 60 [50 for kappa].

Tests for the difference between paired observations

The *Mann-Whitney test for paired data* is described by Agresti (1984: 208-209). If stratified data are entered, the results of the tests in the strata are combined by Stouffer's method (Stouffer *et al.* 1949: 5; DeMets 1987), based on weighted averages of the test results in the strata, using three different sets of weights for the *Z* values –weighting them equally, by the sample sizes in the strata, and by the square roots of the sample sizes. A simple test is also done on the combined (pooled) data.

The *Fleiss-Everitt test* for ordered categories is described by Fleiss (1981: 122-123).

The *Wilcoxon signed-ranks test* uses the formula provided by Siegel and Castellan (1988: 92, formula 5.5), but allowing for the effect of ties on the variance by replacing the denominator (as suggested by Sprent 1993: 53 and Mehta and Patel 1991: 7-10) by $\sqrt{\sum(R_i / 4)}$, where R_i = the rank of the difference between paired observations. Nondiscrepant pairs are ignored. If there are fewer than 20 pairs, significance is appraised by using critical levels for one-tailed P = .05, .025, .01, .005, .0025, and .0005 (derived from Siegel and Castellan 1988: Table H; and Zar 1998: Table B.12).

The *permutation test* is explained by Siegel and Castellan (1988: 95-100).

The *extended McNemar test* is described by Bowker (1948), Everitt (1977: 114-115) and Zar (1998: formula 9.22). Corresponding cells that both have zero values are omitted from the calculation of this chi-square. The contributions that specific pairs of categories make to a significant chi-square ($P < 0.05$) are computed by formula 6 of Maxwell (1970). In McNemar tests for single categories, the degrees of freedom are defined as $k-1$ (where k = number of categories) for testing *a posteriori* hypotheses (Fleiss 1981: 121).

The *Stuart-Maxwell chi-square test* is computed by Fleiss and Everitt's method (Fleiss 1981: 120-122; Everitt 1977: 115-116).

Heterogeneity test

The tests for goodness of fit with a symmetry model, on which the heterogeneity test is based, are described by Agresti (1984: 202).

Odds ratios and related tests

The *generalized odds ratio*, which is Agresti's α′ (Agresti 1980), is computed by the formula provided by Lui (2004: 126), and its 95% confidence interval by the logarithmic-transformation method of formula 6.14 (Lui 2004: 127). For stratified data, the assumed common value of the GOR is the exponent of a weighted average of the logs of the GOR values in the strata, and its 95% confidence interval is computed from the estimated variance of this weighted average (Agresti 1980: 63).

If there is a zero observed frequency of pairs in any cell, 0.5 isadded in each cell.

The test for the consistency of odds ratios between pairs of categories, the maximum-likelihood estimation of mutually consistent odds ratios, and the estimation of confidence intervals are described by Pike, Casagrande and Smith (1975).

Kappa and related results

The basic formulae are provided by Fleiss (1981: chapter 13). Kappa for single categories and for the total distribution (*overall kappa*) are calculated by formulae 13.10 to 13.12, and *weighted kappa* by formulae 13.27 to 13.29, with weights calculated by a formula (13.31) suggested by Cicchetti and Allison (1971). For tests of the null hypothesis that kappa is zero (formulae 13.14 and 13.35), the standard error (for an underlying zero value of kappa) is calculated by formula 13.13. For tests of the hypothesis that kappa has an underlying value other than zero, and for confidence intervals, the standard error appropriate for non-zero values is calculated by

formulae 13.15 to 13.18. Confidence intervals are estimated from the standard error (if the upper confidence limit exceeds 1, it is reduced to 1).

Bias is appraised by the extended McNemar (symmetry) test (see above), and BAK (*bias-adjusted kappa*) and PABAK (*prevalence-adjusted bias-adjusted kappa*) by the methods described by Byrt *et al.* (1993).

In the combined analysis of several samples or strata, the estimate of the supposed *common or overall value* of *kappa* is calculated in two ways: by computing a weighted mean, using the inverse of the variance of each *kappa* as its weight (Fleiss 1981: formula 13.21); and by computing a weighted mean, using the size of the stratum as the weight. The confidence intervals of the common kappa are estimated by Fleiss's formula 13.23.

The *heterogeneity test* is based on Fleiss's formula 13.22. The *measures of heterogeneity* (Higgins and Thompson 2002) are *H* and *I-squared*. *H* is computed by Higgins and Thompson's formula 6, and increased to 1 (indicating absence of heterogeneity) if it less than 1. A test-based interval is computed by Method III. *I-squared* and its 95% interval are computed from *H*, using formula 10.

Some computations are omitted if division by zero or other problems are encountered. In some instances, zero values are changed to 0.00001 to permit computation.

Distinguishability of categories

This measure is computed by the method described by Darroch and McLeod (1986).

Rank correlation coefficients and other measures of ordinal association

The computation of *tau*, *gamma*, and Somers' *D* is based on *S*, the difference between the numbers of concordant and discordant pairs, as explained by Kendall (1970: 45-46) and Agresti (1984: 157-159).

The formula for *tau* makes allowance for tied observations (Siegel and Castellan 1988: 249, formula 9.10). If the number of pairs N > 30, the significance of *S* is tested by a large-sample method whose use Agresti (1984: 180) suggests if the numbers of concordant and discordant pairs both exceed 100. If this condition is not met the program reports P as approximate. The formula is

$$Z = (S - CC) / \sqrt{V}$$

where V = variance of S, making allowance for tied ranks (Kendall 1970: formula 4.3)

As recommended by Kendall (1970:54-58), $CC = 1$ unless one variable has only two values and the other has tied ranks, in which case

$$CC = [(2N - T_F - T_L) / \text{Intervals}] / 2$$

where Intervals = the number of different ranks for the non-dichotomous variable, minus one

T_F and T_L = ties involving the first and last ranks (respectively) of the non-dichotomous variable

Gamma is calculated by a formula provided by Siegel and Castellan (1988: 292, formula 9.32). If N > 30, the significance test for *S* (see above) is used as a test for *gamma.*

Somers' *Dxy* and *Dyx* are calculated by Siegel and Castellan's formulas 9.41 and 9.42 (1988: 304-305). Significance is tested by a Z test (Siegel and Castellan 1988: 309, formula 9.47), based on the variance computed by Siegel and Castellan's formula 9.45.

Spearman's *rho* is computed by a formula that takes account of tied ranks (Siegel and Castellan 1988: 241, formula 9.7). It is not calculated if numbers are too large for the program to handle. The *t*-test for the significance of *rho* (Siegel and Castellan 1988: 243, footnote), used if N > 30, is based on the null variance. An approximate 95% confidence interval 8(Zar 1996: 398) is estimated if N is 10 or more and *rho* is 0.9 or less, based on the Fisher *z* transformation

$$z = 0.5\ln[(1 + rho) / (1 - rho)]$$

The confidence limits for *rho* {Fieller, Hartley and Pearson (1957, 1961) are

$$\exp[2(z \pm 1.96SEz) - 1] / \exp[2(z - 1.96SEz) + 1]$$

where $SEz = \sqrt{}[1.06 / (N - 3)]$.

D1. PAIRED NUMERICAL OBSERVATIONS (NORMAL DISTRIBUTION)

This module is appropriate for the analysis of paired numerical observations (in different subjects or the same subject) where a normal distribution is assumed. It appraises differences and agreement between the two sets of observations It can be used to analyse matched-control trials and case-control studies, before-after studies, reliability studies, comparisons of measurement methods, and other comparisons of paired subjects or observations.

The observations entered may be measurements in paired subjects, e.g. matched cases and controls, or replicated measurements in the same subjects. Each pair of matched observations (labelled "A" and "B") can be entered in a separate line, or pairs with the same values can be entered together, with their frequency; up to 500 lines may be entered. Replicated measurements can be entered in any order, unless “A” and “B” represent defined instruments, observers, times, conditions, etc.

If the data are stratified, enter each stratum in turn. Click on “All strata” whenever combined results are required.

The program provides a **comparison of the paired observations (**including the Bradley-Blackwood test, Student's paired *t*-test, and Pitman's test), **measures of agreement** (correlation coefficient, six intraclass correlation coefficients, Lin’s concordance correlation coefficient, repeatability coefficients, the standard error of measurement, the confidence interval for the “true value” corresponding to an observed measurement, Spearman-Brown coefficients of reliability, St Laurent's correlation coefficient, 95% limits of agreement , and the association between the difference and the mean value**),** and **ANOVA tables.**.

If *stratified data* are entered, the paired one-tailed *t* tests in the separate strata are combined, and the *heterogeneity* of the P-values in the strata is tested.

Comparison of the paired observations

The program displays means, standard deviations and standard errors for the two sets of observations, and the mean difference between the observations, with its standard deviation, standard error and 90%, 95% and 99$ confidence intervals. It also provides linear regression coefficients, with their standard errors.

The tests for differences are the Bradley-Blackwood test, which simultaneously tests the means and variances (Bradley and Blackwood 1989; Bartko 1994), Student's paired *t*-test, which compares the means, and Pitman's test for the equality of variances. Two-tailed P-values are displayed.

If *stratified data* are entered, the paired one-tailed *t* tests in the separate strata are combined by Stouffer’s method (Stouffer et al. 1949, p. 45; DeMets 1987) to produce overall one-tailed tests that control for the stratifying variables. Three different sets of weights are used for this purpose – weighting the test results equally, by the sample sizes in the strata, and by the

square roots of the sample sizes. In addition, the *heterogeneity* of the P-values in the strata is tested.

Measures of agreement

The measures of agreement have special relevance to studies of reliability, comparisons of measurement methods, and the clinical application of measurements.

A simple correlation coefficient, intraclass correlation coefficients, and Lin's concordance correlation coefficient are computed in all instances.

If the paired observations are positively correlated the program also provides measures that may be useful if A and B are replicate measurements, or if they denote two different methods of measurement. The measures for use in studies of replicate measurements are repeatability coefficients, the standard error of measurement, and the confidence interval for the "true value" corresponding to an observed measurement. The measures that are appropriate in comparisons of measurement methods are St Laurent's correlation coefficient (for use if one of the methods is regarded as a "gold standard"), and 95% limits of agreement. The program displays the correlation coefficient between the difference and mean of A and B, and the linear regression of the difference on the mean.

The simple *correlation coefficient* is seldom helpful in comparisons of methods of measurement (Bland and Altman 1995a; Altman 1991: 401-402), since at best it points to an association between the measurements, and does not tell how closely they agree; moreover, its value tends to be high if the subjects are very different, and low if they are similar.

Intraclass correlation coefficients, which are appropriate for interval-scale data with an assumed normal distribution, are measures of agreement that express the correlation (in terms of absolute agreement) between measurements within individuals or sets of matched individuals. Six intraclass correlation coefficient (ICC) values are computed (Shrout and Fleiss 1979), with their 95% confidence intervals.

Each ICC is appropriate in a different situation. (a) The values with the rubric "two-way model with fixed raters" are appropriate in studies where the matched observations in each set represent various "unique" raters, and no inferences are made about other raters; "raters" denote the various observers, treatments, methods or conditions of observation, matched individuals, or (in a reliability study of a questionnaire or other scale) questions or other scale items, that were studied. Two such ICCs are provided. The first, which Shrout and Fleiss refer to as model 3.1, uses a single measurement as the unit of analysis, and the second (model 3,k) uses an average measurement. (b) The two ICC values reported as "two-way model with random raters" are appropriate if the raters were randomly selected from a larger population of raters and it is proposed to generalize the findings to this larger population. If analysis is based on a single measurement, this is model 2,1; if it based on an average measurement, it is model 2,k. (c) The third pair of ICC values, entitled "one-way random model", is appropriate in methodological or other studies where the measurements are replications by the same observer or using the same instrument, and the order in which they are entered does not matter (this does not apply to the other ICC values).. They apply to the use of a single measurement (model 1,1) – e.g. in studies to determine the reliability of a single measurement – or to an average measurement (model 1,k) – e.g. in studies to determine the reliability of an average measurement.

The maximum value of an ICC is 1; the lower limit is an indeterminate negative value. As a rule of thumb, it has been suggested that ICC values above 0.75 should be regarded as evidence of excellent, and values above 0.4 as evidence of good, reliability (Shoukri and Pause1998: 27).

In the appraisal of replicated measurements a low ICC may express variability of the characteristic measured, as well as low reliability of measurement; this is especially important if measurements were conducted at different times. The usefulness of the ICC in comparisons of two methods of measurement (Bartko 1994; Lee 1992) is constrained by these and other limitations (Muller and Buttner 1994; Bland and Altman 1995a).

The *concordance correlation coefficient* is computed with its 95% confidence interval. Suggested by Lin (1989) as an improved measure of the reproducibility of measurements, its use is appropriate in comparisons where the two observers (or measurement methods) are selected “at random” to represent all observers (or measurement methods) to whom the assessed consistency relates; whereas if they are “fixed”–- e.g. in a comparison of two kinds of measuring instrument – it is more appropriate to use the intraclass correlation coefficient (Mueller and Buettner 1994). The Fisher *z* transformation of the coefficient is displayed, with its standard error, for use if the findings are to be compared with those in a different set of paired observations; (for this purpose, the standard error of the difference between two *z* transformations is the square root of the sum of their variances). The value of any correlation coefficient, including Lin's concordance coefficient, is affected by the *range of values* included in the analysis (Lin and Chinchilli 1997) - the wider the range, the stronger the correlation - and this should be taken into account when coefficients are appraised or coefficients based on different samples are compared. The program therefore reports this range (the range of the means of paired values).

The *coefficients of repeatability* express the expectation (with 95% confidence) of the maximum size of the absolute difference between paired observations. Two coefficients are provided, with their approximate confidence intervals. The first (Bland and Altman 1986; Chinn 1990) is valid if there is no bias (no systematic difference between the observations), i.e. if the mean difference between observations is zero; this may not be so if the measurement process alters the quantity or if knowledge of the first measurement affects the second. The second coefficient controls for any effect of bias; it is based on the residual within-subjects sum-of-squares, after removal of the between-ratings component.

The *standard error of measurement* (Fleiss 1986: 11) –.also called the “technical error” (Kahn and Sempos 1989: 239-242) or “the SE of an obtained score” (Guilford and Fruchter 1986: 413) – is an index of reliability that expresses variation between observers and other causes of differences between repeated observations. To aid in its interpretation, its ratios to the standard deviation among persons and to the mean value are displayed.

The program computes an approximate 95% *confidence interval for the “true value”* corresponding to an observed measurement or the mean of two or three measurements. These should be used with caution, since they assume that the width of the confidence interval is independent of the magnitude of the value (Guilford and Fruchter 1986: 413).

St Laurent's gold-standard correlation coefficient is a measure of criterion validity – it is a measure of the agreement between a measurement and a “gold standard” (St Laurent 1998).

Two values are displayed, with their approximate 95% confidence intervals, taking A or B in turn as the "gold standard". The procedure assumes that the "gold- standard" measurements and the differences between the two sets of measurements are normally distributed.

The *95% limits of agreement* (Bland and Altman 1995a, 1995b; Altman 1991: 397-400) answer the question, "given a measurement by one method, how far might this be from a measurement by the other method?" These demarcate the bounds of the range that, with a 95% probability, includes the difference between single measurements of the same subject by the two methods. The 95% confidence intervals of the limits of agreement are estimated (the limits of agreement may be very imprecise if the sample is small).

Use of the 95% limits of agreement assumes that the differences are reasonably constant throughout the range of measurement. To check this assumption, the program displays the *coefficient of correlation between the difference and the mean* of the two values, and the *regression of the difference on the mean.* The correlation and regression coefficients may be expected to be zero if the mean difference and the scatter of differences do not change with increasing values. If the difference and the mean are correlated, it may be appropriate to repeat the computation after log-transformation of the measurements, since the difference between log-transformed values may not change with increasing values. (To do this, click on "Repeat", then on "Lognormal distribution assumed", and then on "Run".)

Even when one of the methods of measurement is a new one and the other is an accepted standard, it is preferable to examine the relationship between the difference and the mean value rather than the relationship between the difference and the standard measurement, which (as shown by Bland and Altman 1995b) is likely to be misleading.

Spearman-Brown coefficients of reliability provide estimates of the effect of using the means of replicated observations. They predict what the reliability would be if two, three, four, or five replications were averaged.

ANOVA tables

If the paired observations are positively correlated, an analysis of variance (ANOVA) table for the linear regression between the difference between the two ratings and the mean of the two ratings is displayed.

In all instances, a two-way mixed model ANOVA table is displayed, showing between-subjects, within-subjects and between-ratings sums of squares. (The P-values based on F tests in the ANOVA tables are one-tailed.)

METHODS

To avoid computational problems in extreme situations, zero divisors are replaced by 0.000001.

Comparison of the paired observations

Formulae for the Bradley-Blackwood test, Student's paired t-test, and Pitman's test are provided by Bartko (1994). Linear regression methods are explained in all basic statistics textbooks.

If *stratified data* are entered, the results of the one-tailed *t* tests in the strata are combined by Stouffer's method (Stouffer *et al*. 1949: 5; DeMets 1987), based on weighted averages of the *z* values computed for each test by transforming its one-tailed P-value to the corresponding normal score (Hedges and Olkin 1985: 39). Three different sets of weights are used — weighting the *z* values equally, by the sample sizes in the strata, and by the square roots of the sample sizes. In addition, the heterogeneity of the P-values in the strata (Wolf 1986: 45) is tested. The heterogeneity test uses Wolf's formula:

$$\text{chi-square } (k - 1 \text{ d.f.}) = \sum(z_i - \text{MeanZ})^2$$

where k = number of strata,
z_i = z value in stratum $_i$
MeanZ = mean z value.

Measures of agreement

The significance test for the *correlation coefficient* uses Hotelling's modified *z* transformation (Sokal and Rohlf 1981: 583-587) if N < 30.

The following formulae (Shrout and Fleiss 1979) are used for the six intraclass correlation coefficients. Shrout-Fleiss ICC models 1,1 and 1,k are computed from a one-way random effects model ANOVA, models 2,1 and 2,k from a two-way random effects model ANOVA, and models 3,1 and 3,k from a two-way mixed effects model ANOVA.

$$\text{ICC model 1,1} = (\text{MSB} - \text{MSW}) / [\text{MSB} + (\text{k} - 1)\text{MSW}]$$
$$\text{ICC model 1,k} = (\text{MSB} - \text{MSW}) / \text{MSB}$$
$$\text{ICC model 2,1} = (\text{MSB} - \text{MSE}) / [\text{MSB} + (\text{k} - 1)\ \text{MSE} + \text{k}(\text{MSJ} - \text{MSE}) / \text{N}]$$
$$\text{ICC model 2,k} = (\text{MSB} - \text{MSE}) / [\text{MSB} + (\text{MSJ} - \text{MSE}) / \text{N}]$$
$$\text{ICC model 3,1} = (\text{MSB} - \text{MSE}) / [\text{MSB} + (\text{k} - 1)\text{MSE}]$$
$$\text{ICC model 3,k} = (\text{MSB} - \text{MSE}) / \text{MSB}$$

where MSB = between-subjects mean square
MSE = residual within-subjects mean square
MSW = within-subjects mean square
N = number of subjects
k = number of observations in matched set

Formulae for confidence intervals for the six ICC models are provided by McGraw and Wong (1996) in their Table 7, where they are referred to as ICC(1) and ICC(k) for Case 1, and ICC(A,1) and ICC(A,k) for Cases 2 and 3, The formulae (except those for models 2,1 and 2,k) are set out in a convenient code by Steinley and Wood (2000). Linear interpolation is used to estimate F values that are based on non-integer degrees of freedom (and 1 d.f. is substituted for <1 d.f.) in the computation of confidence intervals for models 2,1 and 2,k; the latter results may differ slightly from those provided by SPSS, which handles non-integer degrees of freedom differently.

Intraclass correlation coefficients are not computed if the correlation coefficient is 1 or –1.

The *Spearman-Brown prediction formula* (Fleiss 198: 14-15: formula 1.3) for reliability (*R)* is

$$R = \text{N}r / [1 + (\text{N} - 1)r]$$

where N = number of replicates that are averaged
r = intraclass correlation coefficient (model 1,1)

Fleiss's formula 1.31 is used to estimate the number of replicates required to obtain a reliability of 0.75 or 0.8:

$$N = P(1 - r) / [r(1 - P)]$$

where P = 0.75 or 0.8

The *concordance correlation coefficient* is computed by formula 19.76 of Zar (1998: 409), with *n* substituted for (n – 1) in the denominator, and its 95% confidence interval is based on variance formula 2 of Lin (1989), as corrected by Lin (2000). [Version 1.14 and earlier versions of PAIRSetc used Zar's formulae, which yield slightly different results.] The confidence interval is not computed if the correlation coefficient is 1 or –1.

The formulae for the two *repeatability coefficients* (Bland and Altman 1986; Chinn 1990) are

$$1.96\sqrt{(\text{D}^2 / \text{N})} \text{ or}$$
$$1.96\sqrt{(2.\text{SSW} / \text{N})}$$

and (controlling for any effect of bias)

$$1.96\sqrt{[2.SSE / (N - 1)]}$$

where D = difference between paired observations
N = number of pairs
SSW = within-subjects sum-of-squares
SSE = residual within-subjects sum-of-squares (excluding the between-ratings component).

Approximate 95% confidence intervals are obtained by substituting confidence limits for SSW and SSE, estimated by the method described by Zar (1998: formula 7.16), in the above formulae.

The formula for the *standard error of measurement* SEm is provided by Kahn and Sempos (1989: 240). SEm is also the square root of the within-subjects mean square shown in the ANOVA table (Fleiss 1986: 11). The formula for the SD among persons is also provided by Kahn and Sempos (1989: 241).

The 95% *confidence intervals for the "true value"* are estimated from the SD of the differences between values, by the method described by Peat *et al.* (1994); the *t*-distribution is used in the computation.

St Laurent's gold-standard correlation coefficient (St Laurent 1998) is computed by the formula

$$R_g = \sqrt{\{1 / [2B(1 / R_c) - 1] + 1\}}$$

where B = regression coefficient (slope) of the approximate measurement on the gold-standard measurement
R_c = concordance correlation coefficient.

An approximate 95% confidence limit is computed in accordance with St Laurent's Proposition 1.

The *95% limits of agreement* (Chinn 1991) are

$$D - 1.96(SD) \text{ and}$$
$$D + 1.96(SD).$$

The 95% confidence limits for the limits of agreement (Bland and Altman 1986; Altman 1991: 422-423) are estimated by subtracting and adding *t*.SE. In these formulae,
D = mean of the differences (Value 1 minus Value 2)
SD = standard deviation of the differences
$SE = \sqrt{[SD^2 / N) + (t^2.SD^2 / 2N)]}$, which reduces to $SD \sqrt{[2 + t^2]} / \sqrt{(2N)}$
t = the value in the *t* distribution corresponding to a two-tailed P of 0.05 with (N - 1) degrees of freedom
N = number of pairs

The *Spearman-Brown prediction formula* (Wikipedia) is

$$Nr / [1 + (N - 1)r]$$

where N = number of replicates that are averaged
r = intraclass correlation coefficient.

This application of the Spearman-Brown formula was suggested by its use by Solomon (2004).

ANOVA tables

The ANOVA tables are explained by Bartko (1994).

D2. PAIRED NUMERICAL OBSERVATIONS (LOGNORMAL DISTRIBUTION)

This module is appropriate for the analysis of paired numerical observations (in different subjects or the same subject) that have a lognormal distribution (such as, for example, bronchial responsiveness, recovery times after drug administrations, or the domestic house-dust allergen level). It appraises differences and agreement between the two sets of observations It can be used to analyse matched-control trials and case-control studies, before-after studies, reliability studies, comparisons of measurement methods, and other comparisons of paired subjects or observations.

It may be useful in reliability studies of a normally-distributed variable, if the simple difference between the observations under comparison is found to increase with the level of the measurement. In such instances, the difference between the logs of the observations (i.e., the ratio of the measurements) may be found to be reasonably constant throughout the range of measurement, facilitating estimation of the agreement between measurements.

Computations are based on the logarithms of the values that are entered, which may be measurements in paired subjects or repeated measurements in the same subjects. Each pair of matched observations (labelled "A" and "B") can be entered in a separate line, or pairs with the same values can be entered together, with their frequency; up to 500 lines may be entered.

If the data are stratified, enter each stratum in turn. Click on “All strata” whenever combined results are required.

The program provides a **comparison of the paired log-transformed observations**, including the Bradley-Blackwood test, Student's paired *t*-test, and Pitman's test, and **measures of agreement between the log-transformed observations** (correlation coefficient, intraclass correlation coefficient, Lin’s concordance correlation coefficient, repeatability coefficients, the standard error of measurement, the confidence interval for the “true value” corresponding to an observed measurement, St Laurent's correlation coefficient, 95% limits of agreement , and the association between the difference and the mean value**).**

If *stratified data* are entered, the paired one-tailed *t* tests in the separate strata are combined, and the *heterogeneity* of the P-values in the strata is tested.

Comparison of the paired log-transformed observations

The program displays the ratio of the paired values, with its confidence intervals.

The tests for differences between the log-transformed observations are the Bradley-Blackwood test, which simultaneously tests the means and variances (Bradley and Blackwood1989; Bartko 1994), Student's paired t-test, which compares the means, and Pitman's test for the equality of variances. Two-tailed P values are displayed.

If *stratified data* are entered, the paired one-tailed *t* tests in the separate strata are combined by Stouffer's method (Stouffer et al. 1949, p. 45; DeMets 1987) to produce overall one-tailed tests that control for the stratifying variables. Three different sets of weights are used for this purpose – weighting the test results equally, by the sample sizes in the strata, and by the square roots of the sample sizes. In addition, the *heterogeneity* of the P-values in the strata is tested.

Measures of agreement between the log-transformed observations

The measures of agreement have special relevance to studies of reliability, comparisons of measurement methods, and the clinical application of measurements.

A simple correlation coefficient, the intraclass correlation coefficient, and Lin's concordance correlation coefficient are computed in all instances.

If the paired observations are positively correlated the program also provides measures that may be useful if A and B are replicate measurements, or if they denote two different methods of measurement. The measures for use in studies of replicate measurements are repeatability coefficients, the standard error of measurement, and the confidence interval for the "true value" corresponding to an observed measurement. The measures that are appropriate in comparisons of measurement methods are St Laurent's correlation coefficient (for use if one of the methods is regarded as a "gold standard"), and 95% limits of agreement. The program displays the correlation coefficient between the ratio and mean of A and B.

The simple *correlation coefficient* is seldom helpful in comparisons of methods of measurement (Bland and Altman 1995a; Altman 1991: 401-402), since at best it points to an association between the (log-transformed) measurements, and does not tell how closely they agree.

The *intraclass correlation coefficient* (ICC) is a measure of agreement that expresses the correlation between measurements within individuals or pairs of matched individuals. It ranges from -1 to +1, zero indicating no agreement. The ICC is displayed with its significance level and 95% confidence interval. The ICC is affected by the degree of variation among the subjects, and may be misleadingly low if the subjects are very similar, or if differences between paired observations are large relative to the differences between subjects (Bartko 1994). In the appraisal of replicated measurements a low coefficient may express variability of the characteristic measured, as well as low reliability of measurement; this is especially important if measurements were conducted at different times. The usefulness of the ICC in comparisons of two methods of measurement (Bartko 1994; Lee 1992) is constrained by these and other limitations (Muller and Buttner 1994; Bland and Altman 1995a).

The *concordance correlation coefficient* is computed with its 95% confidence interval. Suggested by Lin (1989) as an improved measure of the reproducibility of measurements, its use is appropriate in comparisons where the two observers (or measurement methods) are selected "at random" to represent all observers (or measurement methods) to whom the assessed consistency relates; whereas if they are "fixed"–- e.g. in a comparison of two kinds of measuring instrument – it is more appropriate to use the intraclass correlation coefficient (Mueller and Buettner 1994). The Fisher *z* transformation of the coefficient is displayed, with its standard error, for use if the findings are to be compared with those in a different set

of paired observations; (for this purpose, the standard error of the difference between two *z* transformations is the square root of the sum of their variances).

The *coefficients of repeatability* express the expectation (with 95% confidence) of the maximum size of the absolute difference between paired log-transformed measurements (i.e., for the ratio of paired measurements). Two coefficients are provided, with their approximate confidence intervals. The first (Bland and Altman 1986; Chinn 1990) is valid if there is no bias (no systematic difference between the observations), i.e. if the mean difference is zero; this may not be so if the measurement process alters the quantity or if knowledge of the first measurement affects the second. The second coefficient controls for any effect of bias; it is based on the residual within-subjects sum-of-squares, after removal of the between-ratings component.

The *standard error of measurement* (Fleiss 1986: 11) –.also called the “technical error” (Kahn and Sempos 1989: 239-242) or “the SE of an obtained score” (Guilford and Fruchter 1986: 413) – is an index of reliability that expresses variation between observers and other causes of differences between repeated observations. The standard error of measurement is expressed in logarithmic units.

The program computes an approximate 95% *confidence interval for the “true value”* corresponding to an observed measurement. This should be used with caution, since it assumes that the width of the confidence interval is independent of the magnitude of the value (Guilford and Fruchter 1986: 413).

St Laurent's gold-standard correlation coefficient is a measure of criterion validity – it is a measure of the agreement between a measurement and a “gold standard” (St Laurent 1998). Two values are displayed, with their approximate 95% confidence intervals, taking A or B in turn as the “gold standard”. The procedure assumes that the “gold- standard” measurements and the differences between the two sets of (log-transformed) measurements are normally distributed.

The *95% limits of agreement* (Bland and Altman 1995a, 1995b; Altman 1991: 397-400) answer the question, “given a measurement by one method, how far might this be from a measurement by the other method?” These demarcate the bounds of the range that, with a 95% probability, includes the difference between log-transformed measurements of the same subject by the two methods (i.e., the ratio of the measurements). The 95% confidence intervals of the limits of agreement are estimated (the limits of agreement may be very imprecise if the sample is small).

Use of the 95% limits of agreement assumes that the differences are reasonably constant throughout the range of measurement. To check this assumption, the program displays the *coefficient of correlation between the difference and the mean* of the two log-transformed values, and the *regression of the difference on the mean*. The correlation and regression coefficients may be expected to be zero if the mean difference and the scatter of differences do not change with increasing values

Even when one of the methods of measurement is a new one and the other is an accepted standard, it is preferable to examine the relationship between the difference and the mean value rather than the relationship between the difference and the standard measurement, which (as shown by Bland and Altman 1995b) is likely to be misleading.

METHODS

If zero values are encountered, 1 is added to all values before log-transforming them To avoid computational problems in extreme situations, zeroes are sometimes changed to 0.0000001 or 0.000001.

Comparison of the paired log-transformed observations

Formulae for the Bradley-Blackwood test, Student's paired *t*-test, and Pitman's test are provided by Bartko (1994). Linear regression methods are explained in all basic statistics textbooks.

If *stratified data* are entered, the results of the one-tailed *t* tests in the strata are combined by Stouffer's method (Stouffer *et al*. 1949: 5; DeMets 1987), based on weighted averages of the *z* values computed for each test by transforming its one-tailed P-value to the corresponding normal score (Hedges and Olkin 1985: 39). Three different sets of weights are used – weighting the *z* values equally, by the sample sizes in the strata, and by the square roots of the sample sizes. In addition, the heterogeneity of the P-values in the strata (Wolf 1986: 45) is tested. The heterogeneity test uses Wolf's formula:

$$\text{chi-square } (k - 1 \text{ d.f.}) = \sum(z_i - \text{MeanZ})^2$$

where k = number of strata,
z_i = z value in stratum $_i$
MeanZ = mean z value.

Measures of agreement between the log-transformed observations

The significance test for the *correlation coefficient* uses Hotelling's modified *z* transformation (Sokal and Rohlf 1981: 583-587) if $N < 30$.

The following coefficients are computed only if the correlation coefficient is positive.

The *intraclass correlation coefficient* that is computed is a mixed model ICC for two fixed ratings, assuming a two-way mixed analysis of variance model (Bartko 1994).

The *concordance correlation coefficient* is computed by formula 19.76 of Zar (1998: 409), with *n* substituted for ($n - 1$) in the denominator, and its 95% confidence interval is based on variance formula 2 of Lin (1989), as corrected by Lin (2000). [Version 1.14 and earlier versions of PAIRSetc used Zar's formulae, which yield slightly different results.] The confidence interval is not computed if the correlation coefficient is 1 or –1.

The formulae for the two *repeatability coefficients* (Bland and Altman 1986; Chinn 1990) are

$$1.96\sqrt{(D^2 / N)} \text{ or}$$

$$1.96\sqrt{(2.SSW / N)}$$

and (controlling for any effect of bias)

$$1.96\sqrt{[2.SSE / (N - 1)]}$$

where D = difference between paired observations
N = number of pairs
SSW = within-subjects sum-of-squares
SSE = residual within-subjects sum-of-squares (excluding the between-ratings component).

Approximate confidence intervals are obtained by substituting confidence limits for SSW and SSE, estimated by the method described by Zar (1998: formula 7.16), in the above formulae.

The formula for the *standard error of measurement* SEm is provided by Kahn and Sempos (1989: 240). SEm is also the square root of the within-subjects mean square shown in the ANOVA table (Fleiss 1986: 11). The formula for the SD among persons is also provided by Kahn and Sempos (1989: 241).

The 95% *confidence interval for the "true value"* is estimated from the SD of the differences between (log-transformed) values, by the method described by Peat *et al.* (1994); the *t*-distribution is used in the computation.

St Laurent's gold-standard correlation coefficient (St Laurent 1998) is computed by the formula

$$R_g = \sqrt{\{1 / [2B(1 / R_c) - 1] + 1\}}$$

where B = regression coefficient (slope) of the approximate measurement on the gold-standard measurement
R_c = concordance correlation coefficient.
An approximate 95% confidence limit is computed in accordance with St Laurent's Proposition 1.

The *95% limits of agreement* (Chinn 1991) are

D - 1.96(SD) and

D + 1.96(SD).

The 95% confidence limits for the limits of agreement (Bland and Altman 1986; Altman 1991: 422-423) are estimated by subtracting and adding t.SE. In these formulae,
D = mean of the differences (Value 1 minus Value 2)
SD = standard deviation of the differences
SE = $\sqrt{[SD^2 / N) + (t^2.SD^2 / 2N)]}$, which reduces to SD $\sqrt{[2 + t^2]} / \sqrt{(2N)}$
t = the value in the t distribution corresponding to a two-tailed P of 0.05 with (N - 1) degrees of freedom
N = number of pairs

D3. PAIRED NUMERICAL OBSERVATIONS (NORMALITY NOT ASSUMED)

This module is appropriate for the analysis of paired numerical observations (in different individuals or the same individual), where a normal or lognormal distribution is not assumed. It appraises differences and agreement between the two sets of observations It can be used to analyse matched-control trials and case-control studies, before-after studies, reliability studies, comparisons of measurement methods, and other comparisons of paired subjects or observations.

The observations entered may be measurements in paired subjects, e.g. matched cases and controls, or replicated measurements in the same subjects. Each pair of matched observations (labelled "A" and "B") can be entered in a separate line, or pairs with the same values can be entered together, with their frequency; up to 500 lines may be entered.

If the data are stratified, enter each stratum in turn. Click on “All strata” whenever combined results are required.

The program provides a **comparison of the paired observations**, including nonparametric tests (permutation test, Wilcoxon signed-ranks tests, and Hollander's test for bivariate symmetry), the median difference between the two values, and the median ratio of the two values in the population (with their confidence intervals), **measures of agreement** (concordance correlation coefficient, 95% limits of agreement for untransformed and log-transformed data}, **nonparametric regression analysis**, and **rank correlation coefficients and other measures of association**.

If *stratified data* are entered, the paired one-tailed Wilcoxon signed-ranks tests in the separate strata are combined, and the *heterogeneity* of the P-values in the strata is tested.

Comparison of the paired observations

The program displays the median and mean values in the two sets of observations, and the *median difference* between the two values in the population, with its approximate 95% confidence intervals. In a matched-control trial or before-after study, the median difference is an estimator of the treatment effect. On the assumption that the data come from distributions that are identical except in the magnitude of the values, these results express the difference between the population means, as well as the difference between the population medians.

The *median ratio* of the two values in the population, with its confidence intervals, is estimated in the same way, after log-transforming the observations. The results that are displayed are the exponents of the median difference computed from log-transformed values and its confidence limits.

The *permutation (randomization) test* for paired replicates is performed only if the number of pairs (N) is 25 or less. It may be slow, since it requires processing of 2^N possibilities, i.e. 33,554,432 if N = 25; optionally, the procedure can be aborted. The test is appropriate for

interval-scale variables. It assumes that the difference between paired observations is a measure of the difference in the characteristic that is measured; no assumptions are made about normality or other characteristics of the distribution. Exact one-tailed P-values are displayed if $P < 0.05$; the one-tailed value is doubled and shown as a two-tailed value.

The *Wilcoxon signed-ranks test* (Siegel and Castellan 1988: 87-95) tests whether the median discrepancy between paired observations is zero. It is appropriate if the differences between paired observations are an acceptable basis for ranking the differences in the characteristic that is measured. The test is based on the assumption that the distribution of intra-pair differences is symmetric around their median; if this condition is not met some statisticians suggest transformation of the data in order to enhance symmetry (Altman 1991: 204). The program provides a skewness index (0% = complete symmetry, 100% = extreme asymmetry in either direction) and (if there are no zero or negative values) repeats the test, using log-transformed values, which may reduce asymmetry.

If *stratified data* are entered, the one-tailed Wilcoxon sighed-ranks tests in the separate strata are combined by Stouffer's method (Stouffer *et al*. 1949, p. 45; DeMets 1987) to produce overall one-tailed tests that control for the stratifying variables. Three different sets of weights are used for this purpose – weighting the test results equally, by the sample sizes in the strata, and by the square roots of the sample sizes. In addition, the *heterogeneity* of the P-values in the strata is tested.

Hollander's test for bivariate symmetry (exchangeability) tests the null hypothesis that paired numerical observations are interchangeable; for example, in a before-after trial using the same subjects, that there is no treatment effect (Hollander and Wolfe 1999: 94-104). It is sensitive to differences between the paired observations and in their dispersion. The program uses a large-sample approximation (Hollander and Wolfe 1999: 96-97) to determine the P value; the results should be used with caution if the sample is small. A low P indicates that the paired observations are not interchangeable.

Measures of agreement

Lin's *concordance correlation coefficient* (Lin 1989 and 2000) is computed, with its 95% confidence interval. This is appropriate for appraising the similarity between paired measurements of the same subjects, with the aim of comparing two observers or measurement methods or appraising the reproducibility of a single method. The value ranges from 1 (complete agreement) to -1 (complete disagreement). The results should be regarded as approximate; although computer simulations have shown that the coefficient is robust and can cope with samples from non-normal distributions (Lin 1989); it is based on an assumption of normality.

The value of any correlation coefficient, including Lin's concordance coefficient, is affected by the *range of values* included in the analysis (Lin and Chinchilli 1997) - the wider the range, the stronger the correlation - and this should be taken into account when coefficients are appraised or coefficients based on different samples are compared. The program therefore reports this range (the range of the means of paired values).

The *95% limits of agreement* (Bland and Altman 1995a, 1995b; Altman 1991: 397-400) are appropriate when paired measurements of the same subjects have been entered in order to compare two observers or methods of measurement. The limits of agreement express the

range that, with approximately 95% probability, includes the difference between single measurements of the same subject by the two observers or methods, answering the question, "given a measurement by one observer or method, how far might this be from a measurement by the other observer or method?" The limits are unreliable if the sample is small, and are not displayed if under 20 pairs of observations are entered. Their confidence intervals should be regarded as rough approximations. The limits of agreement method assumes that the differences are reasonably constant throughout the range of measurement. As a check on this assumption, the program displays Kendall's rank correlation coefficient (*tau b*; see below) for the difference and the mean of the two values. This may be expected to be zero if the mean difference and the scatter of differences do not change with increasing values. Even when one of the methods of measurement is a new one and the other is an accepted standard, it is preferable to examine the relationship between the difference and the mean value rather than the relationship between the difference and the standard measurement, which (as shown by Bland and Altman 1995b) is likely to be misleading.

Approximate 95% limits of agreement are also computed for the ratio of the two measurements, together with Kendall's rank correlation coefficient for the relationship between the ratio and the geometric mean of the two values. This computation is based on log- transformed data, and is not done if there are zero or negative observations.

Nonparametric regression analysis

The nonparametric regression analysis procedure (which assumes interval-scale measurements) has the advantage of robustness – i.e., discrepant "outlier" observations have a reduced effect. Estimators of the intercept (*alpha*) and slope (*beta*) coefficients in the population are computed, with 90, 95, and 99% confidence intervals for the latter coefficients. Computation may be slow for large samples and can be aborted by the user. Computation is aborted if the samples are too large for the program to handle.

Three alternative ways of estimating *beta* are used, depending on the total number of pairs and the number of discrepant values. Two estimators of *alpha* are computed, and both are shown if they differ. The first estimator is recommended if it cannot be assumed that deviations from the regression line are symmetrical, and the second is recommended if the symmetry assumption is tenable.

Rank correlation coefficients and other measures of association

Kendall's and Spearman's rank correlation coefficients (*tau b* and *rho*, respectively) are computed. These have different numerical values but are similar in their ability to detect associations (Siegel and Castellan 1988: 251).

Goodman and Kruskal's *gamma* and Somers' asymmetric D may be regarded as measures of how effectively the rank of a pair of observations with respect to one observation can be predicted from their rank with respect to the other observation (see Hildebrand, Laing, and Rosenthal 1977). The *D* statistics are appropriate when one of the observations is clearly the dependent one, e.g. one that comes later in time; *Dxy* is appropriate when A is dependent, and *Dyx* when B is dependent.

Tau, Kruskal's *gamma*, and Somers' *D* depend on a comparison of the ranks of the paired observations. All possible pairs are taken into account in the computation of *tau*, whereas

pairs that tie are disregarded in the calculation of *gamma*, and pairs that tie with respect to one (the independent) observation are omitted from the computation of Somers' *D*. *Tau* is the geometric average of *Dxy* and *Dyx*.

A conservative ("outside") 95% confidence interval is estimated for *tau*. For small samples this estimate may be inordinately wide

METHODS

If zero values are encountered, 1 is added to all values before log-transforming them

Comparison of the paired observations

The estimation of the *median difference* and its confidence intervals is described by Campbell and Gardner (2000). For 25 or fewer pairs, the program uses critical values provided in Table 18.6 of Altman *et al*. (2000); for larger samples, it uses the formula provided by Campbell and Gardner (2000: 42). *The median ratio* of the two values in the population is estimated in the same way, after log-transforming the observations. The results that are displayed are the exponents of the median difference computed from log-transformed values) and its confidence limits.

The permutation test assumes that under the null hypothesis the differences between paired observations are equally likely to be positive or negative. Taking each of these possibilities for each pair, the sum of the differences is computed for each possible combination of findings. The P-value is the proportion of these outcomes that are as extreme as, or more extreme than, the outcome in the actual observations. The procedure is explained by Siegel and Castellan (1988: 95-100).

The *Wilcoxon signed-ranks test* uses the formula provided by Siegel and Castellan (1988: 92, formula 5.5), but allowing for the effect of ties on the variance by replacing the denominator (as suggested by Sprent 1993: 53 and Mehta and Patel 1991: 7-10) by √∑[Si] / 4), where Si = the square of the rank of the difference between paired observations. Nondiscrepant pairs are ignored. If there are fewer than 20 pairs, significance is appraised by using critical levels for one-tailed P = .05, .025, .01, .005, .0025, and .0005 (derived from Siegel and Castellan 1988: Table H; and Zar 1998: Table B.12). If the sample is larger a normal approximation is used, with allowance made for ties

The formula for the *skewness index* is

$$\text{abs}[(H - M) - (M - H)] / (H - L)$$

where M is the median of the observed intra-pair differences
H is their top decile
L is their lowest decile.

The deciles are determined by the methods explained by Zar (1998: 26-27). Pairs with no discrepancies are taken into account in the computation of the median difference and the skewness index, but not in the significance test.

If *stratified data* are entered, the one-tailed Wilcoxon sighed-ranks tests in the separate strata are combined by averaging their signed *z* values (Stouffer *et al*. 1949, p. 45; DeMets 1987). Three different sets of weights are used for this purpose – weighting the test results equally, by the sample sizes in the strata, and by the square roots of the sample sizes. In addition, the heterogeneity of the P-values in the strata is tested, using the formula (Wolf 1986: 45):

$$\text{chi-square } (k - 1 \text{ d.f.}) = \sum(Zi - \text{MeanZ})^2$$

where k = number of strata,
Z_i = z value in stratum i
MeanZ = mean z value.

Hollander's test for bivariate symmetry is described by Hollander and Wolfe (1999: 94-104).

Measures of agreement

The *concordance correlation coefficient* is computed by formula 19.76 of Zar (1998: 409), with n substituted for $(n - 1)$ in the denominator, and its 95% confidence interval is based on variance formula 2 of Lin (1989), as corrected by Lin (2000). [Version 1.14 and earlier versions of PAIRSetc used Zar's formulae, which yield slightly different results.] The confidence interval is not computed if the correlation coefficient is 1 or –1.

Approximate *95% limits of agreement* are computed by a nonparametric procedure described by Bland and Altman 1999. They are determined by excluding the lower and upper 2.5% of the observed distribution of differences. Their confidence intervals are based on confidence intervals for the relevant quantiles (Campbell and Gardner 2000: 39). They should be regarded as rough approximations, both because the method of computing confidence intervals for the quantiles assumes a normal distribution, and because when one confidence limit (lower or upper) falls outside the observed range of differences, it is arbitrarily placed at the same distance from the point estimate as the other confidence limit. The 95% limits of agreement for the ratio of the two measurements are computed in the same way, using log-transformed data.

Nonparametric regression analysis

The nonparametric regression analysis procedures are described by Daniel (1995: 622-625), Sprent (1993: 195-202) and Sen (1968).

Three alternative ways of estimating *beta* (the slope coefficient) are used, depending on the total number of pairs and the number of discrepant values. If up to 30 pairs of observations are entered, Theil's estimator (Theil 1950) is computed by a method described by Sprent (1993: 195-198). If more than 30 pairs are entered, Sen's method (Sen 1968) is used when possible. The program cannot cope with Sen's method if there are more than 146 pairs of observations with different values of the independent variable, and it then employs the abbreviated Theil method (Sprent 1993: 198-202), which uses a systematic sample of the data. For the Sprent and abbreviated Theil methods, which (unlike Sen's method) assume distinct values of the independent variable, the program treats tied observations as if they were not identical by imputing differences of (alternately) 0.000001 or -0.000001.

The point estimate of *beta* (b) is the median value of b_{ij}, where $b_{ij} = (y_j - y_i) / (x_j - x_i)$ for each pair of values of the independent variable x (x_i and x_j) and the corresponding values of the dependent variable y (y_i and y_j). Using Sprent's method, b_{ij} is calculated for all of the N(N-1)/2 possible pairs of values; zero values of (x-x_i) are changed to 0.000001 or -0.000001 (alternately). In Sen's procedure b_{ij} is calculated only if (x_j-x_i) is not zero. In the abbreviated Theil procedure the N pairs of observations are arranged with the values of the independent variable in a monotonically rising sequence, and each of the first N/2 pairs is then linked with the pair situated N/2 positions further along the array; b_{ij} is computed only for these linked observations; zero values of (x_j-x_i) are changed to 0.000001 or -0.000001.

Confidence intervals for *beta* are obtained from an array of values of b_{ij} in order of increasing magnitude. Sen's method (Sen 1968) uses critical values provided by a large-sample formula based on a variance estimate corrected for ties, and Sprent's method (Sprent 1993: 199-202) uses critical values based on the critical value for Kendall's tau for significance at nominal 10%, 5%, and 1% levels in two-tailed tests, obtained from Siegel and Castellan (1988: 363, TableRII) and Sprent (1993: Table IX). Approximate confidence intervals are estimated in a similar way in the abbreviated Theil procedure, using critical values based on formula 2.3 in Sprent (1993: 34).

Two estimators of the *alpha* coefficient are computed (Dietz 1989; Daniel 1995: 623-624). The first is the median of the (y_i - $b.x_i$]) terms for the N pairs of observations, and the second (Daniel 1995: 623-624) is the median of the averages of the (y_i - $b.x_i$) terms calculated for each of the pairwise combinations of observations. The second estimator of *alpha* is not calculated if the abbreviated Theil procedure is used.

Rank correlation coefficients and other measures of association

The computation of *tau*, *gamma*, and Somers' *D* is based on *S*, the difference between the numbers of concordant and discordant pairs, as explained by Kendall (1970: 45-46) and Agresti (1984: 157-159).

The formula for *tau* makes allowance for tied observations (Siegel and Castellan 1988: 249, formula 9.10). If the number of pairs N > 30, the significance of *S* is tested by a large-sample method whose use Agresti (1984:

180) suggests if the numbers of concordant and discordant pairs both exceed 100. If this condition is not met the program reports P as approximate. The formula is

$$Z = (S - CC) / \sqrt{V}$$

where V = variance of S, making allowance for tied ranks (Kendall 1970: formula 4.3)

As recommended by Kendall (1970:54-58), CC = 1 unless one variable has only two values and the other has tied ranks, in which case

$$CC = [(2N - T_F - T_L) \; / \; \text{Intervals}] \; / 2$$

where Intervals = the number of different ranks for the non-dichotomous variable, minus one

T_F and T_L = ties involving the first and last ranks (respectively) of the non-dichotomous variable

A conservative ("outside") 95% confidence interval is estimated for t*au*, using formula 4.12 of Kendall (1970: 64).

Gamma is calculated by a formula provided by Siegel and Castellan (1988: 292, formula 9.32). If N > 30, the significance test for *S* (see above) is used as a test for *gamma.*

Somers' *Dxy* and *Dyx* are calculated by Siegel and Castellan's formulas 9.41 and 9.42 (1988: 304-305). Significance is tested by a Z test (Siegel and Castellan 1988: 309, formula 9.47), based on the variance computed by Siegel and Castellan's formula 9.45.

Spearman's *rho* is computed by a formula that takes account of tied ranks (Siegel and Castellan 1988: 241, formula 9.7). It is not calculated if numbers are too large for the program to handle. The *t*-test for the significance of *rho* (Siegel and Castellan 1988: 243, footnote), used if N > 30, is based on the null variance. An approximate 95% confidence interval (Zar 1998: 392) is estimated if N is 10 or more and *rho* is 0.9 or less, based on the Fisher *z* transformation

$$z = 0.5\ln[(1 + rho) \; / \; (1 - rho)]$$

The confidence limits for *rho* {Fieller, Hartley and Pearson (1957, 1961) are

$$\exp[2(z \pm 1.96SEz) - 1] \; / \; \exp[2(z - 1.96SEz) + 1]$$

where $SEz = \sqrt{[1.06 / (N - 3)]}$.

If there are 30 or fewer pairs, the significance of t*au* is appraised by using critical levels for one-tailed P = 0.05, 0.025, 0.01, and 0.005 (Siegel and Castellan 1988: Tables RI and RII), and the significance of *rho* by using critical levels for one-tailed P = 0.05, 0.025, 0.01, 0.005, and 0.001 (Siegel and Castellan 1988: Table Q). If N > 30, a Z test is used for *tau* and *gamma*, and a *t*-test for *rho*. The Z test is appropriate for large samples, and P is reported as "approximate" if criteria suggested by Agresti (1984: 180) are not met.

D4. ANALYSIS OF PAIRED SURVIVAL DATA

This module is appropriate for the analysis of trials and follow-up surveys that study paired survival data, e.g. in paired individuals or in the two eyes of the same subjects.

A survival time ("time to event") is the number of time units (usually days or months) from the start of observation until the occurrence of a specified end-point event (such as death, the onset of a disease or complication, recovery from a disease, or return to work) or (if the event has not occurred) until withdrawal from observation. The main reasons for withdrawal, or *censoring*, are loss of contact, circumstances that dictate removal from the study, and conclusion of the study.

Each pair of survival times (A and B) may be entered separately, or (if specific paired values occur more than once) the paired values can be entered with their frequency.
Censored survival times are entered by appending "+", e.g .by entering "37+". Up to 500 pairs of survival times may be entered

To obtain results that are relevant to specific periods that are of interest, these periods can be entered (e.g., 24 months, to obtain information about 2-year survival).

The program provides a Kaplan-Meier life-table analysis for each group of observations (**cumulative survival proportions** with their 95% confidence intervals, **median and mean survival times**, and the **incidence rate** of the event), **comparisons of survival proportions,** the **hazard ratio** (with 95% confidence intervals), the **trends in the early and later periods of follow-up**, and **tests comparing the survival distributions (**Prentice-Wilcoxon and Gehan tests**)**.

Cumulative survival proportions

For each group of observations, the cumulative survival proportions (expressed as percentages) at each survival time entered are estimated by the Kaplan-Meier procedure. Cumulative survival proportions are also computed for any survival times that have been specified as of special interest, with their approximate 95% confidence intervals; these are large-sample limits, and Rothman and Greenland (1998: 289-90) recommend their use only if at least five events were observed and there are at least five survivors under observation at the time of the calculation; a warning is displayed if these conditions are not met.

The step-by-step survival proportions that are reported provide raw data for the construction of survival curves, consisting of horizontal lines with vertical steps whenever the survival proportion changes.

Median and mean survival times

Where possible, median and mean survival times are reported for each group of observations.

Whether survival times are censored or not, the median survival time is defined as the time at which the cumulative survival probability drops to 50% or below. An approximate standard error and 95% confidence interval are reported; these values may be inaccurate if the sample is small (Machin and Gardner 2000: 97)..

If the survival probability is not precisely 50% at the reported median survival time, an alternative median is also reported, based on linear interpolation between the times straddling the 50% mark.

The program also computes the median survival time expected if the distribution is exponential; if this is very different from the observed median, the assumption of exponentiality can be rejected..

The mean survival time is displayed, with its 95% confidence interval. If there are censored survival times, these values are estimates.

Incidence rate of the event

The average rate of events and its confidence intervals are estimated from the mean survival time and its confidence limits. If any survival times are censored, the rate is an estimate.

Comparisons of survival proportions

For specific survival times that have been specified as being of special interest, the program displays the difference between the survival proportions in the two groups of observations, and the ratio of these proportions, with their approximate 95% confidence intervals. The confidence intervals should be used with caution if the survival times were selected *a posteriori*, after examination of the data (Altman 1991: 376).

Hazard ratio

The hazard ratio, which is similar to a relative risk, expresses the relative survival experience of the two groups. The program also displays the values (in each group of observations) on which the hazard ratio is based – the number of observed events and the “extent of exposure” or “expected events”, and their ratio.

Trends in the early and later periods of follow-up

As a simple indication of possible time-related differences between the survival distributions, the program summarizes the change in the cumulative survival proportion in each group of observations, in the early and later segments of the follow-up period (usually using the median survival period for Group A as the cutting-point). The change is expressed as the drop in the survival percentage.

Comparison of the changes may point to trends that are different in the two groups or time periods. Differences in trend in the two periods may be obscured in the overall results.

Number needed to avoid one event

For use in studies in which the events are avoidable, the program reports the number of individuals who are needed in the group with a longer survival time, in order to avoid a single case.

Tests comparing the survival distributions

Two tests are performed: the Prentice-Wilcoxon test (Prentice 1978) and the Gehan test (Gehan 1965). Both tests allow for censored observations. One-tailed and two-tailed P values are shown.

When data are heavily censored, great differences can exist between the results of the two tests (O'Brien and Fleming 1987).

Computer simulations indicate that the Prentice-Wilcoxon test is more powerful in most situations, but the Gehan test may be more powerful if the survival times follow an exponential distribution (Woolson and O'Gorman 1992).

METHODS

Cumulative survival proportions

Cumulative survival proportions are estimated by the Kaplan-Meier technique (Kaplan and Meier 1958; Armitage *et al.* 2002: 575-576; Machin and Gardner 2000: 94-96).

95% confidence intervals for survival proportions at specific selected times are computed from the estimated variance of the logit of the proportion, using Greenwood's formula (Rothman and Greenland 1998: 289-90).

Median and mean survival times

The *median survival time* is defined as the time at which the cumulative survival probability drops to 50% or below. Its approximate standard error and 95% confidence interval are computed by the formulae provided by Machin and Gardner (2000: 97-98), based on the survival times at which the survival probabilities reach or cross the 45% and 55% levels, or if these probabilities are equal, the 40% and 60% levels. The effective sample size required for the calculation is the total sample size minus the number censored before the median survival time (Machin and Gardner (2000: 94). If the sample is small, the results are unreliable.

If the survival probability is not precisely 50% at the reported median survival time, an alternative median is also reported, based on linear interpolation between the times straddling the 50% mark (Selvin 1996: 374).

The median survival time expected if the distribution is exponential is the sum of the survival times (whether censored or not) divided by the number of events (Altman 1991: 385).

The **mean survival time** and its confidence intervals are computed in the usual way if no survival times are censored,. Otherwise, a nonparametric estimate of the mean is computed, based on formula 11.29 of Selvin (1996: 371); its standard error is computed by formula 11.31 and used for interval estimation; for this purpose, the longest survival time is treated as uncensored, even if it is censored.

A mean/median survival time is also computed, based on the assumption that the distribution is exponential (Selvin 1996, formula 11.19; Altman 1991: 385). Its standard error is computed by Selvin's formula 11.20.

Incidence rate of the event

Since (in a closed population) an incidence rate is the reciprocal of the average time until occurrence of the event (Rothman 1986: 29; Morrison 1979), the reciprocals of the mean survival time (or the estimate of the mean survival time) and its confidence limits are used as estimates of the average rate of events and its confidence limits.

Comparisons of survival proportions

For comparisons of survival proportions, the estimation of the variances and confidence intervals of the differences and ratios is described by Rothman and Greenland (1998, 291-292). Formulae 16-15 and 16-16 are used, based on. the estimated variances of the logits of the proportions (Rothman and Greenland 1998, pp 289-90). The proportions are treated as independent.

Ratio of median survival times

The computation of a confidence interval for the ratio of the median survival times in the two groups (Simon 1986), on the assumption that the survival times have an exponential distribution, is described by Altman (1991: 384-385). The median survival times used for this purpose are those at which the cumulative survival probability drops to 50% or below.

Hazard ratio

The program computes the Pike hazard ratio estimator (Pike 1972).

Trends in the early and later periods of follow-up

Changes in the survival percentage in each group of observations are reported, in the early and later periods of follow-up. The cutting-point used for this purpose is based on the median survival period for Group A (or, if this median is not reached, on the point at which the cumulative survival proportion drops to 60%). The longest survival time entered determines the end of the later period. Where possible, the interval defined for Group A is applied to Group B also. Linear interpolation is used where necessary.

Number needed to avoid one event

The number of individuals who are needed in the group with a longer survival time in order to avoid a single case is computed from the difference between survival proportions and its estimated variance (Altman and Anderson 1999),

Tests comparing survival distributions

The Prentice-Wilcoxon and Gehan tests are done in accordance with the detailed procedures set out by Woolson and O'Gorman (1992). For the Prentice-Wilcoxon test, the *delta* value for each pair of survival times is multiplied by the frequency of the combination, if it is more than 1.

E. COMPARISON OF SUBJECTS WITH TWO OR MORE MATCHED CONTROLS (“YES-NO” VARIABLE)

This module is appropriate for the analysis of case-control studies, clinical trials and cohort studies in which each index subject (each case, experimental subject, or individual exposed to a risk or protective factor) has a fixed number (2-20) of individually matched controls, and the dependent variable is dichotomous (“yes-no”), e.g. “yes” = exposure to a risk factor (in a case-control study), the success of a treatment, or the presence of a disease (in a cohort study). It compares the findings in the index subjects and their matched controls.

The program refers to index subjects as “cases”. The number of controls per case must be entered. Then each set of matched observations can be entered in a separate line, or sets with the same findings can be entered together, with their frequency. The required entries for each pattern of findings are 0 (“no”) or 1 (“yes”) for the “case”, and the number of matched controls with “yes”.

If the data are stratified, enter each stratum in turn. Click on “All strata” whenever combined results are required.

The program provides **tests for the difference** between the “cases” and their controls (exact Fisher's and mid-P tests, Mantel-Haenszel test, and Walter's test for binary data), the **odds ratio** (maximum-likelihood and Mantel-Haenszel estimates), and ***kappa.***

If *stratified data* are entered, the Walter’s tests in the separate strata are combined, the *heterogeneity* of the P-values in the strata is tested, and an overall *kappa* is computed .

Tests for the difference

The program provides exact Fisher's and mid-P tests, the Mantel-Haenszel test, and Walter's test for binary data (with and without a continuity correction).

If *stratified data* are entered, the Walter's tests in the separate strata (continuity-corrected) are combined by averaging their *z* values (Stouffer *et al.* 1949: 45; DeMets 1987) and computing an overall P that controls for the stratifying variables. P-values are computed in three ways, weighting the strata by different methods: weighting them equally, by sample sizes (the number of pairs), and by the square roots of the sample sizes. In addition, a test is done for the *heterogeneity* of the P-values in the strata (Wolf 1986: 45).

Odds ratio

Maximum-likelihood and Mantel-Haenszel estimates of the odds ratio are computed, with exact (Fisher's and mid-P) and approximate confidence intervals. In occasional extreme instances, computational problems prevent the use of exact methods for the calculation of confidence intervals for the odds ratio. Approximate confidence intervals for the maximum-likelihood estimate of the odds ratio are shown only if exact intervals are not computed.

Jewell's low-bias estimator of the odds ratio (Jewell 1984) is also displayed. This serves to draw attention to the tendency for the odds ratio in a sample, especially a small one, to

overestimate the true odds ratio in the population represented. A disadvantage of the estimator is that it is affected by the direction of computation; its value when the number of case: "yes", control: "no" pairs is the numerator of the ratio is not the reciprocal of its value when this number is the denominator.

Kappa

The program computes *kappa*, which expresses the agreement among all the observations in the matched sets, and may serve to express the effectiveness of the matching procedure, since it indicates the extent to which the findings in matched pairs are more similar than findings in individuals from different pairs.

The probability of chance agreement is taken into account in the calculation of *kappa*. A value of 1 indicates perfect agreement (after allowing for this probability of chance agreement) between ratings; 0 indicates no agreement other than what can be attributed to chance, and a negative value indicates less than chance agreement. Fleiss (1981: 218) suggests that a value of 0.75 or more indicates excellent agreement, and 0.40 or less indicates poor agreement. Alternative guidelines are: over 0.80, very good agreement; 0.61-0.80, good; 0.41-0.60, moderate; 0.21-0.40, fair; and 0.20 or less, poor agreement (Altman 1991).

If stratified data are entered, an *overall kappa* (weighted by sample sizes) is computed.

METHODS

The program can cater for up to 20 controls per case.

Tests for the difference

The computation of *exact probabilities* uses an efficient algorithm for calculating the coefficients of the conditional distribution (Martin and Austin 1991, 1996), using code from David O. Martin's public-domain EXACTBB program.

The *Mantel-Haenszel* chi-square test for matched observations is described by Rothman (1986: 262-263: formulae 13-15 and 13-18).

The formula for *Walter's test* for binary data is formula 2 in Walter (1980); for a continuity-corrected test, 0.5 is subtracted from the absolute value of the numerator.

If *stratified data* are entered, the Walter's tests in the separate strata (continuity-corrected) are combined by averaging their *z* values (Stouffer *et al*. 1949, p. 45; DeMets 1987). Three different sets of weights are used for this purpose – weighting the test results equally, by the sample sizes in the strata, and by the square roots of the sample sizes. In addition, the heterogeneity of the P-values in the strata is tested, using the formula (Wolf 1986: 45):

$$\text{chi-square } (k - 1 \text{ d.f.}) = \sum(Zi - \text{MeanZ})^2$$

where k = number of strata,
Z_i = z value in stratum i
MeanZ = mean z value.

Odds ratio

Rothman (1986) explains the computation of maximum-likelihood (pp 254-255, 257-258) and Mantel-Haenszel point estimates (pp 256, 258: formulae 13-7 and 13-9) of the odds ratio and their approximate confidence intervals (pp 268-270: formulae 13-37 and 13-38, and pp 273-275). The computation of exact intervals uses an

efficient algorithm for calculating the coefficients of the conditional distribution (Martin and Austin 1991, 1996), using code from David O. Martin's public-domain EXACTBB program.

The *low-bias estimator of the odds ratio* is computed by Jewell's formula (Jewell 1984: 431), whether the number of controls per case is fixed or variable. If there is one control per case the estimator is

$b/(c + 1)$,

where b = number of “case Yes, control No” pairs
c = number of “case No, control Yes” pairs.

Kappa

Kappa is calculated by formulae 13.10 to 13.12 of Fleiss (1981). To test the null hypothesis by dividing kappa by its standard error, the standard error (for an underlying zero value of kappa) is calculated by formula 13.13. The hypothesis that agreement is better than chance is tested by formula 13.14 or 13.35.

If *stratified data* are entered, an *overall kappa* (weighted by sample sizes) is computed.

F. COMPARISON OF THREE OR MORE MATCHED SAMPLES ("YES-NO" VARIABLE)

This module compares the findings in 3 to 10 related samples (each observation being matched with an observation in each other sample) where the dependent variable is dichotomous ("yes-no"). The data may be sets of observations in matched individuals, or separate sets of observations in the same individuals. The 3 to10 samples can, but need not, lie in an ordered sequence (e.g. in a trial comparing different doses).

The program may be used, for example, to analyse a clinical trial in which matched subjects receive 3 to10 different treatments, or one in which each subject receives 3 to10 different treatments, or one in which each subject receives the same treatment under 3 to10 different conditions, or an observational study comparing matched subjects who have different degrees of exposure to a risk, or are measured under different defined circumstances, or are appraised by different clinicians or interviewed by different interviewers, or a study in which the same individuals are observed under different specified conditions, or at various specified times, or are asked different specified questions.

If the samples lie in an ordered sequence, they should be numbered accordingly. If there is a reference group, it should be entered as sample 1. The pattern of findings in the members of the matched set must be entered, using 0 for "no" and 1 for "yes" (e.g., "0" for the observation in sample 1, "1" for the matched observation in sample 2, "0" for the matched observation in sample 3, etc. Matched sets can be entered individually, or sets with the same pattern of findings can be entered together, with their frequency.

If the data are stratified, enter each stratum in turn. Click on "All strata" whenever combined results are required.

The program provides **tests comparing the matched samples** (Cochran's *Q* test, Page's test for trend, and multiple-comparison tests), **odds ratios**, and ***kappa***.

If *stratified data* are entered, the Cochran Q tests and Page tests in the separate strata are combined and the *heterogeneity* of the P-values in the strata is tested.

Tests comparing the matched samples

Cochran's Q test, which is an extension of the McNemar test for matched pairs, tests the null hypothesis that the probability of a "yes" result is the same in each sample, against the alternative that the relative probabilities in the different samples are consistent for all sets of related observations; that is, if in one set the probability of "yes" is larger in sample 1 than in sample 2, this is so in all sets. With three samples or small numbers (Tate and Brown 1970) a P-value that is near a borderline of significance should be treated with caution; the program provides a warning.

Page's test is appropriate if the samples fall into an ordered sequence. It is a test for the presence of a monotonic trend (Siegel and Castellan 1988: 184-188). The test is conservative when applied to dichotomous data, because of the large number of ties (Hollander and Wolfe 1999, p. 292).

A *multiple-comparisons* procedure is used to compare the percentage of "yes" observations in sample 1 (taken as the reference group) with the percentage in each other sample.

If *stratified data* are entered, the Cochran Q tests and Page tests in the separate strata are combined by Stouffer's method (Stouffer *et al*. 1949, p. 45; DeMets 1987) to produce overall tests that control for the stratifying variables. Three different sets of weights are used for this purpose – weighting the test results equally, by the sample sizes in the strata, and by the square roots of the sample sizes. In addition, the *heterogeneity* of the P-values in the strata is tested.

Odds ratios

Odds ratios comparing each possible pair of samples are calculated.

Kappa

The program computes *kappa*, which expresses the agreement among all the observations in the matched sets, and may serve to express the effectiveness of the matching procedure, since it indicates the extent to which the findings in matched pairs are more similar than findings in individuals from different pairs (Fleiss 1981: 233).

The probability of chance agreement is taken into account in the calculation of *kappa*. A value of 1 indicates perfect agreement (after allowing for this probability of chance agreement) between ratings; 0 indicates no agreement other than what can be attributed to chance, and a negative value indicates less than chance agreement. Fleiss (1981: 218) suggests that a value of 0.75 or more indicates excellent agreement, and 0.40 or less indicates poor agreement. Alternative guidelines are: over 0.80, very good agreement; 0.61-0.80, good; 0.41-0.60, moderate; 0.21-0.40, fair; and 0.20 or less, poor agreement (Altman 1991).

METHODS

Tests comparing the matched samples

Cochran's Q test is described by Siegel and Castellan (1986: 170-174), Daniel (1978: 241-244) and Zar (1998: 268-270).

Page's test is described by Siegel and Castellan (1988: 184-188). A large-sample approximation (formula 7.10) is used, since the available tables of critical values for small numbers are inappropriate in the presence of many ties (Hollander and Wolfe 1999: 291-292.

The *multiple- comparison* tests use Zar's formulae 12.61 and 12.62

If *stratified data* are entered, the Cochran *Q* tests in the separate strata are combined by averaging their *z* values (Stouffer *et al*. 1949, p. 45; DeMets 1987). Three different sets of weights are used for this purpose – weighting the test results equally, by the sample sizes in the strata, and by the square roots of the sample sizes. In addition, the heterogeneity of the P-values in the strata is tested, using the formula (Wolf 1986: 45):

$$\text{chi-square } (k - 1 \text{ d.f.}) = \sum(Zi - \text{MeanZ})^2$$

where k = number of strata,
Z_i = z value in stratum i
MeanZ = mean z value.

The Page tests for trend are combined in the same way, but using the signed *z* values provided by the tests, and without excluding sets that exhibit no differences between their members. The Page tests are not combined if there are 12 or fewer sets in any stratum, or 21 or fewer sets if the dependent variable has 3 categories.

Odds ratios

For each pair of samples, the odds ratio is the number of matched pairs that have “yes” for the first sample and “no” for the second, divided by the number with “no” for the first sample and ”yes” for the second.

Kappa

Kappa and its standard error are calculated by formulae 13.50 and 13.53 of Fleiss (1981).

G. COMPUTE *KAPPA* FOR 3 OR MORE RATINGS

This module appraises the agreement between a fixed number (3 or more) of matched observations with respect to a variable with 2-10 categories. It might be used to measure the agreement between 3 or more ratings of the same individuals, e.g. by different observers or tests, or between ratings made by the same observer on different occasions.

The numbers of ratings (k = 3 or more) and the number of categories (2-10) must be entered. The findings in the set of ratings are then entered, by entering the number of ratings falling into each category (these should add up to k). Each set of ratings can be entered separately, , or sets with the same pattern of findings can be entered together, with their frequency.

The program provides the overall ***kappa***, and *kappa* values for individual categories.

If stratified data are entered, an overall value of *kappa* is computed.

Kappa

The overall *kappa* is computed, with its standard error and significance. *Kappa* values are also reported for individual categories, with their significance; but these test results should be treated with caution, since they are not based on a multiple-comparison procedure.

For *stratified data* are entered, an overall value of *kappa*, weighted by sample size, is computed.

The probability of chance agreement is taken into account in the calculation of *kappa*. A value of 1 indicates perfect agreement (after allowing for this probability of chance agreement) between ratings; 0 indicates no agreement other than what can be attributed to chance, and a negative value indicates less than chance agreement. Fleiss (1981: 218) suggests that a value of 0.75 or more indicates excellent agreement, and 0.40 or less indicates poor agreement. Alternative guidelines are: over 0.80, very good agreement; 0.61-0.80, good; 0.41-0.60, moderate; 0.21-0.40, fair; and 0.20 or less, poor agreement (Altman 1991).

METHODS

Kappa

Kappa and its standard error are calculated by formulae 13.50 and 13.53 of Fleiss (1981).

H. COMPARISON OF TWO GROUPS OR TWO MEASURES (FIXED NUMBER OF MATCHED NUMERICAL OBSERVATIONS)

This module compares two sets (designated "cases" and "controls") of matched numerical observations. It can be used to compare two groups – index subjects with matched controls in a case-control study, cohort study, or trial – or two measurement methods.

For a comparison of groups, each matched set must contain between 3 and 11 observations in all., comprising a fixed number of "cases" (1 to 5) and a fixed number of "controls" (1 to 10). The matched sets of observations must be entered individually, after entering the numbers of cases and controls per set. Up to 500 sets may be entered.

For a comparison of measurement methods (A and B), equal-sized sets of replicate measurements by the two methods are required (2 to5 by each method). The two methods may be applied to the same subjects or to different subjects. The program terms the measurements by method A as "cases", and those by method B as "controls". After entering the numbers of "cases" and "controls" per set (numbers which must be identical), the measurements of each subject by method A must be entered, in a separate line, followed (in the same line) by the measurements (of the same or a different subject) using method B.

The results relevant to a comparison of groups are three **tests** (Rosner's and Walter's tests and a paired *t*-test) for the difference between the mean values, approximate confidence intervals for the **difference between the mean values**, and **between-sets and within-sets variances**.

The results relevant to a comparison of measurements include a **95% repeatability coefficient** and **ANOVA table** for each method; the **95% limits of agreement** between the methods and the **relationship between the difference and the mean value** (appropriate if the two methods were applied to the same subjects); and **F-tests** for the difference between the methods, for the effect of repeated measurements, and for interaction, and a repeated-measures **ANOVA tables value** (appropriate if the two methods were applied to different subjects).

Tests

Rosner's test is a generalization of the paired *t*-test that takes account of within-sets and between-sets variability (Rosner 1982). If single index subjects are compared with controls, it appraises the significance of the differences between their values. If two groups of observations are compared, it appraises the difference between the mean values in the two groups. Two P-values may be displayed. If so, these may be regarded as the bounds of the true P-value. The true P-value depends on the relative magnitude of the within-sets and between-sets variabilities (see below), as explained by an on-screen message. The test sometimes presents technical difficulties, and is omitted.

Walter's test (Walter 1980) tests the significance of the mean case-control difference weighted by the numbers of cases and controls in the set. Rosner (1982) points out that (unlike his test) Walter's test assumes zero between-sets variability, and may therefore provide a misleadingly low P-value if there is much between-sets variability.

The *paired t-test* tests the significance of the unweighted mean difference between the case and control means within each matched set. Rosner (1982) points out that (unlike his test) the paired *t* test assumes zero within-sets variability, and may therefore provide a misleadingly low P-value if there is much within-sets variability.

If the numbers of cases and controls (assumed to be the numbers of replications by two methods of measurement) are equal, *F-tests* are performed for the difference between the two methods, for differences between repeated measurements, and for interaction - i.e. for a difference between the methods in the uniformity (reliability) of repeated measurements. Each of the latter two tests is done three times - without adjustment, and with two adjustments. The adjusted tests are Fleiss's "Approximation 3", which is not appropriate in all situations, and his "Approximation 4", which is valid in all situations but may be extremely conservative (Fleiss 1985: 227). The F-tests are appropriate only if the two methods of measurement were applied to different subjects.

Difference between the mean values

The program displays the mean case-control difference and its standard error, computed separately by the Rosner and Walter procedures and for unweighted data, with approximate 90%, 95%, and 99% confidence intervals.

Between-sets and within-sets variances

The between-sets variance computed by Rosner's procedure (Rosner 1982) is reported. This represents the variation between matched sets, and the within-sets variance represents the variation within either the case or the control group for a specific matched set. The ratio of the two variances is an indication of the value of multiple matching. If the between-sets variance is much larger than the within-sets variance, multiple matching brings little benefit (Rosner 1982; Lee and Wilkens 1994).

95% repeatability coefficient

If the numbers of cases and controls (assumed to be the numbers of replications by two methods of measurement) are equal, the 95% repeatability coefficient is computed for each method. This expresses the expectation (with 95% confidence) of the maximum size of the absolute difference between two observations using the same method.

95% limits of agreement

If the numbers of cases and controls (assumed to be the numbers of replications by two methods of measurement) are equal, the 95% limits of agreement are computed. These (which are appropriate only if the two methods of measurement were applied to the same subjects) answer the question, "given a measurement by one method, how far might this be from a measurement by the other method?" They demarcate the bounds of the range that, with a 95% probability, includes the difference between single measurements of the same

subject by the two methods. The 95% confidence intervals of the limits of agreement are estimated (the limits of agreement may be very imprecise if the sample is small).

Use of the 95% limits of agreement assumes that the differences are reasonably constant throughout the range of measurement. To check this assumption, the program displays *Spearman's coefficient of correlation between the difference and the mean level* (also appropriate only if the two methods of measurement were applied to the same subjects). The correlation coefficient may be expected to be zero if the mean difference does not change with increasing values. Even when one of the methods of measurement is a new one and the other is an accepted standard, it is preferable to examine the relationship between the difference and the mean value rather than the relationship between the difference and the standard measurement, which (as shown by Bland and Altman 1995b) is likely to be misleading.

ANOVA tables

If the numbers of cases and controls (assumed to be the numbers of replications by two methods of measurement) are equal, a one-way ANOVA table is displayed for each method, showing the between-subjects and within-subjects components of variance, as well as a repeated-measurement ANOVA for the combined data (Fleiss 1986: 220-228), which is appropriate only if the two methods of measurement were applied to different subjects.
.

METHODS

The two groups of observations are referred to as "cases" and "controls".

Tests

Rosner's test (Rosner 1982) is a generalization of the paired *t*-test that takes account of within-sets and between-sets variability. It adjusts and appraises the significance of the mean within-set difference. The test sometimes presents technical difficulties, since it requires the computation of maximum-likelihood estimates by an iterative procedure that may fail to find an appropriate (positive) root. If this difficulty is encountered (usually because of marked within-set variability) an appropriate message is displayed.

In Rosner's procedure the within-pairing variability is calculated by Rosner's formula 2.2 (Rosner 1982), and maximum likelihood estimates of the between-pairing variability and the adjusted mean case-control difference are then computed by an iterative process, using the van Wijnsgaarden-Dekker-Brent root-solver (Press *et al.* 1989: 283-286). The adjustment takes account of the numbers of cases and controls per set, using their reciprocals. Significance is appraised by Rosner's formula 2.3, using alternative degrees of freedom when referring the test statistic (*lambda*) to the *t*-distribution, namely $N - 2R$ and $R - 1$ (where N = number of subjects and R = number of matched sets). This provides two P-values (both of which are shown if they differ appreciably), which may be regarded as the bounds of the true P-value. The true value depends on the relative magnitude of the within-sets and between-sets variabilities.

Walter's test uses formula 2.4 of Rosner (1982). This permits application of the test to situations where there are matched sets with two or more cases.

The *paired t-test* is calculated by the usual formula (see, e.g. Selvin 1991: 65, formula 2.51), except that in each matched set the two values (of case and control) are replaced by the means (of cases, if there is more than one case, and of controls, if there is more than one control).

The *F-tests* are based on a repeated-measurement ANOVA (see Fleiss 1986: 220-228). The adjustments, which Fleiss calls Approximations 3 and 4, involve changes to the degrees of freedom (Fleiss 1986: 227:

formulae 8.9 and 8,10); the changed degrees of freedom are rounded off to the nearest whole number. This ANOVA is not done if the number of measurements varies for different subjects.

Difference between the mean values

In Rosner's procedure (see above), the adjusted mean case-control difference is computed by weighting the difference in each matched set by

$$1 / \{B + W.[(1 / N1) + (1 / N2)]\}$$

where

B = between-sets variance
W = within-sets variance
N1 and N2 = numbers of cases and controls in the set.

In Walter's procedure, the difference in each matched set is weighted by

$$1 / [(1 / N1) + (1 / N2)]$$

95% repeatability coefficient

The computation of the coefficient of repeatability is explained by Bland and Altman (1999: 149).

95% limits of agreement

The 95% limits of agreement and their confidence intervals are computed by the method explained by Bland and Altman (1999; section 5.1: formulae 5.3 and 5.10), using within-subject mean squares based on one-way analyses of variance for the two methods (Guilford and Fruchter 1981: 234-5: formulae 13.15 and 13.16).

I. COMPARISON OF 3 TO 10 SAMPLES OR REPLICATES (FIXED NUMBER OF MATCHED NUMERICAL OBSERVATIONS)

This module compares the findings in 3 to 10 related samples (each observation being matched with an observation in each other sample) where the dependent variable is numerical (ordinal or interval-scale). The data may be sets of observations in matched individuals, or separate sets of observations in the same individuals, such as replicated measurements. The 3 to10 samples can, but need not, lie in an ordered sequence (e.g. in a trial comparing different doses).

The program may be used, for example, to analyse a clinical trial in which matched subjects receive 3 to 10 different treatments, or one in which each subject receives 3 to 10 different treatments, or the same treatment under 3 to 10 different conditions, or an observational study comparing matched subjects who have different degrees of exposure to a risk factor or are measured under different defined conditions or appraised by different clinicians or interviewed by different interviewers, or a study in which the same individuals are observed under different specified conditions or at various specified times, or are asked different specified questions. If the samples lie in an ordered sequence, they should be numbered accordingly. If there is a reference group, it should be entered as sample 1. In reliability studies, the program compares replicate measurements; replicates may be entered in any order, unless they represent defined instruments, observers, times, conditions, etc.

The actual observations in the members of the matched set may be entered, or (optionally) their ranks can be entered, e.g. 1 3 2 instead of 6.1 11 9; or (for ties) 1 4 2.5 2.5 instead of 6.1 11 9 9 (giving tied observations the mean of the ranks they would have if they differed slightly). Matched sets can be entered individually, or sets with the same findings can be entered together. Up to 500 sets or patterns may be entered.

For stratified data, enter each stratum in turn, and click on “All strata” for combined results.

The program provides **tests (***Friedman's two-way analysis of variance by ranks***,** *Page's test for a monotonic trend,* an *F-test* [with the ANOVA table on which it is based], and *multiple comparisons*) and **measures of agreement** (*Kendall's concordance coefficient, Spearman's correlation coefficient,* six *intraclass correlation coefficients, repeatability coefficient, Spearman-Brown coefficients of reliability).* Some of the procedures are *nonparametric,* and are applicable to all numerical data Others (*F*-test, intraclass correlation coefficient, repeatability coefficient, Spearman-Brown coefficients of reliability, some of the multiple comparisons) are *parametric,* and are applicable only to interval-scale data with an assumed normal distribution*. The parametric procedures are not appropriate if ranks are entered.

If *stratified data* are entered, the Friedman and Page tests in the separate strata are combined and the *heterogeneity* of the P-values in the strata is tested.

* [As pointed out by Altman (1991: 330), it may not be the raw data, but the residual values (after allowing for the effects of sample membership and matched-set membership), that should be normally distributed.]

Tests

Friedman's two-way analysis of variance by ranks (Siegel and Castellan 1986: 174-183; Zar 1998: 263-267) is applicable to all numerical data. It tests the null hypothesis that the values in the different samples represent the same population median, against the alternative that at least two of the samples have different medians.

Page's test for a monotonic trend (Siegel and Castellan 1988: 184-188) is applicable to all numerical data. For the test to be meaningful, the samples should be entered in the sequence to be tested. The test might, for example, be a way of appraising the dose-response relationship in a trial in which different doses are given to different matched samples or to the same individuals at different times.

The *F test*, which is appropriate for interval-scale data with an assumed normal distribution, test the null hypothesis that there is no difference among the mean values of the various samples. It is based on a two-factor (repeated-measure) analysis of variance.

The *multiple-comparison tests* include comparisons of each sample with sample 1 (assumed to be the reference group), and comparisons of each sample with each other sample. Both nonparametric tests (applicable to all numerical data) and parametric tests are performed. Nonparametric pairwise comparisons are done only if the Friedman test reveals a significant difference ($P < 0.05$) between samples; the median of each set of matched observations is displayed, with (if the number of observations is at least 10) the interquartile range. Parametric multiple-comparison tests (Zar 1998, 210-214, 217-218) are done only if the F test is significant ($P < 0.05$); they use the Dunnett procedure to compare the mean of sample 1 with all other sample means. and the Tukey procedure or "honestly significant difference test" for pairwise comparisons; the mean of each matched set of observations is displayed.

If *stratified data* are entered, the results of the Friedman analyses of variance in the separate strata are combined by Stouffer's method (Stouffer *et al.* 1949, p. 45; DeMets 1987) to produce overall P-values that control for the stratifying variables. Three different sets of weights are used for this purpose – weighting the test results equally, by the sample sizes in the strata, and by the square roots of the sample sizes. In addition, the *heterogeneity* of the P-values in the strata is tested.

Measures of agreement

Kendall's coefficient of concordance (which varies between 0 and 1) is based on the ranks of the observations within each related set, and expresses the degree of similarity of their ranking in different samples.

The *average Spearman's coefficient of rank correlation* between all possible pairs of rankings can vary from $-1 / (k - 1)$ to 1, where k is the number of matched observations in a set.

Intraclass correlation coefficients, which are appropriate for interval-scale data with an assumed normal distribution, are measures of agreement that express the correlation (in terms of absolute agreement) between measurements within individuals or sets of matched individuals. Six intraclass correlation coefficient (ICC) values are computed (Shrout and Fleiss 1979), with their 95% confidence intervals.

Each ICC is appropriate in a different situation. (a) The values with the rubric "two-way model with fixed raters" are appropriate in studies where the matched observations in each set represent various "unique" raters, and no inferences are made about other raters; "raters" denote the various observers, treatments, methods or conditions of observation, matched individuals, or (in a reliability study of a questionnaire or other scale) questions or other scale items, that were studied. Two such ICCs are provided. The first, which Shrout and Fleiss refer to as model 3.1, uses a single measurement as the unit of analysis, and the second (model 3,k) uses an average measurement. (b) The two ICC values reported as "two-way model with random raters" are appropriate if the raters were randomly selected from a larger population of raters and it is proposed to generalize the findings to this larger population. If analysis is based on a single measurement, this is model 2,1; if it based on an average measurement, it is model 2,k. (c) The third pair of ICC values, entitled "one-way random model", is appropriate in methodological or other studies where the measurements are replications by the same observer or using the same instrument, and the order in which they are entered does not matter (this does not apply to the other ICC values).. They apply to the use of a single measurement (model 1,1) – e.g. in studies to determine the reliability of a single measurement – or to an average measurement (model 1,k) – e.g. in studies to determine the reliability of an average measurement.

The maximum value of an ICC is 1; the lower limit is an indeterminate negative value. As a rule of thumb, it has been suggested that ICC values above 0.75 should be regarded as evidence of excellent, and values above 0.4 as evidence of good, reliability (Shoukri and Pause1998: 27).

In the appraisal of replicated measurements a low ICC may express variability of the characteristic measured, as well as low reliability of measurement; this is especially important if measurements were conducted at different times.

The *coefficient of repeatability* is applicable if replicate measurements were entered, and is appropriate for interval-scale data with an assumed normal distribution. It expresses the expectation (with 95% confidence) for the maximum size of the absolute difference between a pair of observations, assuming that repeatability is similar at all magnitudes. Approximate confidence intervals are estimated for the coefficient.

Spearman-Brown coefficients of reliability provide estimates of the effect of using the means of replicated observations. They predict what the reliability would be if two, three, four, or five replications were averaged.

METHODS

Tests

In Friedman's two-way analysis of variance by ranks (Siegel and Castellan 1986: 174-183; Zar 1998: 263-267), the program uses criteria for $P < 0.05$, 0.01, and 0.001 listed by Zar (1998: Table B.14) if there are 3 or 4 samples with less than 16 values in each, or 5 or 6 samples with less than 11 values in each. Otherwise significance is appraised by use of the Friedman statistic, which has an approximately chi-square distribution unless numbers are small, and also by using Iman and Davenport's *F* (Iman and Davenport 1980), which is generally more powerful (Zar 1998: 264). The formula for Iman and Davenport's *F* is provided by Sprent (1993: 145) and Zar (1998: formula 12.47), with $N - 1$ and $(k - 1)(N - 1)$ degrees of freedom. If the rankings in

the sets are identical, *F* has a value of infinity, and

$$P = (1 / N)(k - 1),$$

where N = number of samples
k = number of sets.

In *Page's trend test* (Siegel and Castellan 1988: 184-188), Page's statistic *L* is calculated by formula 7.7, and *Z* by formula 7.10. A one-tailed P-value is computed, based on the normal distribution; if numbers are small, however (3 groups with less than 21 observations in each, or 4-10 groups with less than 13), the Page statistic *L* is compared with critical values for P < 0.05, 0.01, and 0.001 (Siegel and Castellan 1988: 354-355, Table N).

The *F test* is based on a two-factor (repeated-measure) analysis of variance (Zar 1998, 255-260).

The *non parametric multiple comparison tests* are described by Siegel and Castellan (1988: 180-183); the comparisons with sample 1) use critical values for P < 0.05 and 0.01 derived from Siegel and Castellan (1988: 321, Table Aiii), and the pairwise comparisons use critical levels for P < 0.05, 0.01, and 0.001.

The *parametric multiple comparison tests* use the Dunnett procedure (Zar 1998, 217-218) to compare the mean of sample 1 with all other sample means; and the Tukey procedure (Zar 1998, 210-214) for pairwise comparisons. The results are appraised in relation to critical values of the *Q* distribution (Zar 1998, Tables B6 and B7), and are reported as P < 0.01, < 0.05, or not significant.

If *stratified data* are entered, the results of the Friedman analyses of variance in the separate strata are combined by averaging their *z* values (Stouffer *et al.* 1949, p. 45; DeMets 1987). Three different sets of weights are used for this purpose – weighting the test results equally, by the sample sizes in the strata, and by the square roots of the sample sizes. In addition, the heterogeneity of the P-values in the strata is tested, using the formula (Wolf 1986: 45):

$$\text{chi-square } (k - 1 \text{ d.f.}) = \sum(Zi - \text{MeanZ})^2$$

where k = number of strata,
Z_i = z value in stratum i
MeanZ = mean z value.

The Page tests for trend are combined in the same way, but using the signed *z* values provided by the tests, and without excluding sets that exhibit no differences between their members. The Page tests are not combined if there are 12 or fewer sets in any stratum, or 21 or fewer sets if the dependent variable has 3 categories.

Measures of agreement

Kendall's coefficient of concordance is derived from the Friedman statistic by formula 12.51 of Zar (1998). Its significance is tested by computing chi-square (using formula 9.19 of Siegel and Castellan 1988: 269), unless there are under 21 matched sets and under 8 samples, when use is made of the critical values in Table T of Siegel and Castellan 1988: 365).

The *average Spearman's coefficient of rank correlation* between all possible pairs of rankings is derived from Kendall's coefficient of concordance (Siegel and Castellan 1988: 362).

The following formulae (Shrout and Fleiss 1979) are used for the six intraclass correlation coefficients. Shrout-Fleiss ICC models 1,1 and 1,k are computed from a one-way random effects model ANOVA, models 2,1 and 2,k from a two-way random effects model ANOVA, and models 3,1 and 3,k from a two-way mixed effects model ANOVA.

ICC model 1,1 = (MSB – MSW) / [MSB + (k – 1)MSW]
ICC model 1,k = (MSB – MSW) / MSB
ICC model 2,1 = (MSB – MSE) / [MSB + (k – 1) MSE + k(MSJ – MSE) / N]
ICC model 2,k = (MSB – MSE) / [MSB + (MSJ – MSE) / N]
ICC model 3,1 = (MSB - MSE) / [MSB + (k - 1)MSE]
ICC model 3,k = (MSB – MSE) / MSB

where MSB = between-subjects mean square
MSE = residual within-subjects mean square
MSW = within-subjects mean square
N = number of subjects
k = number of observations in matched set

Formulae for confidence intervals for the six ICC models are provided by McGraw and Wong (1996) in their Table 7, where they are referred to as ICC(1) and ICC(k) for Case 1, and ICC(A,1) and ICC(A,k) for Cases 2 and 3, The formulae (except those for models 2,1 and 2,k) are set out in a convenient code by Steinley and Wood (2000). Linear interpolation is used to estimate F values that are based on non-integer degrees of freedom (and 1 d.f. is substituted for <1 d.f.) in the computation of confidence intervals for models 2,1 and 2,k; the latter results may differ slightly from those provided by SPSS, which handles non-integer degrees of freedom differently.

The *Spearman-Brown prediction formula* (Fleiss 198: 14-15: formula 1.3) for reliability (*R)* is

$$R = Nr / [1 + (N - 1)r]$$

where N = number of replicates that are averaged
r = intraclass correlation coefficient (model 1,1)

This application of the Spearman-Brown formula was suggested by its use by Solomon (2004).

Fleiss's formula 1.31 is used to estimate the number of replicates required to obtain a reliability of 0.75 or 0.8:

$$N = P(1 - r) / [r(1 - P)]$$

where P = 0.75 or 0.8

The computation of the *coefficient of repeatability* is explained by Bland and Altman (1999: 149). Approximate confidence intervals are obtained by substituting confidence limits for the within-samples variance, estimated by the method described by Zar (1998: formula 7.16), in the formulae.

J. COMPARISON OF SUBJECTS WITH VARYING NUMBERS OF MATCHED CONTROLS (“YES-NO” VARIABLE)

This module is appropriate for the analysis of case-control studies, clinical trials and cohort studies in which each index subject (each case, experimental subject, or individual exposed to a risk or protective factor) has a variable number (1-20) of individually matched controls, and the dependent variable is dichotomous (“yes-no”), e.g. “yes” = exposure to a risk factor (in a case-control study), the success of a treatment, or the presence of a disease (in a cohort study). It compares the findings in the index subjects and their matched controls.

The program refers to index subjects as “cases”. Each set of matched observations can be entered in a separate line, or sets with the same findings can be entered together, with their frequency. The required entries for each pattern of findings are 0 (“no”) or 1 (“yes”) for the “case”, the number of matched controls with “yes”, and the number of matched controls with “no”.

If the data are stratified, enter each stratum in turn. Click on “All strata” whenever combined results are required.

The program provides **tests (**Mantel-Haenszel test, Walter's test for binary data), the **odds ratio** (Mantel-Haenszel estimates, low-bias estimate), and **kappa**.

If *stratified data* are entered, an overall Mantel-Haenszel test is done, the results of the Walter’s tests in the separate strata are combined, the *heterogeneity* of the P-values in the strata is tested, and an overall *kappa* is computed.

Tests

The program performs a Mantel-Haenszel test (without a continuity correction) and Walter's test for binary data (with and without a continuity correction).

If *stratified data* are entered, an overall Mantel-Haenszel test is done, and the Walter's tests in the separate strata (continuity-corrected) are combined by averaging their *z* values (Stouffer *et al*. 1949: 45; DeMets 1987) and computing an overall P that controls for the stratifying variables. P-values are computed in three ways, weighting the strata by different methods: weighting them equally, by sample sizes (the number of pairs), and by the square roots of the sample sizes. In addition, a test is done for the *heterogeneity* of the P-values in the strata (Wolf 1986: 45).

Odds ratio

Mantel-Haenszel estimates of the odds ratio and its 90%, 95%, and 99% confidence intervals.are computed. and Jewell's low-bias estimator of the odds ratio (Jewell 1984) is shown.

Kappa

.

The program computes *kappa*, which expresses the agreement among all the observations in the matched sets, and may serve to express the effectiveness of the matching procedure, since it indicates the extent to which the findings in matched pairs are more similar than findings in individuals from different pairs (Fleiss 1981: 233).

The probability of chance agreement is taken into account in the calculation of *kappa*. A value of 1 indicates perfect agreement (after allowing for this probability of chance agreement) between ratings; 0 indicates no agreement other than what can be attributed to chance, and a negative value indicates less than chance agreement. Fleiss (1981: 218) suggests that a value of 0.75 or more indicates excellent agreement, and 0.40 or less indicates poor agreement. Alternative guidelines are: over 0.80, very good agreement; 0.61-0.80, good; 0.41-0.60, moderate; 0.21-0.40, fair; and 0.20 or less, poor agreement (Altman 1991).

METHODS

Tests

The Mantel-Haenszel test uses formula 13-18 of Rothman (1986). If stratified data are entered,

$$\text{chi-square} = (\sum \text{Num}_i)^2 / \sum(\text{Den}_i^2)$$

where Num_i = numerator of Rothman's formula in stratum *i*
Den_i = denominator of Rothman's formula in stratum *i*

The formula for *Walter's test* for binary data is formula 2 in Walter (1980); for a continuity-corrected test, 0.5 is subtracted from the absolute value of the numerator. If *stratified data* are entered, the Walter's tests in the separate strata (continuity-corrected) are combined by averaging their *z* values (Stouffer *et al.* 1949: 45; DeMets 1987). Three different sets of weights are used for this purpose – weighting the test results equally, by the sample sizes in the strata, and by the square roots of the sample sizes. In addition, the heterogeneity of the P-values in the strata is tested, using the formula (Wolf 1986: 45):

$$\text{chi-square } (k - 1 \text{ d.f.}) = \sum(Zi - \text{MeanZ})^2$$

where k = number of strata,
Z_i = z value in stratum i
MeanZ = mean z value.

Odds ratio

The Manterl-Haenszel estimate of the odds ratio is computed by formula 13-9 of Rothman (1986), and its confidence intervals by the procedure described on page 274 of Rothman (1986).

Kappa

Kappa and its standard error are calculated by formulae 13.44 and 13.46 of Fleiss (1981).

K. COMPUTE *KAPPA* FOR A VARIABLE NUMBER OF RATINGS

This module appraises the agreement between a variable number (3 or more) of matched observations with respect to a "yes"-"no" (dichotomous) variable. It might be used to measure the agreement between ratings of the same individuals, e.g. by different observers or tests, or between ratings of the same individuals made by the same observer on different occasions.

The findings in the set of ratings are then entered, by entering the numbers of "yes" ratings and "no" ratings. Each set of ratings can be entered separately, or sets with the same pattern of findings can be entered together, with their frequency.

The program provides the overall ***kappa***, and *kappa* values for individual categories.

If stratified data are entered, an overall value of *kappa* is computed.

Kappa

The overall *kappa* is computed, with its standard error and significance. *Kappa* values are also reported for individual categories, with their significance; but these test results should be treated with caution, since they are not based on a multiple-comparison procedure.

For *stratified data* are entered, an overall value of *kappa*, weighted by sample size, is computed.

The probability of chance agreement is taken into account in the calculation of *kappa*. A value of 1 indicates perfect agreement (after allowing for this probability of chance agreement) between ratings; 0 indicates no agreement other than what can be attributed to chance, and a negative value indicates less than chance agreement. Fleiss (1981: 218) suggests that a value of 0.75 or more indicates excellent agreement, and 0.40 or less indicates poor agreement. Alternative guidelines are: over 0.80, very good agreement; 0.61-0.80, good; 0.41-0.60, moderate; 0.21-0.40, fair; and 0.20 or less, poor agreement (Altman 1991).

METHODS

Kappa

Kappa and its standard error are calculated by formulae 13.44 to 13.46 of Fleiss (1981).

L1. COMPARISON OF TWO GROUPS OF VARYING NUMBERS OF MATCHED NUMERICAL OBSERVATIONS

This module is appropriate for the analysis of case-control or cohort studies, trials, comparisons of methods of measurement, or other studies that compare two groups of matched numerical variables, where some or all of the matched sets have 3 or more observations, and the numbers of observations in the two groups (in each set) may vary. The program compares the two groups of observations.

The groups are arbitrarily referred to as “cases” and “controls”. Optionally, a fixed number can be specified for the cases in each matched set. A matched set may contain 2-9 observations (1-8 cases and 1-8 controls). Each set must be entered in a separate line: first the case or cases, , then a slash (/), then the control or controls, then another slash. For example, the entry for a set containing 1 case and 3 controls might be:

16.23 / 9.8 11.06 15.11 /

Up to 500 sets may be entered.

The program provides three **tests** (Rosner's and Walter's tests and a paired t-test) for the **difference between the mean values** of cases and controls, approximate confidence intervals for this difference, and **between-sets and within-sets variances**.

Tests

Rosner's test is a generalization of the paired *t*-test that takes account of within-sets and between-sets variability (Rosner 1982). Iit appraises the significance of the differences between the mean values in the two groups. Two P-values may be displayed. If so, these may be regarded as the bounds of the true P-value. The true P-value depends on the relative magnitude of the within-sets and between-sets variabilities (see below), as explained by an on-screen message. The test sometimes presents technical difficulties, and is omitted.

Walter's test (Walter 1980) tests the significance of the mean case-control difference weighted by the numbers of cases and controls in the set. Rosner (1982) points out that (unlike his test) Walter's test assumes zero between-sets variability, and may therefore provide a misleadingly low P-value if there is much between-sets variability.

The *paired t-test* tests the significance of the unweighted mean difference between the case and control means within each matched set. Rosner (1982) points out that (unlike his test) the paired *t* test assumes zero within-sets variability, and may therefore provide a misleadingly low P-value if there is much within-sets variability.

Difference between the mean values

The program displays the mean case-control difference and its standard error, computed separately by the Rosner and Walter procedures and for unweighted data, with approximate 90%, 95%, and 99% confidence intervals.

Between-sets and within-sets variances

The between-sets variance represents the variation between matched sets), and the within-sets variance represents the variation within either the case or the control group for a specific matched set. The ratio of the two variances is an indication of the value of multiple matching. If the between-sets variance is much larger than the within-sets variance, multiple matching brings little benefit (Rosner 1982; Lee and Wilkens 1994).

METHODS

Tests

Rosner's test (Rosner 1982) is a generalization of the paired *t*-test that takes account of within-sets and between-sets variability. It adjusts and appraises the significance of the mean within-set difference. The test sometimes presents technical difficulties, since it requires the computation of maximum-likelihood estimates by an iterative procedure that may fail to find an appropriate (positive) root. If this difficulty is encountered (usually because of marked within-set variability) an appropriate message is displayed.

In Rosner's procedure the within-pairing variability is calculated by Rosner's formula 2.2 (Rosner 1982), and maximum likelihood estimates of the between-pairing variability and the adjusted mean case-control difference are then computed by an iterative process, using the van Wijnsgaarden-Dekker-Brent root-solver (Press *et al.* 1989: 283-286). The adjustment takes account of the numbers of cases and controls per set, using their reciprocals. Significance is appraised by Rosner's formula 2.3, using alternative degrees of freedom when referring the test statistic (*lambda*) to the *t*-distribution, namely $N - 2R$ and $R - 1$ (where N = number of subjects and R = number of matched sets). This provides two P-values (both of which are shown if they differ appreciably), which may be regarded as the bounds of the true P-value. The true value depends on the relative magnitude of the within-sets and between-sets variabilities.

Walter's test uses formula 2.4 of Rosner (1982). This permits application of the test to situations where there are matched sets with two or more cases.

The *paired t-test* is calculated by the usual formula (see, e.g. Selvin 1991: 65, formula 2.51), except that in each matched set the two values (of case and control) are replaced by the means (of cases, if there is more than one case, and of controls, if there is more than one control).

Difference between the mean values

In Rosner's procedure (see above), the adjusted mean case-control difference is computed by weighting the difference in each matched set by

$$1 / \{B + W.[(1 / N1) + (1 / N2)]\}$$

where B = between-sets variance
W = within-sets variance
N1 and N2 = numbers of cases and controls in the set.

In Walter's procedure, the difference in each matched set is weighted by $1 / [(1 / N1) + (1 / N2)]$

Between-sets and within-sets variances

These variances are computed by Rosner's procedure (Rosner 1982).

L2. COMPARISON OF TWO METHODS OF MEASUREMENT, USING REPEATED NUMERICAL OBSERVATIONS

This module is appropriate in methodological studies that compare two methods of measuring a numerical variable by applying each method to each subject more than once. . The number of measurements per subject can vary, but for each subject there must be the same number (at least two) by each method.

The measurements of each subject are entered in a separate line.

The program provides the **mean difference** between measurements by the two methods, the **95% limits of agreement** between these measurements, the **relationship between the difference and the mean value**, and **ANOVA** tables.

Mean difference and 95% limits of agreement

The mean difference is the mean of the differences, for each subject, of the means of measurements by method A and measurements by method B.

The *95% limits of agreement* (Bland and Altman 1999) answer the question, "given a measurement by one method, how far might this be from a measurement by the other method?" They demarcate the bounds of the range that, with a 95% probability, includes the difference between single measurements of the same subject by the two methods.

Use of the 95% limits of agreement assumes that the differences are reasonably constant throughout the range of measurement. To check this assumption, the program displays *Spearman's coefficient of correlation between the difference and the mean level.* The correlation coefficient may be expected to be zero if the mean difference does not change with increasing values. Even when one of the methods of measurement is a new one and the other is an accepted standard, it is preferable to examine the relationship between the difference and the mean value rather than the relationship between the difference and the standard measurement, which (as shown by Bland and Altman 1995b) is likely to be misleading.

ANOVA tables

One-way ANOVA tables for each method show the between-subjects and within-subjects components of variance.

METHODS

The *95% limits of agreement* are computed by the method explained by Bland and Altman (1999; section 5.2), using within-subject mean squares based on one-way analyses of variance for the two methods (Guilford and Fruchter 1981: 234-5: formulae 13.15 and 13.16).

M. COMPARISON OF REPLICATE NUMERICAL MEASUREMENTS (VARYING NUMBERS)

This module appraises the agreement between matched numerical measurements, in a study where the numbers of matched measurements vary. It might be used to measure the agreement between replicate ratings of the same individuals by different observers or by the same observer on different occasions.

The measurements of each subject must be entered, in any order, on a separate line.

The program computes a **95% repeatability coefficient,** an **intraclass correlation coefficient** (with its 95% confidence interval) and **Spearman-Brown coefficients of reliability**, and estimates the number of replicates required to obtain a mean-rating ICC of 0.75 or 0.8.

95% repeatability coefficient

This coefficient expresses the expectation (with 95% confidence) for the maximum size of the absolute difference between a pair of observations, assuming that repeatability is similar at all magnitudes. Approximate 95% confidence intervals are estimated for the coefficient.

Intraclass correlation coefficient

The *intraclass correlation coefficient* (ICC), which is appropriate for interval-scale data with an assumed normal distribution, is a measure of agreement that expresses the correlation between measurements within individuals or sets of matched individuals. The program provides an estimate of the Shrout-Fleiss model 1,1 ICC (Shrout and Fleiss 1979), which is based on a "one-way random model"; the coefficient applies to the use of a single measurement. As a rule of thumb, it has been suggested that values above 0.75 indicate excellent, and values above 0.4 good, reliability (Shoukri and Pause1998: 27). Negative ICC values indicate that the within-subject variation is greater than the between-subject variation.

The program reports the effective average number of replicates, on which (if the numbers of replicates vary) the computations are based.

Spearman-Brown coefficients of reliability

Spearman-Brown coefficients of reliability provide estimates of the effect of using the means of replicated observations (Fleiss 1986: 14-15). They predict what the reliability would be if between 2 and 6 replications were averaged. The program also uses the formula in reverse, to estimate the number of replicates required to obtain a mean-rating ICC of 0.75 or 0.8.

METHODS

The computation of the *coefficient of repeatability* is explained by Bland and Altman (1999: 149). It is based on the within-sets variance, computed by formula 13.16 of Guilford and Fruchter (1981: 235). Approximate confidence intervals are obtained by substituting confidence limits for the within-sets variance, estimated by the method described by Zar (1998: formula 7.16), in the formula.

The formula for the *intraclass correlation coefficient* (Shrout-Fleiss ICC model 1,1, computed from a one-way random effects model ANOVA) is:

$$\text{ICC} = (\text{MSB} - \text{MSW}) / [\text{MSB} + (k - 1)\text{MSW}]$$

where MSB = between-subjects mean square
MSW = within-subjects mean square
k = effective average number of replicates per subject.

The *effective average number of replicates* is computed by formula 5 of Ebel (1951). This provides a value (introduced by Snedecor 1946: 234) that is close to the harmonic mean. The use of Ebel's procedure was suggested by Solomon's rating reliability calculator (Solomon 2004).

Formulae for confidence intervals for the ICC models are provided by McGraw and Wong (1996) in their Table 7, where this ICC is referred to as ICC(1). The number of ratings in the formulae, which as appropriate for studies with a fixed number of replicates, is replaced by the effective average number of replicates.

The *Spearman-Brown prediction formula* (Fleiss 198: 14-15: formula 1.3) for reliability (*R)* is

$$R = \text{N}r / [1 + (\text{N} - 1)r]$$

where N = number of replicates that are averaged
r = intraclass correlation coefficient

Fleiss's formula 1.31 is used to estimate the number of replicates required to obtain a reliability of 0.75 or 0.8:

$$N = P(1 - r) / [r(1 - P)]$$

where P = 0.75 or 0.8

Mis1. EFFECT OF MISCLASSIFICATION: COMPARISON OF CASES AND MATCHED CONTROLS

This module appraises the effect of misclassification (nondifferential or differential) on a comparison of cases and matched controls with respect to their exposure to a risk or protective factor. It demonstrates the effect of the sensitivity and specificity of the measure of exposure, by computing the "true" findings that would give rise to the observed findings..

The program requires entry of the observed frequencies in a paired-data 2x2 table, and estimates of the sensitivity and specificity (in cases and in controls) of the measure of exposure.

The program computes what the frequencies would be if there were no misclassification, i.e. the *"true" frequencies* that would have given rise to the observed finding, together with the *"true" odds ratio* based on the computed frequencies. Confidence intervals are displayed for the observed and "true" odds ratios.

The computed "true" results are not shown if they are unrealistic (if a "true" frequency is negative). A message is displayed saying that the observed frequencies are not compatible with the sensitivity and specificity values, and that if the entries are correct, the findings may represent sampling error.

METHODS

The program constructs a 4 x 4 matrix representing four equations that express the relationship between the observed and true (correctly classified) frequencies, and solves them by calculating the inverse of the matrix and postmultiplying this by a vector composed of the observed frequencies. The procedure, a generalization of Barron's procedure for nondifferential misclassification (Barron 1977), is described by Kleinbaum, Kupper and Morgenstern (1982: 228-236) and Greenland and Kleinbaum (1983). If the matrix is not invertible an error message is displayed..

Exact Fisher's 95% confidence intervals are computed for the odds ratios; the "true" ratio is based on the "true" frequencies, after rounding them off to the nearest integer. The intervals are computed by an algorithm described by Martin and Austin (1991) and using code from David O. Martin's public-domain EXACTBB program. Uncertainty of the sensitivities and specificities is not taken into consideration.

Mis2. EFFECT OF MISCLASSIFICATION: COMPARISON OF MATCHED EXPOSED AND UNEXPOSED SUBJECTS

This module appraises the effect of misclassification (nondifferential or differential) on a comparison of matched subjects exposed and unexposed to a risk or protective factor, where the dependent variable is a disease or some other outcome. It demonstrates the effect of the sensitivity and specificity of the measure of the outcome variable, bycomputing the "true" findings that would give rise to the observed findings..

The program requires entry of the observed frequencies in a paired-data 2x2 table, and estimates of the sensitivity and specificity (in the exposed and unexposed groups) of the measure of the outcome variable..

The program computes what the frequencies would be if there were no misclassification, i.e. the *"true" frequencies* that would have given rise to the observed finding, together with the *"true" odds ratio* based on the computed frequencies. Confidence intervals are displayed for the observed and "true" odds ratios.

The computed "true" results are not shown if they are unrealistic (if a "true" frequency is negative). A message is displayed saying that the observed frequencies are not compatible with the sensitivity and specificity values, and that if the entries are correct, the findings may represent sampling error.

METHODS

The program constructs a 4 x 4 matrix representing four equations that express the relationship between the observed and true (correctly classified) frequencies, and solves them by calculating the inverse of the matrix and postmultiplying this by a vector composed of the observed frequencies. The procedure, a generalization of Barron's procedure for nondifferential misclassification (Barron 1977), is described by Kleinbaum, Kupper and Morgenstern (1982: 228-236) and Greenland and Kleinbaum (1983). If the matrix is not invertible an error message is displayed..

Exact Fisher's 95% confidence intervals are computed for the odds ratios; the "true" ratio is based on the "true" frequencies, after rounding them off to the nearest integer. The intervals are computed by an algorithm described by Martin and Austin (1991) and using code from David O. Martin's public-domain EXACTBB program. Uncertainty of the sensitivities and specificities is not taken into consideration.

Mis3. EFFECT OF MISCLASSIFICATION: COMPARISON OF ANY TWO MATCHED GROUPS

This module appraises the effect of misclassification (nondifferential or differential) on a comparison of any two matched groups with respect to a dependent variable It demonstrates the effect of the sensitivity and specificity of the measure of the dependent variable, by computing the “true” findings that would give rise to the observed findings..

The program requires entry of the observed frequencies in a paired-data 2x2 table, and estimates of the sensitivity and specificity (in groups A and B) of the measure of the dependent variable.

The program computes what the frequencies would be if there were no misclassification, i.e. the *"true" frequencies* that would have given rise to the observed finding, together with the *"true" odds ratio* based on the computed frequencies. Confidence intervals are displayed for the observed and “true” odds ratios.

The computed “true” results are not shown if they are unrealistic (if a “true” frequency is negative). A message is displayed saying that the observed frequencies are not compatible with the sensitivity and specificity values, and that if the entries are correct, the findings may represent sampling error.

METHODS

The program constructs a 4 x 4 matrix representing four equations that express the relationship between the observed and true (correctly classified) frequencies, and solves them by calculating the inverse of the matrix and postmultiplying this by a vector composed of the observed frequencies. The procedure, a generalization of Barron's procedure for nondifferential misclassification (Barron 1977), is described by Kleinbaum, Kupper and Morgenstern (1982: 228-236) and Greenland and Kleinbaum (1983). If the matrix is not invertible an error message is displayed..

Exact Fisher’s 95% confidence intervals are computed for the odds ratios; the “true” ratio is based on the “true” frequencies, after rounding them off to the nearest integer. The intervals are computed by an algorithm described by Martin and Austin (1991) and using code from David O. Martin's public-domain EXACTBB program. Uncertainty of the sensitivities and specificities is not taken into consideration.

P1. POWER OF TEST FOR DIFFERENCE BETWEEN PROPORTIONS (MATCHED PAIRS)

This module computes the power of a McNemar test for a difference between proportions observed in matched subjects, or in the same individuals (as in before-after studies, comparisons of diagnostic procedures, and crossover trials).

The program requires entry of the desired level of significance (for a one-sided or two-sided test), the sample size (the number of pairs of observations), the odds ratio to be detected, and either the expected number or the expected percentage of pairs with discrepant ("yes-no" and "no-yes") results.

Optionally, the percentage of expected losses of pairs in a projected study (nonresponses, dropouts, exclusions from the analysis, etc.) can be entered, and the sample size that is entered will be reduced accordingly before power is computed. This does of course not allow for possible bias. If the expected loss of observations is L%, the expected loss of pairs may be about $2L$ - $[L^2 / 10000]$ %.

Results should be used with caution if samples are very small.

METHODS

Power is computed by the asymptotic unconditional method. The formula is an inversion of formula 3 of Julious *et al*. (1999), and is specified by Sahai & Kurshid (1996b: top of page 562). If an odds ratio under 1 is entered, the computation uses its reciprocal; for this purpose, an odds ratio of 0 is first converted to 0.000001.

If an expected loss rate is entered, the sample size is reduced before power is computed, and so is the expected number of discrepant pairs, if this number was entered.

P2. POWER OF TEST FOR COMPARING DISTRIBUTION OF ORDERED CATEGORIES (MATCHED PAIRS)

This module computes the power of a test (e.g., the Mann-Whitney test for paired data) for a difference between paired observations using an ordinal scale (such as “mild-moderate-severe”). The paired observations may relate to matched subjects, or to the same individuals (as in before-after studies, comparisons of diagnostic procedures, and crossover trials).

The program requires entry of the desired level of significance (for a one-sided or two-sided test), the sample size (the number of pairs of observations), and the odds ratio to be detected. The procedure assumes a proportional odds model; that is, the odds ratio is assumed to be the same, whatever cutting-point may be used when combining adjacent ordered categories to convert the frequency-distribution table into a 2x2 table.

The estimate of power is a conservative one (i.e., it underestimates power), especially if there are many categories.

Optionally, the percentage of expected losses in a projected study (nonresponses, dropouts, exclusions from the analysis, etc.) can be entered, and the sample size that is entered will be reduced accordingly before power is computed. This does of course not allow for possible bias. If the expected loss of observations is L%, the expected loss of pairs may be about $2L$ - $[L^2 / 10000]$ %.

METHODS

The program uses an inversion of the simple “rule-of-thumb” formula recommended by Julious *et al*. (1999: formula 2) for estimating sample size for these tests.

If an expected loss rate is entered, the sample size is reduced before power is computed

P3. POWER OF TEST FOR DIFFERENCE BETWEEN MEANS (MATCHED PAIRS)

This module computes the power of a paired *t*-test for a difference between means observed in paired observations, in matched subjects, or in the same individuals (as in before-after studies, comparisons of diagnostic procedures, and crossover trials).

The program requires entry of the desired level of significance (for a one-sided or two-sided test), the sample size (the number of pairs of observations), and the difference to be detected (e.g. observation A minus observation B). In addition, the standard deviation of the differences between paired values is required. This can be entered, if its value is known or can be assumed. If not, there are two alternatives that permit computation of the standard deviation. These are: (a) entry of the within-subject mean square in an ANOVA (the residual within-subject mean square, after removal of the between-subjects component), if this is known (possibly from a published study; and (b) entry of the standard deviations of the two sets of observations, together with the correlation coefficient between the two sets (if a zero coefficient is entered, this will provide a conservative estimate of sample size).

Optionally, the percentage of expected losses in a projected study (nonresponses, dropouts, exclusions from the analysis, etc.) can be entered, and the sample size that is entered will be reduced accordingly before power is computed. This does of course not allow for possible bias. If the expected loss of observations is L%, the expected loss of pairs may be about $2L - [L^2 / 10000]$ %.

METHODS

The program uses an inversion of formula 1 of Julious *et al*. (1999).

If the standard deviation of the differences is not entered, it is computed either from the within-subject mean square, by multiplying its square root by $\sqrt{2}$ (Julious *et al*. 1999), or from the standard deviations of the two sets of observation (SD_a and SD_b) and the correlation coefficient *(r)*, as

$$\sqrt{(SD_a^2 + SD_b^2 + 2rSD_aSD_b)}$$

(Sokal and Rohlf 1981: 573).

If an expected loss rate is entered, the sample size is reduced before power is computed

S1. SAMPLE SIZES: "YES-NO" DATA: DIFFERENCE (MCNEMAR TEST)

This module computes the sample size (the number of discrepant pairs of observations and the total number of pairs of observations) required for a McNemar test to detect a difference of a given magnitude between paired dichotomous ("yes"/"no") observations in matched subjects or in the same individuals (as in before-after studies, comparisons of diagnostic procedures, and crossover trials). It also computes the numbers of matched sets required for case-control studies with more than one matched control per case.

Three entry options are offered: (a) entry of the odds ratio to be detected and the expected percentage of discrepant ("yes-no" and "no-yes") pairs, or (b) entry of the odds ratio to be detected, the assumed value of the **matching factor** (see below) and the expected proportion of "yes" in the set of observations where that proportion is lower, or (c) the expected proportions of "yes" in both sets of matched observations. The first two options are preferable to the third. In addition, the required significance level and power must be entered.

If the expected proportions of "yes" in the two sets of observations are entered, the computation provides results based on the assumption that that the two sets are mutually independent. The required number of pairs that is reported is a maximal estimate, unless the matched observations are negatively correlated. The stronger the positive correlation, the more the overestimation, as demonstrated in Table 2 of Lehr (2001). If there is a negative correlation (that is, if a "yes" is likely to be associated with a "no" in the matched observation, as might occur in a paired before-after study where the first response influences the second, the computed sample sizes are underestimates. An additional "worst-case" maximal requirement is calculated, for use in such instances.

Optionally, the program will inflate sample sizes to compensate for the probability that not all the selected observations will be included in the analysis, e.g. because of failure to locate addresses, refusal to participate, or missing data. This requires entry of the expected percentage of pairs that will be lost. This inflation does of course NOT compensate for possible selection bias.. If the expected loss of observations is L%, the expected loss of pairs may be about $2L$ - $[L^2 / 10000]$ %.

Matching factor

The matching factor is a measure of the degree to which the two sets of findings are similar because of matching. In a well-matched case-control study similarity may be expected between the exposure status of cases and their matched controls; and in a well-matched cohort study or trial, matched subjects may be expected to be similar with respect to prognostic factors affecting the outcome. The more similar the findings, the larger the sample sizes required.

The matching factor may be derived from the expected 2 x 2 table showing the paired results; it is the product of the two numbers of concordant pairs, divided by the product of the two numbers of discordant pairs. In a case-control study this is the *exposure odds ratio*,

measuring the unconditional association of the exposure status of a case with that of a matched control (Fleiss and Levin 1988, Lachin 1992).

The matching factor is 1 if the findings are independent, and is seldom much more than 2.5 (Fleiss and Levin 1988).

METHODS

If the odds ratio and expected percentage of discrepant pairs are entered, the required number of discrepant pairs is computed by formula 2 of Julious *et al.* (1999), and the required total number of pairs by formula 3 of Julious *et al.* (1999) (formula 5.4 of Sahai & Kurshid 1996b); this is an asymptotic unconditional method that has been shown to approximate satisfactorily to the results of computer simulations (Connett *et al.* 1987).

The same method is used if the expected proportions of "yes" in both sets of observations are entered, after estimating the numbers of pairs with discrepancies in each direction (*S* and *T*) from the proportions of "yes" (*P1* and *P2*), using formulae assuming an independent distribution (Royston 1993; Julious *et al.* 1999: 245):

$S = [P1(1 - P2)]$ and

$T = [P2(1 - P1)]$

and then estimating the odds ratio and proportion of discrepant pairs from *S* and *T*:

$\text{Odds ratio} = S / T$

$\text{Proportion of discrepant pairs} = S + T$

For the "worst-case" estimate,

$S = \min(P1, 1 - P2)$

$T = P2 - P1 + S$

If the matching factor is entered, sample sizes are computed by the multinomial unconditional procedure (Connor 1987; Lachin 1992: formulas 17 and 21), which is slightly conservative. If the calculated sample size is under 30, use is instead made of the local unconditional variance (Mitra 1958, Lachin 1992: formula 19), which is then more accurate. The estimated number of discordant pairs is also displayed.

Sample sizes for case-control studies with more than one matched control per case are calculated by formula 4 of Julious *et al.* (1999).

All sample sizes are rounded up to the next whole number.

If an expected non-inclusion rate (*R*%) is entered, the program multiplies computed sample sizes by

$1 / [1 - (R / 100)]$

before rounding them up

S2. SAMPLE SIZES: “YES-NO” DATA: AGREEMENT (*KAPPA*)

This module computes the sample size required in a study to determine *kappa* for two categories and two sets of observations.

The assumed value of kappa, the assumed proportion of “yes” findings (which is assumed to be similar in both sets of observations), and the required significance level must be entered. In addition, one of the following must be entered: (a) the required *powe*r; (b) the desired *width of the confidence interval* for *kappa* (if the significance level is set at 5%, this refers to the 95% confidence interval); or (c) the desired *lower confidence limit* for *kappa* (if the significance level is set at 5%, this refers to the lower 95% confidence limit).

If power is entered, the program computes the sample sizes required to determine whether a *kappa* of the specified magnitude is significantly higher than 0.4 (taken to mean fair or good agreement) or 0.6 (taken to mean good agreement).

Optionally, the program will inflate sample sizes to compensate for the probability that not all the selected observations will be included in the analysis, e.g. because of failure to locate addresses, refusal to participate, or missing data. This requires entry of the expected percentage of pairs that will be lost. This inflation does of course NOT compensate for possible selection bias.. If the expected loss of observations is L%, the expected loss of pairs may be about $2L$ - [L^2 / 10000] %.

METHODS

If power (1 – *beta*) is entered, the program computes the sample size required to determine whether the lower [(1-*alpha*)*100]% confidence interval of the specified *kappa* exceeds 0.4 or 0.6. Computation is based on a non-centrality parameter that is derived from (1 - *beta*) and (2 x *alpha*), and entered in the sample size formula provided by Donner and Eliasziw (1992).

If the desired width of the confidence interval or the desired lower confidence level is entered, the program uses the procedure described by Donner (1999; formula 2.2).

All sample sizes are rounded up to the next whole number.

If an expected non-inclusion rate (R%) is entered, the program multiplies computed sample sizes by

$$1 / [1 - (R / 100)]$$

before rounding them up

S3. SAMPLE SIZES: “YES-NO” DATA: EQUIVALENCE TEST

This module computes the number of pairs required for a test of the equivalence of two sets of paired “yes-no” observations. This may be useful in the planning of equivalence tests in matched case-control studies, matched-control parallel trials, crossover trials, and comparisons of diagnostic or screening tests.

The program requires entry of the desired significance level and power, the magnitude of the difference (between the proportions of “yes”) that is regarded as negligible, and the expected percentage of discrepant (“yes-no” and “no-yes”) pairs.

Sample sizes are computed for an equivalence test based on the performance of two one-sided tests, and for a one-sided test (e.g. for non-inferiority of a new treatment or screening test in comparison with an established one).

Optionally, the program will inflate sample sizes to compensate for the probability that not all the selected observations will be included in the analysis, e.g. because of failure to locate addresses, refusal to participate, or missing data. This requires entry of the expected percentage of pairs that will be lost. This inflation does of course NOT compensate for possible selection bias.. If the expected loss of observations is L%, the expected loss of pairs may be about $2L$ - $[L^2 / 10000]$ %.

METHODS

The program uses the procedures described by Liu *et al.* (2002) to compute the sample sizes required to test for equivalence, on the assumption that the observed proportions of "yes" in the two sets of observations are identical. Sample sizes are computed for sample-based tests, applying a continuity correction (which increases the required sample size) unless otherwise stated. The computation without a continuity correction uses formula 7 of Liu *et al.*; the computation with a continuity correction requires an iterative process to solve an equation (Liu *et al.* 2002: 239); the van Wijnsgaarden-Bekker-Brent root-solver (Press *et al.* 1989: 283-286) is used for this purpose. Sample sizes for a one-sided test (e.g. a non-inferiority test) are computed in a similar way, with appropriate changes of significance level and power (Liu *et al* 2002: 239). If any computed sample size is too small to ensure at least one discrepant pair in each direction (applying the expected proportion of discrepant pairs *PropDP*), it is raised to $1 / (PropDP / 2)$ to meet this condition.

All sample sizes are rounded up to the next whole number.

If an expected non-inclusion rate (R%) is entered, the program multiplies computed sample sizes by

$$1 / [1 - (R / 100)]$$

before rounding them up

S4. SAMPLE SIZES: ORDERED CATEGORIES: DIFFERENCE

This module computes the number of pairs of observations required for a test to detect a given difference between paired observations using ordered categories (such as "mild-moderate-severe"). The observations may relate to matched subjects, or to the same individuals (as in before-after studies, comparisons of diagnostic procedures, and crossover trials).

The procedure used is a simple "rule-of-thumb" one, and the estimate of sample sizes is a conservative one, especially if there are many categories.*

If the majority of observations are expected to be in a single extreme category (e.g. in the "well" category of a health scale), Julious *et al*. (1999) recommend calling this category "yes" and determining the sample size needed for "yes-no" data (module S1). If there are many categories, they suggest that the data be treated as normally distributed (module S5).

The odds ratio to be detected must be entered, together with the required significance level and power. The procedure assumes a proportional odds model; that is, the odds ratio is assumed to be the same, whatever cutting-point may be used when combining adjacent ordered categories to convert the frequency-distribution table into a paired-data 2x2 table.

Optionally, the program will inflate sample sizes to compensate for the probability that not all the selected observations will be included in the analysis, e.g. because of failure to locate addresses, refusal to participate, or missing data. This requires entry of the expected percentage of pairs that will be lost. This inflation does of course NOT compensate for possible selection bias.. If the expected loss of observations is L%, the expected loss of pairs may be about $2L - [L^2 / 10000]$ %.

* A more exact estimate can be obtained by a procedure provided by the PEPI program SAMPLES, which requires entry (in addition to the odds ratio) of the expected relative distribution of positive-discrepant pairs (pairs with discrepancies consistent in direction with the odds ratio) that have different degrees of discrepancy (Julious and Campbell 1998).

METHODS

The program uses formula 2 of Julious *et al*. (1999, Appendix). The procedure is a simple "rule-of-thumb" one that estimates the number of discordant pairs needed for a two-category situation and takes this as the total number of pairs required for a comparison of ordered categories.

All sample sizes are rounded up to the next whole number.

If an expected non-inclusion rate (R%) is entered, the program multiplies computed sample sizes by

$$1 / [1 - (R / 100)]$$

before rounding them up

S5. SAMPLE SIZES: NUMERICAL DATA: DIFFERENCE (PAIRED *T* TEST)

This module computes the number of pairs of observations required for a paired *t* test to detect a difference of a given magnitude between the means of observations in matched subjects or in the same individuals (such as observations in matched pairs, before-after observations in the same individuals. or cross-over trials) (as in before-after studies, comparisons of diagnostic procedures, and crossover trials).

The difference to be detected (e.g. mean A minus mean B), and the required significance level and power must be entered. The standard deviation of the differences between paired values is also required. This should be entered if its value is known or can be assumed. Alternatively, the program can compute the standard deviation. This requires entry of either (a) the within-subject mean square in an ANOVA (the residual within-subject mean square, after removal of the between-subjects component), if this is known (possibly from a published study); or (b) the known or assumed standard deviations of the two sets of observations, together with the known or assumed correlation coefficient between the two sets (if a zero coefficient is entered, this will provide a conservative estimate of sample size).

Optionally, the program will inflate sample sizes to compensate for the probability that not all the selected observations will be included in the analysis, e.g. because of failure to locate addresses, refusal to participate, or missing data. This requires entry of the expected percentage of pairs that will be lost. This inflation does of course NOT compensate for possible selection bias.. If the expected loss of observations is L%, the expected loss of pairs may be about $2L$ - [L^2 / 10000] %.

Note that for a trial comparing two independent groups, each of them having paired values for each individual (e.g. before and after treatment), module H2 of COMPARE2 should be used, entering the difference to be detected between paired observations, and the standard deviations or variance of the differences between paired observations (Lachin 1981).

METHODS

If the within-subject mean square is entered, its square root is multiplied by √ 2 to obtain the standard deviation (S.D.) of the differences (Julious *et al*. 1999). If the S.D.s of the two sets of observation (SD_a and SD_b) and the correlation coefficient (r) are entered, the S.D. of the differences is calculated (Sokal and Rohlf 1981: 573) as

$$\sqrt{[SD_a^2 + SD_b^2 + 2r(SD_a)(SD_b)]}$$

The required number of pairs is estimated (for a one-sided test) by formula 2.1 of Guenther (1981), and (for a two-sided test) by the same formula using *alpha / 2* instead of *alpha* (formula 1 of Julious *et al*. 1999).

All sample sizes are rounded up to the next whole number. If an expected non-inclusion rate (R%) is entered, the program multiplies computed sample sizes by

$$1 / [1 - (R / 100)]$$

before rounding them up

S6. SAMPLE SIZES: NUMERICAL DATA: AGREEMENT (INTRACLASS CORRELATION COEFFICIENT)

This module computes the sample size required in a study to measure agreement by using an intraclass correlation coefficient (ICC). It may be appropriate in a reliability study in which there are a fixed number (two or more) observations of each subject, or in studies using cluster samples of a fixed size.

The required significance level, the number of observations per subject or set, and the expected ICC must first be entered. Then two options are offered: (a) entry of the required power and the value against which the expected ICC is to be tested; in a reliability study, the latter value is the lowest acceptable ICC; choices that have been suggested are 0.4 (moderate measurement reliability, 0.6 (substantial) or 0.8 (almost perfect); in other studies, it may be zero; and (b) entry of the desired width of the confidence interval for the ICC.

If option (a) is selected, the program uses a simple approximation (Walter *et al.* 1998) whose results have excellent agreement with exact results. It provides the sample size required to test the null hypothesis that the ICC is equal to the value against it is to be tested, against the alternative that it is higher. The method is appropriate for studies in which the ICC can be estimated from an appropriate one-way ANOVA, e.g. those in which each subject is observed by different observers, by different methods, or at different times. Between-subjects and inter-subject variation are taken into account. Walter *et al.* suggest that the method may also be a practical compromise for studies in which a two-way analysis (e.g. taking account of variation between specific observers) would be appropriate.

If option (b) is chosen, the program uses an approximation that Bonett (2002) has developed and shown to be very accurate. This method is appropriate for studies in which the ICC can be estimated from a one-way or two-way ANOVA, e.g. those in which each subject is observed by different observers, by different methods, or at different times, in which between-subjects, inter-subject, and (if necessary) between- observers or between-methods variation must be taken into account

When planning a reliability study, it may be helpful to compare the sample sizes required for different numbers of observations per subject.

Optionally, the program will inflate sample sizes to compensate for the probability that not all the selected observations will be included in the analysis, e.g. because of failure to locate addresses, refusal to participate, or missing data. This requires entry of the expected percentage of pairs or sets that will be lost. This inflation does of course NOT compensate for possible selection bias.. If the expected loss of observations is L%, the expected loss of pairs may be about $2L$ - [L^2 / 10000] %, and the maximal loss of larger sets will be $3L$%.

METHODS

If the required power and the value against which the expected ICC is to be tested are entered, the computation uses formula 12 of Walter *et al*.(1998), with the recommended addition of 0.5 if the number of observations per subject/set is 2.

If the desired width of the confidence interval for the ICC is entered, the computation uses formula 3 of Bonett (2002), with the correction suggested if the number of observations per set is 2 and the expected ICC is 0.7 or more.

All sample sizes are rounded up to the next whole number. If an expected non-inclusion rate (*R*%) is entered, the program multiplies computed sample sizes by

1 / [1 - (R / 100)]

before rounding them up

S7. SAMPLE SIZES: NUMERICALDATA: EQUIVALENCE TEST

This module computes the number of pairs required for a test of the equivalence of the means of two sets of paired numerical observations . This may be useful in the planning of equivalence tests in matched case-control studies, matched-control parallel trials, crossover trials, and comparisons of diagnostic or screening tests.

The program requires entry of the desired significance level and power, the magnitude of the difference (between means) that is regarded as negligible, the mean of the reference set of observations, the expected absolute difference between the means of the two sets (which must be less than the maximum difference regarded as negligible).

The standard deviation of the differences between paired values is also required. This should be entered if its value is known or can be assumed. Alternatively, the program can compute the standard deviation. This requires entry of either (a) the within-subject mean square in an ANOVA (the residual within-subject mean square, after removal of the between-subjects component), if this is known (possibly from a published study); or (b) the known or assumed standard deviations of the two sets of observations, together with the known or assumed correlation coefficient between the two sets (if a zero coefficient is entered, this will provide a conservative estimate of sample size). The standard deviation of the differences (entered or computed) must be less than the mean value in the reference set.

Either set of observations may be chosen as the reference set, but in a study comparing new and established treatments the established treatment is usually selected. In such studies, a recommended definition of a negligible difference is from 0 to 20% of the mean of the reference set. The mean value must be positive. The standard deviation of the differences (entered or computed) must be less than the mean value in the reference set.

Sample sizes are computed for an equivalence test based on the performance of two one-sided tests, and for a single one-sided test (e.g. for non-inferiority of a new treatment in comparison with an established one).

Optionally, the program will inflate sample sizes to compensate for the probability that not all the selected observations will be included in the analysis, e.g. because of failure to locate addresses, refusal to participate, or missing data. This requires entry of the expected percentage of pairs that will be lost. This inflation does of course NOT compensate for possible selection bias.. If the expected loss of observations is L%, the expected loss of pairs may be about $2L$ - [L^2 / 10000] %, and the maximal loss of larger sets will be $3L$%.

METHODS

The program uses the procedure described by Chow and Wang (2001) for a crossover design using raw data. Specifically, it uses the second set of equations designated as "B1" in Appendix B. The required number of pairs is computed by an iterative process, using the van Wijnsgaarden-Dekker-Brent root-solver (Press *et al.* 1989: 283-286). The value 0.2 in Chow and Wang's equations is replaced by D/M, where D is the value entered as the maximum bound of a negligible difference, and M is the mean of the reference set. The same equations

are used to estimate the number of pairs required for a one-sided test, with appropriate changes of significance level and power (Liu *et al*. 2002: 239).

If the standard deviation of the differences is not entered, it is computed either from the within-subject mean square, by multiplying its square root by $\sqrt{2}$ (Julious *et al*. 1999), or from the standard deviations of the two sets of observation (SD_a and SD_b) and the correlation coefficient *(r)*, as

$$\sqrt{(SD_a^2 + SD_b^2 + 2rSD_aSD_b)}$$

(Sokal and Rohlf 1981: 573).

All sample sizes are rounded up to the next whole number. If an expected non-inclusion rate (*R*%) is entered, the program multiplies computed sample sizes by

$$1 / [1 - (R / 100)]$$

before rounding them up

REFERENCES

Abramson JH, Gahlinger PM (2001) Computer programs for epidemiologists: PEPI version 4. Sagebrush Press: Salt Lake City, Utah]

Agresti A (1980) Generalized odds ratios for ordinal data. Biometrics 36: 69-67.

Agresti A (1984) Analysis of ordinal categorical data. New York: John Wiley & Sons.

Agresti A (1996) An introduction to categorical data analysis. New York: Wiley.

Agresti A (1990) Categorical data analysis. New York: Wiley.

Ahn C, Odom-Maryon T (1995) Estimation of a common odds ratio under binary cluster sampling. Statistics in Medicine 14: 1567-1577.

Altman DG (1991) Practical statistics for medical research. London: Chapman and Hall.

Altman DG (1998) Confidence intervals for the number needed to treat. British Medical Journal 317: 1309-1312

Altman DG, Andersen PK (1999) Calculating the number needed to treat for trials where the outcome is time to an event. British Medical Journal 319: 1492-1495.

Altman DG, Machin D, Bryant TN, Gardner MJ, eds. (2000) Statistics with confidence, 2nd edn. BMJ Books.

Armitage P, Berry G, Matthews JNS (2002) Statistical methods in medical research, 4th edn. Oxford: Blackwell Science.

Barlow W, Lai M-Y, Azen SP (1991) A comparison of methods for calculating a stratified kappa. Statistics in Medicine 10: 1465-1472.

Bartko JJ (1994) Measures of agreement: a single procedure. Statistics in Medicine 13: 737-745.

Basu S, Basu A (1995) Comparison of several goodness-of-fit tests for the kappa statistic based on exact power and coverage probability. Statistics in Medicine 14: 347-356.

Bennett BM, Hsu P (1960) On the power function of the exact test for the 2x2 contingency table. Biometrika 47: 393-398.

Bennett EM, Alpert R, Goldstein AC (1954) Communications through limited response questioning. Public Opinion Quarterly 18: 303-308.

Bjerre LM, LeLorier J (2000) Expressing the magnitude of adverse effects in case-control studies: "the number of patients needed to be treated for one additional patient to be harmed". British Medical Journal 320: 503-506.

Bland JM, Altman DG (1995a) Comparing two methods of clinical measurement: a personal history. International Journal of Epidemiology 24 (supp. 1): S7-S14.

Bland JM, Altman DG (1995b) Comparing methods of measurement: why plotting difference against standard method is misleading. Lancet 346: 1085-1087.

Bland JM, Altman DG (1986) Statistical methods for assessing agreement between two methods of clinical assessment. Lancet i: 307-310.

Bland JM, Altman DG (1999) Measuring agreement in method comparison studies. Statistical Methods in Medical Research 8: 136-160.

Bloch DA, Kraemer HC (1989) 2 x 2 kappa coefficients: measures of agreement or association. Biometrics 45: 269-287.

Bonett DG (2002) Sample size requirements for estimating intraclass correlations with desired precision. Statistics in Medicine 21: 1331-1335.

Bowker AH (1948) A test for symmetry in contingency tables. Journal of the American Statistical Association 43: 572-574.

Bradley EL, Blackwood LG (1989) Comparing paired data: a simultaneous test of means and variances. The American Statistician 43: 234-235.

Brenner H, Gefeller O (1994) Chance-corrected measures of the validity of a binary test. Journal of Clinical Epidemiology 47: 627-633.

Breslow NE, Day NE (1087) Statistical methods in cancer research, Vol. II. The design and analysis of cohort studies. Lyon: International Agency for Research on Cancer.

Bristol DR (1989) Sample sizes for constructing confidence intervals and testing hypotheses. Statistics in Medicine 6:803-811.

Burr EJ (1964) Small-sample distributions of the two-sample Cramer-von Mises' W-square and Watson's U-square. Annals of Mathematical Statistics 35: 1091-98.

Byrt T, Bishop J, Carlin JB (1993) Bias, prevalence and kappa. Journal of Clinical Epidemiology 46: 423-429.

Campbell MJ, Gardner MJ (2000) Medians and their differences. In: Altman DG, Machin D, Bryant TN, Gardner MJ, eds (2000) Statistics with confidence, 2nd edn. BMJ Books, pp 36-44.

Casagrande JT, Pike MC, Smith PG (1978a) The power function of the 'exact' test for comparing two binomial distributions. Applied Statistics 27:176-180.

Casagrande JT, Pike MC, Smith PG (1978b) Algorithm AS 129: The power function of the 'exact' test for comparing two binomial distributions. Applied Statistics 27:212-219.

Chatellier G, Zapletal E, Lemaitre D, Menard J, Degoulet P (1996) The number needed to treat: a clinically useful nomogram in its proper context. British Medical Journal 312:426-429.

Chinn S (1990) The assessment of methods of measurement. Statistics in Medicine 9: 351-362.

Chinn S (1991) Repeatability and method comparison. Thorax 46: 454-456.

Chow S-C, Wang H (2001) On sample size calculation in bioequivalence trials. Journal of Pharmacokinetics and Pharmacodynamics 28: 155-169.

Cicchetti DV, Allison T (1971) A new procedure for assessing reliability of scoring EEG sleep recordings. American Journal of EEG Technology 11: 101-109.

Connell FA, Koepsell TD (1985) Measures of gain in certainty from a diagnostic test. American Journal of Epidemiology 121: 744-753.

Connett JE, Smith JA, McHugh RB (1987) Sample size and power for pair-matched case-control studies. Statistics in Medicine 6: 53-59.

Connor RJ (1987) Sample size for testing differences in proportions for the paired-sample design. Biometrics 43: 207-211.

Cox DR, Oakes D (1984) Analysis of survival data. London: Chapman & Hall.

Daly LE (1998) Confidence limits made easy: interval estimation using a substitution method. American Journal of Epidemiology 147: 783-790.

Daniel WW (1995) Biostatistics: a foundation for analysis in the health sciences, 6th edn. New York: John Wiley & Sons.

Darroch JN, McCloud P (1986) Category distinguishability and observer agreement. Australian Journal of Statistics 28: 371-388.

DeMets DL (1987) Methods for combining randomized clinical trials: strengths and limitations. Statistics in Medicine 6: 341-348.

Dietz EJ (1989) Teaching regression in a nonparametric statistic course. The American Statistician 43: 35-40.

Donald A, Donner A (1987) Adjustments to the Mantel-Haenszel chi-square statistic and odds ratio variance estimator when the data are clustered. Statistics in Medicine 6: 491-499.

Donner A (1984) Approaches to sample size estimation in the design of clinical trials - a review. Statistics in Medicine 3: 194-214.

Donner, Allan (1999) `Sample size requirements for interval estimation of the intraclass *kappa* statistic. Communications in Statistics, Part B -- Simulation and Computation 28: 415-429 .

Donner A, Eliasziw M (1992) A goodness-of-fit approach to inference procedures for the *kappa* statistic: confidence interval construction, significance testing and sample size determination. Statistics in Medicine 11: 1511-1519.

Donner A, Eliasziw M, Klar N (1994) A comparison of methods for testing homogeneity of proportions in teratologic studies. Statistics in Medicine 13: 1253-1264.

Donner A, Klar N (1996) The statistical analysis of *kappa* statistics in multiple samples. Journal of Clinical Epidemiology 49: 1053-1058.

Durkalski VL, Palesch YY, Lipsitz SR, Rust PF (2003) Analysis of clustered matched-pair data. Statistics in Medicine 22: 2417-2428.

Ebel RL (1951). Estimation of the reliability of ratings. Biometrika 16: 407-424.

Eliasziw M, Donner A (1991) Application of the McNemar test to non-independent matched pair data. Statistics in Medicine 10: 1981-1991.

Everitt BS (1977) The analysis of contingency tables. London: Chapman and Hall.

Farrington CP, Manning G (1990) Test statistics and sample size formulae for comparative binomial trials with null hypothesis of non-zero risk difference or nun-unity relative risk. Statistics in Medicine 9: 1457-1494.

Feinstein AR (1995) Meta-analysis: statistical alchemy for the 21st century. Journal of Clinical Epidemiology 48: 71-79.

Fieller EC, Hartley HO, Pearson ES (1957) Tests for rank correlation coefficients. I. Biometrika 44: 470-481.

Fieller EC, Hartley HO, Pearson ES (1961) Tests for rank correlation coefficients. II. Biometrika 48: 29-40.

Fleiss JL (1981) Statistical methods for rates and proportions. New York: John Wiley & Sons.

Fleiss JL (1986) The design and analysis of clinical experiments. New York: John Wiley & Sons.

Fleiss JL, Levin B (1988) Sample size determination in studies with matched pairs. Journal of Clinical Epidemiology 41 :727-730.

Freedman LS (1982) Tables of the number of patients required in clinical trials using the logrank test. Statistics in Medicine 1: 121-129.

Gehan E (1965) A generalized Wilcoxon test for comparing arbitrarily single-censored samples. Biometrika 52: 203-223.

Goodman SN, Berlin JA (1994) The use of predicted confidence intervals when planning experiments and the misuse of power when interpreting results. Annals of Internal Medicine 121:200-206, appendix.

Greenland S (1987) Variance estimators for attributable fraction estimates consistent in both large strata and sparse data. Statistics in Medicine 6: 701-708.

Greenland S (1994) Corrections. Statistics in Medicine 13: 99.

Greenland S (1999) Re: "Confidence limits made easy: interval estimation using a substitution method". American Journal of Epidemiology 149: 884.

Greenland S, Kleinbaum DG (1983) Correcting for misclassification in two-way tables and matched-pair studies. International Journal of Epidemiology 12:93-97.

Guenther WC (1981) Sample size formulas for normal theory *t* tests. The American Statistician 35: 243-244.

Guilford JP, Fruchter B (1986) Fundamental statistics in psychology and education, 6th edn, Singapore: McGraw-Hill.

Halperin M, Gilbert PR, Lachin JM (1987) Distribution-free confidence intervals for Pr(X1<X2). Biometrics 43: 71-80.

Hedges LV, Olkin I (1985) Statistical methods for meta-analysis. Orlando: Academic Press.

Higgins JPT, Thompson SG (2002) Quantifying heterogeneity in a meta-analysis. Statistics in Medicine 21: 1539-1558.

Hildebrand DK, Laing JM, Rosenthal H (1977) Analysis of Ordinal Data. Beverly Hills: Sage Publications.

Hirji KF, Tang M-L, Vollset SE, Elashoff RM (1994) Efficient power computation for exact and mid-P tests for the common odds ratio in several 2 x 2 tables. Statistics in Medicine 13: 1539-1549.

Hoehler FK (2000) Bias and prevalence effects on kappa viewed in terms of sensitivity and specificity. Journal of Clinical Epidemiology 53: 499-503.

Hollander M, Wolfe DA (1999) Nonparametric statistical methods, 2nd edn. New York: John Wiley & Sons.

Hsieh FY, Bloch DA, Larsen MD (1998). A simple method of sample size calculation for linear and logistic regression. Statistics in Medicine 17: 1623-1634.

Iman RL, Davenport JM (1980) Approximations of the critical region of the Friedman statistic. Communications in Statistics - Theory and Methods A9: 571-595.

Iyengar S, Greenhouse JB (1988) Selection models and the file drawer problem. Statistical Science 3: 109-117.

Jewell NP (1984) Small-sample bias of point estimators of the odds ratio from matched sets. Biometrics 40: 421-435.

Julious SA, Campbell MJ (1998) Sample size calculations for paired or matched ordinal data. Statistics in Medicine 17: 1635-1642.

Julious SA, Campbell MJ, Altman DG (1999) Estimating sample sizes for continuous, binary, and ordinal outcomes in paired comparisons: practical hints. Journal of Biopharmaceutical Statistics 9: 241-251.

Kahn HA, Sempos CT (1989) Statistical methods in epidemiology. New York: Oxford University Press.

Kendall MG (1970) Rank correlation methods, 4th edn. London: Griffin.

Kim J S (1997) Determining sample size for testing equivalence. Medical Device and Diagnostic Industry Magazine. Available on the Internet at www.devicelink.com/archive/97/020.html

Kaplan EL, Meier P (1958) Nonparametric estimation from incomplete observations. Journal of the American Statistical Association 53: 457-481.

Kleinbaum DG, Kupper LL, Morgenstern H (1982) Epidemiological research: principles and quantitative methods. New York: Van Nostrand Reinhold.

Kraemer HC, Bloch DA (1988) Kappa coefficients in epidemiology: an appraisal of a reappraisal. Journal of Clinical Epidemiology 41:959-968.

Kraemer HC, Periyakoil VS, Noda A (2002) Tutorial in biostatistics: kappa coefficients in medical research. Statistics in Medicine 21: 2109-2129.

Kuritz SJ, Landis JR (1987) Attributable risk ratio estimation from matched-pairs case-control data. American Journal of Epidemiology 125: 324-328.

Lachin JM (1981) Introduction to sample size determination and power analysis for clinical trials. Controlled Clinical Trials 2: 93-113.

Lantz CA, Nebenzahl E (1996) Behavior and interpretation of the *kappa* statistic: resolution of the two paradoxes. Journal of Clinical Epidemiology 49: 431-434.

Lee J (1992) Evaluating agreement between two methods for measuring the same quantity: a response. Computers in Biology and Medicine 22: 369-371.

Lee Y, Wilkens L (1994) Comparing means based on generalized matched sampling. Psychiatry Research 54: 305-306.

Lee W-C (1999) Selecting diagnostic tests for ruling out or ruling in disease: the use of the Kullback-Leibler distance. International Journal of Epidemiology 28: 521-525.

Lehr RG (2001) Some practical considerations and a crude formula for estimating sample size for McNemar's test. Drug Information Journal 35 :1227-1233

Lin L I-K (1989) A concordance correlation coefficient to evaluate reproducibility. Biometrics 45: 255-268.

Lin L I-K (2000) A note on the concordance correlation coefficient. Biometrics 56: 324-325.

Lin L I-K, Chinchilli V (1997) Rejoinder to the letter to the editor from Atkinson and Nevill. Biometrics 53: 777-778.

Liu G (2000) Sample size for epidemiologic studies. In: Gail MH, Benichou J (eds) Encyclopedia of epidemiologic methods, Chichester: John Wiley and Sons, pp. 777-794.

Liu J-P, Hsueh H-M, Hsieh E, Chen JJ (2002) Tests for equivalence or non-inferiority in paired binary data. Statistics in Medicine 21: 231-245.

Lui K-J (1996) Notes in case-control studies with matched pairs under inverse sampling. Biometrical Journal (1996) 38: 681-693

Lui K-J (2001a) Interval estimation of the attributable risk in case-control studies with matched pairs. Journal of Epidemiology and Community Health 55: 885-890.

Lui K-J (2001b) Notes on testing equality in dichotomous data with matched pairs. Biometrical Journal 43: 313-321.

Lui K-J (2004) Statistical evaluation of epidemiological risk. Chichester: John Wiley & Sons.

Machin D, Gardner MJ (2000) Time to event studies. In: Altman DG, Machin D, Bryant TN, Gardner MJ, eds (2000) Statistics with confidence, 2nd edn. BMJ Books, pp 93-194.

MacLure M, Willett WC (1987) Misinterpretation and misuse of the *kappa* statistic. American Journal of Epidemiology 126: 161-169.

Mantel N (1963). Chi-square tests with one degree of freedom: extensions of the Mantel-Haenszel procedure. American Statistical Association Journal 58: 690-700.

Martin D, Austin H (1991) An efficient program for computing conditional maximum likelihood estimates and exact confidence limits for a common odds ratio. Epidemiology 2: 359-362.

Martin DO, Austin H (1996) Exact estimates for a rate ratio. Epidemiology 7: 29-33.

Maxwell AE (1970) Comparing the classification of subjects by two independent judges. British Journal of Psychiatry 116: 651-655.

Maxwell WE (1977) Coefficients of agreement between observers and their interpretation. British Journal of Psychiatry 130: 79-83.

McGraw KO, Wong SP (1996) Forming inferences about some intraclass correlation coefficients. Psychological Methods 1 :30-46.

Mehta C, Patel N (1991) StatXact Statistical software for exact nonparametric inference: User manual version 2. Cambridge MA: Cytel Software Corporation.

Mitra SK (1958) On the limiting power function of the frequency chi-square test. Annals of Mathematical Statistics 29: 1221-1233.

Morris JA, Gardner MJ (2000) Epidemiological studies. In: Altman DG, Machin D, Bryant TN, Gardner MJ. Statistics with confidence, 2nd edn. BMJ Books.

Morrison AS (1979) Sequential pathogenic components rates. American Journal of Epidemiology 108: 709-718.

Mueller R, Buttner P (1994) A critical discussion of intraclass correlation coefficients. Statistics in Medicine 13:2465-2476.

Nam J-M (1997) Establishing equivalence of two treatments and sample size requirements in matched-pair design. Biometrics 53: 1422-1430.

Newcombe RG (1998a) Interval estimation for the difference between independent proportions: comparison of eleven methods. Statistics in Medicine 17: 873-890.

Newcombe RG (1998b) Improved confidence intervals for the difference between binomial proportions based on paired data. Statistics in Medicine 17: 2635-2650.

Newcombe RG. Altman DG (2000) Proportions and their differences. In: Altman DG, Machin D, Bryant TN, Gardner MJ. Statistics with confidence, 2nd edn. BMJ Books, pp. 45-56.

O'Brien PC, Fleming TR (1987) A paired Prentice-wilcoxon test for censored paired data. Biometrics 43: 169-180.

Obuchowski NA (1998) On the comparison of correlated proportions for clustered data. Statistics in Medicine 17: 1495-1507.

Orwin R (1983) A fail-safe N for effect size in meta-analyses. Journal of Educational Statistics 8: 157-159.

Overall JE (1990) Comment. Statistics in Medicine 9:379-82.

Pike MC (1972) Contribution to the discussion on the paper by Peto R and Peto J: Asymptotically efficient rank invariant test procedures. Journal of the Royal Statistical Society, Series A, 135:201-203.

Peat JK, Unger WR, Combe D (1994) Measuring changes in logarithmic data, with special reference to bronchial responsiveness. Journal of Clinical Epidemiology 47: 1099-1108.

Peto R, Pike MC, Armitage P, Breslow NE, Cox DR, Howard SV, Mantel N. McPherson K, Peto J, Smith PG (1977) Design and analysis of randomized clinical trials requiring prolonged observation of each patient. II. Analysis and examples. British Journal of Cancer 35: 1-39.

Pike MC, Casagrande J, Smith PG (1975) Statistical analysis of individually matched case-control studies in epidemiology: factor under study a discrete variable taking multiple values. British Journal of Preventive and Social Medicine 29: 196-201.

Prentice RL (1978) Linear rank tests with right censored data/. Biometrika 65: 167-179.

Press WH, Flannery BP, Teukolsky SA, Vetterling WT (1989) Numerical recipes in Pascal: the art of scientific computing. Cambridge: Cambridge University Press.

Rao JNK, Scott AJ (1992) A simple method for the analysis of clustered binary data. Biometrics 48: 527-585.

Rockhill B, Newman B, Weinbert C (1998) Use and misuse of population attributable fractions. American Journal of Public Health 88: 15-19.

Roebruck P, Kuhn A (1995) Comparison of tests and sample size formulae for proving therapeutic equivalence based on the difference of binomial probabilities. Statistics in Medicine 14: 1583-1594.

Rosner B (1982) A gneralization of the paired *t*-test. Applied Statistics 31: 9-13.

Rothman KJ (1986) Modern epidemiology. Boston: Little, Brown & Co.

Rothman KJ and Greenland S (1998) Modern epidemiology, 2nd edn. Philadelphia: Lippincott-Raven.

Royston P (1993) A pocket-calculator algorithm for the Shapiro-Francia test for non-normality: an application to medicine. Statistics in Medicine 12: 181-184.

Sackett DL, Richardson WS, Rosenberg W, Haynes RB (1997) Evidence-based medicine: how to practice and teach EBM. New York: Churchill Livingstone.

Sahai H, Khurshid A (1996a) Formulae and tables for the determination of sample sizes and power in clinical trials for testing differences in proportions for the two-sample design: a review. Statistics in Medicine 15: 1-21.

Sahai H, Khurshid A (1996b) Formulae and tables for the determination of sample sizes and power for testing differences in proportions for the matched-pair design: a review. Fundamental and Clinical Pharmacology 20: 554-563.

Salmi LR (1986) Re: Measures of gain in certainty from a diagnostic test (letter). American Journal of Epidemiology 123: 1121-1122.

Samsa GP (1996) Sampling distributions of p_pos and p_neg. Journal of Clinical Epidemiology 49 :917-919.

Satten GA, Kupper LL (1990) Sample size requirements for interval estimation of the odds ratio. American Journal of Epidemiology 131:177-184.

Schoenfeld DA (1983) Sample size formula for the proportional-hazards regression model. Biometrics 39: 499-503.

Schuirmann DJ (1987) A comparison of the two one-sided tests procedure and the power approach for assessing the equivalence of average bioavailability. Journal of Pharmacokinetics and Biopharmaceutics 14: 657-580).

Scott WA (1955) Reliability of content analysis: The case of nominal scale coding. Public Opinion Quarterly 19:321-325.

Selvin S (1996) Statistical analysis of epidemiologic data, 2nd edn. New York: Oxford University Press.

Sen PK (1968) Estimates of the regression coefficient based on Kendall's tau. Journal of the American Statistical Association 63: 1379-1389.

Shiue W-K, Bain, LJ (1982) Experiment size and power comparisons for two-sample Poisson tests. Applied Statistics 31: 130-134.

Shoukri MM, Pause CA (1999) Statistical methods for health sciences, 2nd edn. Boca Raton: CRC Press.

Shrout PE, Fleiss JL (1979). Intraclass correlations: uses in assessing rater reliability.
Psychological Bulletin 86: 20-428

Siegel S, Castellan NJ Jr (1988) Nonparametric statistics for the behavioral sciences, 2nd edn. New York: McGraw-Hill.

Simon R (1986) Confidence intervals for reporting results of clinical trials. Annals of Internal Medicine 105: 429-435.

Sinclair JC, Bracken MB (1994) Clinically useful measures of effect in binary analyses of randomized trials. Journal of Clinical Epidemiology 47: 881-889.

Smeeth L, Haines A, Ebrahim S (1999) Numbers needed to treat derived from meta-analyses - sometimes informative, usually misleading. British Medical Journal 318: 1548-51.

Snedecor GW (1946) Statistical methods, 4th edn. Ames, Iowa: Iowa State College Press.

Sokal RF, Rohlf FJ (1981) Biometry, 2nd edn. New York: W.H. Freeman.

Solomon DJ (2004). The rating reliability calculator. BMC Medical Research Methodology 4: 11.

Sprent P (1993) Applied nonparametric statistical methods, 2nd edn. London: Chapman & Hall.

Steinley D, Wood P (2000). ICC.sas – program to calculate intra-class correlations and confidence intervals. . Internet document: http://www.missouri.edu/~marc/icc2.sas

St Laurent RT (1998) Evaluating agreement with a gold standard in method comparison studies. Biometrics 54: 537-545.

Stouffer SA, Suchman EA, De Vinney LC, Star SA, Williams RM Jr (1949) The American soldier: adjustment during army life, vol. 1. New Jersey: Princeton University Press.

Tate MW, Brown SM (1970) Note on the Cochran Q test. Journal of the American Statistical Association 65:155-160.

Theil H (1950) A rank-invariant method of linear and polynomial regression analysis. III. Koninklijke Nederlandse Akademie Van Wetenschappen, Proceedings, Series A, 53: 1397-1412.

Thode HC Jr (1997) Power and sample size requirements for tests of differences between two Poisson rates. The Statistician 46: 227-230.

Thompson WF, Walter SD (1988a) A reappraisal of the kappa coefficient. Journal of Clinical Epidemiology 41 :949-958.

Thompson WF, Walter SD (1988b) *Kappa* and the concept of independent errors. Journal of Clinical Epidemiology 41 :949-958.

Walter SD (1980) Matched case-control studies with a variable number of controls per case. Applied Statistics 28: 172-179.

Walter SD (2001) Number needed to treat (NNT): estimation of a measure of clinical benefit. Statistics in Medicine 20: 3947-3962.

Walter SD, Eliasziw M, Donner A (1998) Sample size and optimal designs for reliability studies. Statistics in Medicine 17: 101-110.

Westlake W J (1973) The design and analysis of comparative blood- level trials. In: Swarbrick J (ed.) Current concepts in the pharmaceutical sciences: dosage form design and bioavailability. Philadelphia: Lea & Febiger.

Whitehead J (1993) Sample size calculations for ordered categorical data. Statistics in Medicine 12: 2257-2271.

Wikipedia, the free encyclopedia. Available on the Internet at http://en.wikipedia.org/wiki/Spearman-Brown_prediction_formula

Wilson EB (1927). Probable inference, the law of succession, and statistical inference. Journal of the American Statistical Association 22: 209-212.

Wolf FM (1986) Meta-analysis: quantitative methods for research synthesis. Beverly Hills: Sage Publications.

Woolson RF, Bean JA, Rojas PB (1986) Sample size for case-control studies using Cochran's statistic. Biometrics 42: 927-932.

Woolson RF, O'Gorman TW (1992) A comparison of several tests for censored paired data. Statistics in Medicine 11: 193-208.

Yanagawa T, Tango T, Hiejima Y (1994) Mantel-Haenszel-type tests for testing equivalence or more than equivalence in comparative clinical trials. Biometrics 50: 859-864; erratum note in Biometrics 1995 51:392

Zar JH (1998) Biostatistical analysis, 4th edn. Prentice Hall.
